# Homology Modeling of Dissimilatory APS Reductases (AprBA) of Sulfur-Oxidizing and Sulfate-Reducing Prokaryotes

**DOI:** 10.1371/journal.pone.0001514

**Published:** 2008-01-30

**Authors:** Birte Meyer, Jan Kuever

**Affiliations:** Max Planck Institute for Marine Microbiology, Bremen, Germany; Cairo University, Egypt

## Abstract

**Background:**

The dissimilatory adenosine-5′-phosphosulfate (APS) reductase (cofactors flavin adenine dinucleotide, FAD, and two [4Fe-4S] centers) catalyzes the transformation of APS to sulfite and AMP in sulfate-reducing prokaryotes (SRP); in sulfur-oxidizing bacteria (SOB) it has been suggested to operate in the reverse direction. Recently, the three-dimensional structure of the *Archaeoglobus fulgidus* enzyme has been determined in different catalytically relevant states providing insights into its reaction cycle.

**Methodology/Principal Findings:**

Full-length AprBA sequences from 20 phylogenetically distinct SRP and SOB species were used for homology modeling. In general, the average accuracy of the calculated models was sufficiently good to allow a structural and functional comparison between the beta- and alpha-subunit structures (78.8–99.3% and 89.5–96.8% of the AprB and AprA main chain atoms, respectively, had root mean square deviations below 1 Å with respect to the template structures). Besides their overall conformity, the SRP- and SOB-derived models revealed the existence of individual adaptations at the electron-transferring AprB protein surface presumably resulting from docking to different electron donor/acceptor proteins. These structural alterations correlated with the protein phylogeny (three major phylogenetic lineages: (1) SRP including LGT-affected Archaeoglobi and SOB of Apr lineage II, (2) crenarchaeal SRP *Caldivirga* and *Pyrobaculum*, and (3) SOB of the distinct Apr lineage I) and the presence of potential APS reductase-interacting redox complexes. The almost identical protein matrices surrounding both [4Fe-4S] clusters, the FAD cofactor, the active site channel and center within the AprB/A models of SRP and SOB point to a highly similar catalytic process of APS reduction/sulfite oxidation independent of the metabolism type the APS reductase is involved in and the species it has been originated from.

**Conclusions:**

Based on the comparative models, there are no significant structural differences between dissimilatory APS reductases from SRP and SOB; this might be indicative for a similar catalytic process of APS reduction/sulfite oxidation.

## Introduction

ATP sulfurylase, adenosine-5′-phosphosulfate (APS) reductase and sulfite reductase mediate the process of dissimilatory sulfate reduction and, therefore, represent key enzymes in the energy metabolism of the phylogenetically diverse sulfate-reducing prokaryotes (SRP). Sulfate is activated to APS by ATP sulfurylase (Sat) at the expense of ATP; hereafter, APS reductase (Apr) converts APS to AMP and sulfite which is subsequently reduced to sulfide by the activity of sulfite reductase (Dsr) [1]–[4]. Homologous proteins have been shown to exist in several anoxygenic phototrophic and chemotrophic, facultative anaerobic sulfur-oxidizing bacteria (SOB) [3], [5]–[11] in which they were suggested to operate in the reverse direction oxidizing sulfide to sulfate. In SRP as well as in SOB, these enzymes appeared to be located in the cytoplasm or at the cytoplasmic site of the inner membrane [3], [7], [8], [10], [11].

The enzyme APS reductase has been investigated from several SRP and SOB with respect to the molecular parameters, e.g. mass, subunit composition and cofactor stoichiometry; the results confirmed its general conformity although the enzyme was involved in different metabolic pathways (oxidative/reductive) and was isolated from phylogenetically distant species [7], [8], [12], [13]. Its functional unit is suggested to be a 1∶1 heterodimeric complex composed of a FAD-containing alpha-subunit with a molecular mass of 70–75 kDa and a two [4Fe-4S] clusters-containing beta-subunit of 18–23 kDa [12], [13]. There is increasing evidence that a quinone-interacting membrane-bound oxidoreductase, QmoABC, represents the native electron donor in SRP [14], [15]. Recent phylogenetic analyses of AprBA sequences of sulfate-reducers and sulfur-oxidizers demonstrated that their encoding genes, *aprBA*, have been frequently affected by lateral gene transfer (LGT) events [16], [17] which are reflected in the existence of two distantly related oxidative APS reductase types in SOB: Most members of the *Chromatiaceae* possess a homologue (SOB Apr lineage I) distinct to the reductive protein type of the SRP, whereas members of certain SOB families and genera, e.g. *Chlorobiaceae* and betaproteobacterial *Thiobacillus*, harbor SRB-related, putatively LGT-acquired *aprBA* genes (SOB Apr lineage II) [17]. Genome data demonstrated that the composition/arrangement of the *apr* gene loci (occurrence of *qmoABC* or *aprM* genes) of the distinct SOB lineages I and II correlates with the AprBA phylogeny. Based on the concomitant presence of the *apr* and *qmo* genes, the Qmo redox complex has been suggested to act as a functional link between the APS reductase and the membrane quinone pool also in those SOB that harbor SRP-related *apr* gene loci (SOB Apr lineage II) [17] as proposed for the SRP [14]. The native electron donor of the SOB Apr lineage I type APS reductase is still unknown; interestingly, the corresponding genes are always co-transcribed with a conserved ORF, *aprM*, coding for a membrane-integral protein [17].

**Figure 1:**
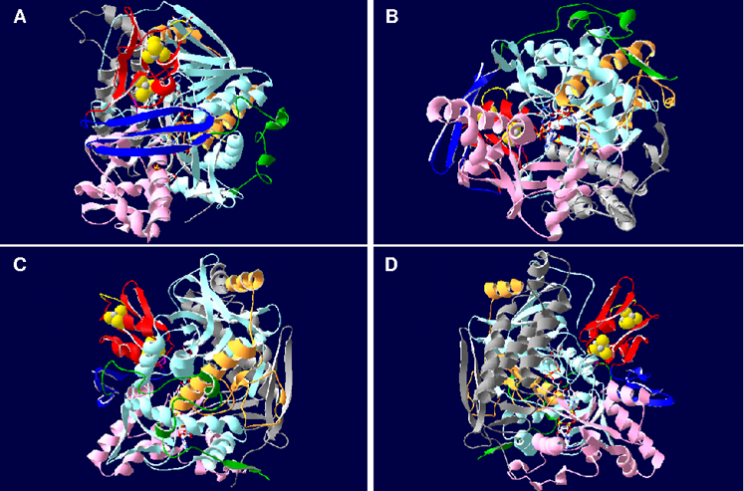
Three-dimensional ribbon structure of APS reductase from *A. fulgidus.* The beta-subunit segments are colored red (ferredoxin segment), blue (3 antiparallel beta-sheets segment), and green (tail segment); the alpha-subunit domains are colored light blue and orange (FAD-binding domain I and II), pink (capping domain), and grey (helical domain). The [4Fe-4S] clusters, FAD and substrate APS are shown as ball-and-stick representations; tryptophan Trp-B48 of AprB is highlighted by violet color. Ribbon structure is shown from (A) top view, (B) bottom view (substrate channel), (C) front view, and (D) back view.

The X-ray structure of the dissimilatory APS reductase isolated from *Archaeoglobus fulgidus* has been elucidated at 1.6 Å resolution by Fritz and coworkers [18]: Its beta-subunit can be subdivided in three segments comprising a bacterial ferredoxin-like segment that envelopes both [4Fe-4S] clusters (amino acids B1-B68), followed by a three-stranded antiparallel beta-sheet (B69-B104) and a tail with a length of 50 Å (B105-B148) (see Fig. 1). The structure of the alpha-subunit can be grouped into the FAD cofactor-binding (amino acids A2-A261 and A394-A487), the capping (A262-A393) and the helical domains (A488-A643) (see Fig. 1); its overall structure classifies this subunit of the APS reductase as member of the fumarate reductase family [18]–[20]. The global part of the beta-subunit is embedded into a broad cleft of the alpha-subunit, while its long tail wraps around the latter increasing the contact surface between both subunits [18] (see Fig. 1). The reaction mechanism of APS reductase has been under debate [21], [22]; Schiffers and coworkers recently determined the X-ray structures of *A. fulgidus* APS reductases in different enzymatic states [23] that confirmed the proposed catalytic mechanism via a nucleophilic attack of the N5 atom of reduced FAD on the sulfur of APS. A covalent FAD-APS intermediate is formed that decomposes spontaneously to AMP and to the FAD-sulfite adduct which is subsequently cleaved, and sulfite is finally liberated [23], [24]. The two electrons required for the reduction of APS were postulated to be transferred one by one over 30 Å via [4Fe-4S] cluster II at the surface of the protein, cluster I and Trp-B48 to the isoalloxazine ring of the buried FAD [18], [23], [24] (see Fig. 1).

The aim of this study was to investigate the structural differences of oxidative and reductive APS reductases present in the phylogenetically and physiologically diverse SRP and SOB based on homology modeling-built protein models. The theoretical protein structure prediction method homology (or comparative) modeling [25]–[27] relies on the observation that the structural conformation of a protein is more highly conserved than its amino acid sequence, and that small or medium changes in sequence typically result in only small changes in the 3D structure [28]. In general, reliable homology models are constructed if the sequence identity exceeds 30% [25]–[27]. The results are discussed in context to the AprBA-based phylogeny and the presence/absence of the Qmo redox complex-encoding genes.

## Analysis

### Sequence analysis and phylogenetics

PSI-BLAST (www.ncbi.nlm.nih.gov/BLAST/) was used to search the non-redundant version of current public databases of the National Center for Biotechnology Information (NCBI) for homologous, full-length sequences of QmoABC and AprBA. The AprBA sequences were implemented into the persisting multiple alignment of SRP- and SOB-derived sequences [16], [17] using the Bioedit (version 7.0.5) sequence alignment editor (http://www.mbio.ncsu.edu/BioEdit/bioedit.html). The QmoA, QmoB, and QmoC proteins were automatically aligned with the web server 3DCoffeeigs (http://igs-server.cnrs-mrs.fr/Tcoffee/) [29], [30]; the initial alignments were refined manually by visual inspection. Phylogenetic analysis of the AprBA, QmoA, QmoB, and QmoC data sets (119, 21, 22, and 18 sequences) was performed with the online version of PhyML (http://atgc.lirmm.fr/phyml) [31]. Maximum likelihood-based phylogenetic trees were constructed using the global rearrangement and randomized species input order options as well as the JTT matrix as amino acid replacement model; regions of insertions and deletions (indels) were omitted from the calculations yielding finally 701, 392, 715, and 357 compared amino acid positions, respectively. Statistical support for the protein trees is given by bootstrap analysis with 100 resamplings.

### Homology modeling

The AprBA sequences of *Pyrobaculum calidifontis* (YP_929769-70), *Pyrobaculum aerophilum* (NP_560098-101), *Caldivirga maquilingensis* (ZP_01711818-9), *Chlorobaculum tepidum* (NP_661758-9), *Pelagibacter ubique* HTCC1062 (YP_266256-7), uncultured alphaproteobacterium EBAC2C11 (AAV31645-6), *Thiobacillus denitrificans* ATCC 25259 (YP_314630-1, YP_316040-1), *Allochromatium vinosum* (AAC23620-1), *Candidatus* Ruthia magnifica (YP_903357-8), *Desulfovibrio vulgaris* str. Hildenborough (YP_010067-8), *Desulfovibrio desulfuricans* ATCC 29577 (AAF36689-90), *Desulfotalea psychrophila* (YP_064840-1), *Desulfobulbus* sp. str. MLMS-1 (ZP_01288426-7), *Olavius algarvensis* Delta1 symbiont (AASZ_01000974), *Syntrophobacter fumaroxidans* (YP_845176-7), uncultured sulfate-reducing bacterium fosws7f8 and fosws39f7 (CAJ31201-2; CAJ31179-80) [32], *Desulfotomaculum reducens* str. MI-1 (ZP_011499890-1), *Thermodesulfobacterium commune* and *Thermodesulfovibrio yellowstonii* (genome sequencing database of The Institute of Genome Research, http://www.tigr.org) were selected to create three-dimensional models of dissimilatory APS reductases from a phylogenetically and physiologically diverse range of SRB and SOB. Comparative modeling by assembly of rigid bodies [25]–[27] was performed with the SWISS-MODEL/ProModII online server for automated protein homology modeling (http://swissmodel.expasy.org) [33], [34] using the recently determined AprBA structures of *Archaeoglobus fulgidus* [18], [23] as templates (Brookhaven Protein Data Bank, PDB, entries 1jnrA/B, 2FJA, 2FJB, 2FJD, 2FJE). The input alignments (SWISS-MODEL “alignment mode”) were based on a structural multiple alignment calculated by 3DCoffee which was manually refined to result in optimal pair wise target-template alignments (to be modeled SOB/SRB AprBA versus the *A. fulgidus* sequence). Model construction by the ProModII program included complete backbone and side chain building, loop building, verification of model quality, including packing, and subsequent energy minimization [33], [34] using the Gromos96 force field [35]. The stereochemical and energetic parameters of the initial 3D protein models were evaluated by the WHATCHECK [36], PROSAII [37], ANOLEA [38], and Verify3D [39] analysis reports provided by the SWISS-MODEL server. The protein structure visualization program DeepView (Swiss-PdbViewer, version 3.7.; available from the ExPASy server http://www.expasy.org/spdbv) [33], [40] was used to optimize the placement of the insertions and deletions considering the template structure context and conservation of structural features with a functional role. The “project mode” of SWISS-MODEL was used to iteratively improve the output 3D models of each SOB and SRB species on the basis of the evaluation software embedded within ProModII. Docking of the [4Fe-4S] clusters, FAD cofactor and substrate APS into the structure of modeled dissimilatory APS reductase was performed by superimposition with the template. The quality of the final model was determined from the B-score values provided by SWISS-MODEL and the root mean square (RMS) deviation values between equivalenced atoms in the amino acids of modeled and template protein. The models were also analyzed for violations of main chain Phi/Psi dihedral bond angle ratios and backbone/side chain steric conflicts. The molecular surface including the electrostatic potential as well as the accessible surface areas were calculated using the corresponding tools implemented into the DeepView program. The figures were generated using the Render3D mode of DeepView.

## Results and Discussion

In general, the average homology model accuracy is a function of the template-target sequence similarity: The dispersion of the model-target structural overlap increases with the decrease in sequence identity between template and target. Errors in models can be divided into five categories: (1) errors in side chain packing, (2) distortions and shifts of a region that is aligned correctly with the template structure, (3) distortions and shifts of a region that does not have an equivalent segment in any of the template structures, (4) distortions and shifts of a region that is aligned incorrectly with the template structures, and (5) a misfolded structure that results from using an incorrect template. Errors 3–5 are relatively infrequent when sequences with more than 40% identity to the templates are modeled; in such a case, approximately 90% of the main chain atoms are likely to be modeled with an RMS deviation of about 1 Å between template and target proteins; the structural differences are usually limited to loops and side chains [25]–[27]. Overall, these models are almost as good as medium-resolution experimental structures, because the proteins at this level of similarity are likely to be as similar to each other as are the structures for the same protein determined by different experimental techniques under different conditions, see [25]–[27] and references therein. When the sequence identity is between 30 and 40%, the structural differences become larger resulting in an increase of the main chain RMS errors to about 1.5 Å for about 80% of residues. The rest of the residues are modeled with large errors because the homology modeling methods generally fail to model structural distortions and rigid body shifts, and are unable to recover from misalignments [25]–[27].

**Figure 2:**
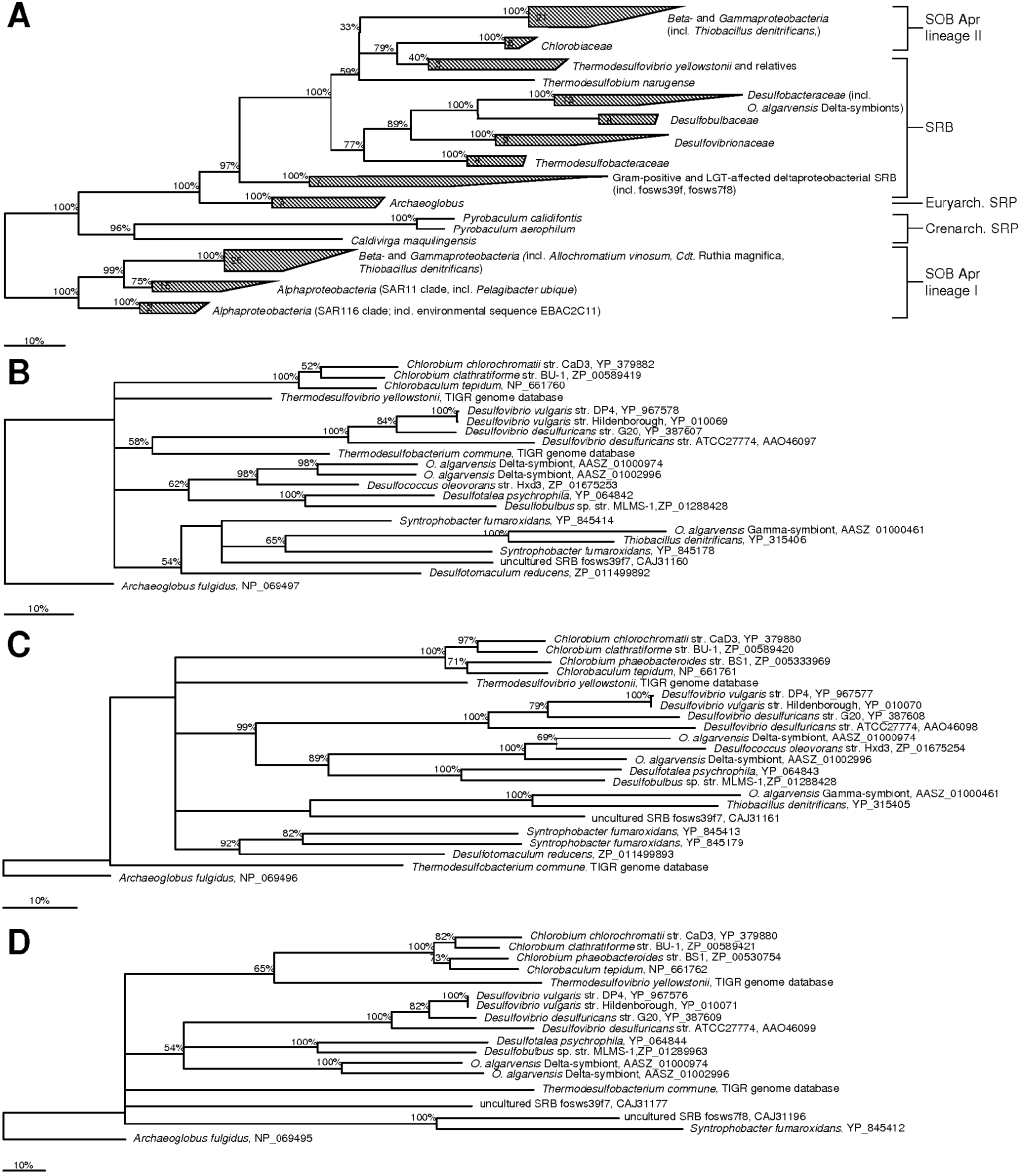
Phylogenetic trees based on (A) AprBA, (B) QmoA, (C) QmoB, and (D) QmoC sequences. The trees were inferred using PhyML (maximum likelihood method). The SOB Apr lineage-I sequence group (A) and the *Archaeglobus fulgidus* QmoABC sequences (B–D) were used as outgroup, respectively. The scale bar corresponds to 10% estimated sequence divergence. Branching orders that were only supported by bootstrap resampling values below 50% are shown as multifurcations; percentages greater than 50% of bootstrap resampling supporting a topological element are indicated near the nodes.

In this study, full-length Apr sequences from 20 phylogenetically distinct SRP and SOB species were used for homology modeling that ranged in their sequence identity values to the *A. fulgidus* templates between 38.6 to 62.6% (beta-subunit) and 47.6 to 60.7% (alpha-subunit) (see supplementary data material Table S1 and S3). The lowest identity values were found for the sequences of SOB Apr lineage-I members, e.g. *Pelagibacter ubique*, (AprB: 38.6 to 45.8%; AprA: 47.7 to 51.5%) and the sequences of crenarchaeal *Pyrobaculum* spp. (38.7 to 42.4%; AprA: 47.6%) which is in accordance to the AprBA phylogeny (see Fig. 2A). The APS reductases of SRB species possessed sequence identities to the *A. fulgidus* templates that ranged between 53.1 to 62.6% (AprB) and 49.5 to 60.7% (AprA) with the highest values received for the Gram-positive SRB and the LGT-affected deltaproteobacterial members [16]; the APS reductases of SOB Apr lineage II had 49.0 to 61.4% (AprB) and 50.2 to 54.0% (AprA) sequence identity to the templates. The overall accuracy of all AprA and most AprB comparative models could be assumed to be sufficiently good to allow their structural and functional comparison (in the models, 78.8 to 99.3% (AprB) and 89.5 to 96.8% (AprA) of the main chain atoms had RMS deviations below 1 Å, see supplementary data material Table S1 and S3).

### Structural comparison of the beta-subunit (AprB) comparative models of the dissimilatory APS reductase among SRP and SOB

**Figure 3:**
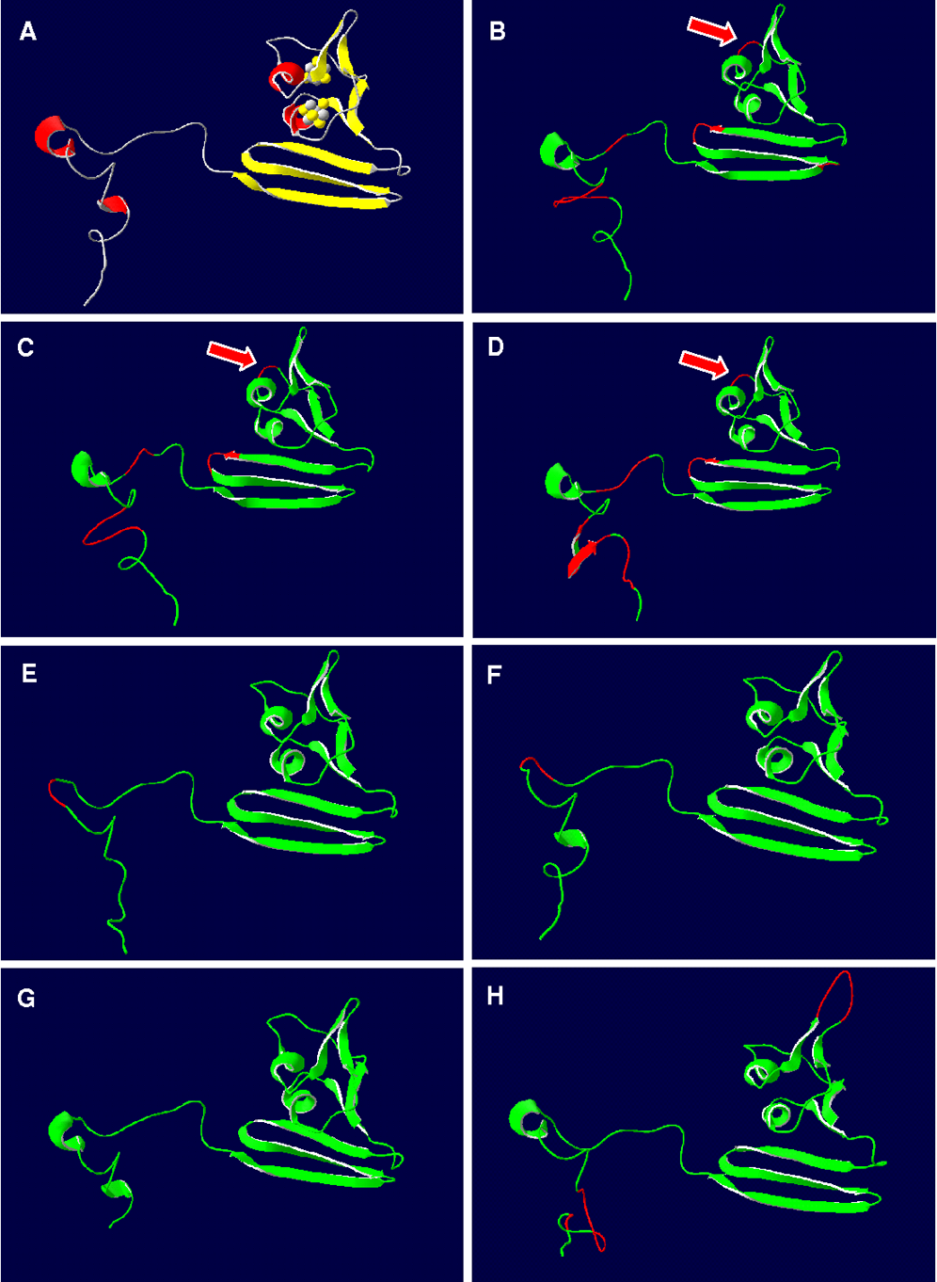
Three-dimensional structure of AprB from *A. fulgidus* (A) and selected, homology modeling-based AprB models from *Allochromatium vinosum* (B) and *Pelagibacter ubique* (C) (as representatives of SOB from Apr lineage-I), *Pyrobaculum calidifontis* (D) (as representative of crenarchaeal SRP), *Desulfotomaculum reducens* (E) (as representative of Gram-positive SRB and LGT-affected deltaproteobacterial SRB), *Desulfovibrio vulgaris* (F) (as representative of non-LGT-affected deltaproteobacterial SRB), *Chlorobaculum tepidum* (G) and *Thiobacillus denitrificans* (H) (as representatives of LGT-affected SOB from Apr lineage-II). Ribbon structure shown from front view (positions of [4Fe-4S] clusters indicated in *A. fulgidus* AprB). Ribbon structure of *A. fulgidus* AprB (A) colored by secondary structure elements; ribbon structures of AprB models (B–H) colored by model confidence factor provided by SWISS-MODEL (green, respective region of model and reference structure superpose; red, respective region of model deviates from the reference structure). The missing flexible loop between Cys-B13 and Gly-B19 (enumeration based on *A. fulgidus* sequence) in models of SOB from Apr lineage-I and *Pyrobaculum* spp. is marked by red arrows.

The three segment-subdivision of the *A. fulgidus* beta-subunit described by Fritz and coworkers [18], [23] is reflected in all comparative models of the dissimilatory APS reductase irrespective of species metabolism type and phylogenetic affiliation. While the presence and orientation of the secondary structure elements are strictly conserved in the functionally important first and second segment of the models (ferredoxin-like and alpha-subunit interface region), the third, the tail segment, is more variable among the investigated SRP and SOB (see Fig. 3 for AprB models of selected SRP/SOB; all AprB models are presented in supplementary material Table S1 and Figure S1). Significantly higher main chain atom RMS deviations of up to 3.28 Å to the *A. fulgidus* template were present in this protein region in comparison with the low values of the first segments that ranged between 0.00 and 0.84 Å (see Table 1). The tail region has been proposed to be responsible for the tightening of the subunit interaction and, thus, a stable heterodimer formation by increasing the contact surface between both subunits [18], [23]. The differing presence of secondary structure elements in the tail segments of the models is a result of the high variability in sequence and length. The structural dispersion of the tail regions might reflect the process of speciation by individual structural adaptations at the interacting surface between the beta- and alpha-subunit of each species. Indeed, the tail segment is the only sequence section of the beta-subunit that contains sufficient phylogenetic information to allow inter- and intrafamily differentiation among the SRP and SOB sequences.

**Table 1:** Root mean square deviations (RMSD) of AprB and AprA comparative models from SRP and SOB with respect to the *A. fulgidus* template structure.

Spezies	AprB				AprA	
	1.+2. segment		3. segment			
	AA	RMSD for backbone/Cα atoms in 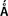 (no. of atoms involved in calculation)	AA	RMSD for backbone/Cα atoms in 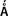 (no. of atoms involved in calculation)	AA	RMSD for backbone/Cα atoms in 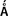 (no. of atoms involved in calculation)
**SOB Apr lineage I**						
*Allochromatium vinosum*	2-102	0.63 (392)/0.47 (98)	103-156	1.50 (184)/1.27 (46)	2-620	0.88 (2424)/0.84 (606)
*Thiobacillus denitrificans*	2-102	0.62 (392)/0.46 (98)	103-156	1.22 (184)/1.14 (46)	2-622	0.79 (2432)/0.73 (608)
*Cdt.* Ruthia magnifica	2-101	0.59 (392)/0.41 (98)	102-155	1.46 (184)/1.30 (46)	2-625	0.96 (2432)/0.94 (608)
*Pelagibacter ubique*	2-101	0.55 (392)/0.35 (98)	102-153	1.56 (184)/1.37 (46)	2-614	1.16 (2400)/1.15 (600)
EBAC2C11	2-101	0.53 (392)/0.34 (98)	102-153	1.53 (184)/1.28 (46)	2-614	1.46 (2400)/1.48 (600)
**Crenarchaeal SRP**						
*Caldivirga maquilingensis*	2-104	0.82 (392)/0.68 (98)	105-154	1.69 (180)/1.56 (45)	2-627	0.88 (2460)/0.82 (615)
*Pyrobaculum calidifontis*	2-101	0.59 (392)/0.44 (98)	102-151	3.28 (168)/3.23 (42)	2-627	1.16 (2440)/1.17 (610)
**SRB and related SOB Apr lineage-II**						
*Desulfotomaculum reducens*	2-104	0.08 (412)/0.05 (103)	105-147	2.11 (172)/2.20 (45)	2-624	0.82 (2456)/0.78 (614)
*Syntrophobacter fumaroxidans*	2-104	0.08 (412)/0.06 (103)	105-147	0.83 (172)/0.78 (43)	2-634	0.71 (2476)/0.65 (619)
fosws7f8	2-104	0.09 (412)/0.07 (103)	105-146	0.87 (180)/0.95 (45)	2-630	0.66 (2480)/0.62 (620)
fosws39f7	2-104	0.00 (412)/0.00 (103)	105-146	0.00 (168)/0.00 (42)	2-634	0.70 (2484)/0.64 (621)
*Thermodesulfobacterium commune*	2-104	0.08 (412)/0.06 (103)	105-152	0.61 (184)/0.50 (46)	2-664	0.97 (2524)/0.92 (631)
*Desulfovibrio vulgaris*	2-104	0.08 (412)/0.06 (103)	105-148	0.67 (176)/0.61 (44)	2-664	1.54 (2512)/1.53 (628)
*Desulfovibrio desulfuricans*	2-104	0.08 (412)/0.06 (103)	105-148	0.65 (176)/0.60 (44)	2-662	1.01 (2520)/0.98 (630)
*Desulfotalea psychrophila*	2-104	0.08 (412)/0.06 (103)	105-138	3.21 (140)/3.18 (35)	2-671	1.04 (2512)/1.00 (628)
*Desulfobulbus* sp. MLMS1	2-104	0.08 (412)/0.06 (103)	105-139	3.21 (140)/3.18 (35)	2-669	1.14 (2516)/1.11 (629)
*O. algarvensis* Delta 1 symbiont	2-104	0.09 (412)/0.06 (103)	105-147	1.31 (172)/1.36 (43)	2-659	1.20 (2504)/1.18 (626)
*Thermodesulfovibrio yellowstonii*	2-104	0.08 (412)/0.06 (103)	105-142	1.46 (140)/1.28 (35)	2-662	1.05 (2492)/1.00 (623)
*Chlorobaculum tepidum*	2-104	0.09 (412)/0.06 (103)	105-140	0.09 (144)/0.07 (36)	2-658	0.91 (2492)/0.88 (623)
*Thiobacillus denitrificans*	2-110	0.38 (412)/0.22 (103)	111-157	1.81 (176)/1.89 (44)	2-666	0.82 (2492)/0.79 (623)

**Figure 4:**
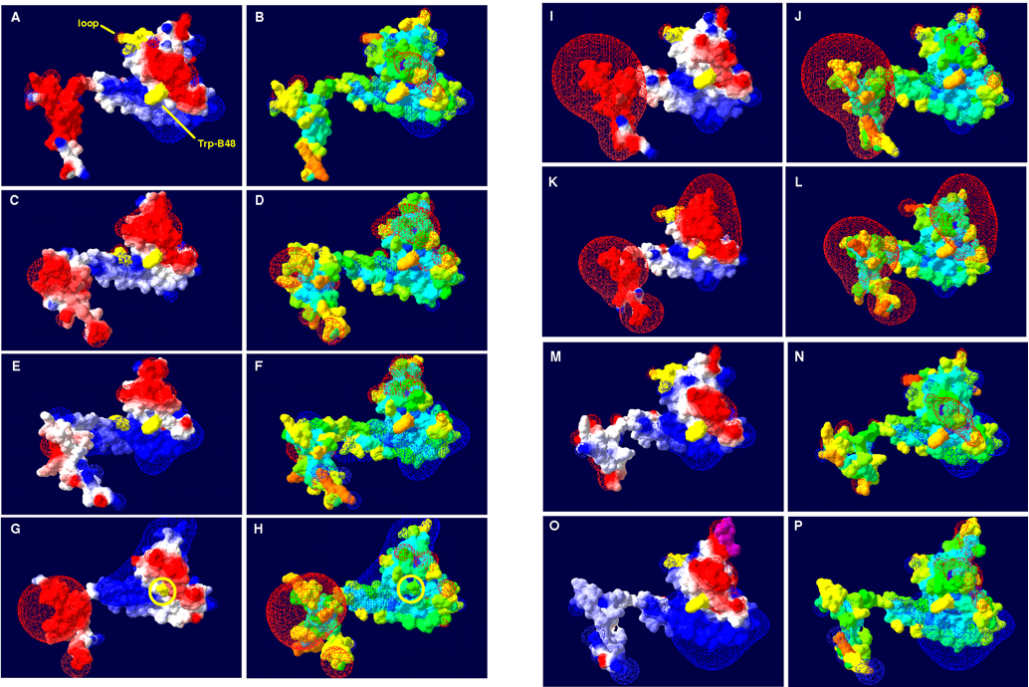
Three-dimensional structure of AprB from *A. fulgidus* (A, B) and selected, homology modeling-based AprB models from *Allochromatium vinosum* (C, D), *Pelagibacter ubique* (E, F), *Pyrobaculum calidifontis* (G, H), *Desulfotomaculum reducens* (I, J), *Desulfovibrio vulgaris* (K, L), *Chlorobaculum tepidum* (M, N) and *Thiobacillus denitrificans* (O, P). Protein molecular surface colored by calculated electrostatic potential are shown in panels A, C, E, G, I, K, M, O (electric charge at the molecular surface is colored with a red (negative), white (neutral), and blue (positive) color gradient; electric field extending into the solvent is shown); the differently present, negatively charged loops in the models of SRP and SOB are marked by yellow color (the additional loop of *Thiobacillus denitrificans* is shown in violet); the electron-transferring Trp-B43/-B48 is marked by yellow color (*Pyrobaculum calidifontis*: Trp-substituting Ala-B43 is highlighted in G and H). Protein molecular surface colored by calculated solvent accessibility are shown in panels B, D, F, H, J, L, N, P.

Besides their overall conformity in the secondary structure element positioning, the first and the second segment of the comparative models showed significant differences in distinct loop regions resulting from insertions and deletions (see Fig. 3 and 4; for details see supplementary data material Table S1 and Figure S1). Interestingly, these structural differences reflected the AprBA-based phylogeny in its separation into three major phylogenetic AprBA clusters: (1) the SRB including the LGT-affected Archaeoglobi and SOB of Apr lineage II, (2) the *Caldivirga*-*Pyrobaculum* group of putative crenarchaeal sulfate-reducers, and (3) the SOB of distinct Apr lineage I (see Fig. 2A). Furthermore, the presence/absence of certain loops among the AprB models of each cluster correlated with the absence/presence of the Qmo redox complex and AprM protein encoding genes in the investigated SRP and SOB genomes [16], [17] and might reflect their different functional linkage to the electron transport chain in the membrane. (1) In all comparative AprB models of SRB, *A. fulgidus* and the SOB of Apr lineage II, there is a flexible loop conservatively located between Cys-B13 and Gly-B19 which comprised predominantly charged amino acids of the following general sequence, K G X D/E K/R (see Fig. 3 and 4; for sequence details see supplementary data material Table S1). This loop is absent in the beta-subunit models of SOB from Apr lineage I and the *Caldivirga*-*Pyrobaculum* group (see Fig. 3B–D and supplementary data material Figure S1). Especially the conserved Lys-B14 and Glu/Asp-B17 are located at exposed, solvent-accessible positions in this loop (see Fig. 4 and supplementary data material Figures S1, panels G/H); they might be responsible for the functional docking of the putative redox partner QmoABC/QmoAB(-HdrBC) to the AprB protein surface adjacent to the [4Fe-4S] cluster II. The Qmo complex presumably operates as physiological electron carrier between the membrane-integral quinone/quinol pool and the cytoplasmic APS reductase in the SRP as well as in the sulfite-oxidizing *Chlorobiaceae* and *Beta*- and *Gammaproteobacteria* that harbor a SRB-related enzyme (SOB Apr lineage II) [17]. *Thiobacillus denitrificans* possessed a second exposed loop of six amino acids positioned between the beta-alpha-beta structure motifs of the ferredoxin-like segment; however, its functional or structural role is not apparent. (2) The crenarchaeal *Caldivirga maquilingensis* and putative sulfate-reducing *Pyrobaculum* spp. did not harbor *qmo* homologous sequences in their genomes [17]; in agreement to the previous proposal, an elongated loop between secondary structure element 2 and 3 was absent in their AprB comparative models (see Fig. 3D and supplementary material Figure S1). The putative physiological electron donor for the dissimilatory APS reductase in these species is unknown; however, naphtoquinones have been described from *Pyrobaculum* species [41], [42]. The electrostatic potential at the protein surface of the crenarchaeal AprB models differed significantly from the other SRP and SOB which might be an indication that the potential interacting redox partner and the electron transfer process of the aforementioned are different and unrelated to the latter (see Fig. 4 and supplementary data material Figures S1, panels E/F). Interestingly, both *Pyrobaculum* APS reductases missed the strictly conserved, electron transfer-relevant Trp-B48 that was substituted by an Ala-B43 residue at the corresponding AprB model position (see Fig. 4 panel G for the *Pyrobaculum calidifontis* AprB model). In all other Apr comparative models, the Trp-B48/43 was located between the S3 of cluster I and the methyl C8M of FAD (distance 12.4 Å) and in van der Waals contact with both redox centers; its indole ring was locked in its position by Thr-A233 and Arg-A232. The electron transfer function of trytophan residues located between two redox centers has been frequently documented [43], [44]; an analogous functional role, however, has not been reported for alanine residues. In consequence, the *Pyrobaculum* spp. APS reductases might have lost their structural ability for electron transfer between both subunits (essential for APS reduction) and, thus, their enzymes will not be functional anymore (see also the structural comparison of the alpha-subunits). Indeed, no cultivated *Pyrobaculum* species has been described to be capable of dissimilatory sulfate reduction [45], [46]; notably, the *aprBA* sequences of the *Pyrobaculum aerophilum* genome are frame-shifted [47] which might be a result of elevated mutation rate in irrelevant enzymes/metabolic pathways. (3) Like the beta-subunits of the crenarchaeal putative sulfate-reducers, the AprB comparative models of the SOB Apr lineage-I sulfur-oxidizers did not contain an elongated loop between secondary structure element 1 and 2 (see Fig. 3B and C and supplementary material Figure S1). Consistently, the genomes of the respective species did not include *qmo* homologues; however, their *aprBA* genes were always preceded and co-transcribed by a membrane-integral protein encoding gene, *aprM*. Interestingly, all AprB models of the SOB Apr lineage I possessed in the antiparallel beta-sheet segment an elongated loop by amino acid insertion between the secondary structure element 8 and 9 that comprised predominantly negatively charged residues (see Fig. 4 panels C and E; supplementary data material Table S1 and Figure S1, compare panels E/F). The latter were exposed to the solvent (see Fig. 4 panels D and F and supplementary data material Figure S1, compare panels G/H) and might function as an interface region for docking the AprBA enzyme to the AprM protein that anchors the dissimilatory APS reductase to the membrane and enables its physical contact to the yet unknown electron receptor. The presumed differing functional linkage of the cytoplasmic SOB Apr lineage I-type APS reductases to the membrane (not involving Qmo complex homologues) was also reflected in the deviating electrostatic potential at the protein surface when compared to the SRB-type AprB models (supplementary data material Figure S1, compare panels E/F).

### The [4Fe-4S] clusters surrounding protein matrix in the AprB models of SRP and SOB

**Figure 5:**
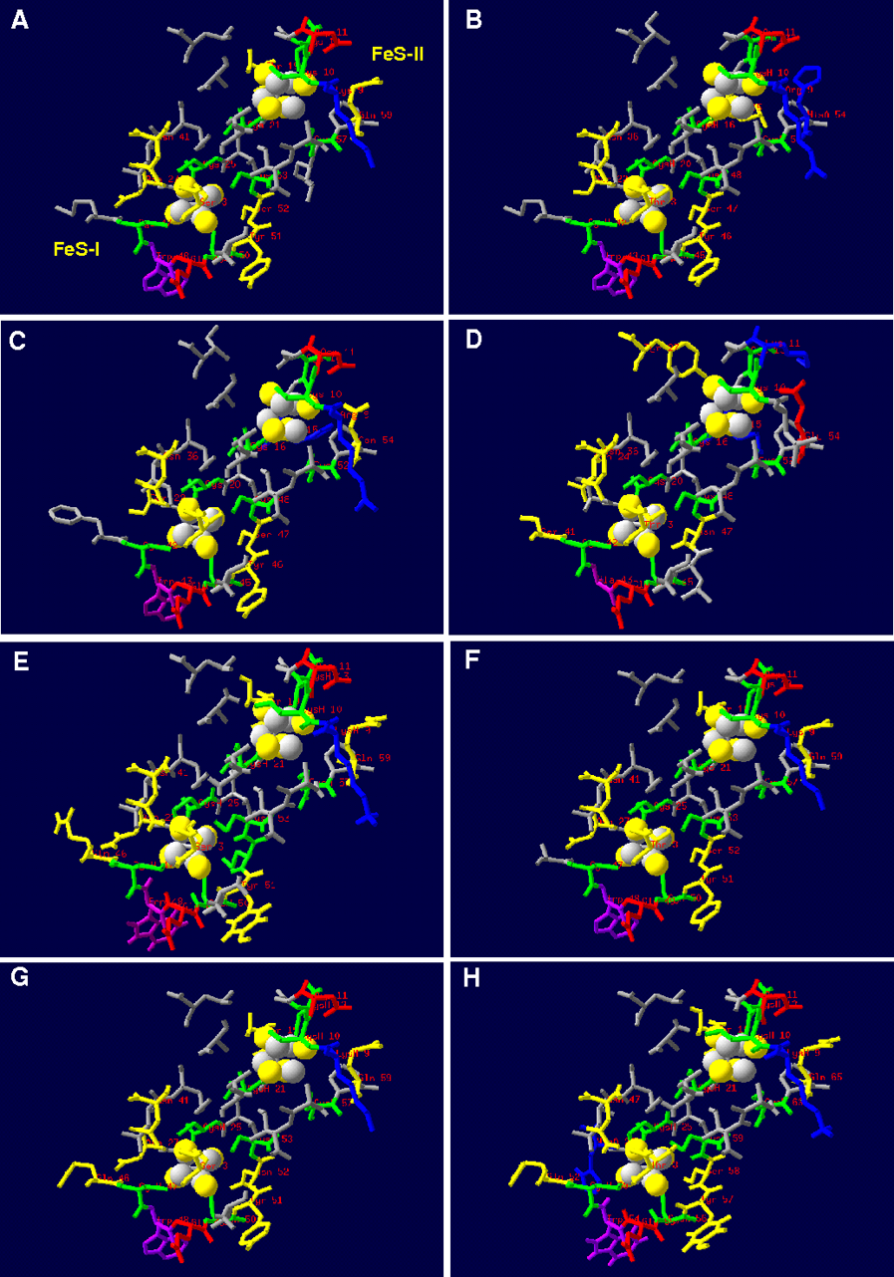
AprB protein matrix surrounding the [4Fe-4S] cluster I and II (residues in a distance of less than 5.0 Å are shown) in the three-dimensional structure from *A. fulgidus* (A) and selected, homology modeling-based models from *Allochromatium vinosum* (B), *Pelagibacter ubique* (C), *Pyrobaculum calidifontis* (D), *Desulfotomaculum reducens* (E), *Desulfovibrio vulgaris* (F), *Chlorobaculum tepidum* (G), and *Thiobacillus denitrificans* (H). Charged and polar residues are marked (positively charged AA, blue; negatively charged AA, red; polar AA, yellow); tryptophan (Trp-B48/-B43) and cysteine residues are highlighted by violet and green color.

The [4Fe-4S] clusters of APS reductases from several sulfate reducing strains have been documented to differ significantly in their reduction potential (approximately −500 mV for cluster II and −60 mV for cluster I) [18], [23], [24]. Based on the X-ray structure of *A. fulgidus*, the large reduction potential difference of the two clusters was explained by their distinctly different surrounding protein matrix (see Fig. 5 panel A). Generally, local dipole in close proximity to the acid-labile sulfur and cysteinyl sulfur atoms modulate the reduction potential of a [4Fe-4S] cluster: Its reduced state can be stabilized by NH-S hydrogen bonds and backbone amide dipoles that shift the reduction potential of an iron-sulfur center to a more positive value. In the *A. fulgidus* APS reductase, the number of polar interactions between the sulfur atoms of cluster I compared to cluster II and the backbone amides at a distance of less than 3.5 Å (17 versus 7) is substantially increased and proposed to be responsible for the high reduction potential of cluster I. In contrast, the close proximity of the negatively charged Asp-B11-carboxylate group to an acid-labile sulfur of the cluster II was suggested to stabilize its oxidized state with the result of a low reduction potential [18], [23] (see Fig. 5 panel A; see also supplementary material Figure S2 for details).

The DeepView program does not allow the calculation of potential interactions between the modeled protein and enzyme-associated cofactors; the structural comparison of the different comparative models and the *A. fulgidus* template was therefore restricted to distance analyses between the [4Fe-4S] clusters and their surrounding protein matrix. Notably, there were no significant structural differences between the comparative models of SRP and those of SOB (see Table 2, see Fig. 5 for AprB models of selected SRP/SOB; for details see supplementary data material Figure S2 and Table S2). Indeed, the protein environments of the clusters from sulfur-oxidizers of Apr lineage II, e.g. *Chlorobaculum tepidum* and *Thiobacillus denitrificans*, were more similar to those of SRP than to those of Apr lineage I sulfur-oxidizers (compare residues in a distance of less than 3.5 Å to the clusters, Table 2). In consequence, there was no increased number of polar interactions between cluster II compared with cluster I and backbone amides in the aforementioned SOB that would modulate the iron-sulfur center reduction potentials in order to favor the reverse “oxidative” electron transfer in these species. According to the models, the structural differences in the [4Fe-4S] ambient protein matrix rather correlated with the AprBA phylogeny: In contrast to the models of SRB including affiliated Archaeoglobi and SOB of Apr lineage II, the Apr lineage I SOB and crenarchaeal SRP models revealed a deviating successive appearance of amino acids near both clusters (see supplementary data material Figure S2 and Table S2). As a result of the missing elongated loop between secondary structure 1 and 2, Ile-B24 and Thr-B3 came very close (2.0 and 3.0 Å, respectively) to the [4Fe-4S] cluster I, whereas cluster II exhibited an increased number of backbone amide contacts at a distance of less than 3.0 Å in contrast to cluster I. Besides the carboxylate group of Asp-B11 (in some cases substituted by Glu-B54), the side chain of a histidine residue (His-B15/54/55) was positioned in proximity (3.5 Å) to cluster II in most AprB models of Apr lineage I SOB (Table 2 and supplementary data material Figure S2 and Table S2). However, if this positively charged surrounding is appropriate to stabilize the reduced state of cluster II and, thus, to shift its reduction potential to more positive values remains to be proven experimentally. Noteworthy, both *Pyrobaculum* spp. AprB models contained two positively charged amino acids adjacent to the acid labile sulfur and cysteinyl sulfur atoms of cluster II that would not favor oxidized state of the latter as proposed to be essential for the intrinsic electron transfer in the SRP-type proteins (see Fig. 5 panel D for *Pyrobaculum calidifontis*). Indeed, this feature might be another indication for the presence of a non-functional enzyme in the investigated *Pyrobaculum* species.

**Table 2:** AprB amino acid residues surrounding the [4Fe-4S] clusters and cysteinyl sulfur atoms in a distance of less than 3.5 Å (sorted by chemical nature of the amino acid side chain)

	Reference structure	SOB of Apr lineage I					Crenarchaeal SRP			SRB and SOB of related Apr lineage II		
[4Fe-4S] cluster	*Archaeoglobus fulgidus*	*Allochromatium vinosum*	*Thiobacillus denitrificans*	*Cdt.* Ruthia magnifica	*Pelagibacter ubique*	EBAC2C11	*Caldivirga maquilingensis*	*Pyrobaculum calidifontis*	*Pyrobaculum aerophilum*	*Chlorobaculum tepidum*	*Thermodesulfo-vibrio yellowstonii*	*Thiobacillus denitrificans*
Cluster I	Cys25, Cys47, Cys50, Cys53	Cys20, Cys42, Cys45, Cys48	Cys20, Cys42, Cys45, Cys48	Cys20, Cys42, Cys45, Cys48	Cys20, Cys42, Cys45, Cys48	Cys20, Cys42, Cys45, Cys48	Cys20, Cys45, Cys48, Cys51	Cys20, Cys42, Cys45, Cys48	Cys20, Cys42, Cys45, Cys48	Cys25, Cys47, Cys50, Cys53	Cys25, Cys47, Cys50, Cys53	Cys25, Cys53, Cys56, Cys59
	Ser3, Asn41, Asn27, Tyr51, Ser52	Thr3, Ser22, Tyr46, Ser47	Thr3, Ser22, Tyr46, Ser47	Thr3, Ser22, Asn23, Tyr46, Ser47	Thr3, Ser22, Tyr46, Ser47	Thr3, Ser22, Tyr46, Ser47	Ser3, Ser22, Tyr49, Asn50	Thr3, Asn24, Asn47	Thr3, Asn24, Asn47	Ser3, Tyr51, Asn52	Ser3, Tyr51, Asn52	Thr3, Tyr57, Ser58
	Asp28, Glu49	Asp23, Glu44	Asp23, Glu44	Glu44	Asp23, Glu44	Asp23, Glu44	Glu47	Asp23, Glu44	Glu49, Glu44	Glu28, Glu49	Asp28, Glu49	Asp28, Glu55
							His23					His27
	Leu29, Val54	Ile24, Pro21, Val49	Pro21, Ile24, Val49	Pro21, Ile24, Val49	Ile24, Val49	Pro21, Ile24, Val49	Pro21, Ile24, Val52, Ile62	Leu46, Val49	Leu46, Val49	Leu29, Val54	Leu29, Val54	Val60
Cluster II	Cys10, Cys21, Cys57, Cys13	Cys13, Cys10, Cys16, Cys52	Cys13, Cys10, Cys16, Cys52	Cys13, Cys10, Cys16, Cys52	Cys13, Cys10, Cys16, Cys52	Cys13, Cys10, Cys16, Cys52	Cys13, Cys10, Cys16, Cys55	Cys13, Cys10, Cys16, Cys52	Cys13, Cys10, Cys16, Cys52	Cys10, Cys13, Cys21, Cys57	Cys10, Cys13, Cys21, Cys57	Cys10, Cys13, Cys21, Cys63
	Gln59	Asn55	Tyr15	Gln55	Asn54	Asn55	Thr11	Tyr27	Tyr27	Gln60	Gln22, Gln60	Gln66
	Asp11, Glu22	Asp11	Asp11, Asp55	Asp11	Asp11	Asp11		Glu54	Glu54	Asp11	Asp11	Asp11
		His54		His15, Arg33	His55	His15		Lys11, His55	Lys11, His55			
	Gly12, Gly60, Ala61	Gly12, Gly14, Val17, Ala56, Ile57	Gly12, Gly14, Val17, Met54, Ala56, Ile57	Gly12, Gly14, Val17, Ala56, Ile57	Gly12, Gly14, Val17, Ala56, Ile57	Gly12, Gly14, Val17, Ala56, Ile57	Gly12, Gly14, Val17, Phe27, Gly58, Ala59	Gly12, Gly14, Val17, Ala56	Gly12, Gly14, Val17, Ala56	Gly12, Ala20, Met22, Ala61	Gly12, Ala20, Ala61	Gly12, Ala20, Met22, Ala67

	Reference structure	SRB									
[4Fe-4S] cluster	*Archaeoglobus fulgidus*	*Desulfotomaculum reducens*	*Syntrophobacter fumaroxidans*	fosws39f	foswsf8	*Thermodesulfobac-terium commune*	*Desulfovibrio desulfuricans*	*Desulfovibrio vulgaris*	*Desulfotalea psychrophila*	*Desulfobulbus* sp.	*O. algarvensis* Delta 1 symbiont
Cluster I	Cys25, Cys47, Cys50, Cys53	Cys25, Cys47, Cys50, Cys53	Cys25, Cys47, Cys50, Cys53	Cys25, Cys47, Cys50, Cys53	Cys25, Cys47, Cys50, Cys53	Cys25, Cys47, Cys50, Cys53	Cys25, Cys47, Cys50, Cys53	Cys25, Cys47, Cys50, Cys53	Cys25, Cys47, Cys50, Cys53	Cys25, Cys47, Cys50, Cys53	Cys25, Cys47, Cys50, Cys53
	Ser3, Asn41, Asn27, Tyr51, Ser52	Ser3, Asn27, Tyr51	Ser3, Asn27, Tyr51, Asn52	Ser3, Asn27, Asn41, Tyr51, Ser52	Ser3, Tyr51	Ser3, Tyr51, Ser52	Thr3, Tyr51, Ser52	Thr3, Tyr51, Ser52	Ser3, Tyr51, Ser52	Ser3, Tyr51, Ser52	Ser3, Ser52
	Asp28, Glu49	Asp28, Glu49	Asp28, Glu49	Asp28, Glu49	Asp28, Glu49	Glu49	Glu49	Glu49	Asp28, Glu49	Asp28, Glu49	Glu49
	Leu29, Val54	Pro26, Leu29, Cys52, Val54	Leu29, Val54	Leu29, Val54	Leu29, Cys52, Val54	Leu29, Val54	Leu29, Val54	Leu29, Val54	Leu29, Val54	Leu29, Val54	Leu29, Phe51, Val54
Cluster II	Cys10, Cys21, Cys57, Cys13	Cys10, Cys13, Cys21, Cys57	Cys10, Cys13, Cys21, Cys57	Cys10, Cys21, Cys57, Cys13	Cys10, Cys13, Cys21, Cys57	Cys10, Cys13, Cys21, Cys57	Cys10, Cys13, Cys21, Cys57	Cys10, Cys13, Cys21, Cys57	Cys10, Cys13, Cys21, Cys57	Cys10, Cys13, Cys21, Cys57	Cys10, Cys13, Cys21, Cys57
	Gln59	Gln60	Gln60	Gln22, Gln60	Gln22, Gln60						Gln22, Thr59, Gln60
	Asp11, Glu22	Asp11	Asp11	Asp11	Asp11	Asp11	Asp11	Asp11	Asp11	Asp11	Asp11
	Gly12, Gly60, Ala61	Gly12, Ala20, Met22, Ala61, Ile62	Gly12, Ala20, Met22, Ala61	Gly12, Met38, Val59, Ala61	Gly12, Ala20, Val59, Ala61	Gly12, Val20, Met22, Gly60, Ala61	Gly12, Ala20, Met22, Gly60, Ala61, Ile62	Gly12, Ala20, Met22, Gly60, Ala61, Ile62	Gly12, Ala20, Met22, Gly60, Ala61, Ile62	Gly12, Ala20, Met22, Gly60, Ala61	Gly12, Ala20, Ala61, Ile62

### Structural comparison of the alpha-subunit (AprA) comparative models of the dissimilatory APS reductase among SRP and SOB

**Figure 6:**
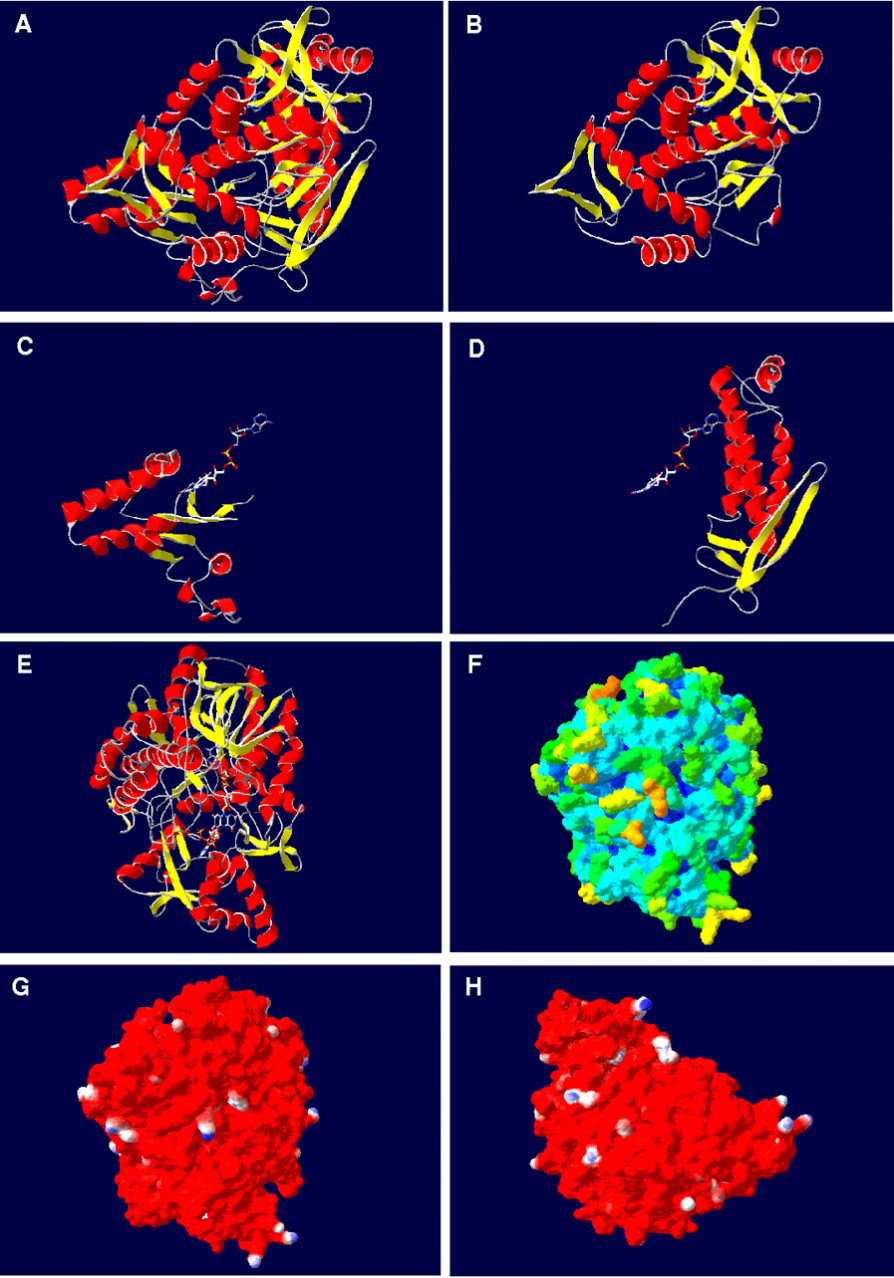
Structure of AprA from *A. fulgidus.* (A–E) Ribbon structure colored by secondary structure elements (position of FAD cofactor indicated), (A) entire model from front view, (B) only FAD-binding domain, (C) only capping domain, (D) only helical domain, and (E) entire protein from top view (position of APS molecule indicated additionally); (F) protein molecular surface colored by calculated solvent accessibility shown from top view; (G, H) protein molecular surface colored by calculated electrostatic potential shown from top and back view (electric charge at the molecular surface is colored with a red (negative), white (neutral, and blue (positive) color gradient).

**Figure 7:**
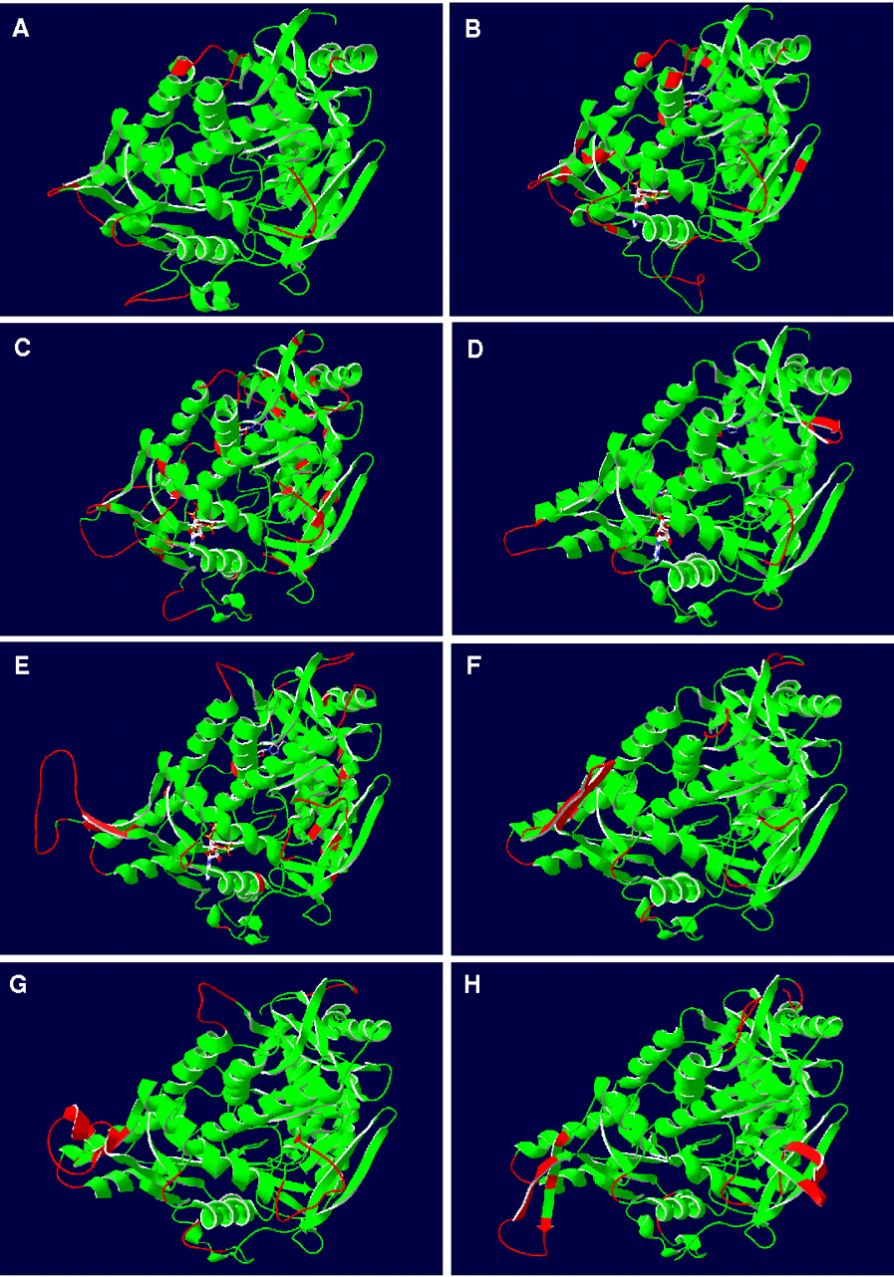
Selected, homology modeling-based AprA models from *Allochromatium vinosum* (A) and *Pelagibacter ubique* (B) (as representatives of SOB from Apr lineage-I), *Pyrobaculum calidifontis* (C) (as representative of crenarchaeal SRP), *Desulfotomaculum reducens* (D) (as representative of Gram-positive SRB and LGT-affected deltaproteobacterial SRB), *Thermodesulfobacterium commune* (E) (as representative of thermophilic SRB), *Desulfovibrio vulgaris* (F) (as representative of non-LGT-affected deltaproteobacterial SRB), *Chlorobaculum tepidum* (G) and *Thiobacillus denitrificans* (H) (as representatives of LGT-affected SOB from Apr lineage-II). Ribbon structure shown from front view (position of FAD cofactor and substrate APS are indicated). Ribbon structure of AprA models colored by model confidence factor provided by SWISS-MODEL (green, respective region of model and reference structure superpose; red, respective region of model deviates from the reference structure).

The *A. fulgidus* alpha-subunit has been described to be subdivided into three domains, the FAD-binding (A2-261, A394-487), capping (A262-393), and the helical domain (A488-643) (see Fig. 1 and 6). The FAD-binding domain constitutes the center, while the capping and helical domains form the periphery of the alpha-subunit (the helical domain is firmly attached to the first whereas the capping domain is partly exposed from the core region). The FAD-binding domain is composed of a central six-stranded parallel beta-sheet (sec. str. elm. 1, 3, 11, 12, 13, 14) that is flanked by four alpha-helices on one side (sec. str. elm. 2, 6, 10, 36) and by a four-stranded mixed beta-sheet on the other (sec. str. elm. 15, 17, 19, 32) (see Fig. 6 panel B). The capping domain is inserted into the polypeptide chain of the aforementioned domain and consists of a four-stranded antiparallel beta-sheet (sec. str. elm. 20. 22, 27, 31) surrounded by eight (mostly short) alpha-helices (see Fig. 6 panel C), whereas the helical domain is primarily composed of three long alpha-helices (sec. str. elm. 39, 41, 42) (see Fig. 6 panel D) [18], [23]. This general fold scheme was present in all comparative AprA models of APS reductases from SOB and SRP (irrespective of species metabolism type and protein phylogenetic affiliation) (see Fig. 7, for details see supplementary data material Table S3 and Figure S3). The conserved nature of the AprA structure was reflected in the low main chain atom RMS deviations from the template structure that ranged between 0.66 Å (uncultured SRB fosws7f8) and 1.20 Å (*O. algarvensis* Delta 1 symbiont) in the SRB and SOB Apr lineage II models (except the model of *Desulfovibrio vulgaris* with 1.54 Å RMSD), between 0.79 Å (*Thiobacillus denitrificans*) and 1.46 Å (environmental sequence EBAC2C11) in the SOB Apr lineage I, and in the crenarchaeal SRP group between 0.88 Å (*Caldivirga maquilingensis*) and 1.16 Å (*Pyrobaculum calidifontis*) (see Table 1). The helical domain which has been suggested to mainly build up the interface region between two αβ-heterodimers [18], [23] constituted the most highly conserved protein region in the AprA models with regard to the presence and orientation of the secondary structure elements (RMSD from 0.06 to 0.99 Å; see supplementary data material Table S3 and Figure S3). In contrast, the FAD-binding and the capping domains of the comparative AprA models showed more structural differences; nevertheless, these alterations were predominantly restricted to certain loop amino acid stretches (e.g. between sec. str. elm. 10 and 11 or 36 and 37) and short secondary structure elements (e.g. alpha-helices, sec. str. elm. 23, 24 or 33) located at the protein surface (see supplementary data material Table S3 and Figure S3). As mentioned in a previous section, the higher structural variability among the latter might reflect the individual adaptations at the contact areas between both APS reductase subunits of each species because these domains comprise the AprA interface areas to the second and third segment of AprB. Overall, the alpha-subunit core region was conserved and structurally uniform among the enzymes of sulfur-oxidizers and sulfate-reducers. Notably, the comparative models of *Thermodesulfovibrio yellowstonii*, *Thermodesulfobacterium commune*, the non-LGT-affected deltaproteobacterial SRB and the SOB of Apr lineage II contained 17 to 21 amino acids long insertions between the secondary structure elements 7 and 8; the inserted amino acid stretches were predicted by SWISS-MODEL to primarily form two extended, antiparallel beta-sheets that are exposed from the core region (see Fig. 7 panel E to H, see also supplementary data material Table S3 and Figure S3). The accuracy of the proposed structures in this AprA model area is highly speculative because of the current computational limitations in calculating the correct fold of loop regions encompassing more than eight residues. Its solvent-exposed location at the AprB-interacting site of the alpha-subunit might be an indication for an involvement in stabilizing the contact between the native electron donor/acceptor protein and the electron-transferring smaller subunit of APS reductase.

### FAD cofactor surrounding protein matrix in the AprA models of SRP and SOB

**Figure 8:**
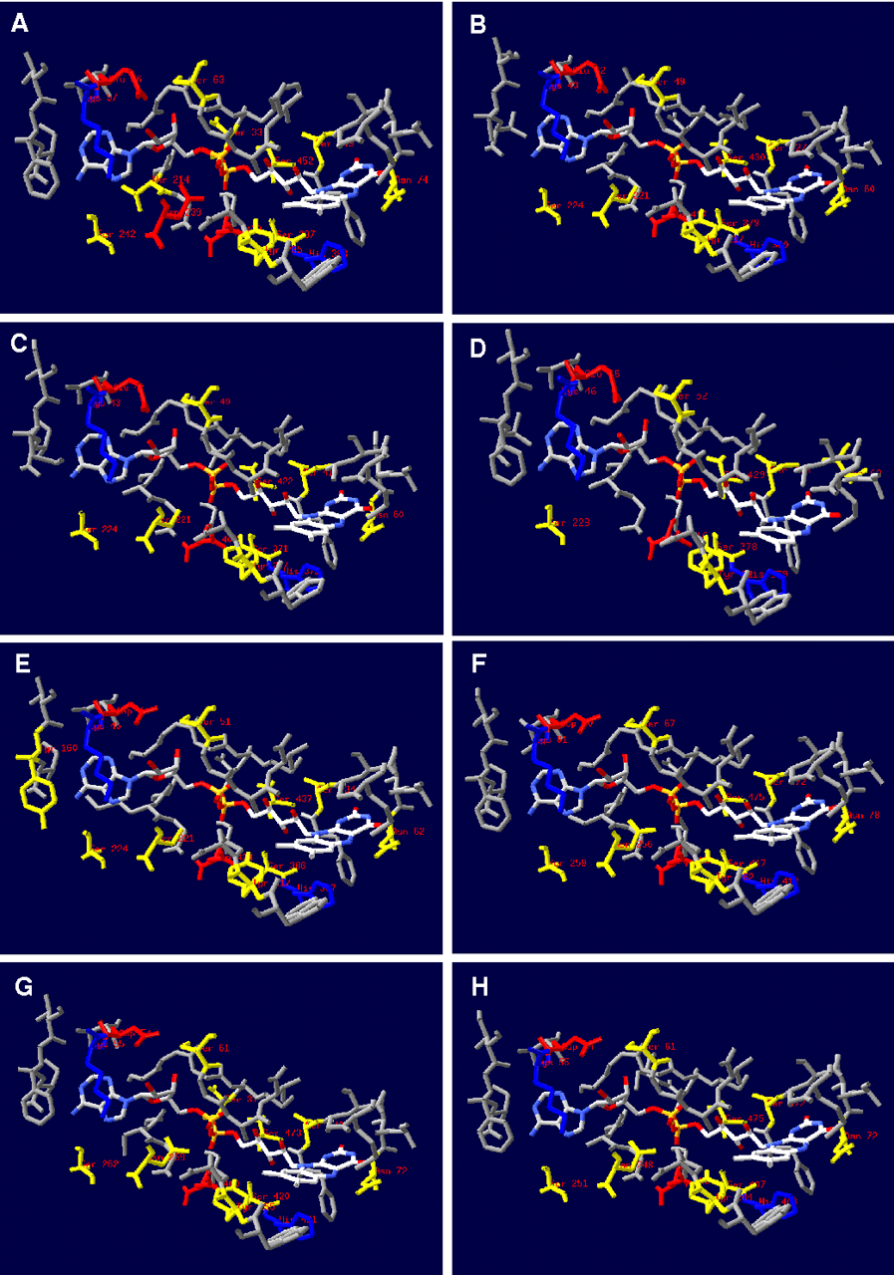
AprA protein matrix surrounding the FAD cofactor (residues in a distance of less than 4.1 Å are shown) in the three-dimensional structure from *A. fulgidus* (A) and selected, homology modeling-based models from *Allochromatium vinosum* (B), *Pelagibacter ubique* (C), *Pyrobaculum calidifontis* (D), *Desulfotomaculum reducens* (E), *Desulfovibrio vulgaris* (F), *Chlorobaculum tepidum* (G), and *Thiobacillus denitrificans* (H). Charged and polar residues are marked (positively charged AA, blue; negatively charged AA, red; polar AA, yellow; uncharged/-polar AA, grey).

**Figure 9:**
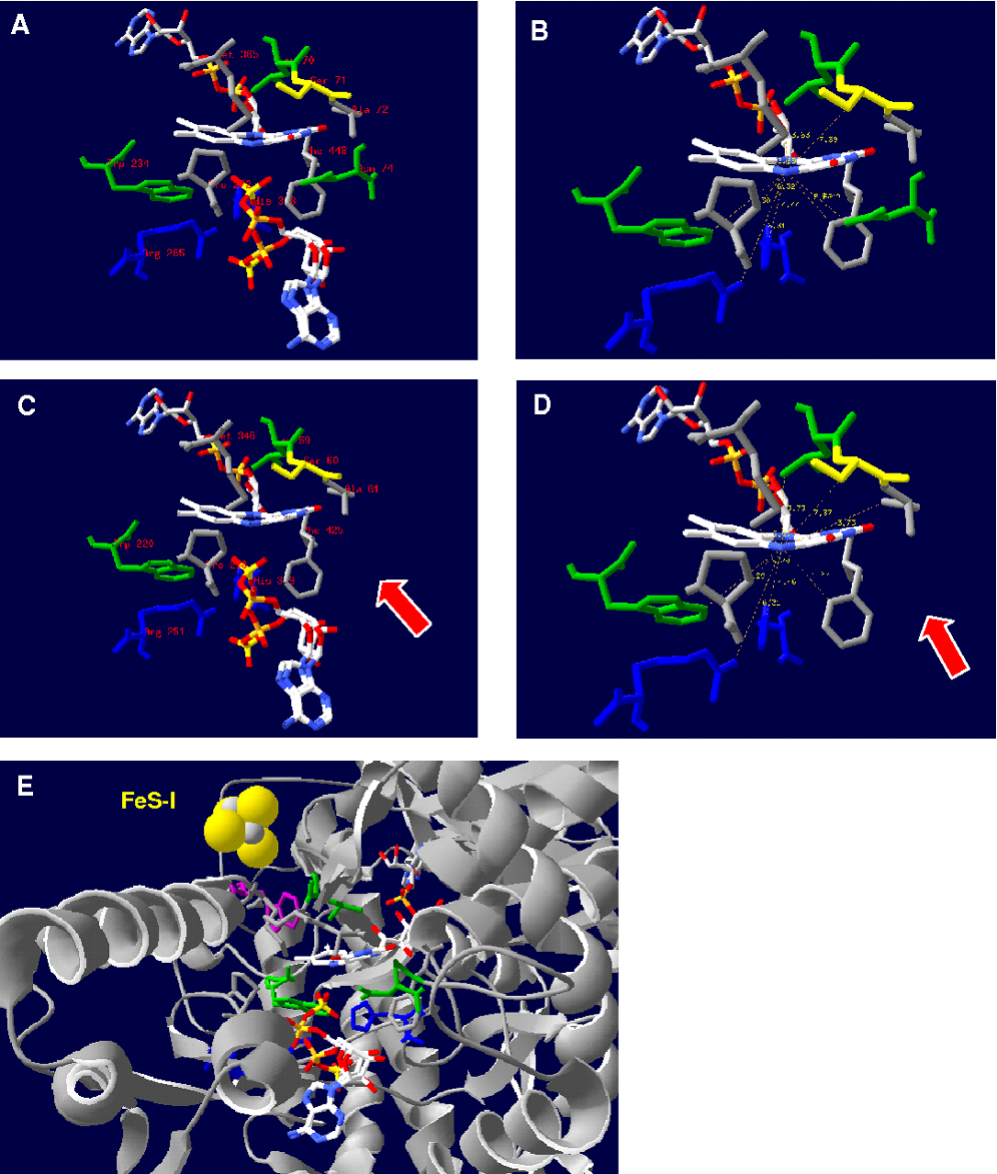
AprA active center from the protein of *A. fulgidus* (A, B) and the homology modeling-based model of *Pyrobaculum calidifontis* (C, D) (residues in a distance of less than 6.5 Å to the N5 atom of FAD cofactor are shown). FAD cofactor and substrate APS (in two conformations) are shown as ball-and-stick representations. Residues involved in the isoalloxazine binding (e.g. *A. fulgidus*: Leu-A70, Asn-A74, Trp-A234) are highlighted by green color (missing Asn-A63 in the *Pyrobaculum calidifontis* AprA model is marked by an arrow), the invariant, positively charged residues His-A398 and Arg-A265 of the active site are blue colored (other AA are colored in grey). Distances are given in Å (B, D). (E) The position of the electron-transferring [4Fe-4S] cluster I and Trp-B48 (highlighted by violet color) of AprB to the FAD cofactor in the AprA protein of *A. fulgidus* is shown (ribbon structure is colored in grey).

**Figure 10:**
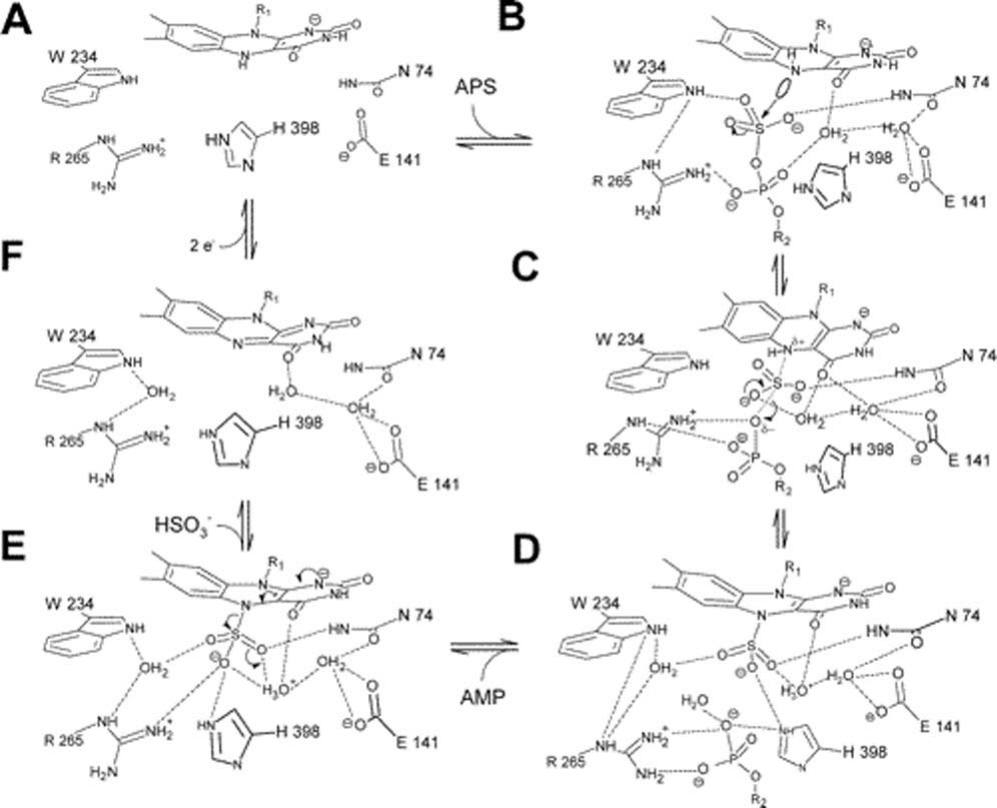
Reaction cycle of the dissimilatory APS reductase from *A. fulgidus* (Schiffer et al., 2006).

The reductive reaction mechanism of the dissimilatory APS reductase proceeds via a nucleophilic attack of the N5 atom (isoalloxazine moiety) of the reduced FAD on the sulfur of APS [18], [23], [24]. According to the AprA comparative models, 35 to 36 mostly conserved amino acids are located at a distance of less than 4.1 Å to the FAD cofactor (comprising residues of sec. str. elm. 1 to 2, 3 to 4, 11, 14 to 15, 16 to 18, 31 to 32, and 35 to 36 including the interjacent loop regions, see Fig. 8; for details see also supplementary data material Figure S3, panels G/H, and supplementary data material Table S4); thus, the cofactor-surrounding protein matrices are highly conserved among the APS reductases of SOB and SRP. As revealed by the *A. fulgidus* 3D protein structure, a pronounced feature of the FADH^−^ state in the enzyme is the substantial bend of the isoalloxazine ring along the N5-N10 axis by an angle of 25° (Fig. 9 and 10). In general, the protein matrix could have a considerable influence on the bending angle of the latter and thereby affect the redox potential of FAD; a flat conformation of FAD (0–10°) favors the oxidized state and a bent “butterfly” conformation (15–30°) favors the reduced state. In the *A. fulgidus* APS reductase, the side chains of Asn-A74 and Trp-A234 that are located at the *re*-face of FAD enforce a shift of the dimethylbenzene and pyrimidine rings toward the *si*-face of the FAD, whereas the pyrazine ring is held in position by Leu-A70 which protrudes toward the *si*-face of FAD (Fig. 9 panels A and B). The induced stabilization of the reduced form of FAD agrees with the experimentally determined higher reduction potentials of ∼−45 mV in APS reductases of several SRP compared to ∼−220 mV of free FAD [18], [23], [24]. Notably, the aforementioned residues are strictly conserved and strongly fixed at their positions in all AprA models of the sulfate reducers (except *Pyrobaculum calidifontis*, see Fig. 9 panels C and D) and even the sulfur-oxidizers (see supplementary data material Figure S4 for details) which might indicate that this cofactor is also present in a bent conformation in the SOB-type APS reductases although the coplanar arrangement of the three aromatic rings would be the energetical favorable conformation in the oxidized state. Indeed, the isoalloxazine ring system of *A. fulgidus* APS reductase revealed an identical bending angle in the oxidized as in the reduced FAD state [18], [23], [24] i.e. the butterfly conformation is maintained in the *A. fulgidus* protein independent of the redox potential of the FAD cofactor and direction of catalytic reaction (Fig. 10). This is also demonstrated by the AprA models of this study that encompassed reductive and oxidative type APS reductases from distinct SRP and SOB. In contrast, other enzymes e.g. thioredoxin reductase [48], alternate the FAD conformation with respect to their oxidized and reduced states. The AprA model of *Pyrobaculum calidifontis* lacked the structurally essential Asn-A63 at the re-face of FAD (see Fig. 9 panels C and D). However, the strained conformation of the isoalloxazine moiety was suggested to be important for efficient electron flow between the redox centers by facilitating the reduction of the oxidized FAD via the [4Fe-4S] clusters. In agreement with the AprB model-derived results, this missing structural feature might be another indication for the non-functionality of the APS reductase present in the investigated *Pyrobaculum* species.

### Active site channel and active center in the AprA models of SRP and SOB

According to the *A. fulgidus* 3D structure, the active site of APS reductase is deeply buried into the protein interior and is only accessible from the outside through a 17 Å long channel of 10 Å diameter which is formed at the interface between the FAD-binding and capping domains [18], [23], [24] (see Fig. 1B and 9E). The entrance of the substrate-binding channel is surrounded by five positively charged residues (Arg-A85, Lys-A281, Lys-A283, Arg-A294, and Arg-A317) attracting negatively charged molecules such as APS, sulfite and AMP. With the exception of Lys-A281 and the AMP-moiety-interacting Arg-A317, the previous residues were not well-conserved among the AprA comparative models of SRP and SOB but were substituted by other positively charged residues of varying positions at the opening of the channel (data not shown). The channel itself has been proposed to be pre-built prior to substrate binding by a hydrophobic cluster of residues (Tyr-A95, Trp-A144, Trp-A234, Phe-A261, Val-A273, Gly-A274, Phe-A277, Leu-A278, Phe-A448) [18], [23], [24] that would allow fast catalysis which is essential especially for SRP that rely on efficient transformation of sulfate to sulfite for energy conservation. Indeed, the aforementioned residues are strictly conserved among the AprA models (note: Tyr-A95 and Phe-A261 are substituted by the hydrophobic amino acids methionine in most deltaproteobacterial SRB and isoleucine in SOB of Apr lineage I proteins; see supplementary data material Table S5). Structural analyses of different states of the *A. fulgidus* enzyme indicated, that substrate binding induces a shift of the isoalloxazine ring towards the channel bottom thereby producing a compressed enzyme-substrate complex. While the conformations of the adenine and ribose ring of APS are well-defined in the active site channel, phosphate and, particularly, the sulfate group appeared to be conformationally rather flexible. Before reduction, the molecule APS is present in a strained conformation with its sulfate moiety directly positioned in front of the pyrazine ring of FADH^−^ (distance of ∼3.4 Å between the sulfur and the N5 atoms). In this position, the sulfate O1 atom has been predicted to interact with the ND2 atom of Asn-A74, its O2 atom with NE2 atom of His-A398, and its O3 atom via a water molecule with Arg-A265 and Trp-A234 and via two water molecules with Glu-A141, Asp-A361, and Asn-A74 (Fig. 10) [18], [23], [24]. Indeed, Asn-A74, Trp-A234, Arg-A265, and His-A398 corresponding residues were predicted to be located in the active sites of all comparative models (except *Pyrobaculum calidifontis*, see previous section) at nearly identical positions and distances to the sulfate group of APS/sulfite and the N5 atom of FAD (see supplementary data material Figure S4 and Table S6). In agreement with the *A. fulgidus* structure, the phosphate and ribose moiety of APS/AMP might be connected with Arg-A265, Val-A273, and Gly-A274 as well as Tyr-A95 and His-A446 corresponding residues in the substrate-binding channel of SRP- and SOB-type models. The adenine ring of APS/AMP is most likely fixed in its position in the models as proposed for the APS reductase of *A. fulgidus* (clamped between Arg-A317 and Leu-A278) [18], [23], [24] (see supplementary material Table S5). Upon APS/AMP binding of the *A. fulgidus* APS reductase, the Arg-A317 residues has been predicted to largely change its conformation by swinging into the channel to form a coplanar arrangement of its guanidine group to the adenine ring [18], [23], [24]. In conclusion, the active site channel including the active center appeared to be structural highly conserved among the APS reductases of SRP and even SOB with only minor alterations by single amino acid substitutions.

### Reaction mechanism of APS reduction and sulfite oxidation

The enzyme-bound APS is held in a strained conformation by the Arg-A317/Leu-A278 clamp fixation of the adenine ring and the curved APS conformation. This energy-rich state has been postulated to become relaxed during the sulfonation reaction via a nucleophilic attack of the N5 atom of reduced FAD on the sulfur atom of APS; in consequence, a covalent FAD-APS intermediate is formed. The energy liberated upon cleavage of the S-O-P mixed anhydride bond of APS is high (80 kJ mol^−1^); catalysis will be supported by an increase in the nucleophilicity of N5 due to the deprotonated N1 atom and of the electrophilicity of the sulfur due to hydrogen bonds between the four sulfate oxygens of APS and Asn-A74, Trp-A234, Arg-A265, and His-A398. Rearrangements of electrons result in the release of AMP accompanied by formation of the N5-sulfite adduct. Subsequent dissociation of the latter to yield oxidized FAD and sulfite might be enzymatically triggered by protonation of the sulfite moiety (via His-A398) (see Fig. 10). Finally, the product sulfite is released and oxidized FAD reduced via the two [4Fe-4S] clusters. Concerning the backward reaction, the oxidative formation of APS from sulfite and AMP, it is assumed that the nucleophile sulfite adds to the N5 atom of oxidized FAD as most electrophilic position of the cofactor followed by a shift of the negative charge on the sulfite sulfur toward the flavin ring. This enables a nucleophilic attack of the charged phosphate oxygen of AMP on the sulfur atom of the FAD-sulfite adduct. After rearrangement of electrons, APS is eliminated and FAD becomes reduced by two electrons, which are subsequently transferred to the iron-sulfur clusters [18], [23], [24].

With respect to the comparative models-based assumption of nearly identical protein matrices surrounding the FAD cofactor, the active site channel and center in the SRP- and SOB-type alpha-subunits, the overall catalytic process of APS reduction/sulfite oxidation in SRP and SOB appeared to be identical irrespective of metabolism type the APS reductase is involved in or species it has been originated from. Indeed, as experimentally proven by enzyme assays [12], [18], [21], [23], [24] and demonstrated by the common occurrence of sulfur isotope fractionation during microbial-mediated sulfate reduction [49]–[51], the forward but also the backward reaction is generally catalyzed by enzymes of the SRP-type (note the sulfite-oxidation unfavorable redox potential of cofactors as determined for the *A. fulgidus* protein). However, the data of UV-vis difference spectra of APS reductases from *A. fulgidus*, *Desulfovibrio desulfuricans*, and *Desulfovibrio vulgaris* (upon addition of sulfite and AMP) indicated the presence of an anionic flavosemiquinone after FAD-sulfite adduct decay and reduction of the flavin. In contrast, the enzyme isolated from the sulfur-oxidizer *Thiobacillus denitrificans* (DSM 807) did not form a stable flavosemiquinone under the same conditions, and the FAD and [4Fe-4S] clusters became immediately reduced. It has been postulated that the presence/absence of this anionic flavin radical might reflect the preferential direction of catalysis in sulfate-reducing and sulfur-oxidizing organisms [18], [23], [24]. On the basis of the comparative models, there appeared to be no significant structural differences between APS reductases of sulfate-reducers and sulfur-oxidizers that would be indicative for a favored oxidation reaction in the SOB-type enzymes (e.g. reverse modulation of redox potential of FAD and iron-sulfur centers) in comparison to the SRP-type proteins.

## Supporting Information

Figure S1 (8.23 MB DOC)

Figure S2 (2.17 MB DOC)

Figure S3 (8.75 MB DOC)

Figure S4 (1.17 MB DOC)

Table S1 (0.76 MB DOC)

Table S2 (0.21 MB DOC)

Table S3 (2.10 MB DOC)

Table S4 (0.18 MB DOC)

Table S5 (0.18 MB DOC)

Table S6 (0.19 MB DOC)
