# Supplementary material for: Homology Modeling of Dissimilatory APS Reductases (AprBA) of Sulfur-Oxidizing and Sulfate-Reducing Prokaryotes
Source: PLoS One. 2008 Jan 30;3(1):e1514. doi: 10.1371/journal.pone.0001514 (PMC2211403; doi:10.1371/journal.pone.0001514)
Supplement: Figure S1 — (8.23 MB DOC) [file pone.0001514.s001.doc]

#### Supplementary data material Figure S1. AprB comparative models of SRP and SOB

###### Reference structure

###### *Archaeoglobus fulgidus*

###### **Structure of AprB from *A. fulgidus*.** (A, B) Ribbon structure colored by secondary structure elements shown from front and back view (positions of [4Fe-4S] clusters indicated); (C-F) protein molecular surface colored by calculated electrostatic potential shown from front and back view (electric charge at the molecular surface is colored with a red (negative), white (neutral, and blue (positive) color gradient; electric field extending into the solvent is shown), in (E) and (F) Trp-B48 and negatively charged loop (Cys-B13 to Arg-B18 are marked by yellow color; (G, H) protein molecular surface colored by calculated solvent accessibility shown from front and back view

######
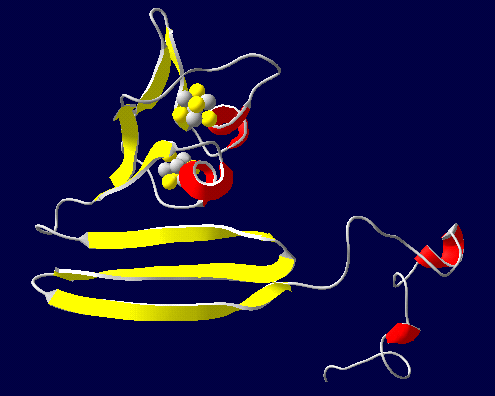


######
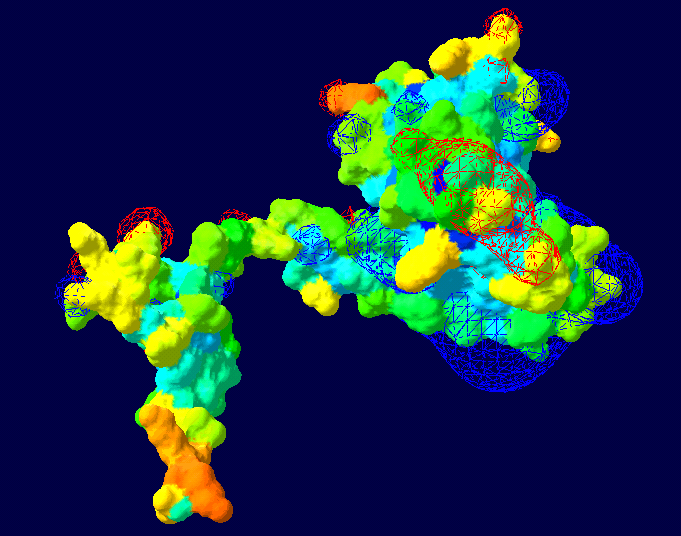

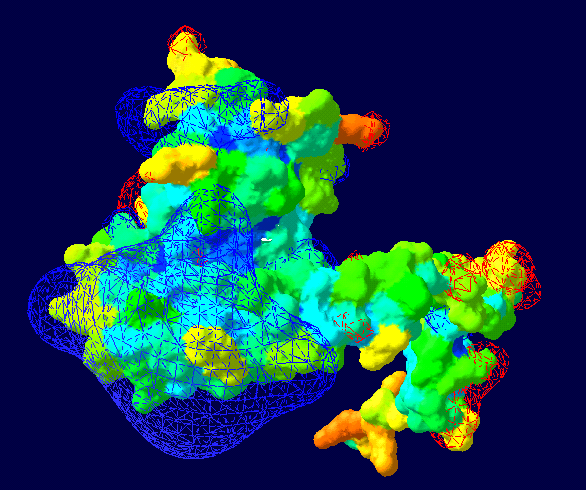

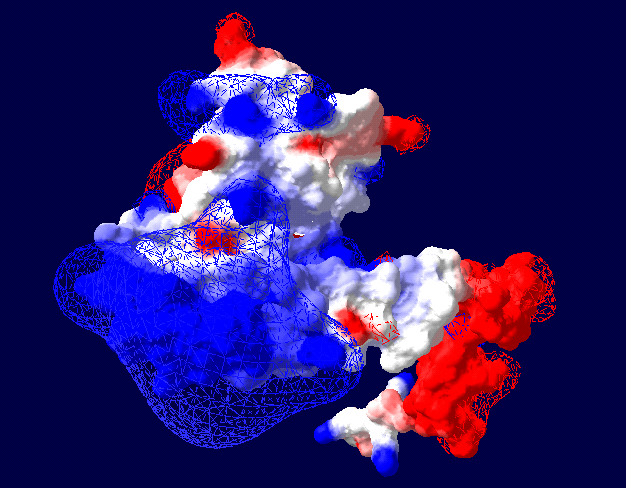

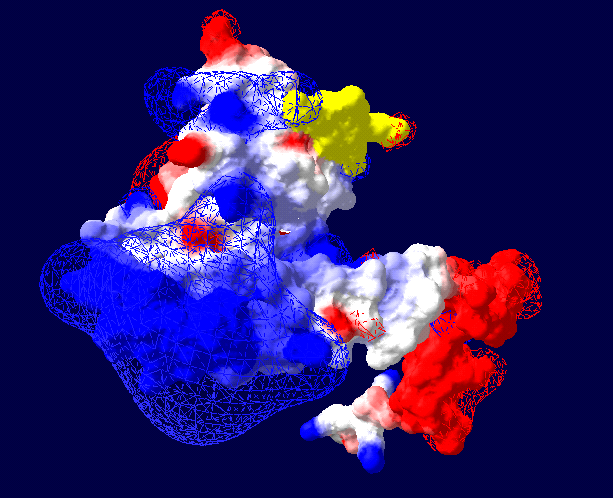


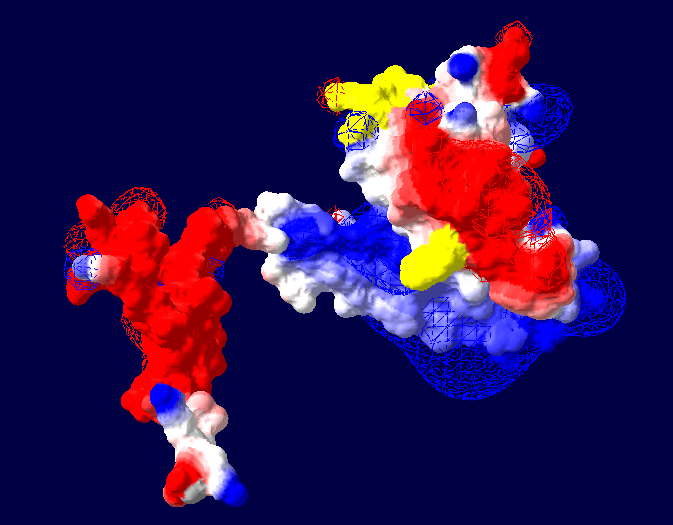

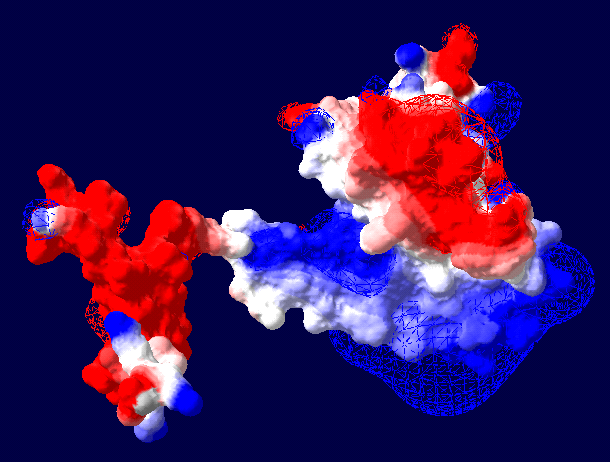

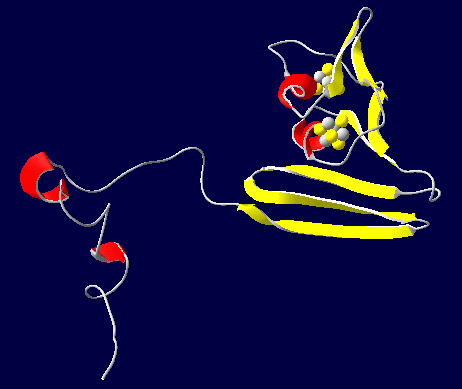


**A**

**C**

**E**

**G**

**B**

**D**

**F**

**H**

###### SOB Apr lineage I

Allochromatium vinosum

AprB model site towards AprA model AprB model site away from AprA

*
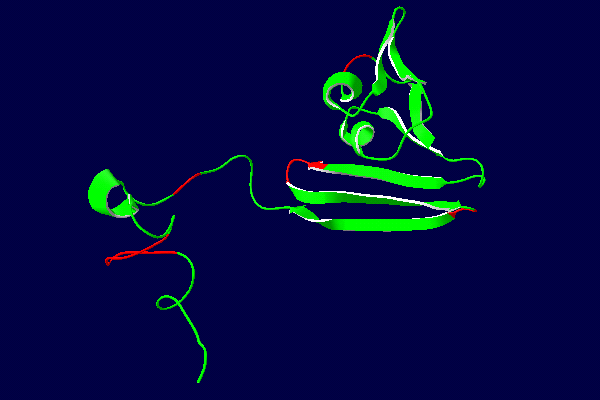

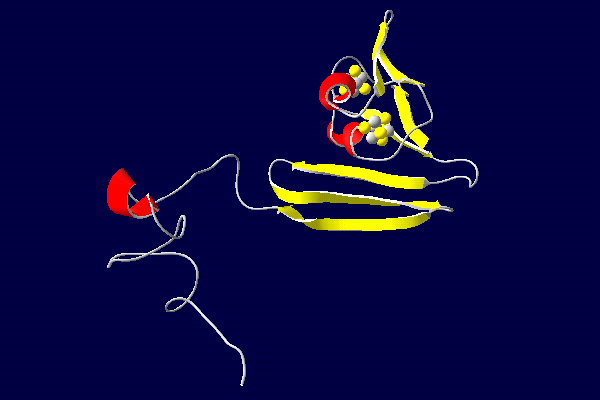
*

# A

# B

3D ribbon structure colored by model confidence 3D ribbon structure colored by secondary structure elements (positions of [4Fe-4S] cluster indicated)


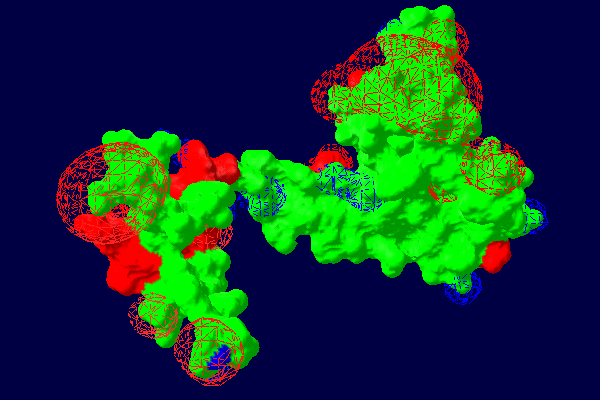

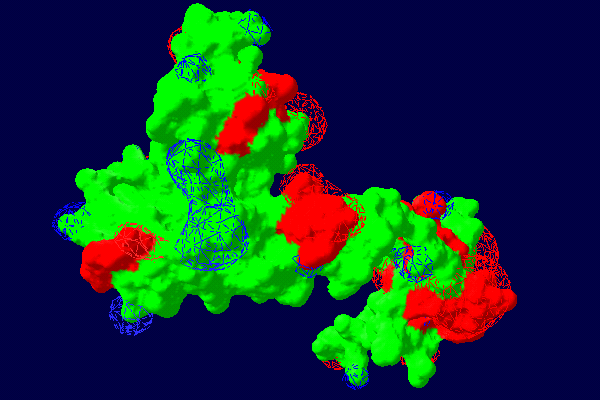


# C

# D


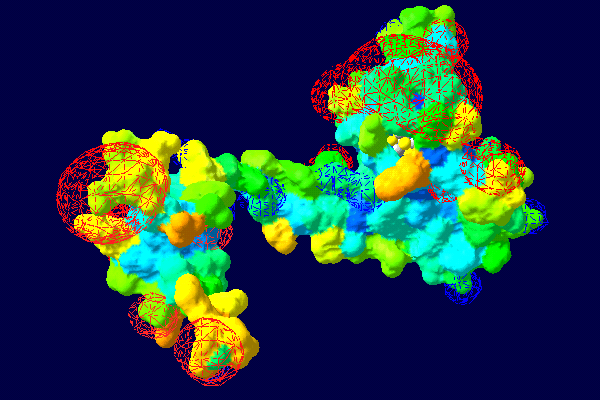

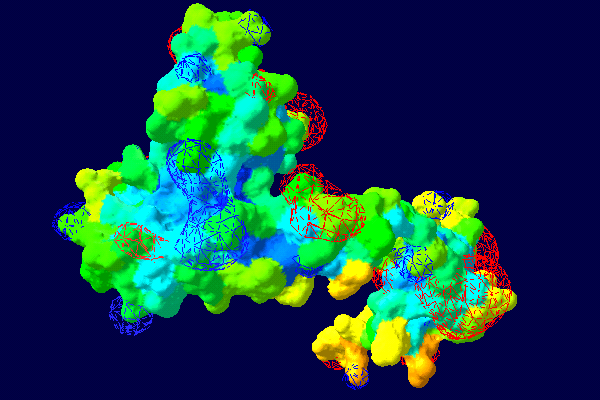
Protein molecular surface colored by model confidence


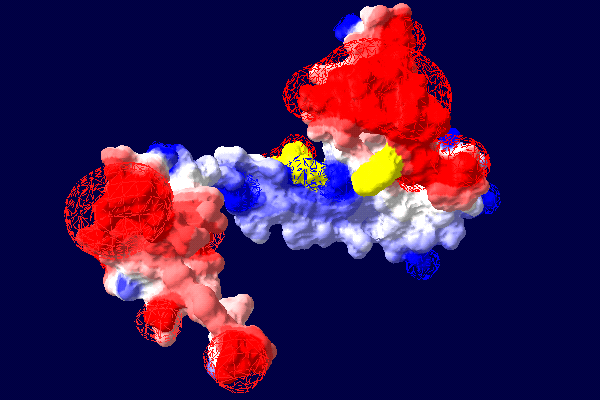

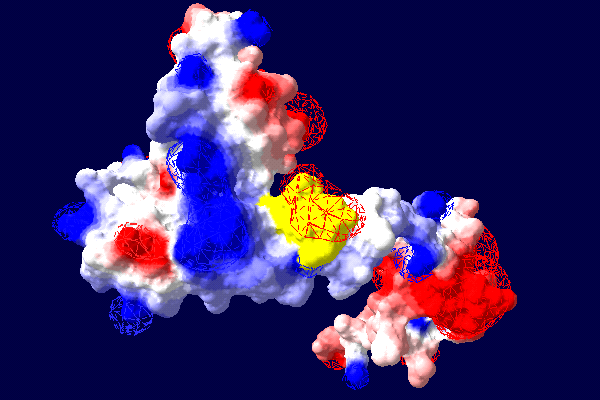


# E

# F

Protein molecular surface colored by calculated electrostatic potential (electric charge at the molecular surface is colored with a red (negative), white (neutral, and blue (positive) color gradient; electric field extending into the solvent is shown); Trp-B43 and negatively charged loop are marked by yellow color)


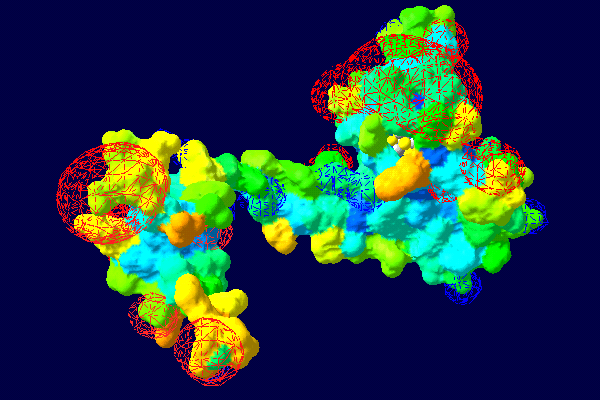

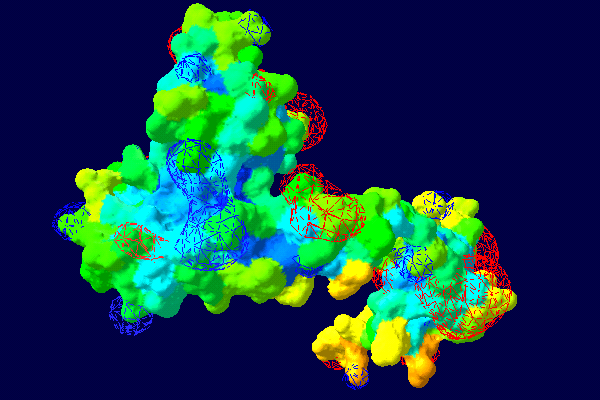


# G

# H

Protein molecular surface colored by calculated solvent accessibility

Thiobacillus denitrificans

AprB model site towards AprA model AprB model site away from AprA


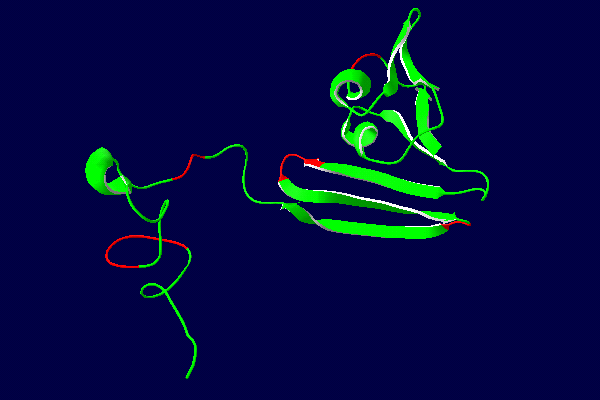

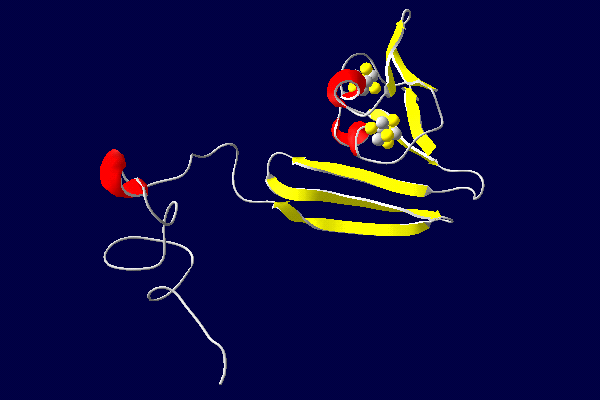


# A

# B

# C

# D

# E

# F

# G

# H

3D ribbon structure colored by model confidence 3D ribbon structure colored by secondary structure elements (positions of [4Fe-4S] cluster indicated)


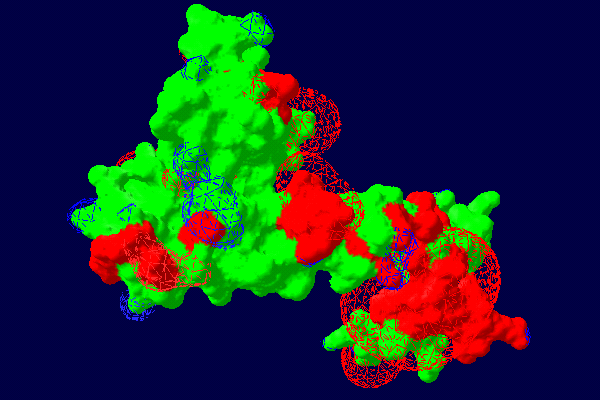

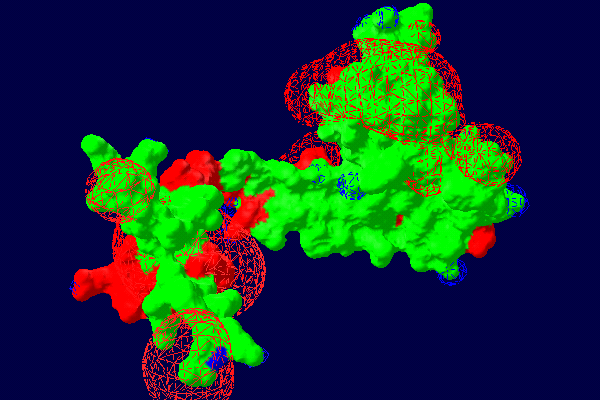


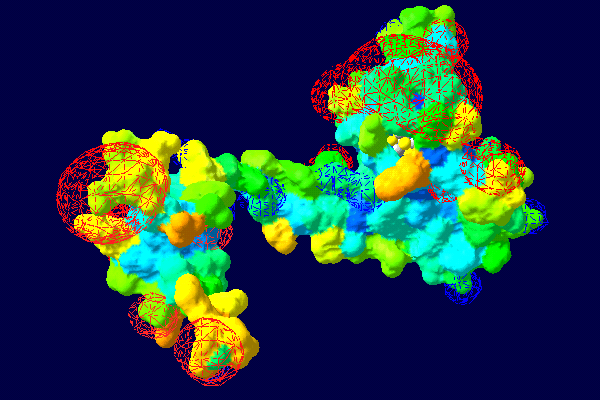

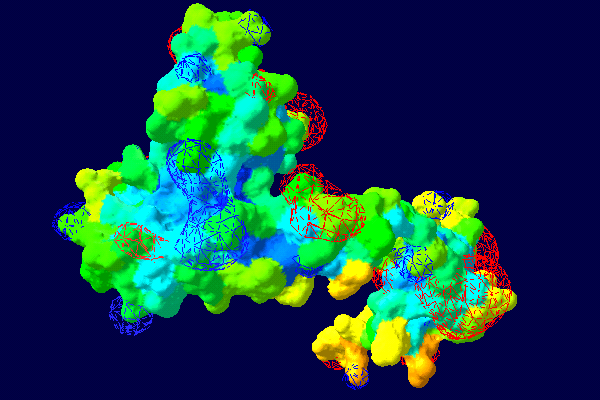
Protein molecular surface colored by model confidence


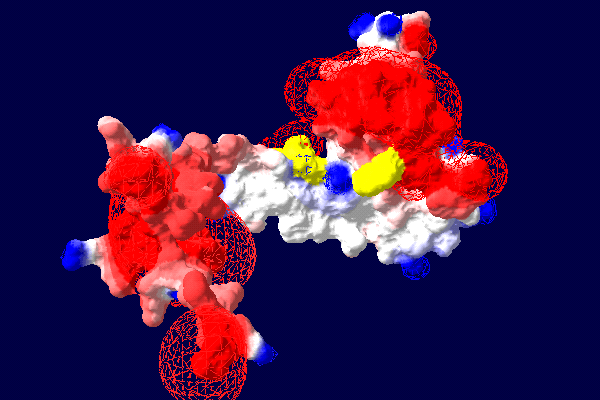

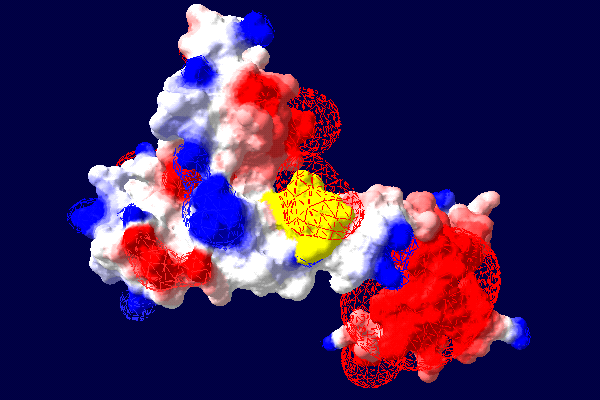


Protein molecular surface colored by calculated electrostatic potential (electric charge at the molecular surface is colored with a red (negative), white (neutral, and blue (positive) color gradient; electric field extending into the solvent is shown); Trp-B43 and negatively charged loop are marked by yellow color)


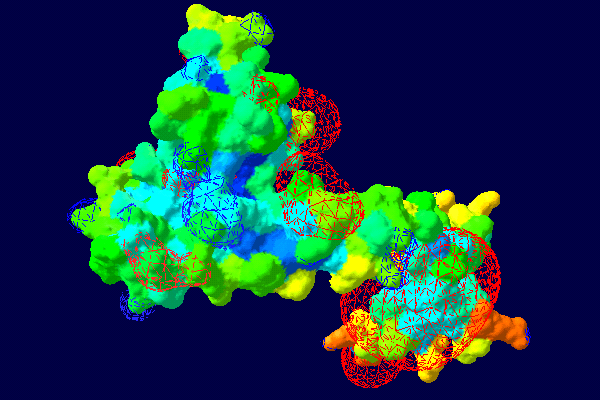

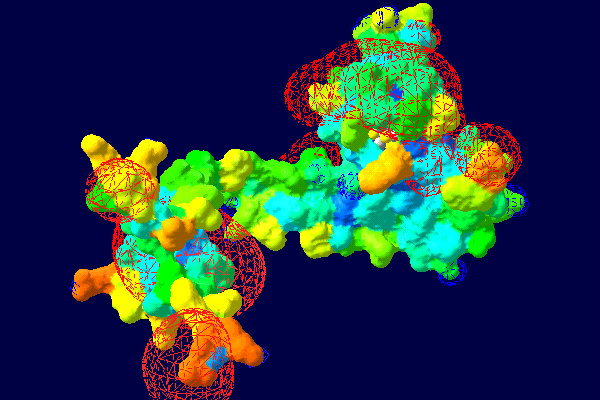


Protein molecular surface colored by calculated solvent accessibility

Candidatus Ruthia magnifica

AprB model site towards AprA model AprB model site away from AprA


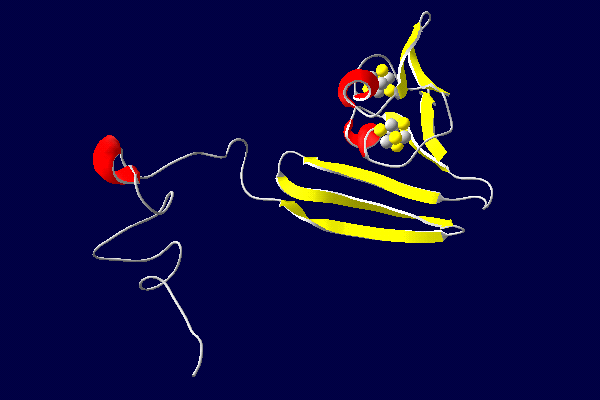


# A

# B

3D ribbon structure colored by model confidence 3D ribbon structure colored by secondary structure elements (positions of [4Fe-4S] cluster indicated)


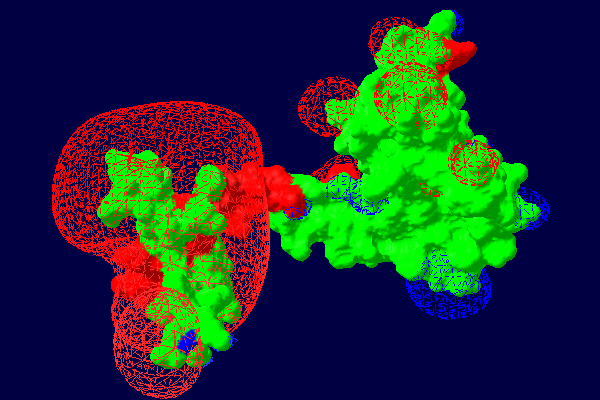

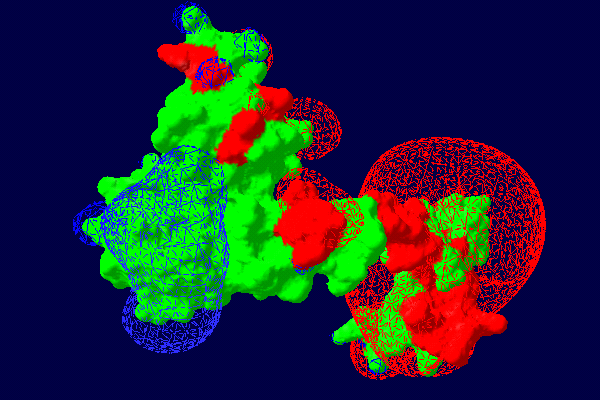


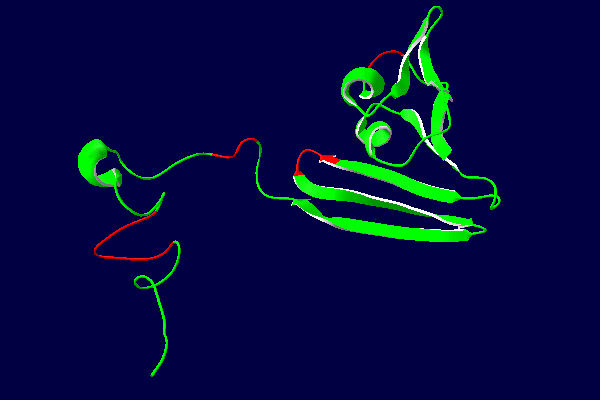


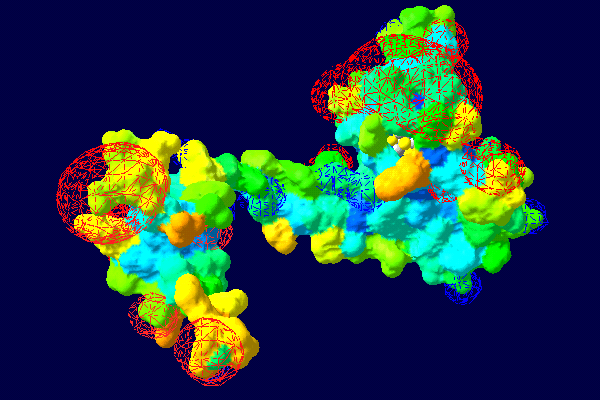

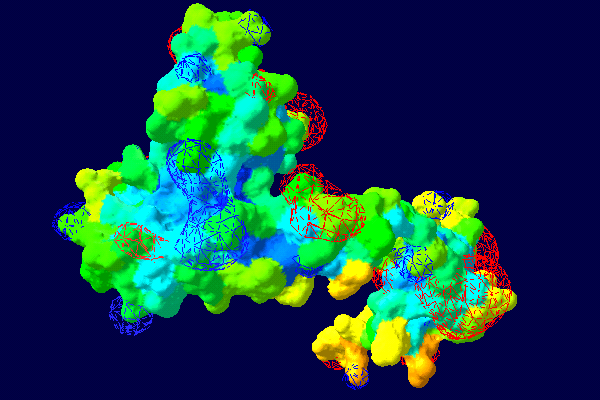
Protein molecular surface colored by model confidence


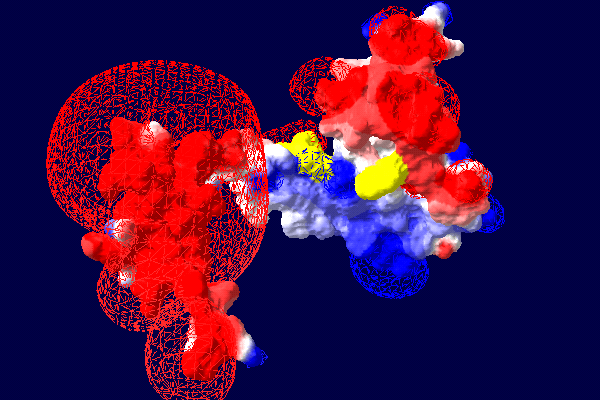

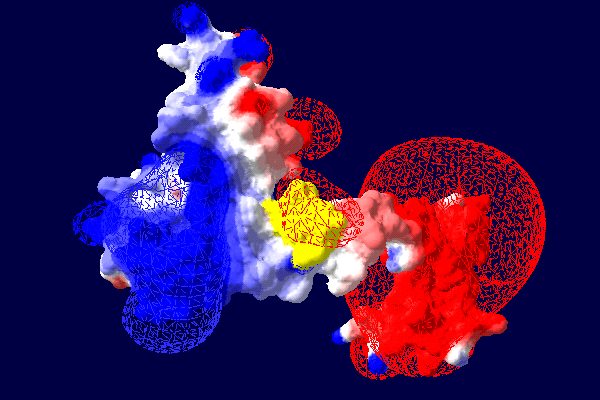


# C

# D

# E

# F

# G

# H

Protein molecular surface colored by calculated electrostatic potential (electric charge at the molecular surface is colored with a red (negative), white (neutral, and blue (positive) color gradient; electric field extending into the solvent is shown); Trp-B43 and negatively charged loop are marked by yellow color)


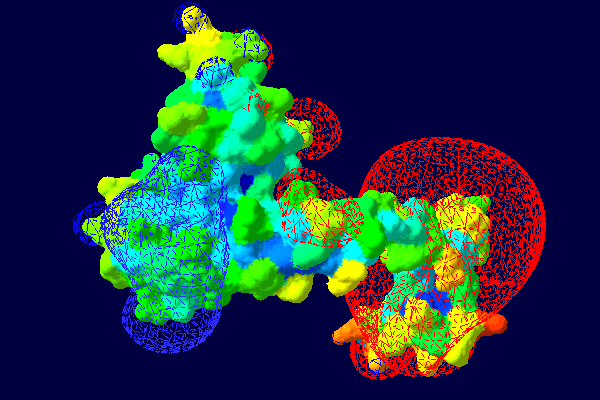

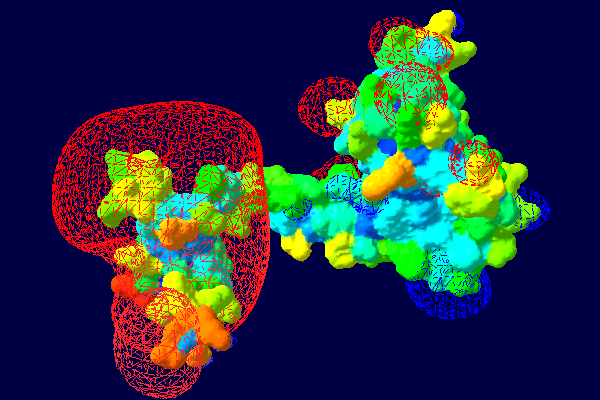


Protein molecular surface colored by calculated solvent accessibility

Pelagibacter ubique

AprB model site towards AprA model AprB model site away from AprA


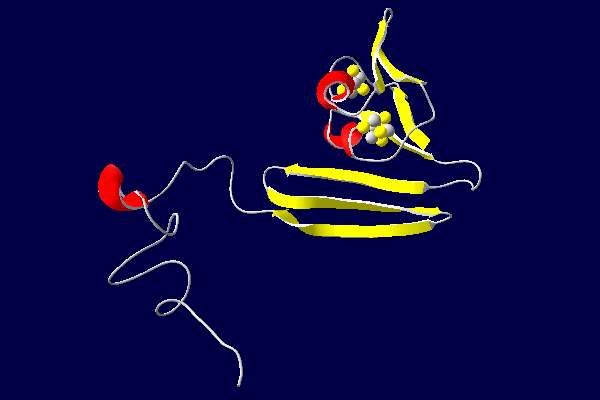


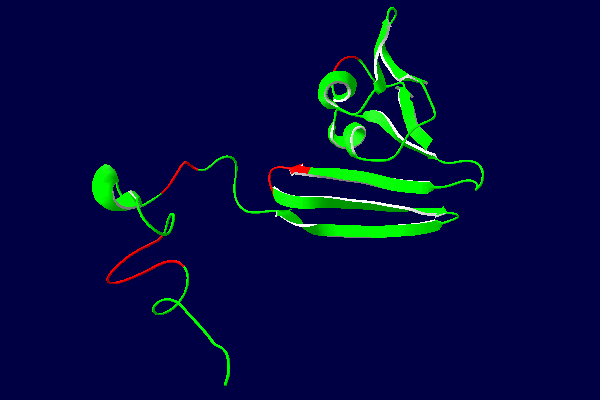


3D ribbon structure colored by model confidence 3D ribbon structure colored by secondary structure elements (positions of [4Fe-4S] cluster indicated)


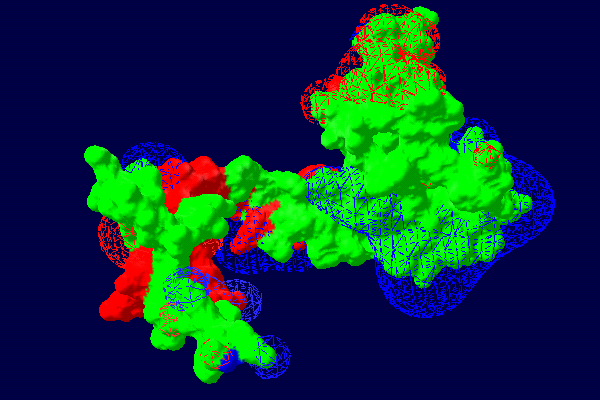

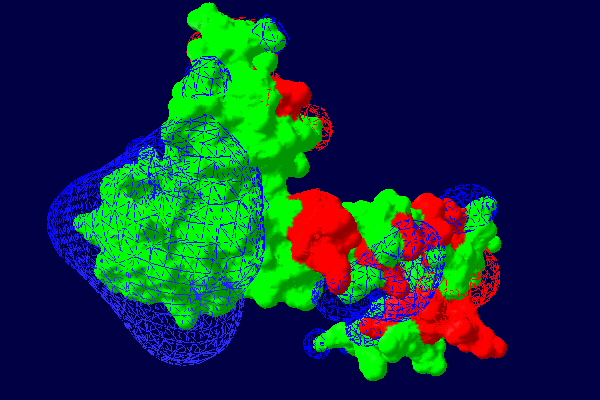


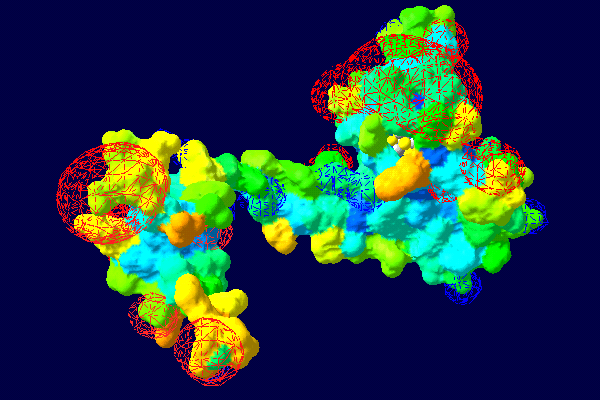

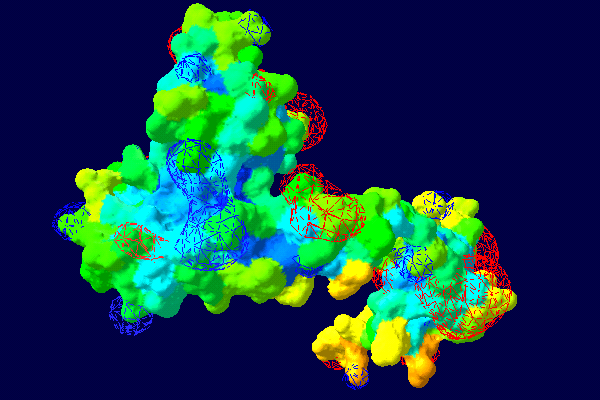
Protein molecular surface colored by model confidence

*
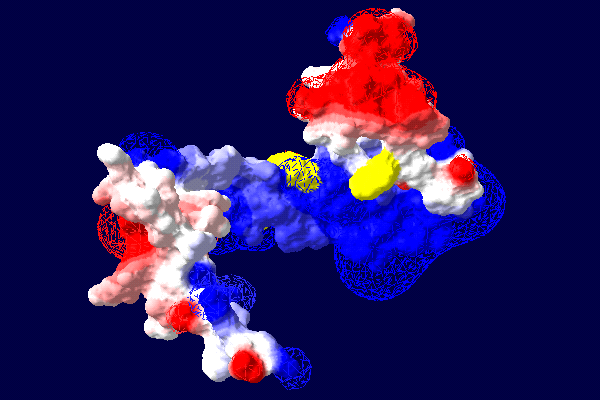

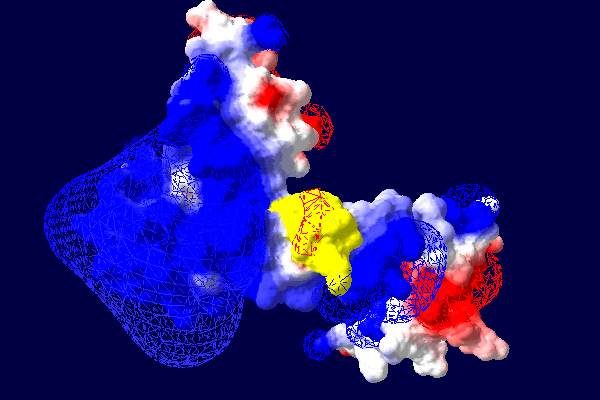
*

# A

# B

# C

# D

# E

# F

# G

# H

Protein molecular surface colored by calculated electrostatic potential (electric charge at the molecular surface is colored with a red (negative), white (neutral, and blue (positive) color gradient; electric field extending into the solvent is shown); Trp-B43 and negatively charged loop are marked by yellow color)


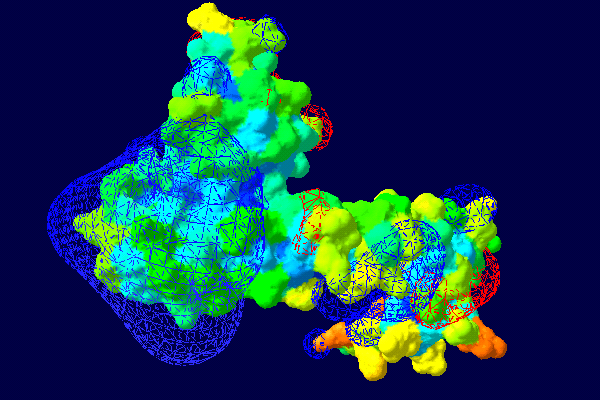

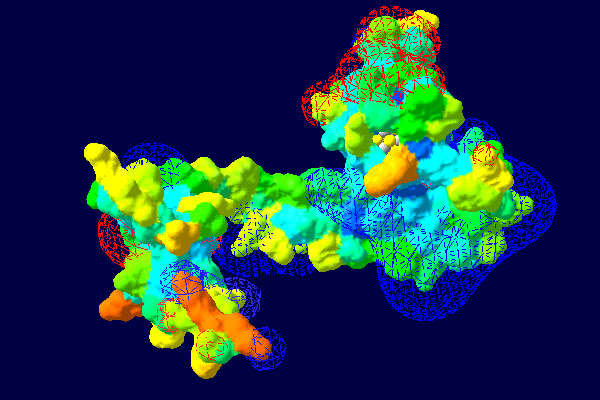


Protein molecular surface colored by calculated solvent accessibility

EBAC2C11

AprB model site towards AprA model AprB model site away from AprA


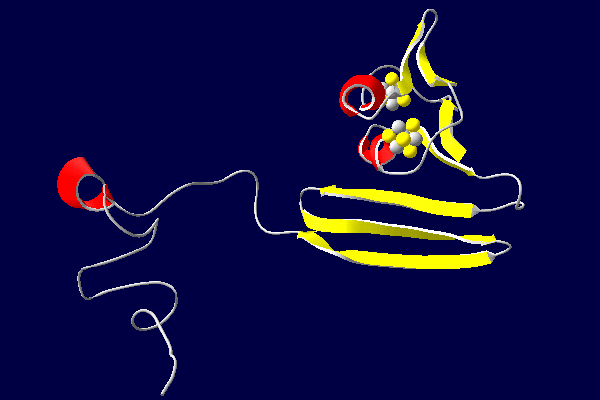


3D ribbon structure colored by model confidence 3D ribbon structure colored by secondary structure elements (positions of [4Fe-4S] cluster indicated)


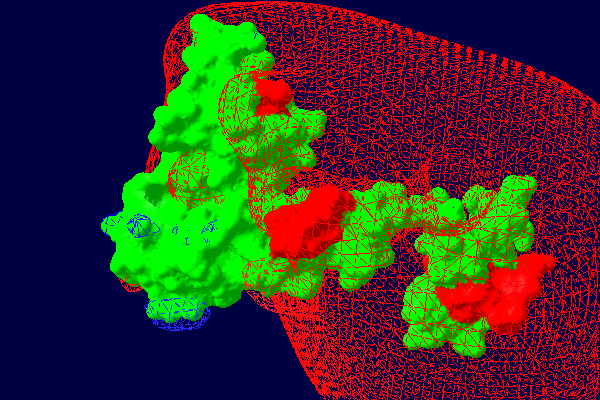

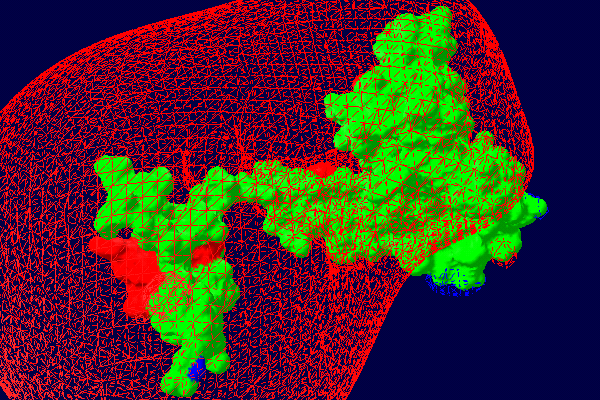


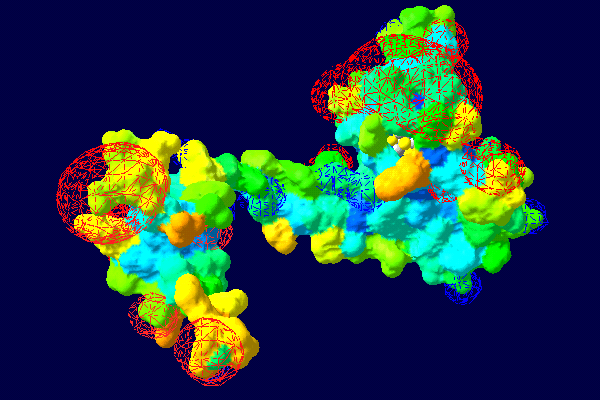

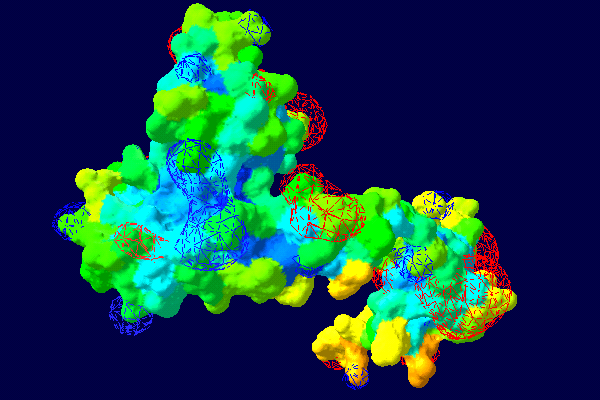
Protein molecular surface colored by model confidence


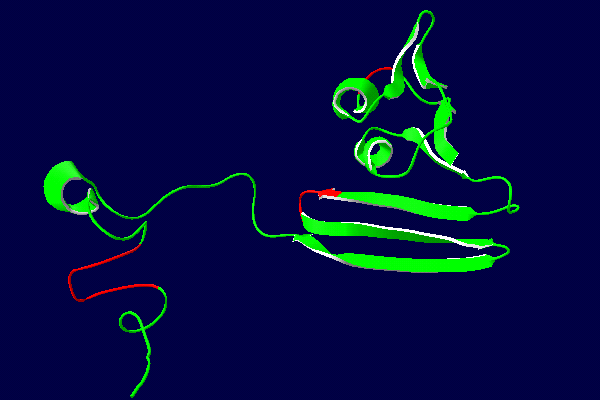


*
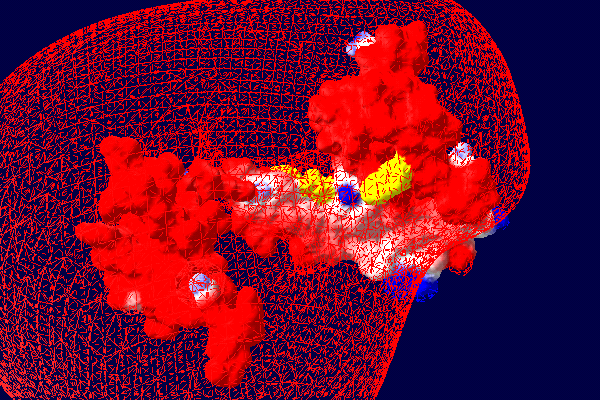

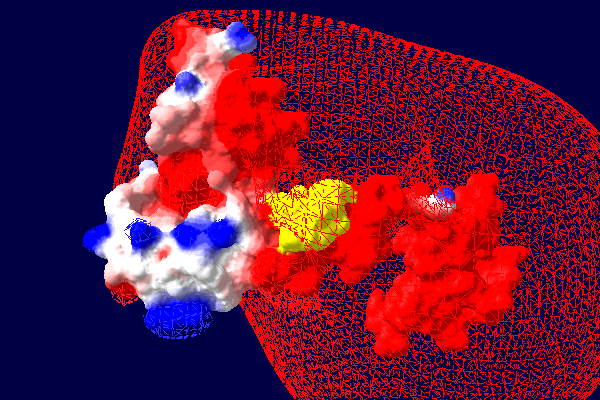
*

# A

# B

# C

# D

# E

# F

# G

# H

Protein molecular surface colored by calculated electrostatic potential (electric charge at the molecular surface is colored with a red (negative), white (neutral, and blue (positive) color gradient; electric field extending into the solvent is shown); Trp-B43 and negatively charged loop are marked by yellow color)


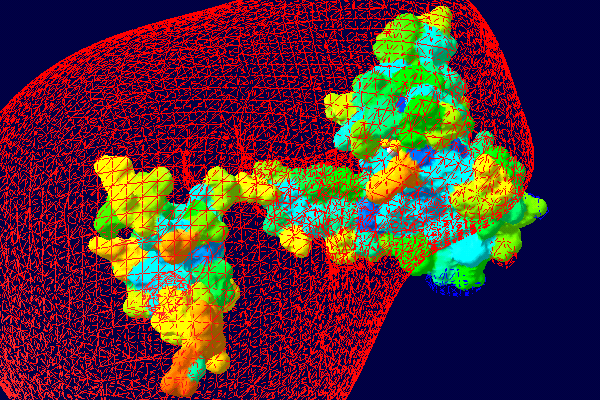

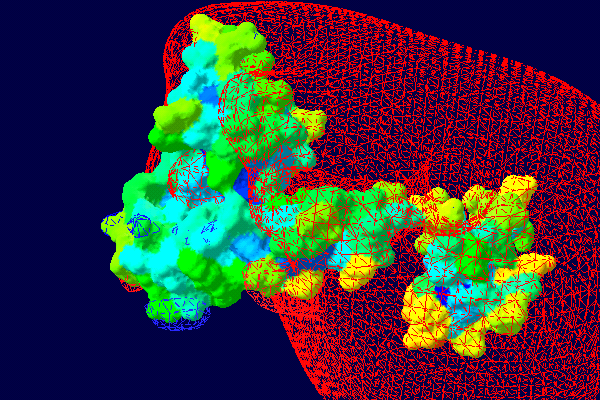


Protein molecular surface colored by calculated solvent accessibility

Crenarchaeal SRP

Pyrobaculum calidifontis

AprB model site towards AprA model AprB model site away from AprA


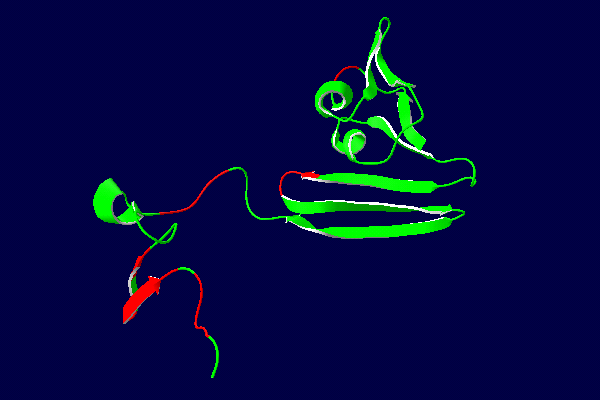

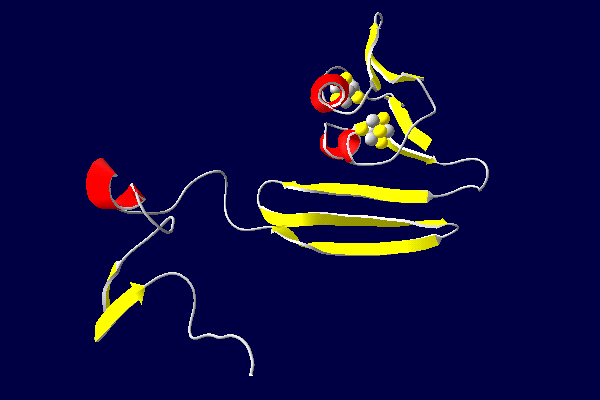


3D ribbon structure colored by model confidence 3D ribbon structure colored by secondary structure elements (positions of [4Fe-4S] cluster indicated)


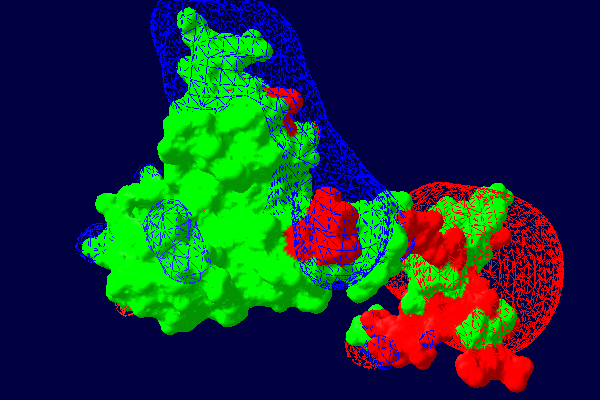

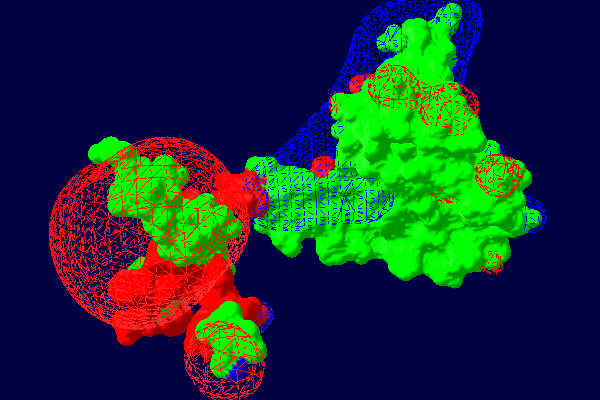


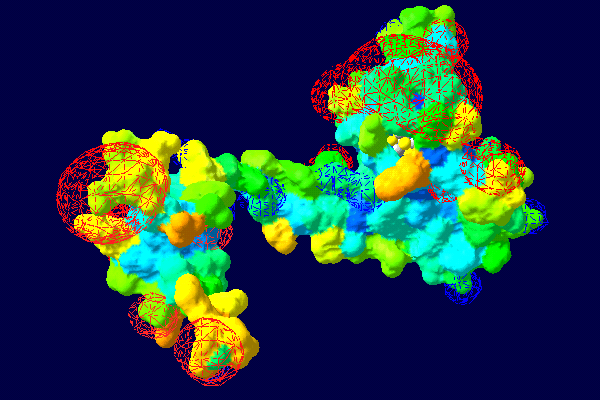

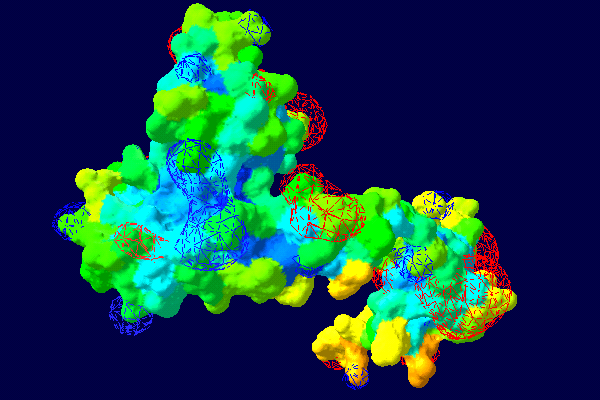
Protein molecular surface colored by model confidence

*
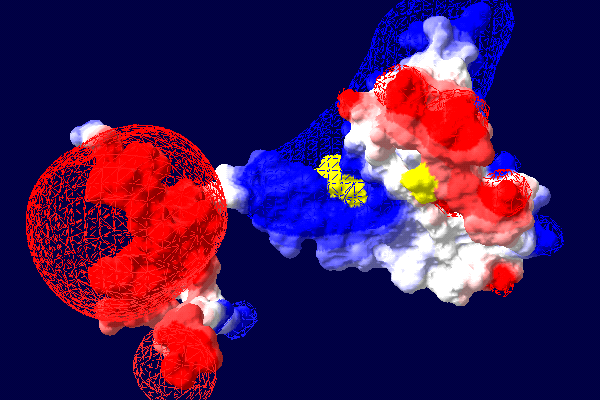

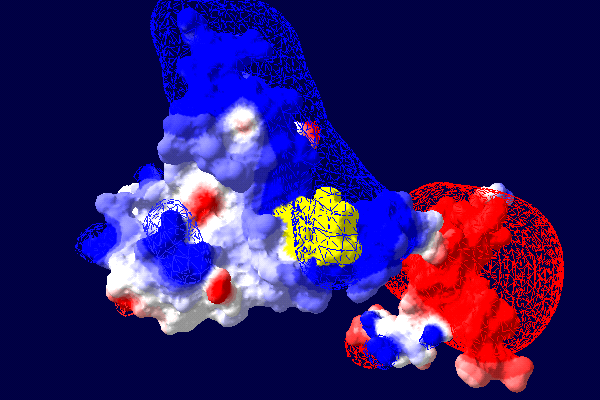
*

# A

# B

# C

# D

# E

# F

# G

# H

Protein molecular surface colored by calculated electrostatic potential (electric charge at the molecular surface is colored with a red (negative), white (neutral, and blue (positive) color gradient; electric field extending into the solvent is shown); Ala-B43 and charged loop are marked by yellow color)


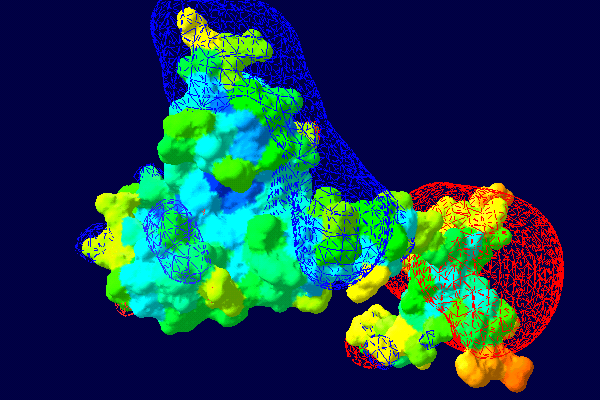

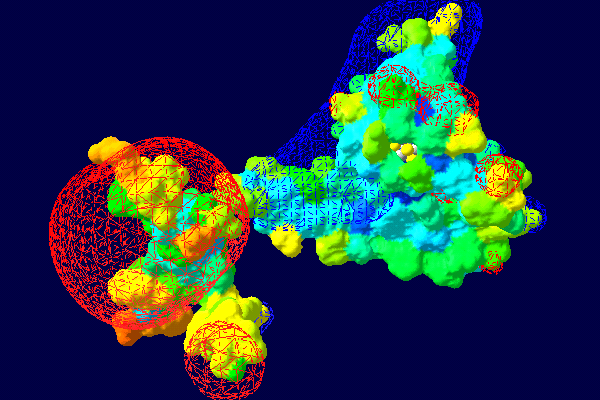


Protein molecular surface colored by calculated solvent accessibility

Caldivirga maquilingensis

AprB model site towards AprA model AprB model site away from AprA


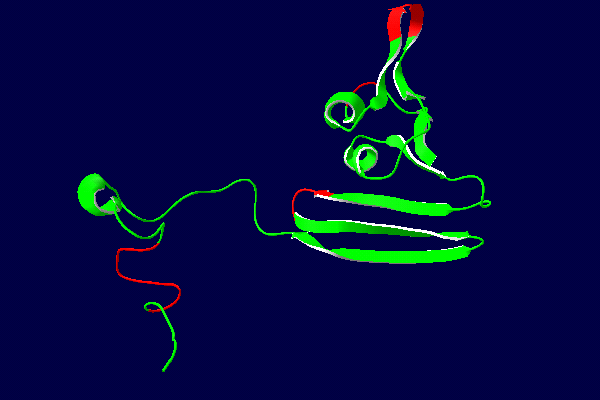

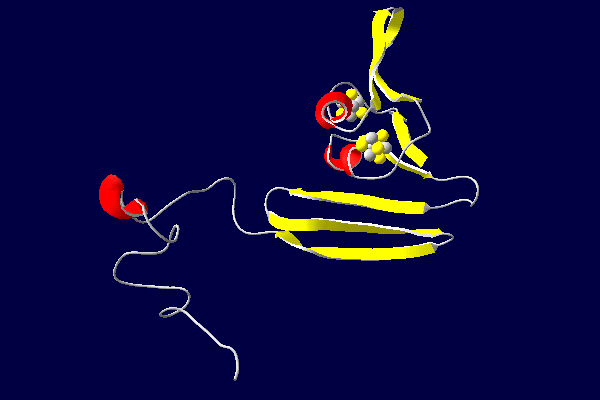


3D ribbon structure colored by model confidence 3D ribbon structure colored by secondary structure elements (positions of [4Fe-4S] cluster indicated)


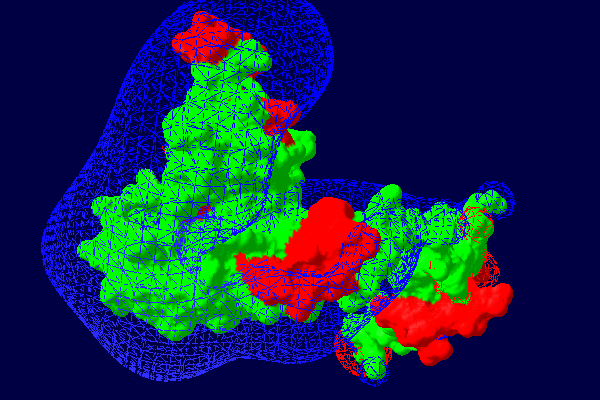

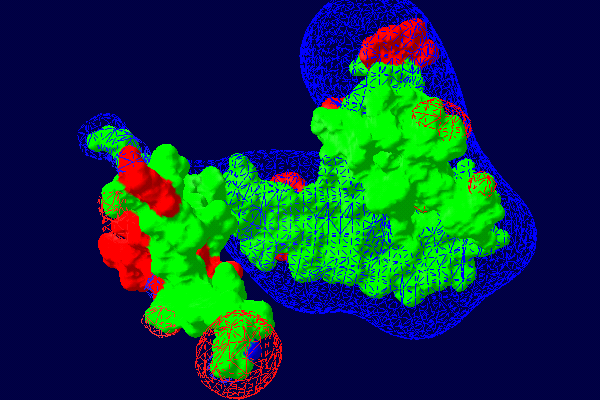


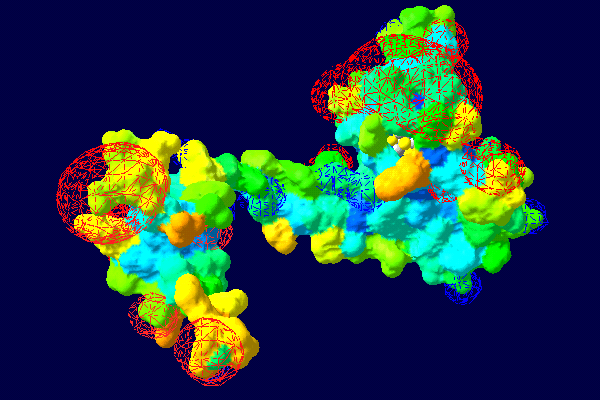

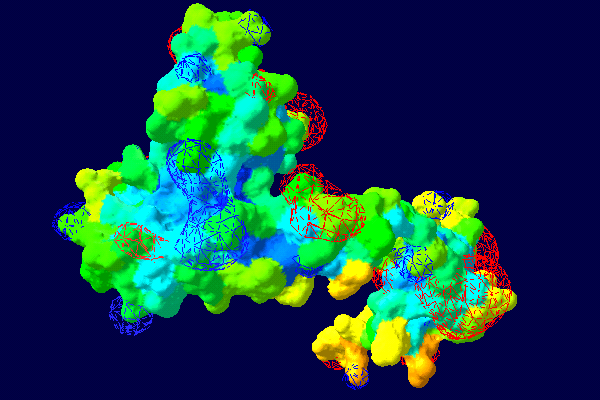
Protein molecular surface colored by model confidence


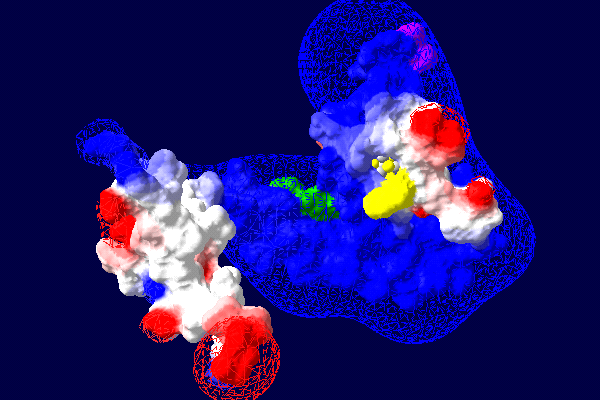

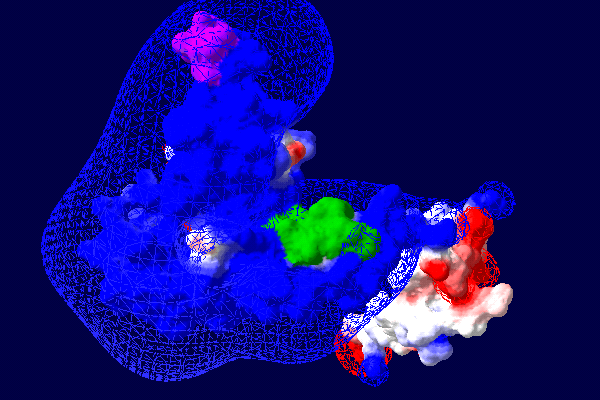


Protein molecular surface colored by calculated electrostatic potential (electric charge at the molecular surface is colored with a red (negative), white (neutral, and blue (positive) color gradient; electric field extending into the solvent is shown); Trp-B43 and charged loop are marked by green color; additional loop is colored in violett)


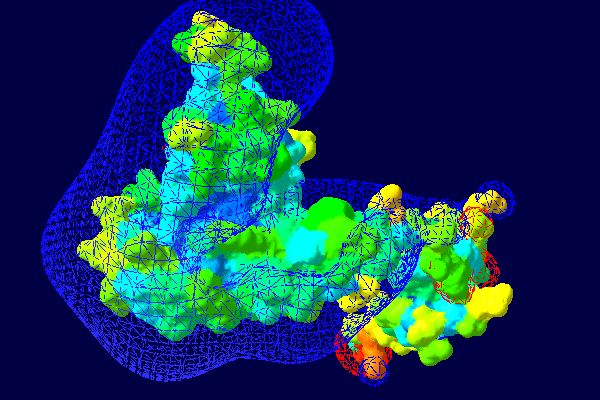

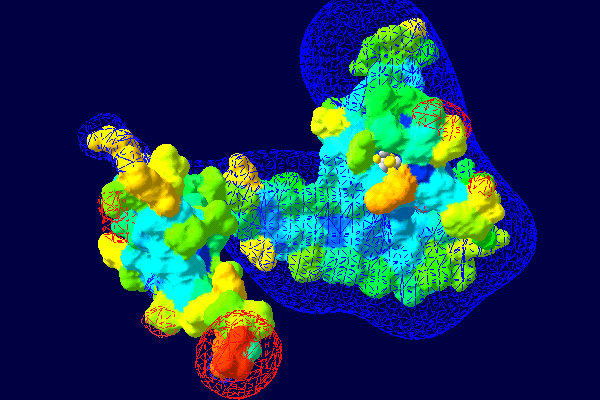


# A

# B

# C

# D

# E

# F

# G

# H

Protein molecular surface colored by calculated solvent accessibility

SRB and related SOB Apr lineage II

Desulfotomaculum reducens

AprB model site towards AprA model AprB model site away from AprA


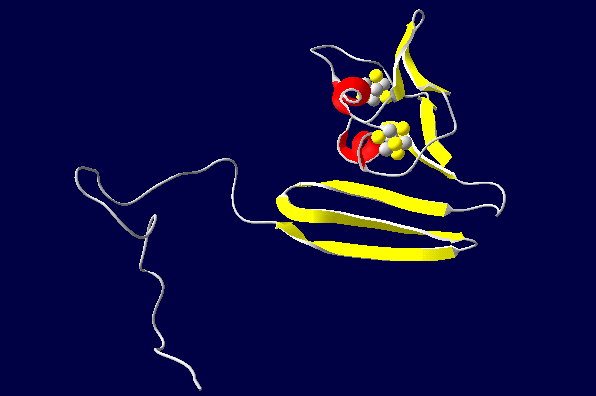


3D ribbon structure colored by model confidence 3D ribbon structure colored by secondary structure elements (positions of [4Fe-4S] cluster indicated)


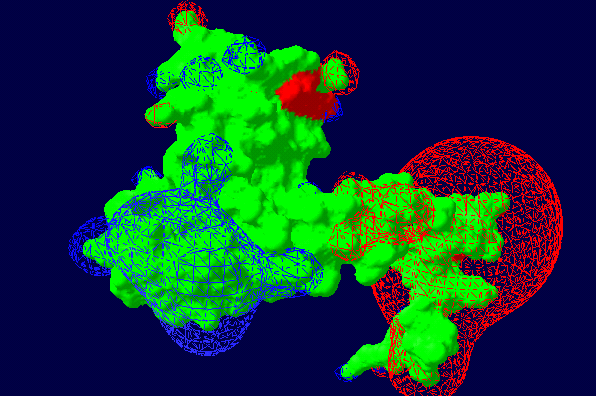

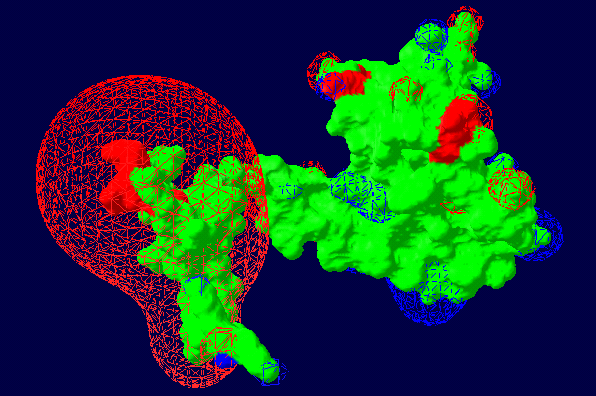


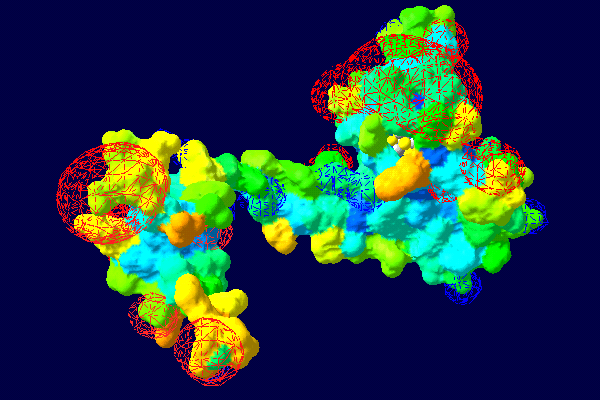

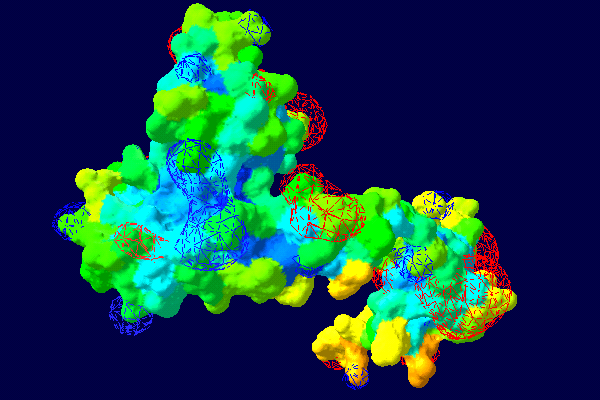
Protein molecular surface colored by model confidence


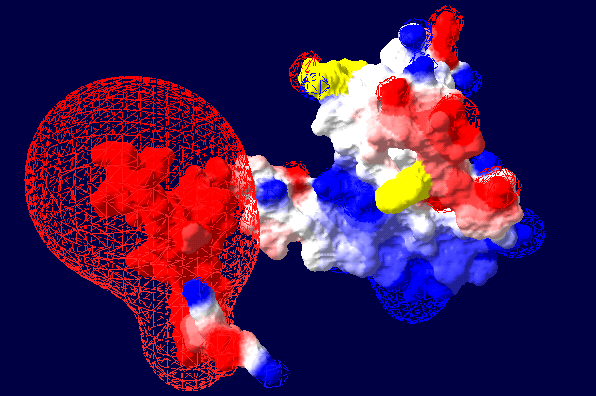

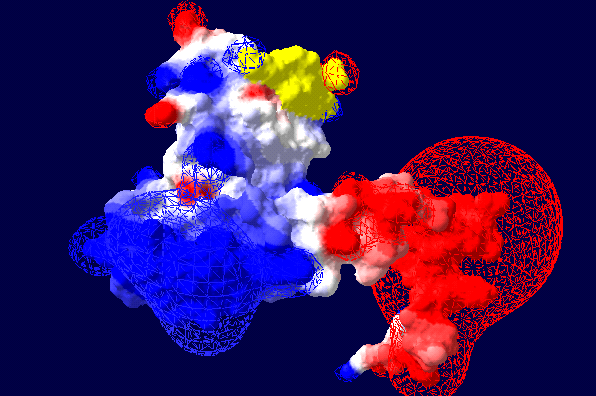


# A

# B

# C

# D

# E

# F

# G

# H


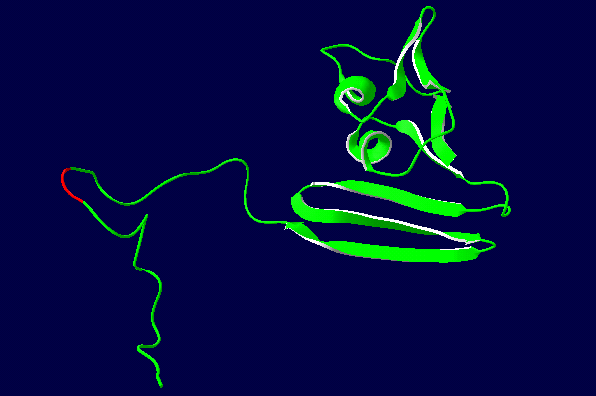
Protein molecular surface colored by calculated electrostatic potential (electric charge at the molecular surface is colored with a red (negative), white (neutral, and blue (positive) color gradient; electric field extending into the solvent is shown); Trp-B48 and exposed, potential Qmo-docking loop are marked by yellow color)


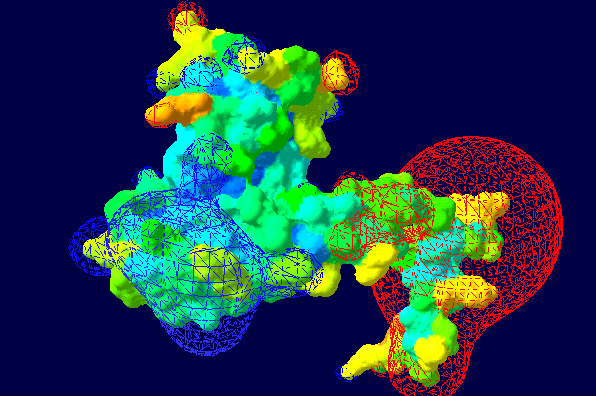

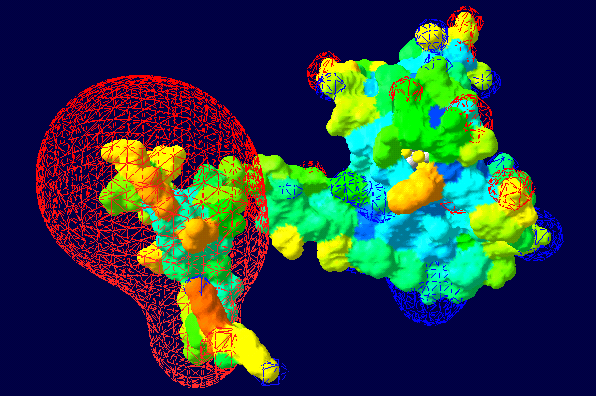


Protein molecular surface colored by calculated solvent accessibility

Syntrophobacter fumaroxidans

AprB model site towards AprA model AprB model site away from AprA


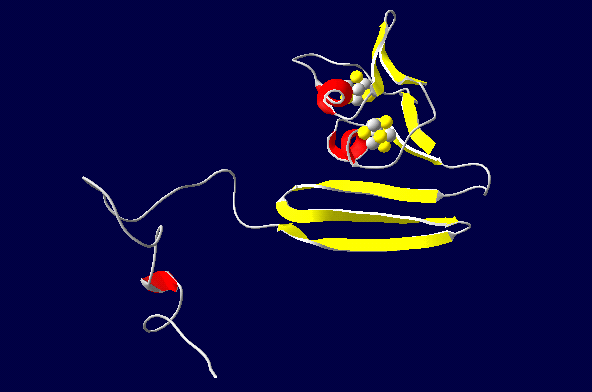


3D ribbon structure colored by model confidence 3D ribbon structure colored by secondary structure elements (positions of [4Fe-4S] cluster indicated)


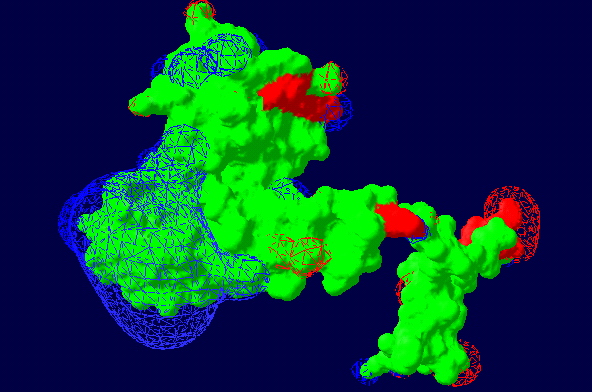

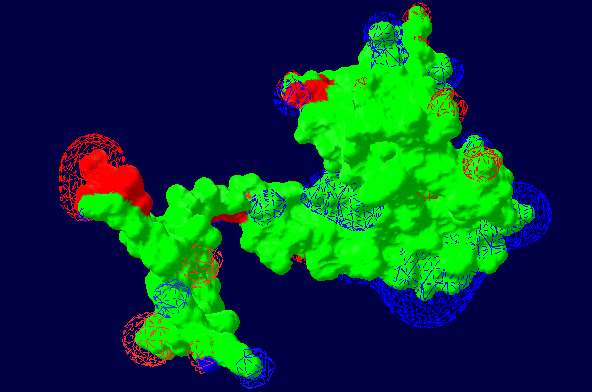


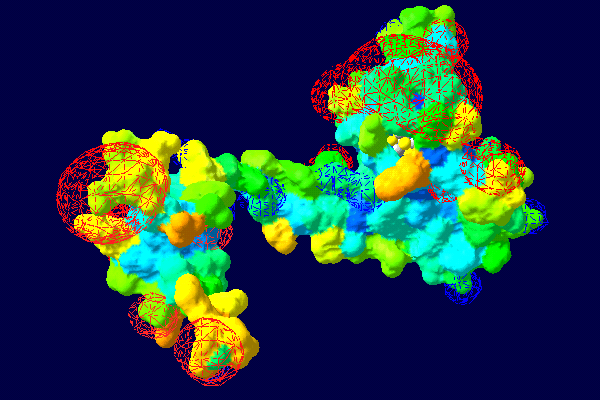

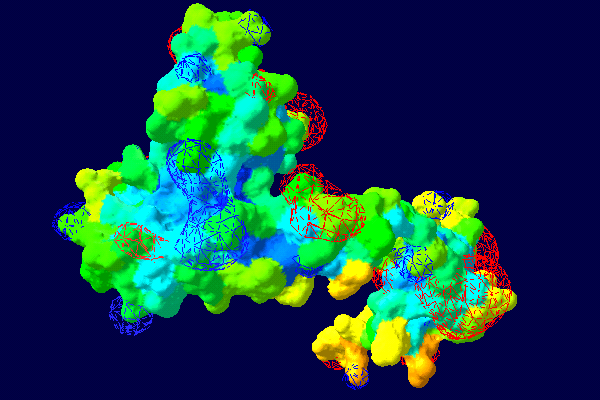
Protein molecular surface colored by model confidence


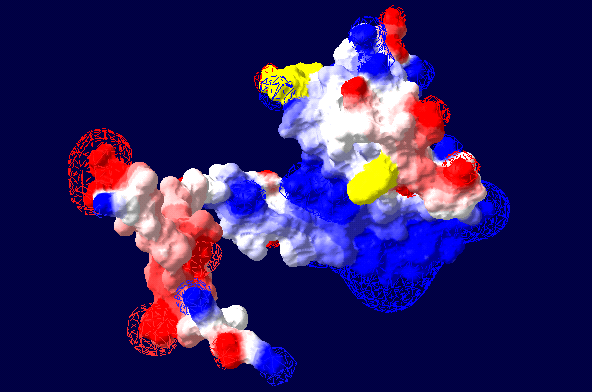

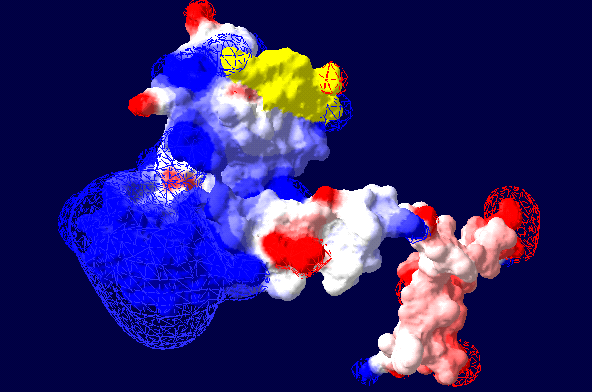


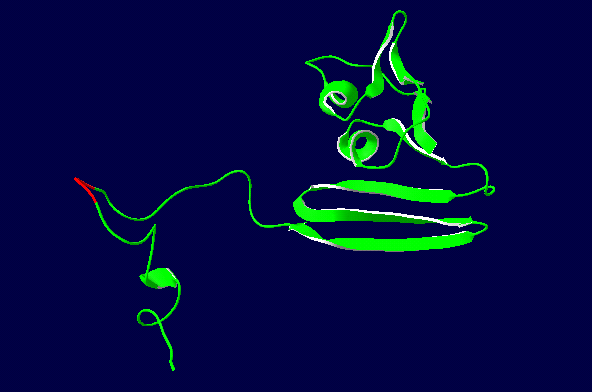


Protein molecular surface colored by calculated electrostatic potential (electric charge at the molecular surface is colored with a red (negative), white (neutral, and blue (positive) color gradient; electric field extending into the solvent is shown); Trp-B48 and exposed, potential Qmo-docking loop are marked by yellow color)


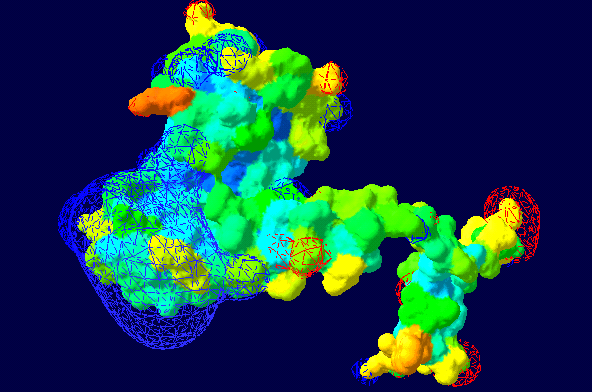

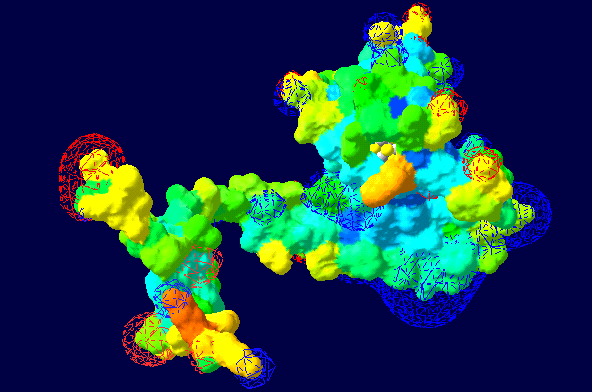


# A

# B

# C

# D

# E

# F

# G

# H

Protein molecular surface colored by calculated solvent accessibility

Fosws39f8

AprB model site towards AprA model AprB model site away from AprA

*
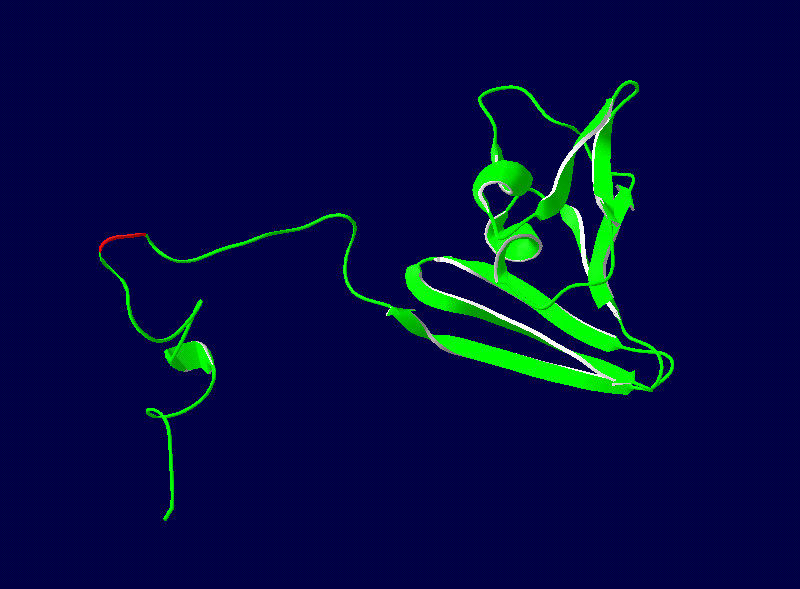

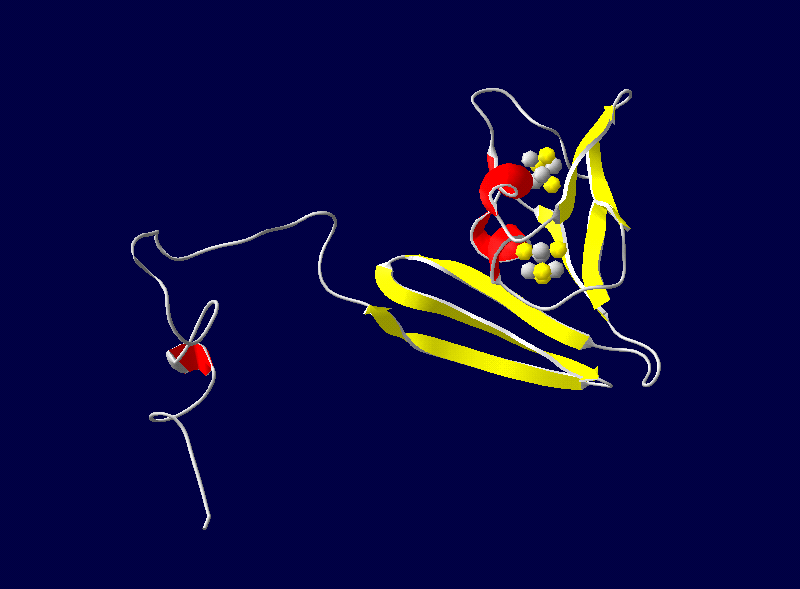
*

# A

# B

# C

# D

# E

# F

# G

# H

3D ribbon structure colored by model confidence 3D ribbon structure colored by secondary structure elements (positions of [4Fe-4S] cluster indicated)

Protein molecular surface colored by model confidence

Protein molecular surface colored by calculated electrostatic potential (electric charge at the molecular surface is colored with a red (negative), white (neutral, and blue (positive) color gradient; electric field extending into the solvent is shown); Trp-B48 and exposed, potential Qmo-docking loop are marked by yellow color)

Protein molecular surface colored by calculated solvent accessibility

Fosws7f8

AprB model site towards AprA model AprB model site away from AprA

3D ribbon structure colored by model confidence 3D ribbon structure colored by secondary structure elements (positions of [4Fe-4S] cluster indicated)

Protein molecular surface colored by model confidence

# A

# B

# C

# D

# E

# F

# G

# H

Protein molecular surface colored by calculated electrostatic potential (electric charge at the molecular surface is colored with a red (negative), white (neutral, and blue (positive) color gradient; electric field extending into the solvent is shown); Trp-B48 and exposed, potential Qmo-docking loop are marked by yellow color)

Protein molecular surface colored by calculated solvent accessibility

Thermodesulfobacterium commune

AprB model site towards AprA model AprB model site away from AprA

3D ribbon structure colored by model confidence 3D ribbon structure colored by secondary structure elements (positions of [4Fe-4S] cluster indicated)

Protein molecular surface colored by model confidence

# A

# B

# C

# D

# E

# F

# G

# H

Protein molecular surface colored by calculated electrostatic potential (electric charge at the molecular surface is colored with a red (negative), white (neutral, and blue (positive) color gradient; electric field extending into the solvent is shown); Trp-B48 and exposed, potential Qmo-docking loop are marked by yellow color)

Protein molecular surface colored by calculated solvent accessibility

Desulfovibrio vulgaris

AprB model site towards AprA model AprB model site away from AprA

3D ribbon structure colored by model confidence 3D ribbon structure colored by secondary structure elements (positions of [4Fe-4S] cluster indicated)

Protein molecular surface colored by model confidence

Protein molecular surface colored by calculated electrostatic potential (electric charge at the molecular surface is colored with a red (negative), white (neutral, and blue (positive) color gradient; electric field extending into the solvent is shown); Trp-B48 and exposed, potential Qmo-docking loop are marked by yellow color)

# A

# B

# C

# D

# E

# F

# G

# H

Protein molecular surface colored by calculated solvent accessibility

Desulfovibrio desulfuricans

AprB model site towards AprA model AprB model site away from AprA

3D ribbon structure colored by model confidence 3D ribbon structure colored by secondary structure elements (positions of [4Fe-4S] cluster indicated)

Protein molecular surface colored by model confidence

Protein molecular surface colored by calculated electrostatic potential (electric charge at the molecular surface is colored with a red (negative), white (neutral, and blue (positive) color gradient; electric field extending into the solvent is shown); Trp-B48 and exposed, potential Qmo-docking loop are marked by yellow color)

# A

# B

# C

# D

# E

# F

# G

# H

Protein molecular surface colored by calculated solvent accessibility

Desulfobulbus sp. str. MLMS-1

AprB model site towards AprA model AprB model site away from AprA

# A

# B

# C

# D

# E

# F

# G

# H

3D ribbon structure colored by model confidence 3D ribbon structure colored by secondary structure elements (positions of [4Fe-4S] cluster indicated)

Protein molecular surface colored by model confidence

Protein molecular surface colored by calculated electrostatic potential (electric charge at the molecular surface is colored with a red (negative), white (neutral, and blue (positive) color gradient; electric field extending into the solvent is shown); Trp-B48 and exposed, potential Qmo-docking loop are marked by yellow color)

Protein molecular surface colored by calculated solvent accessibility

Desulfotalea psychrophila

AprB model site towards AprA model AprB model site away from AprA

3D ribbon structure colored by model confidence 3D ribbon structure colored by secondary structure elements (positions of [4Fe-4S] cluster indicated)

Protein molecular surface colored by model confidence

# A

# B

# C

# D

# E

# F

# G

# H

Protein molecular surface colored by calculated electrostatic potential (electric charge at the molecular surface is colored with a red (negative), white (neutral, and blue (positive) color gradient; electric field extending into the solvent is shown); Trp-B48 and exposed, potential Qmo-docking loop are marked by yellow color)

Protein molecular surface colored by calculated solvent accessibility

Olavius algarvensis Delta 1 symbiont

AprB model site towards AprA model AprB model site away from AprA

# A

# B

# C

# D

# E

# F

# G

# H

3D ribbon structure colored by model confidence 3D ribbon structure colored by secondary structure elements (positions of [4Fe-4S] cluster indicated)

Protein molecular surface colored by model confidence

Protein molecular surface colored by calculated electrostatic potential (electric charge at the molecular surface is colored with a red (negative), white (neutral, and blue (positive) color gradient; electric field extending into the solvent is shown); Trp-B48 and exposed, potential Qmo-docking loop are marked by yellow color)

Protein molecular surface colored by calculated solvent accessibility

Thermodesulfovibrio yellowstonii

AprB model site towards AprA model AprB model site away from AprA

3D ribbon structure colored by model confidence 3D ribbon structure colored by secondary structure elements (positions of [4Fe-4S] cluster indicated)

Protein molecular surface colored by model confidence

# A

# B

# C

# D

# E

# F

# G

# H

Protein molecular surface colored by calculated electrostatic potential (electric charge at the molecular surface is colored with a red (negative), white (neutral, and blue (positive) color gradient; electric field extending into the solvent is shown); Trp-B48 and exposed, potential Qmo-docking loop are marked by yellow color)

Protein molecular surface colored by calculated solvent accessibility

Chlorobaculum tepidum

AprB model site towards AprA model AprB model site away from AprA

3D ribbon structure colored by model confidence 3D ribbon structure colored by secondary structure elements (positions of [4Fe-4S] cluster indicated)

Protein molecular surface colored by model confidence

Protein molecular surface colored by calculated electrostatic potential (electric charge at the molecular surface is colored with a red (negative), white (neutral, and blue (positive) color gradient; electric field extending into the solvent is shown); Trp-B48 and exposed, potential Qmo-docking loop are marked by yellow color)

# A

# B

# C

# D

# E

# F

# G

# H

Protein molecular surface colored by calculated solvent accessibility

Thiobacillus denitrificans

AprB model site towards AprA model AprB model site away from AprA

3D ribbon structure colored by model confidence 3D ribbon structure colored by secondary structure elements (positions of [4Fe-4S] cluster indicated)

rotein molecular surface colored by model confidence

Protein molecular surface colored by calculated electrostatic potential (electric charge at the molecular surface is colored with a red (negative), white (neutral, and blue (positive) color gradient; electric field extending into the solvent is shown); Trp-B54 and exposed, potential Qmo-docking loop are marked by yellow color; additional loop is colored violett

# A

# B

# C

# D

# E

# F

# G

# H

Protein molecular surface colored by calculated solvent accessibility
