# Supplementary material for: Homology Modeling of Dissimilatory APS Reductases (AprBA) of Sulfur-Oxidizing and Sulfate-Reducing Prokaryotes
Source: PLoS One. 2008 Jan 30;3(1):e1514. doi: 10.1371/journal.pone.0001514 (PMC2211403; doi:10.1371/journal.pone.0001514)
Supplement: Figure S2 — (2.17 MB DOC) [file pone.0001514.s002.doc]

**Supplementary data material Figure S2. AprB protein matrix surrounding the [4Fe-4S] clusters**

Reference Residues in a distance <5.0Å to the Charged and polar residues marked Cysteine and tryptophane marked

structure [4Fe-4S] clusters I and II


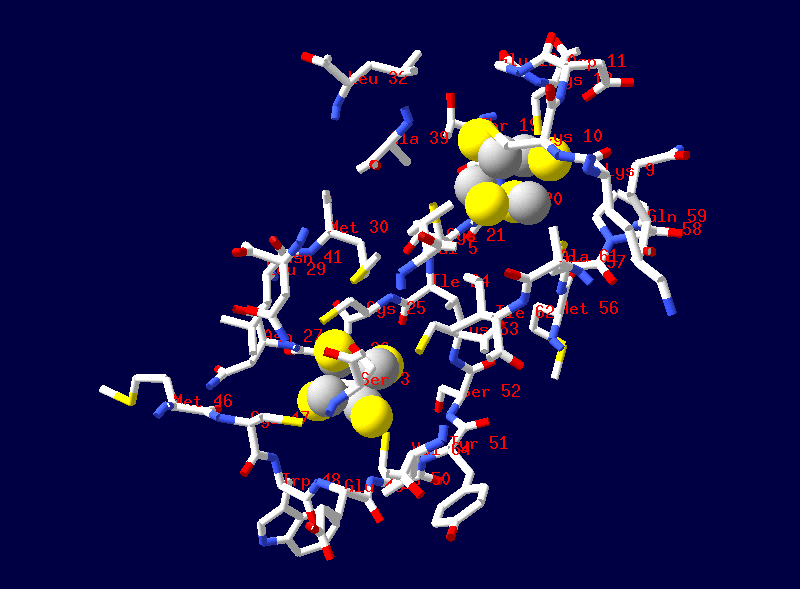

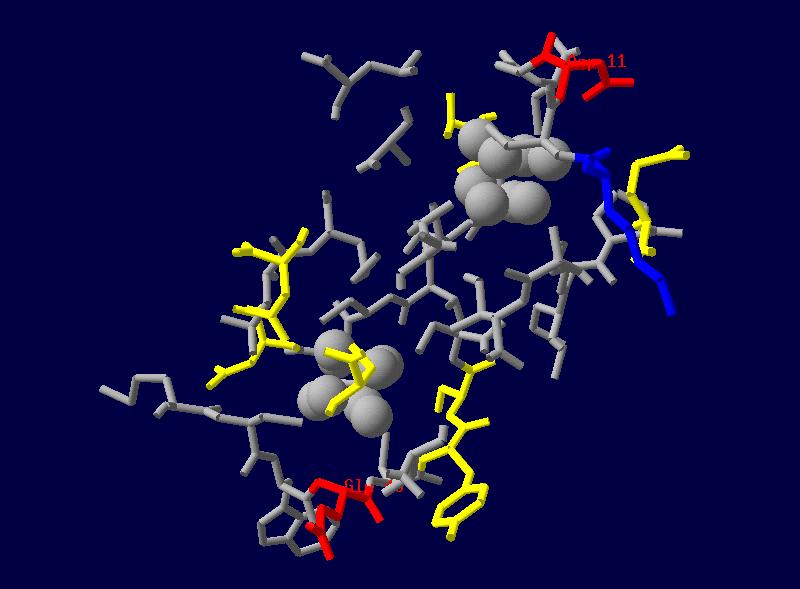

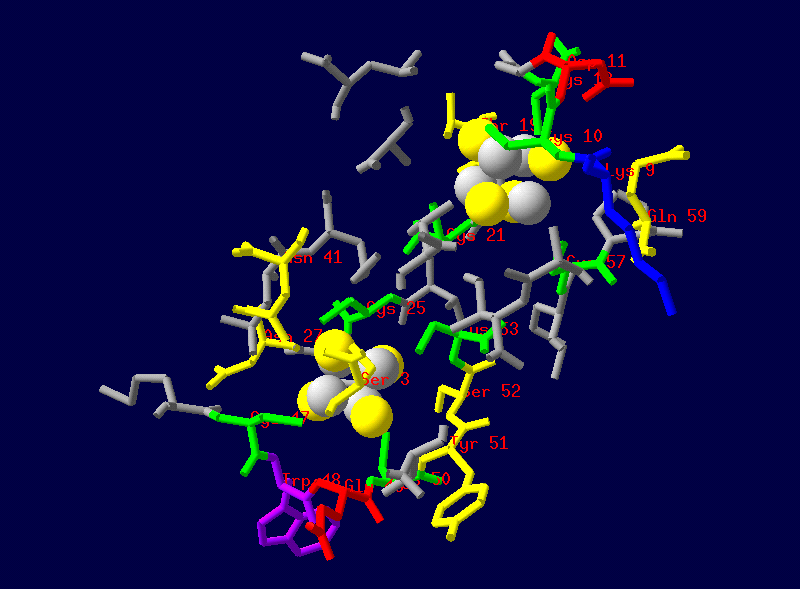


Archaeoglobus

*fulgidus*


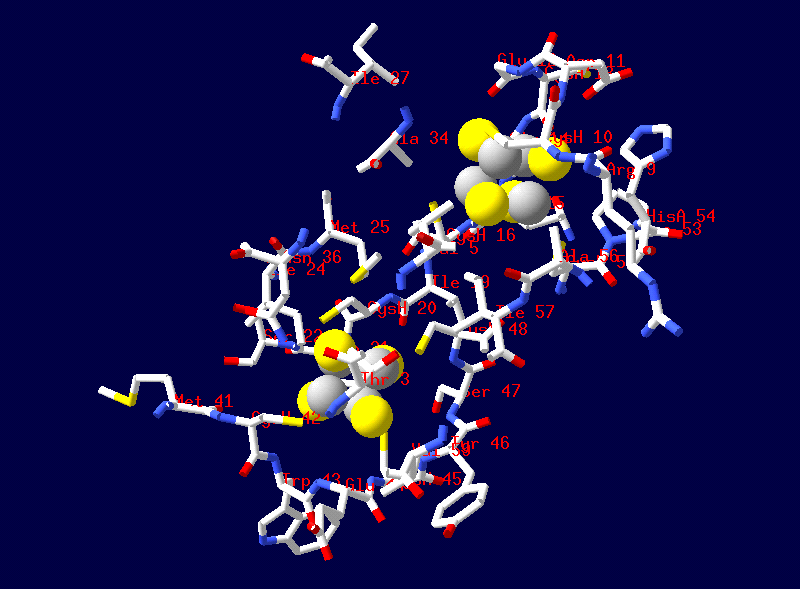

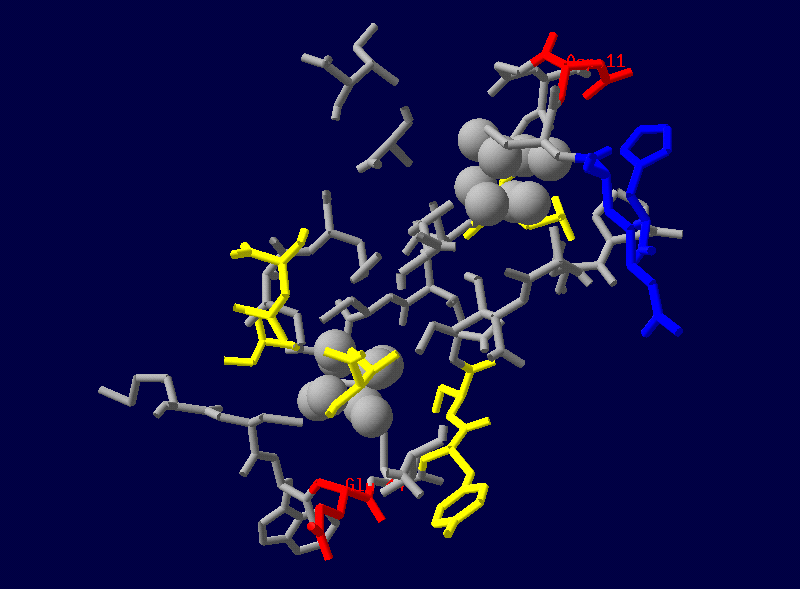

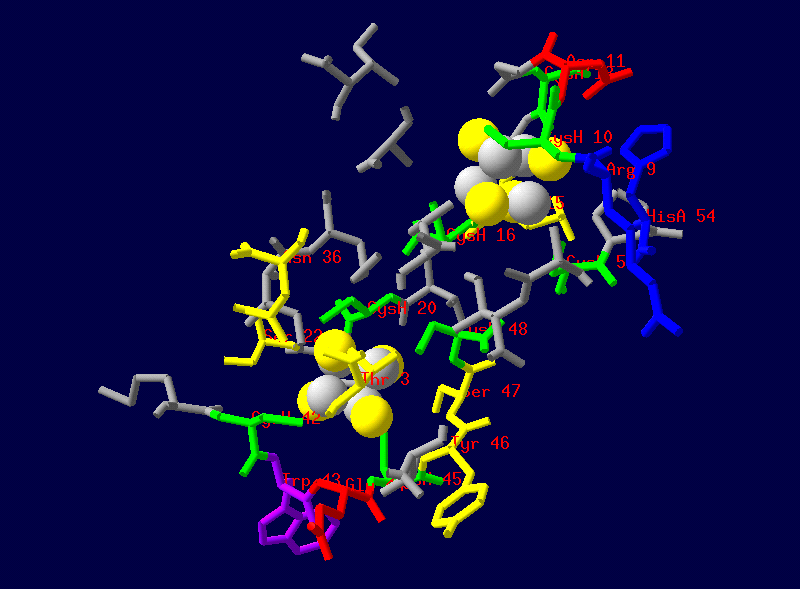

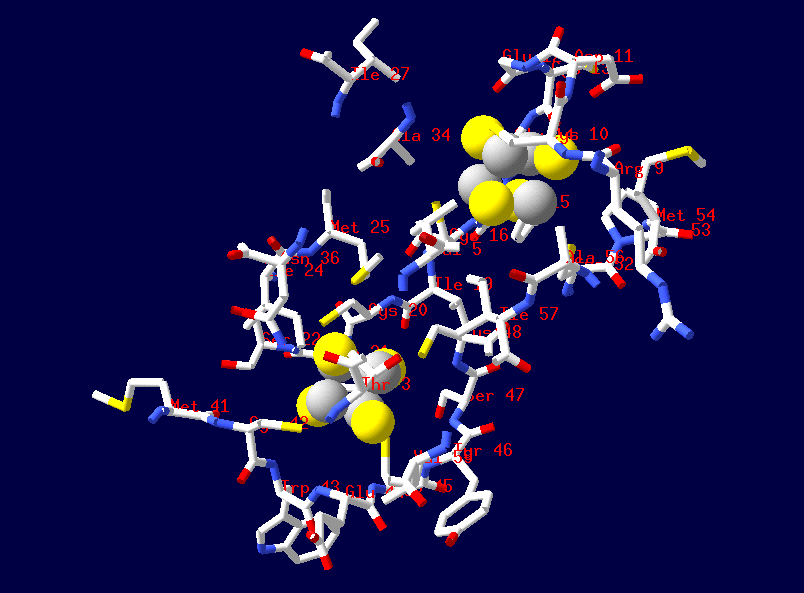
SOB lineage I

Allochromatium

*vinosum*


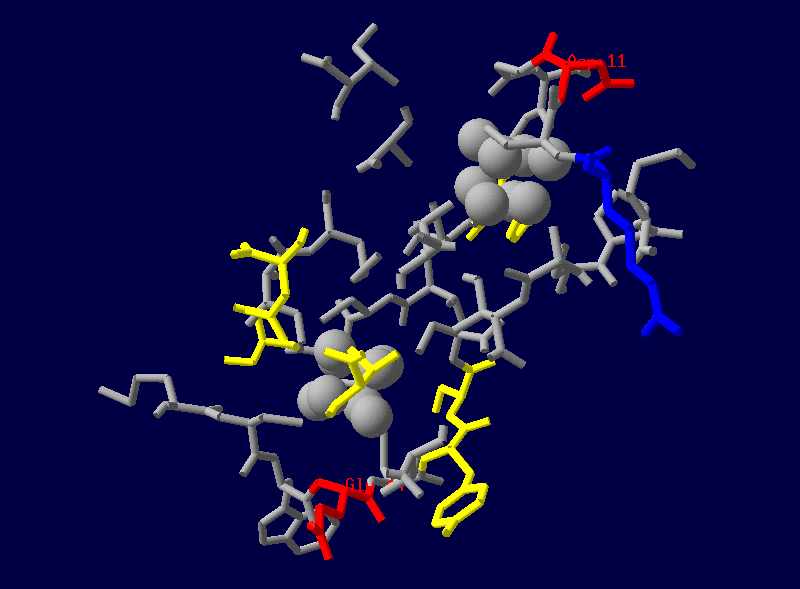

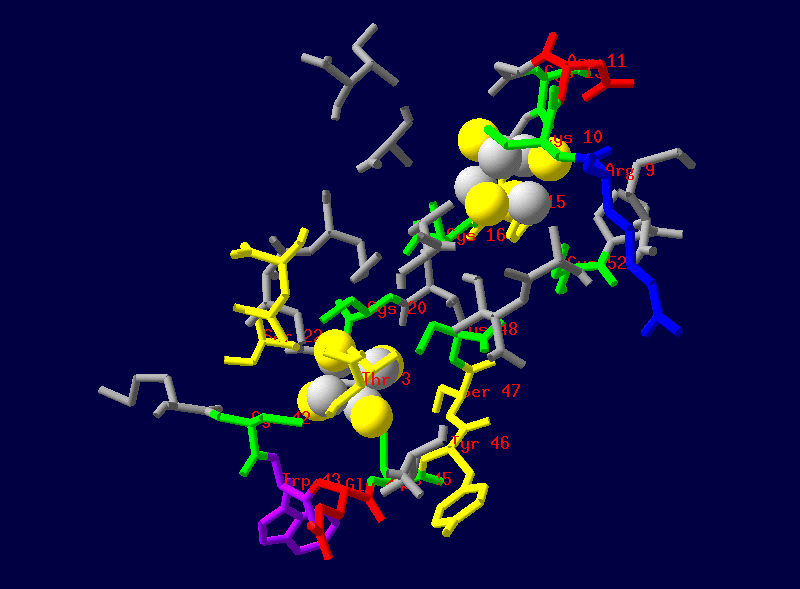
*Thiobacillus*

*denitrificans*

SOB lineage I Residues in a distance <5.0Å to the Charged and polar residues marked Cysteine and tryptophan marked

[4Fe-4S] clusters

*
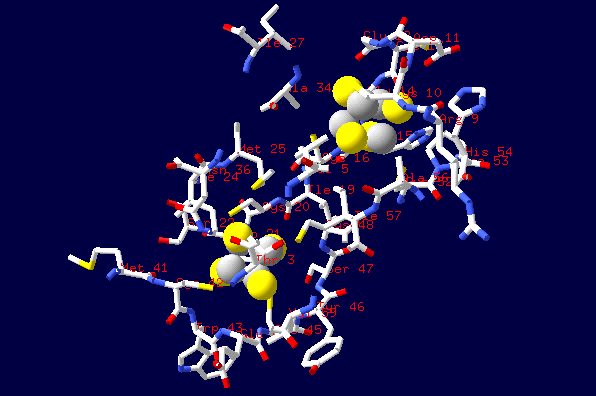

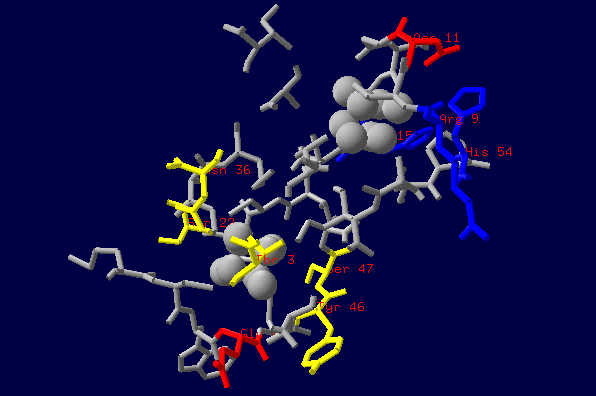

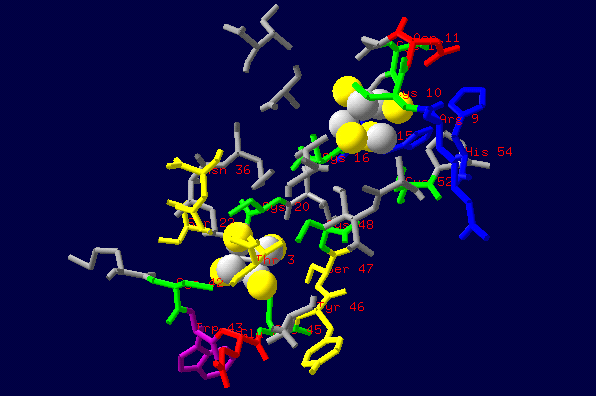
*

*Cdt.* Ruthia

magnifica

*
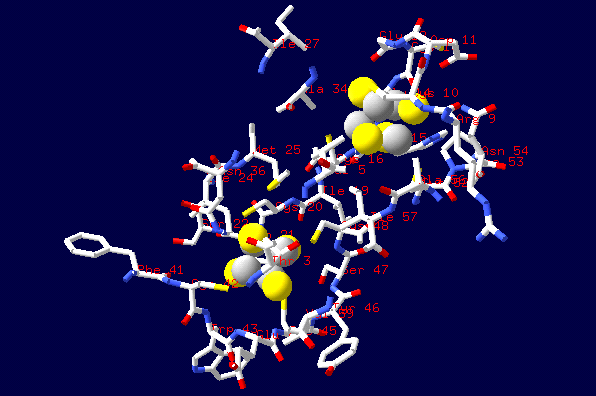

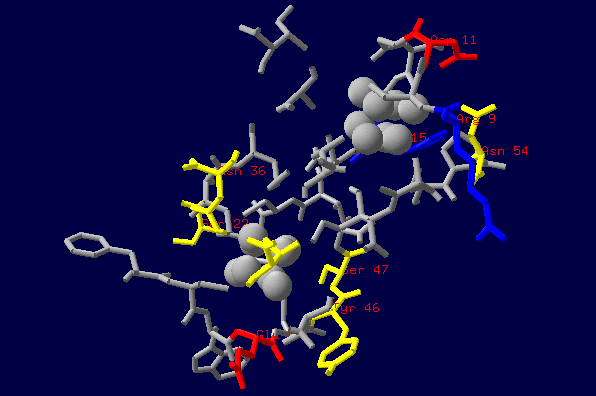

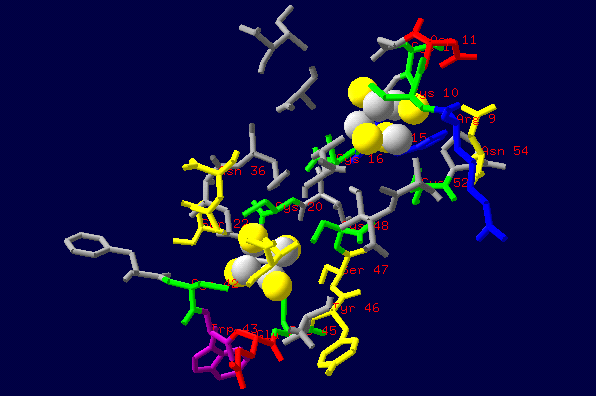

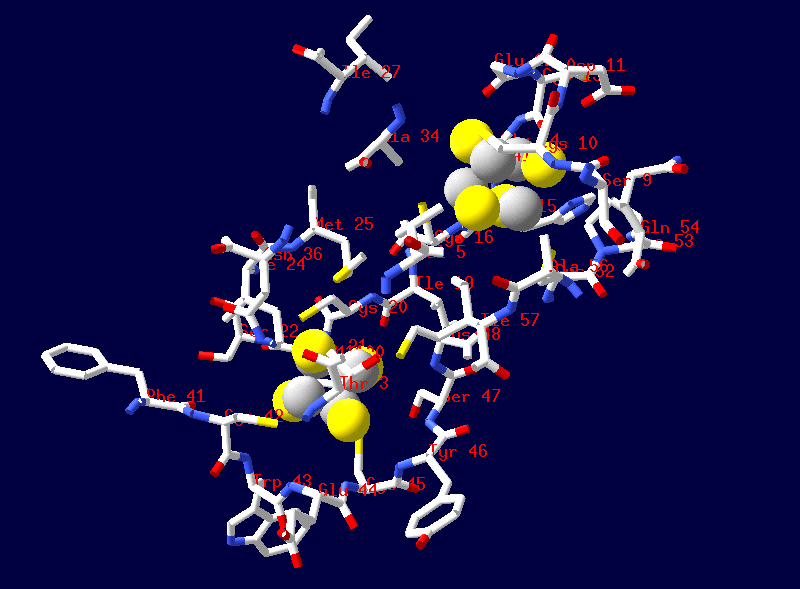

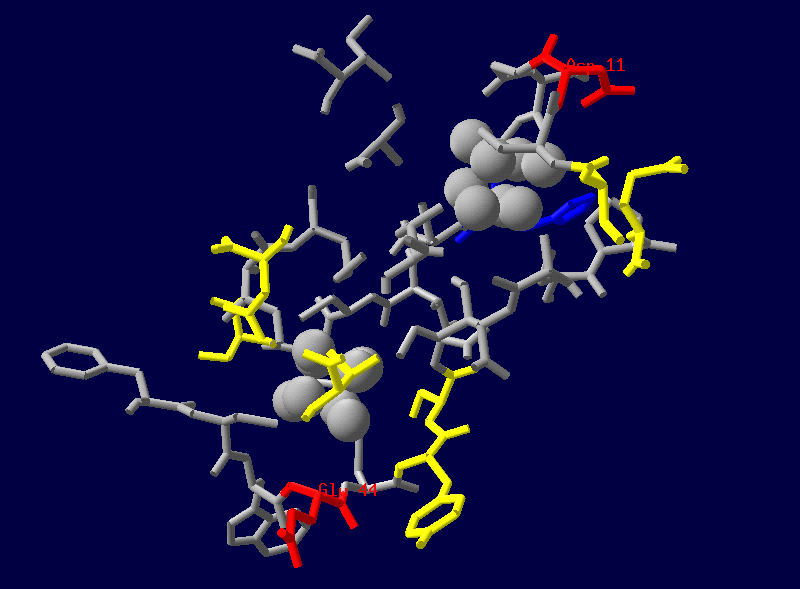

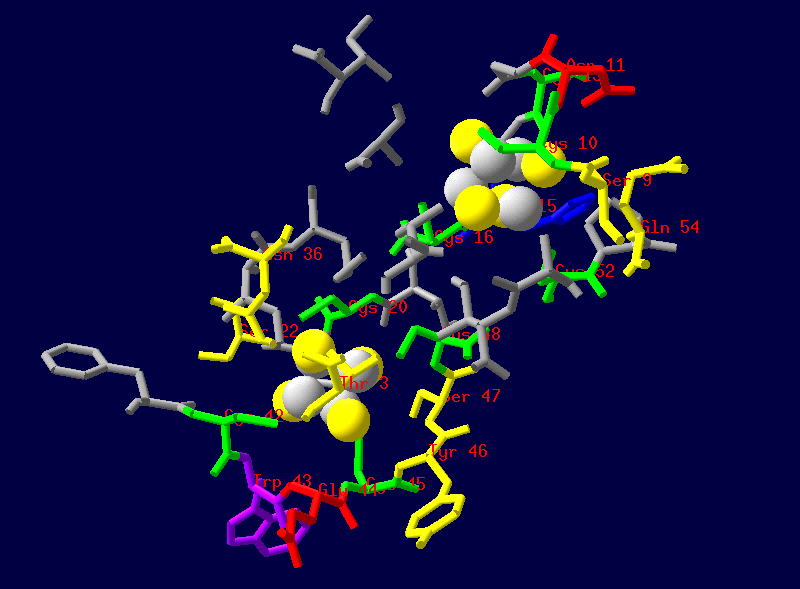
*

*Pelagibacter*

*ubique*

EBAC2C11

Crenarchaeal SRP Residues in a distance <5.0Å to the Charged and polar residues marked Cysteine and tryptophan marked

[4Fe-4S] clusters

*Pyrobaculum*

*calidifontis*

*
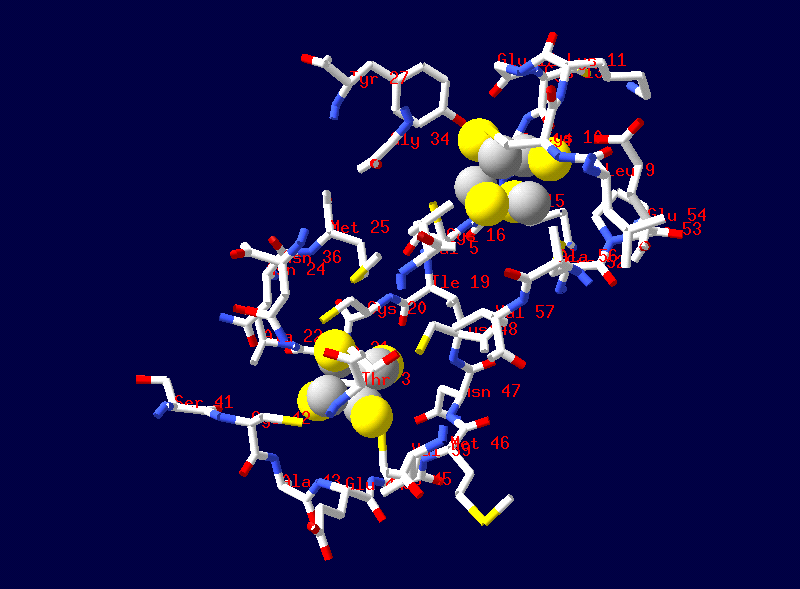
*

*
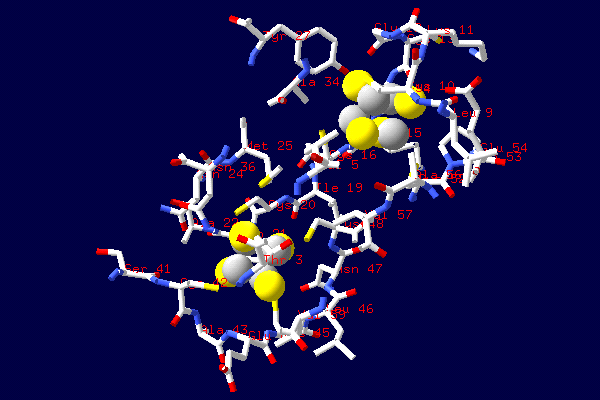

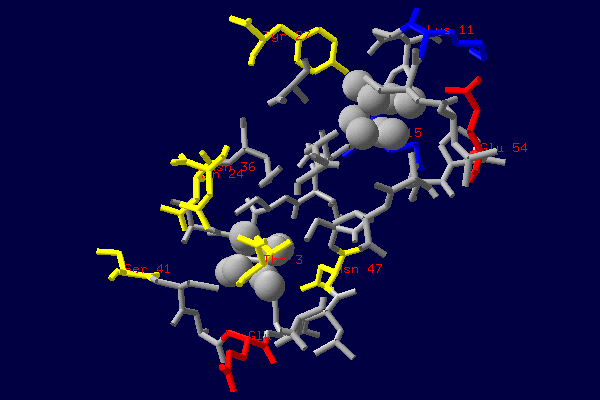

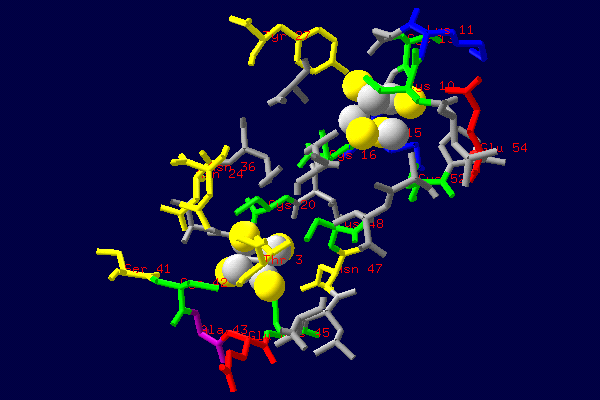

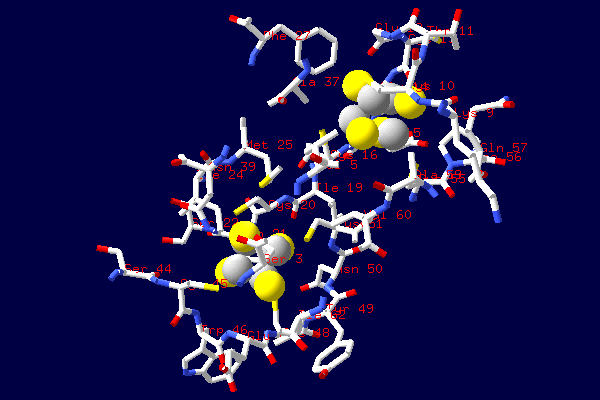

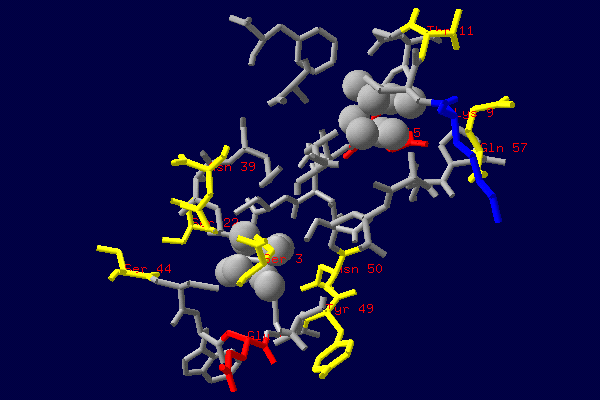

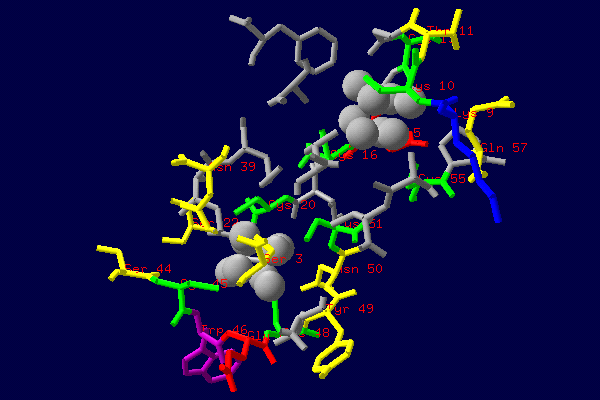

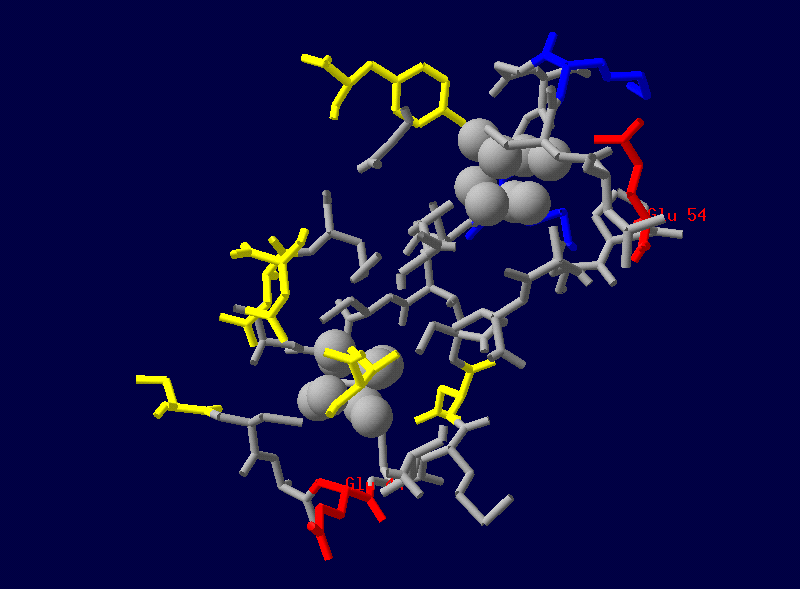

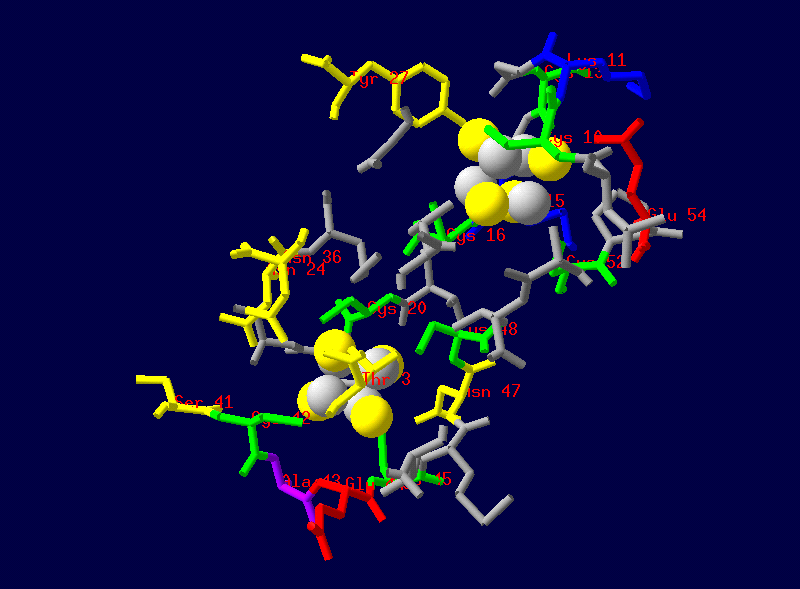
*

*Pyrobaculum*

*aerophilum*

*Caldivirga*

*maquilingensis*

SRB and related Residues in a distance <5.0Å to the Charged and polar residues marked Cysteine and tryptophan marked

SOB lineage II [4Fe-4S] clusters

*Desulfotomaculum*

*reducens*

*Syntrophobacter*

*fumaroxidans*

*
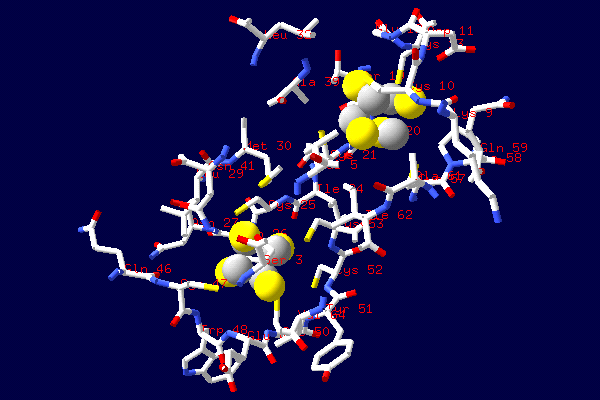

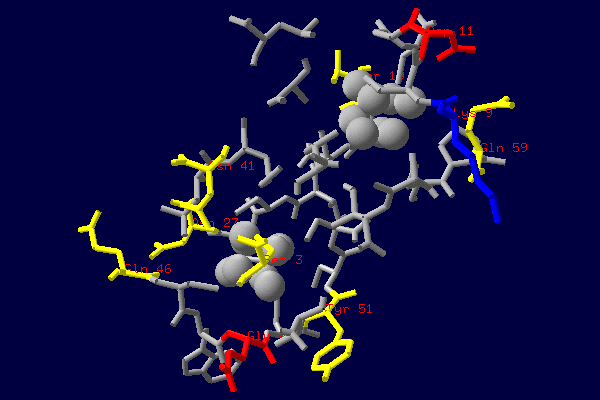

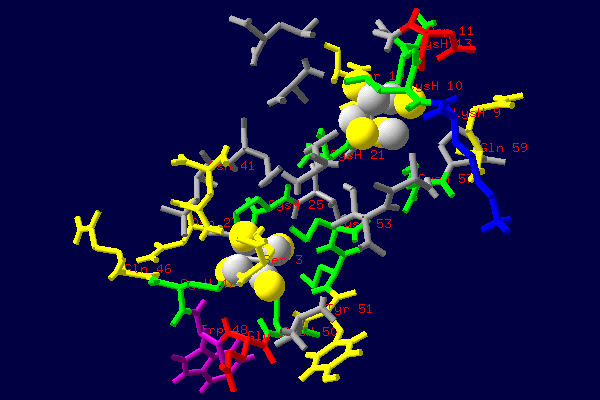

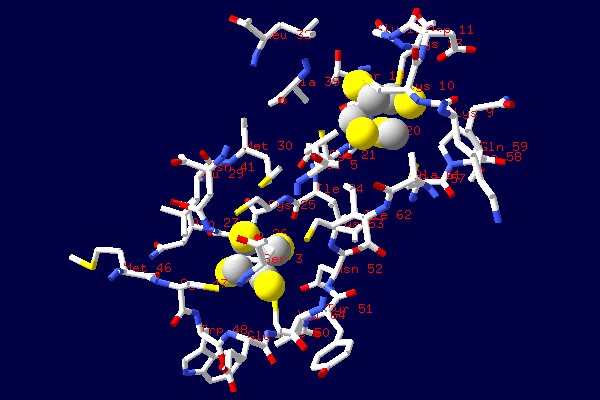

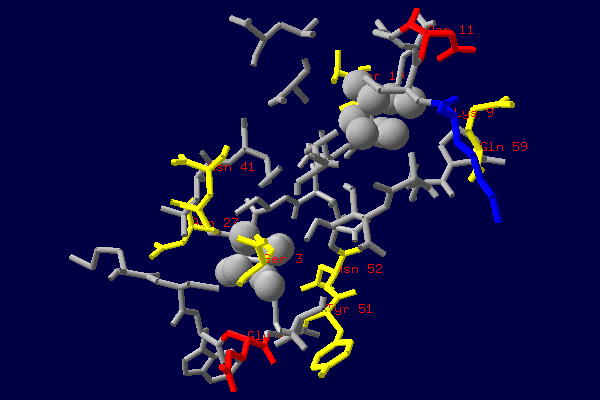

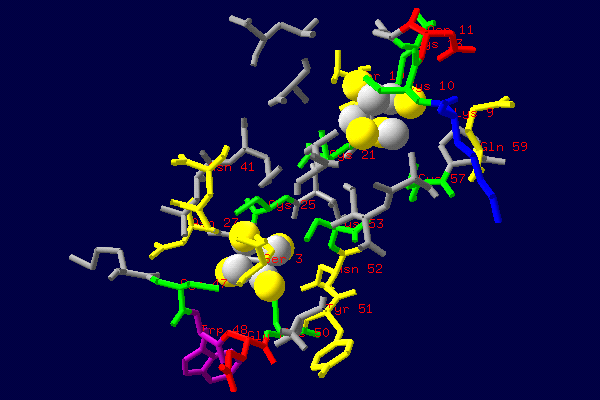

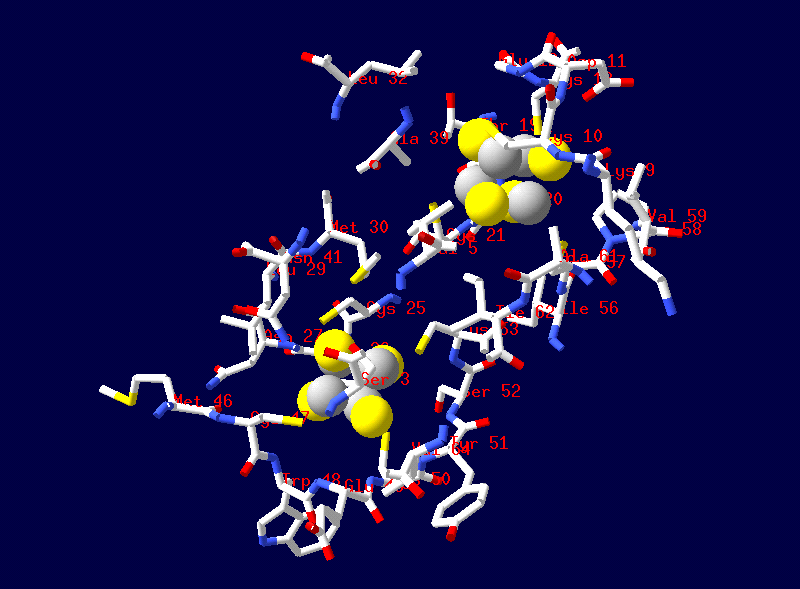

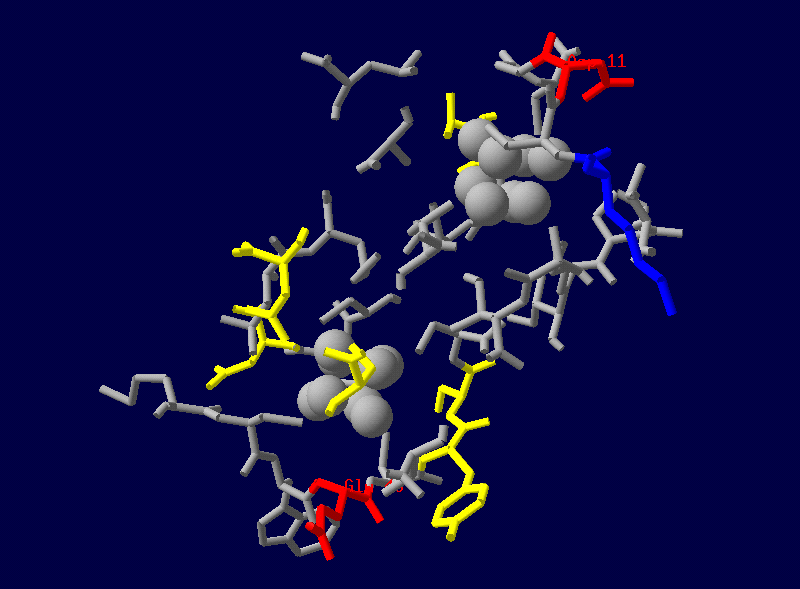

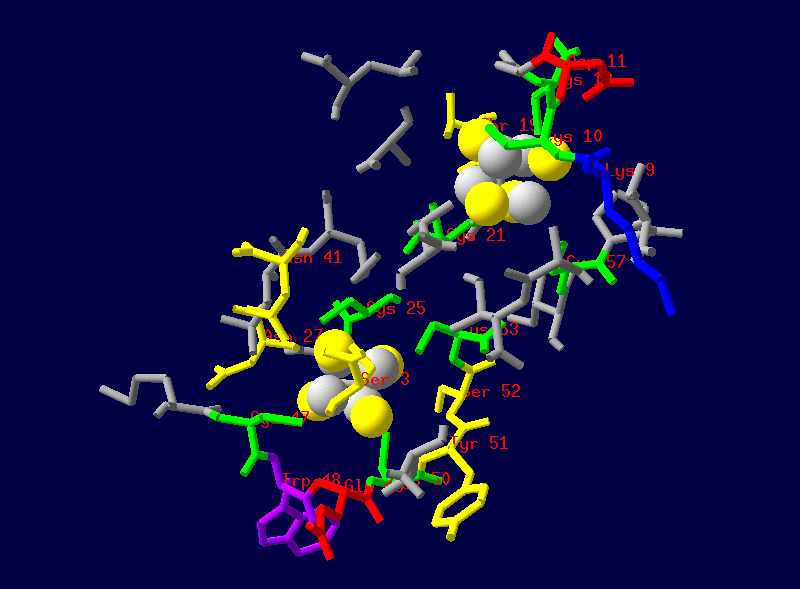
*

Fosws39f7

Residues in a distance <5.0Å to the Charged and polar residues marked Cysteine and tryptophan marked

[4Fe-4S] clusters


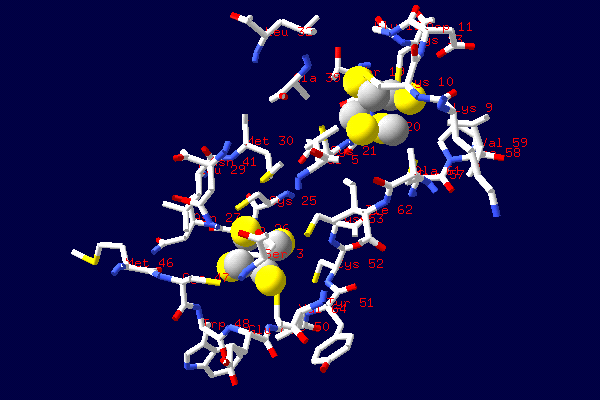

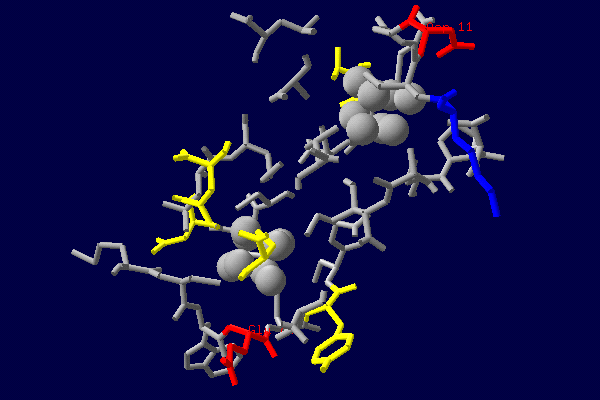

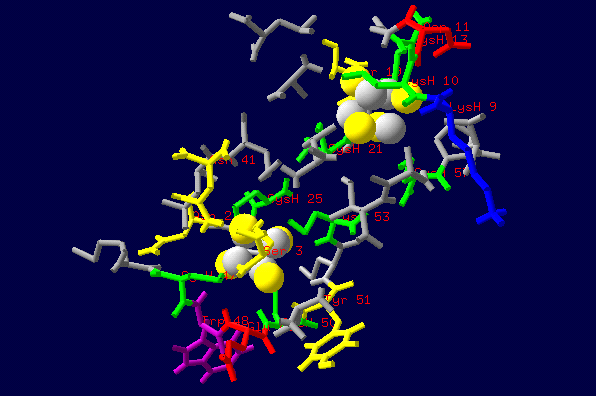
fosws7f8


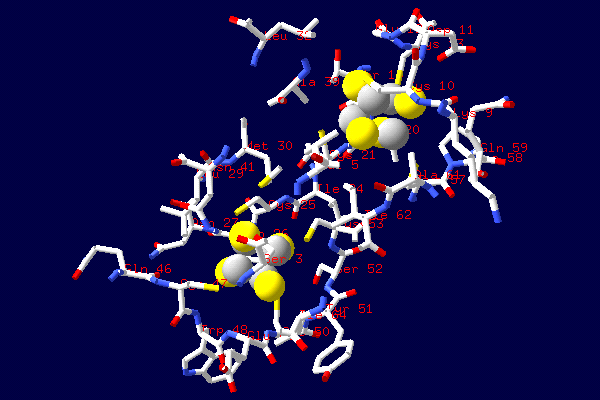

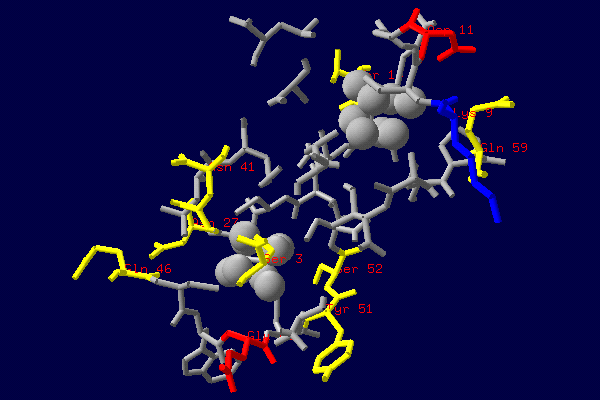

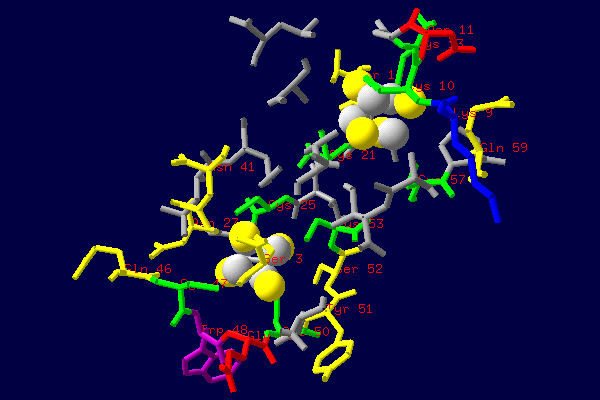
*Thermodesulfo-*

*bacterium*

*commune*

*
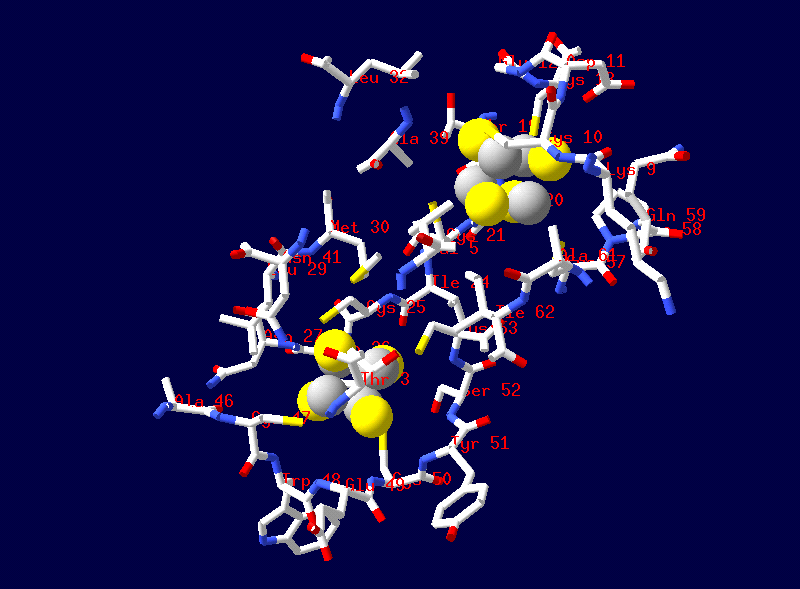

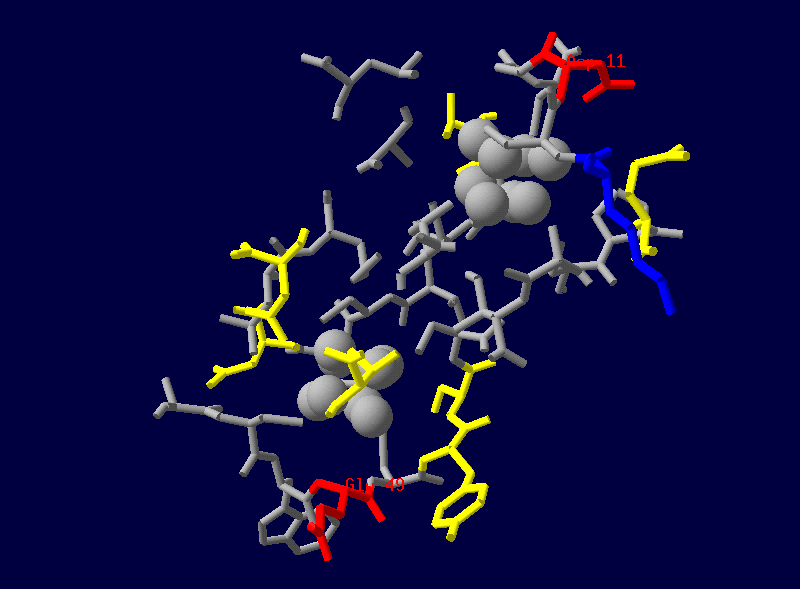

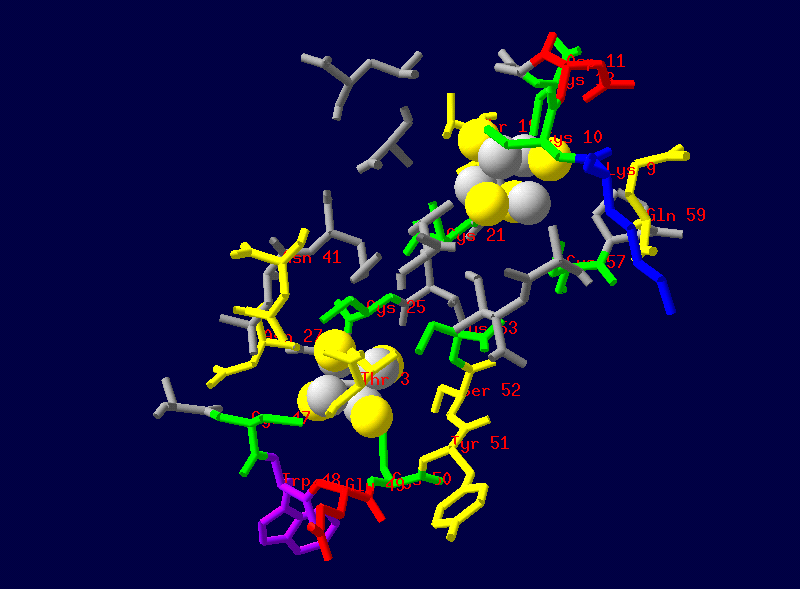
*

*Desulfovibrio*

*desulfuricans*

Residues in a distance <5.0Å to the Charged and polar residues marked Cysteine and tryptophan marked

[4Fe-4S] clusters

*
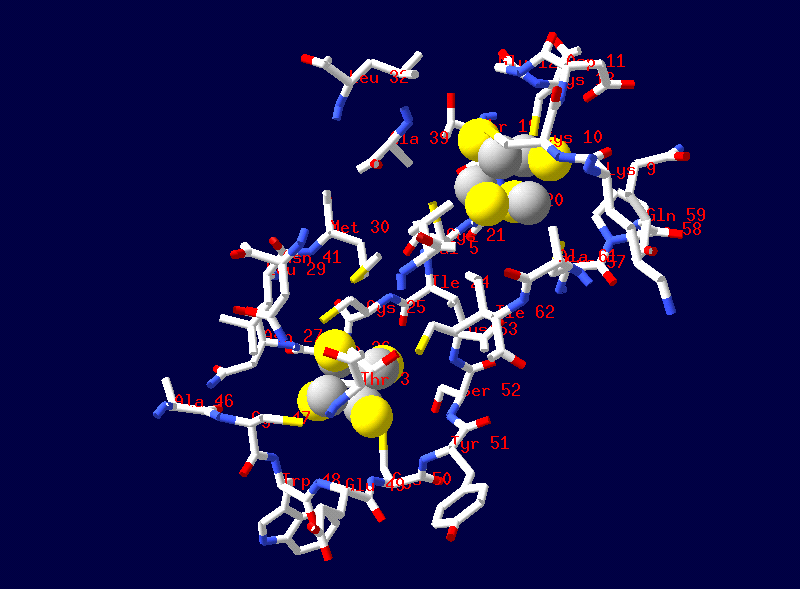

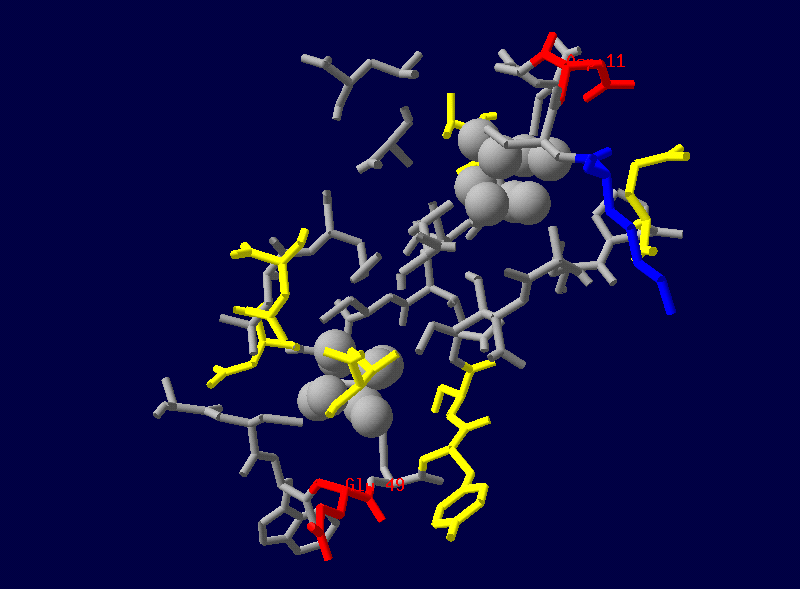

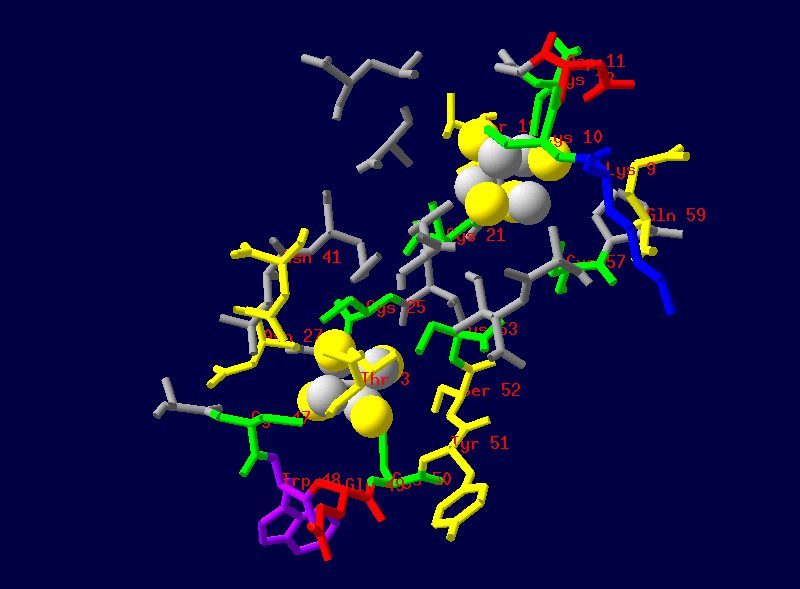
*

*Desulfovibrio*

*vulgaris*


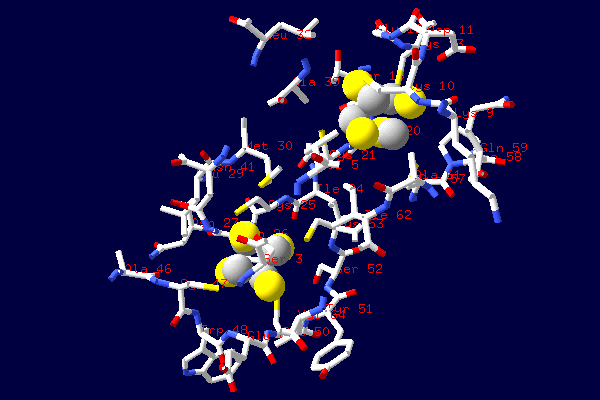

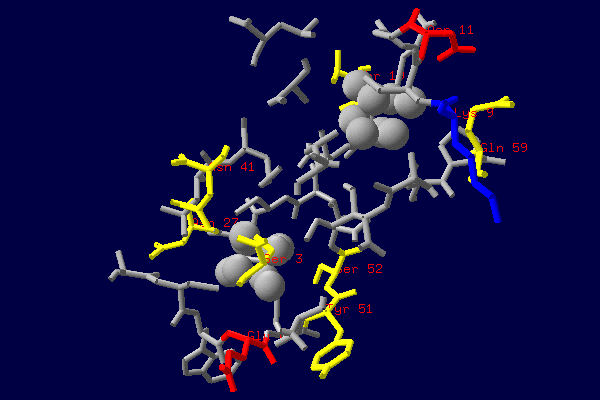

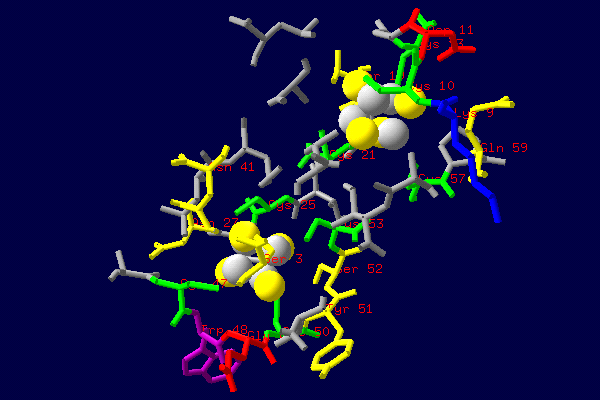


Desulfobulbus

sp.

*
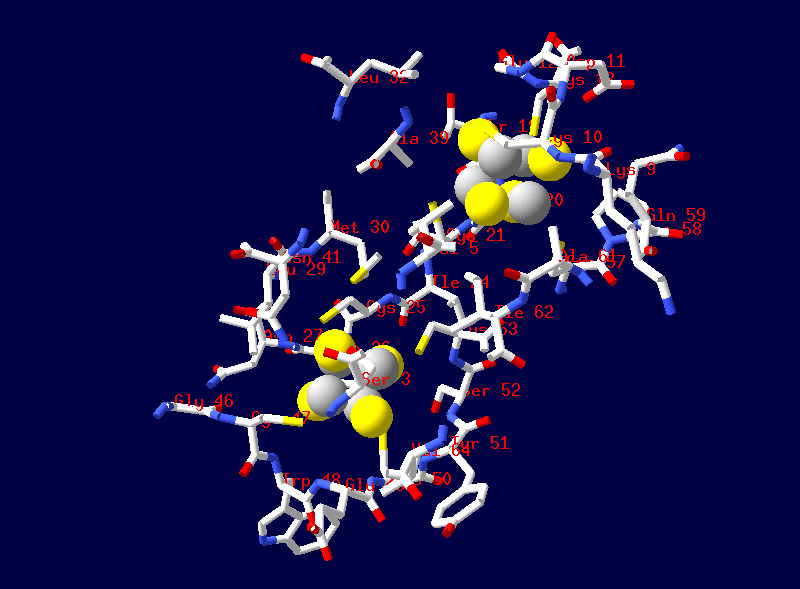

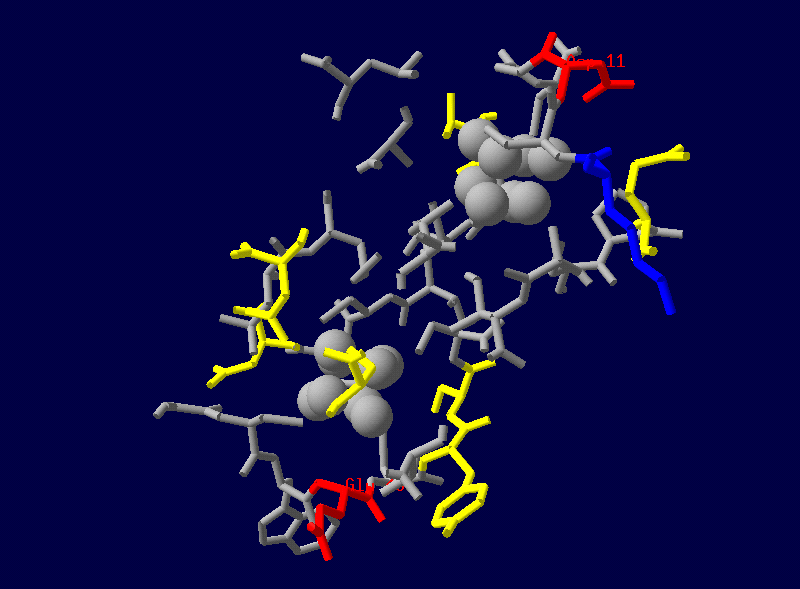

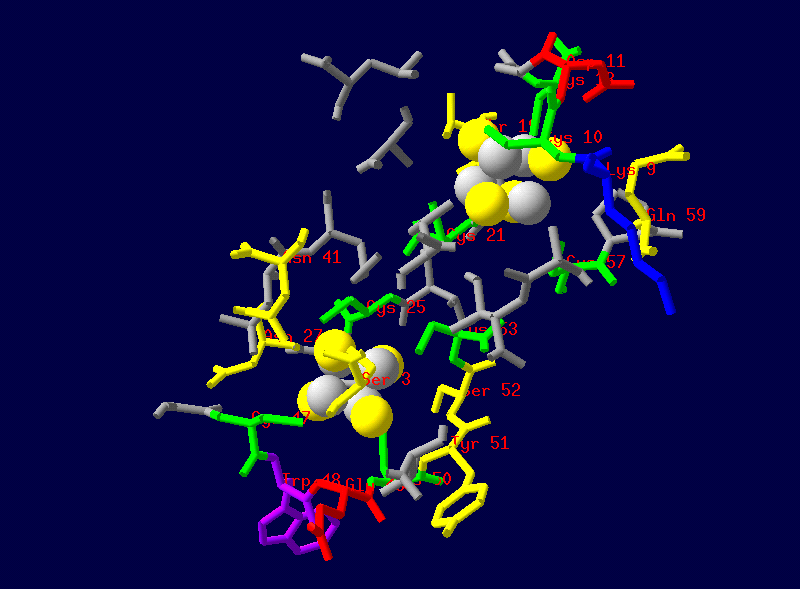
*

*Desulfotalea*

*psychrophila*

Residues in a distance <5.0Å to the Charged and polar residues marked Cysteine and tryptophan marked

[4Fe-4S] clusters

*
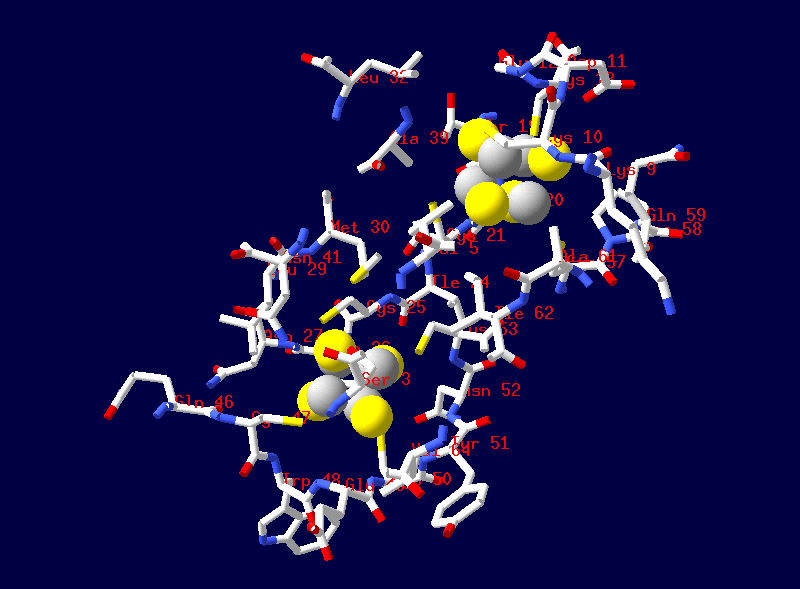

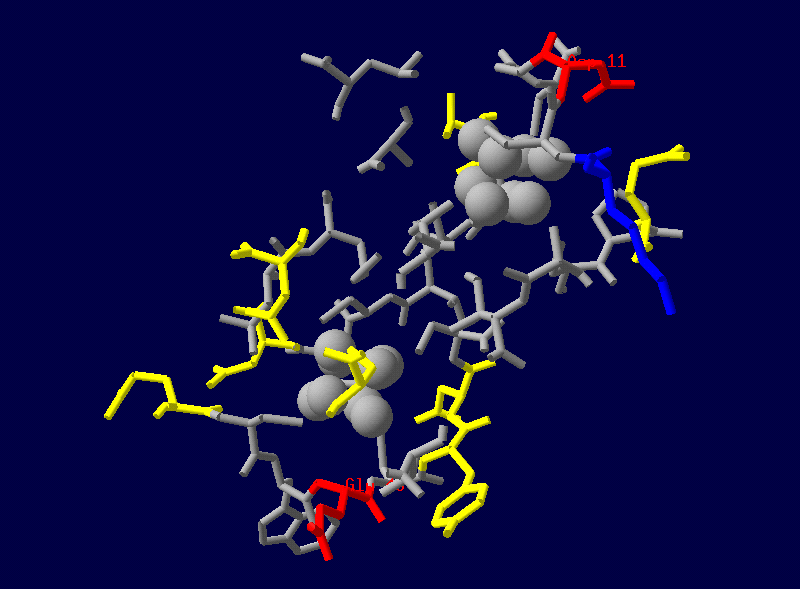

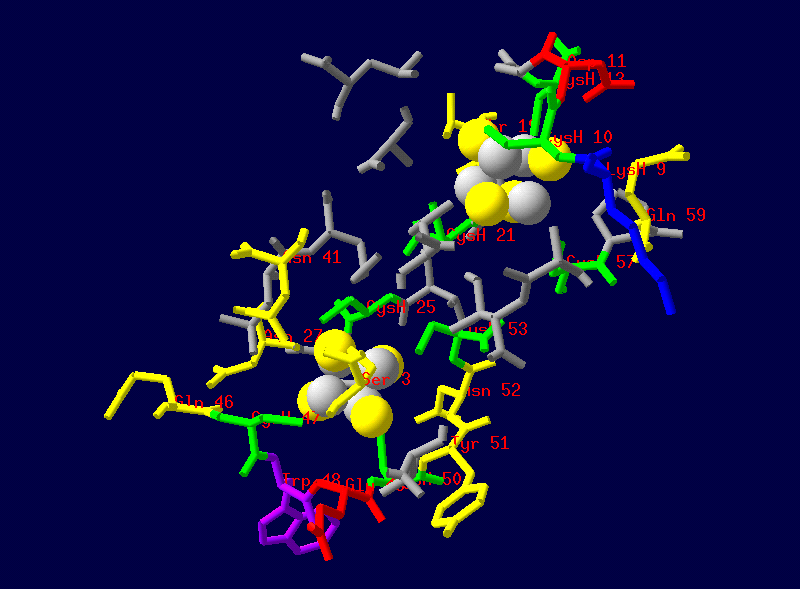

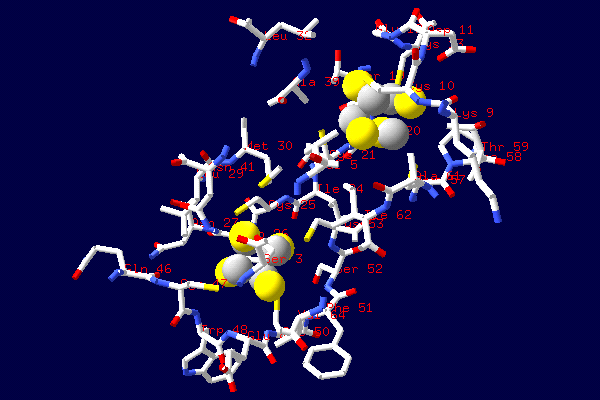

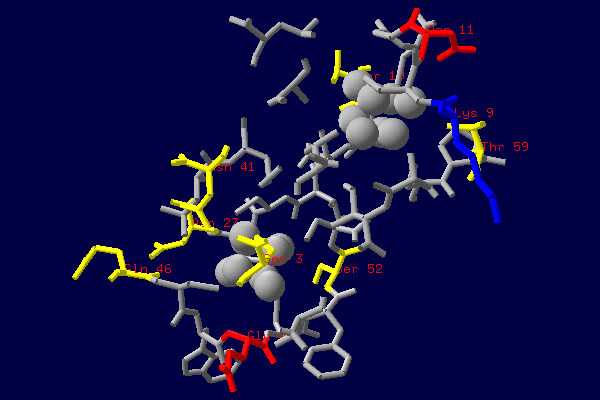

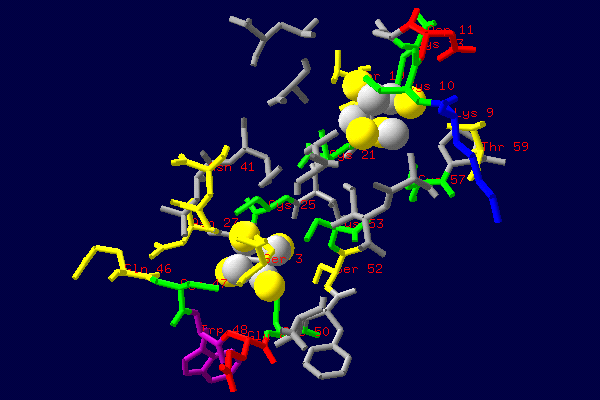

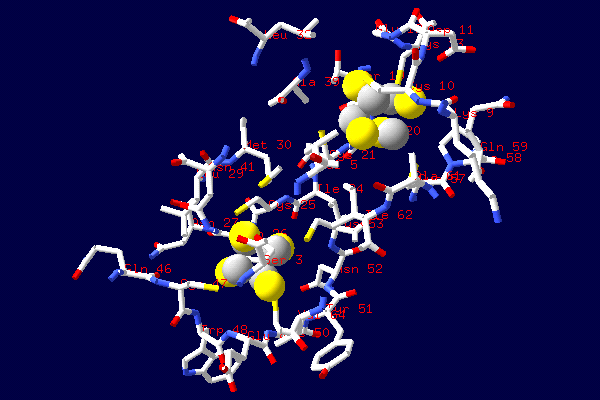
*

O. algarvensis

Delta 1

symbiont

*
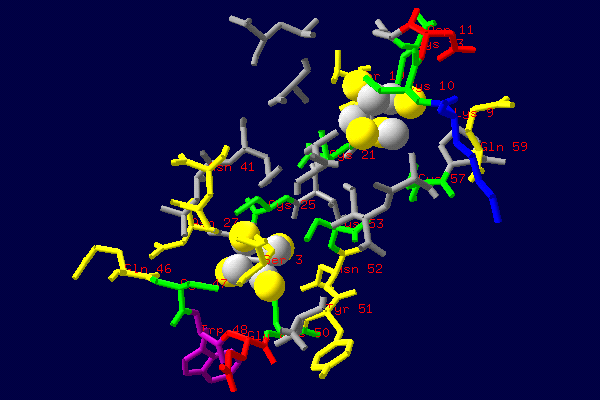
*


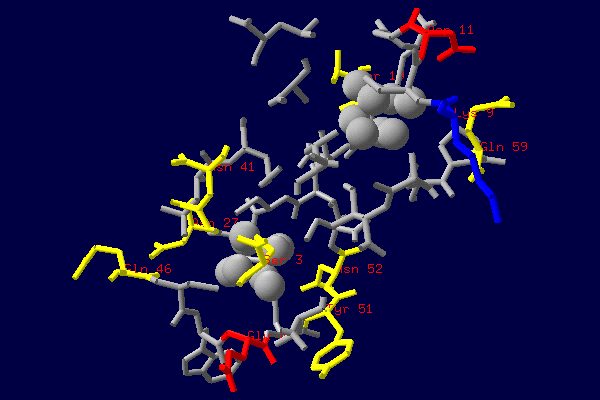
*Thermodesulfo-*

*vibrio*

*yellowstonii*

*Chlorobaculum*

*tepidum*

Residues in a distance <5.0Å to the Charged and polar residues marked Cysteine and tryptophan marked

[4Fe-4S] clusters

*Thiobacillus*

*denitrificans*

*
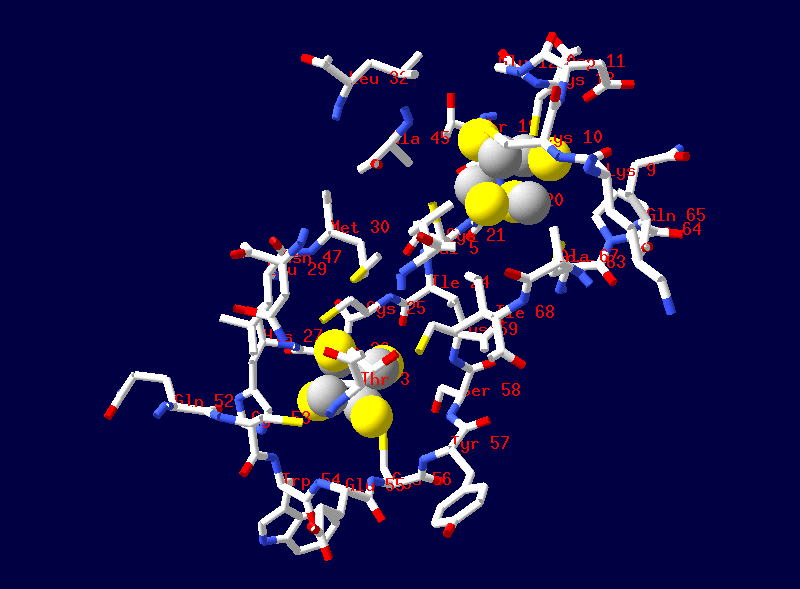

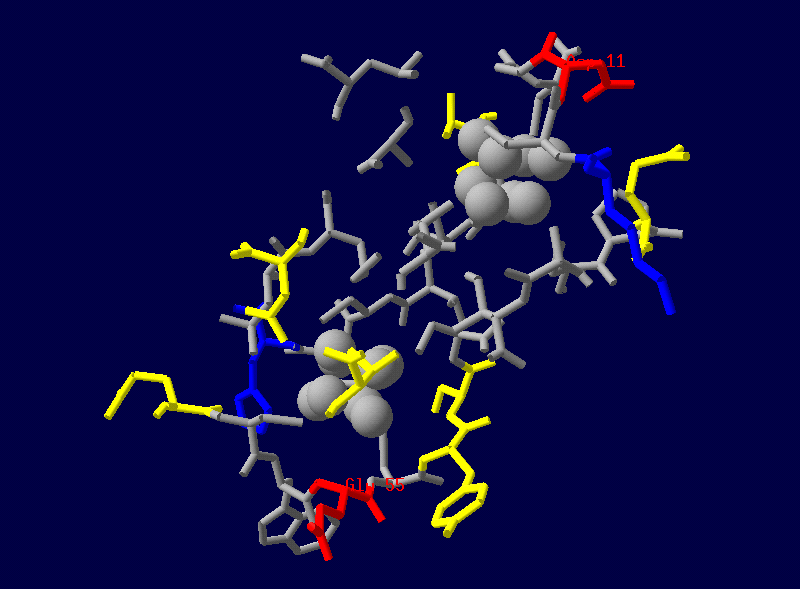

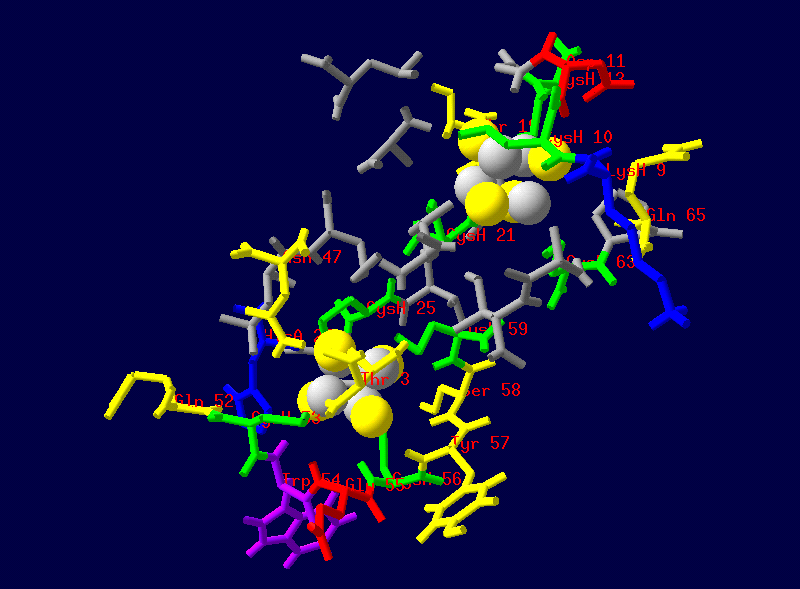
*
