# Supplementary material for: Homology Modeling of Dissimilatory APS Reductases (AprBA) of Sulfur-Oxidizing and Sulfate-Reducing Prokaryotes
Source: PLoS One. 2008 Jan 30;3(1):e1514. doi: 10.1371/journal.pone.0001514 (PMC2211403; doi:10.1371/journal.pone.0001514)
Supplement: Figure S3 — (8.75 MB DOC) [file pone.0001514.s003.doc]

**Supplementary data material Figure S3.** **AprA comparative models of SRP and SOB**

Reference structure


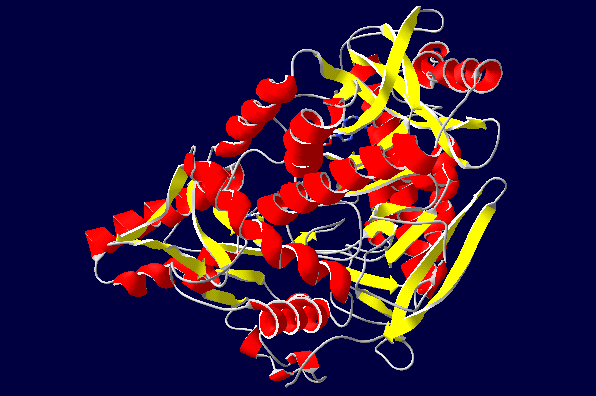
*Archaeoglobus fulgidus*

# B

3D ribbon structure colored by secondary structure elements


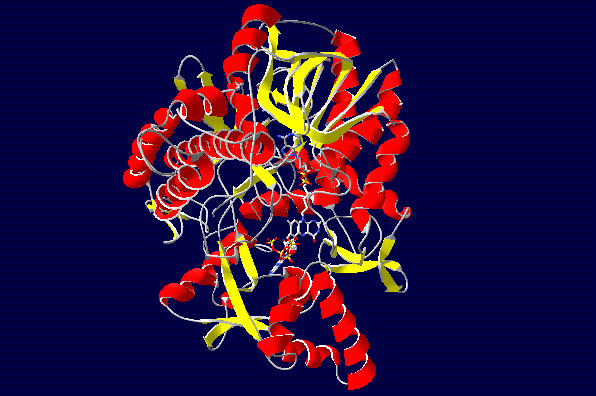

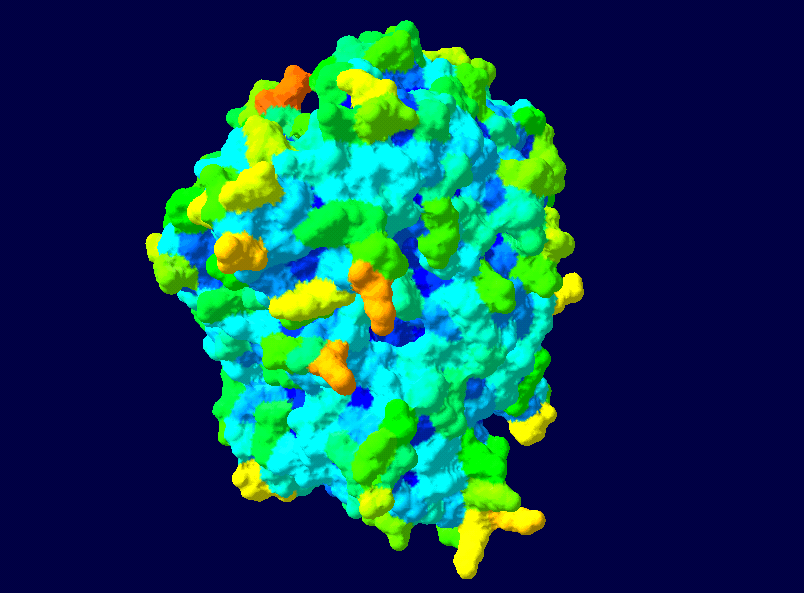
 AprA model shown from front side

# C

# D


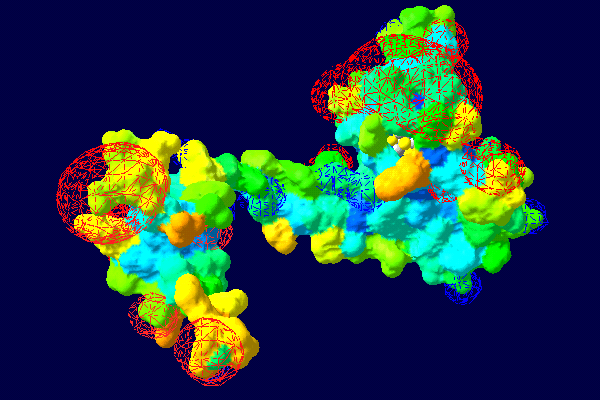

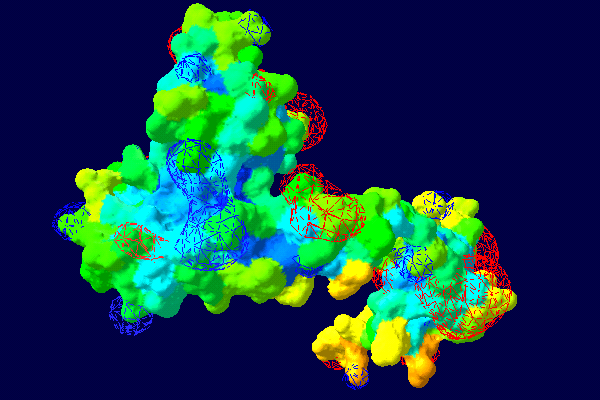
3D ribbon structure colored by model confidence Protein molecular surface colored by accessibility


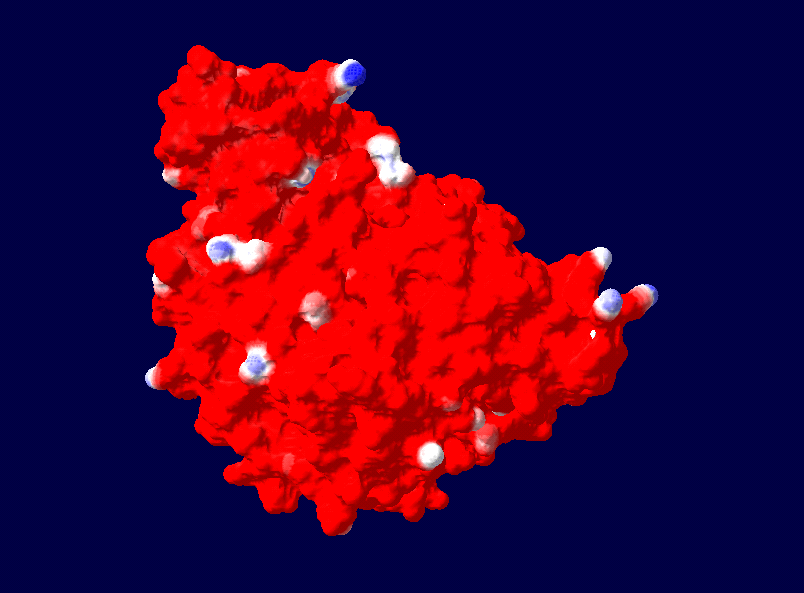

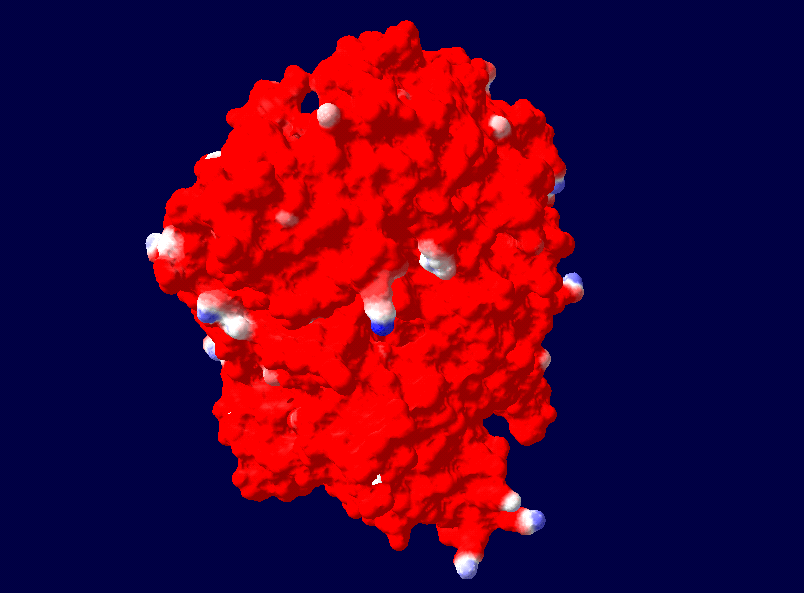
AprA model shown from top view
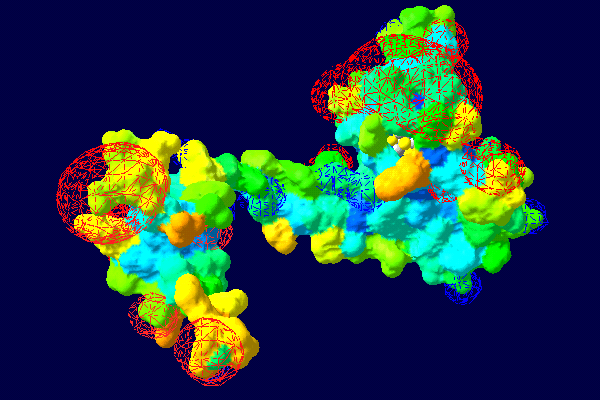

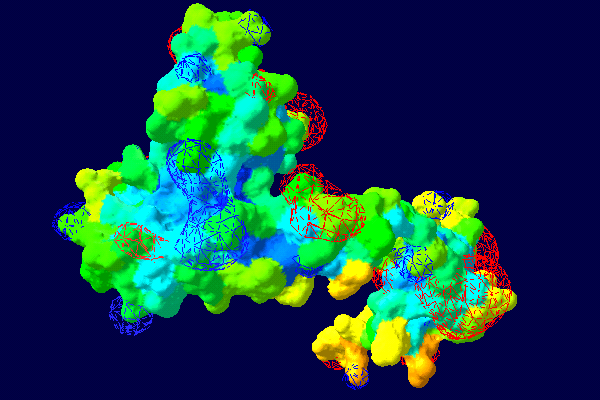


# E

# F

AprA model shown from top view AprA model shown from back side


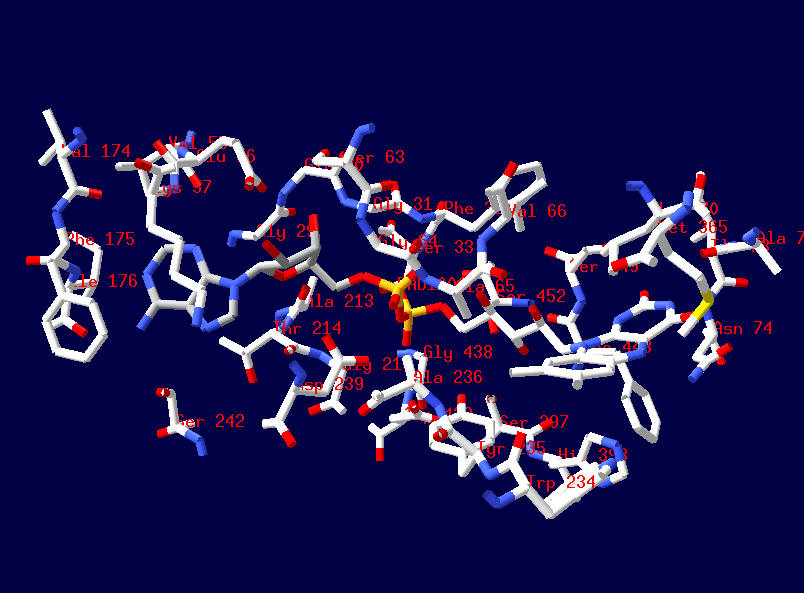

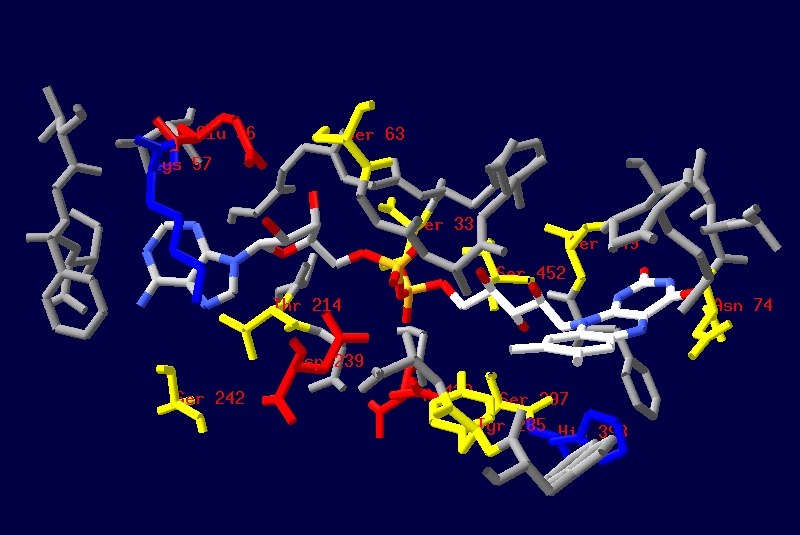
Protein molecular surface colored by calculated electrostatic potential (electric charge at the molecular surface is colored with a red (negative), white (neutral, and blue (positive) color gradient)

# G

# H

# G

# H

FAD surrounding protein matrix: residues present in a distance of < 4.1Å are shown (amino acids are coloured as follows: positively charged, basic AA, blue; negatively charged, acidic AA, red; polar AA, yellow; and unpolar, uncharged AA, grey

**D**

**H**

###### SOB Apr lineage I


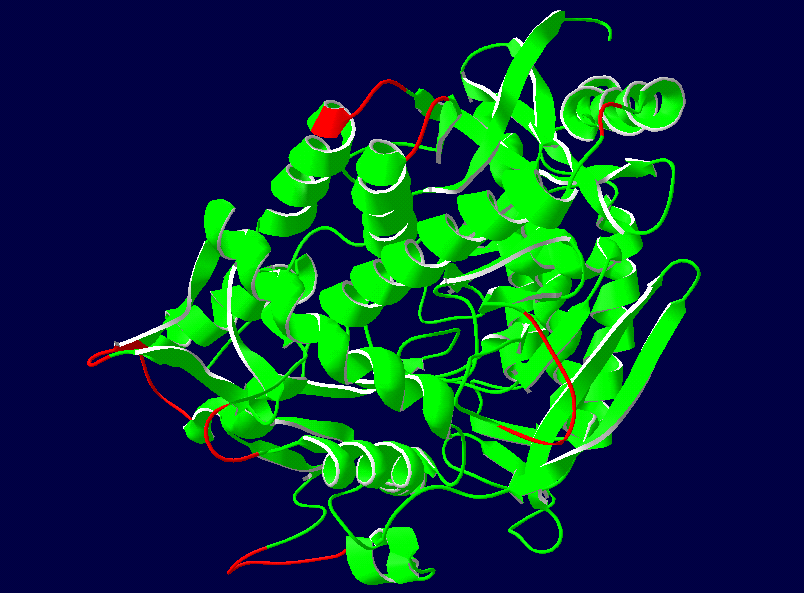

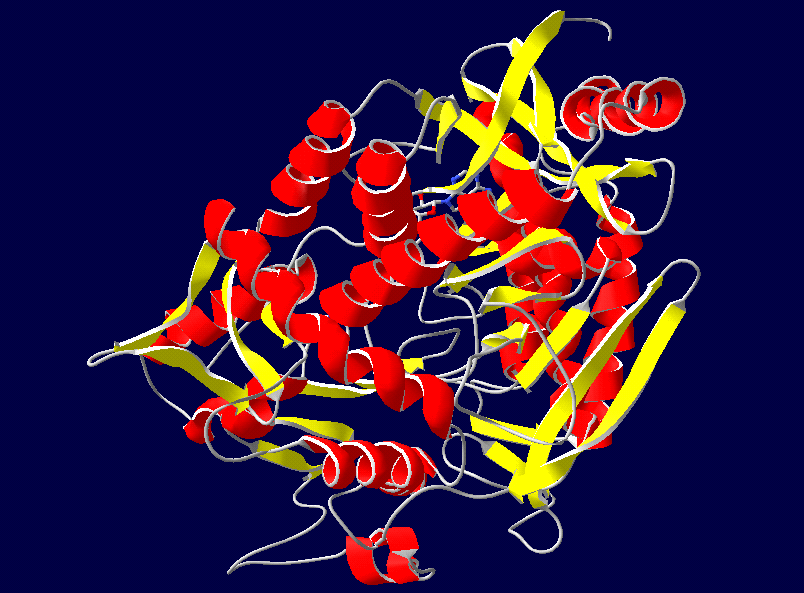
Allochromatium vinosum

# A

# B

3D ribbon structure colored by model confidence 3D ribbon structure colored by secondary structure elements


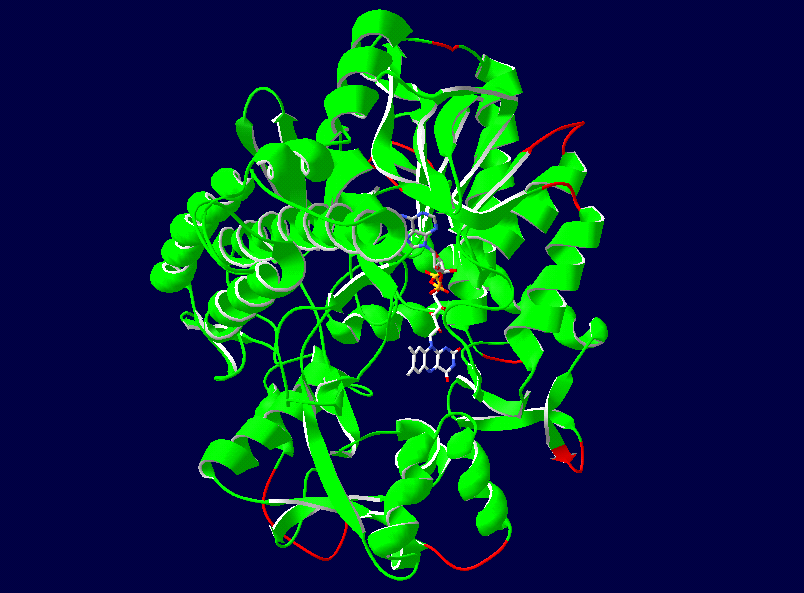

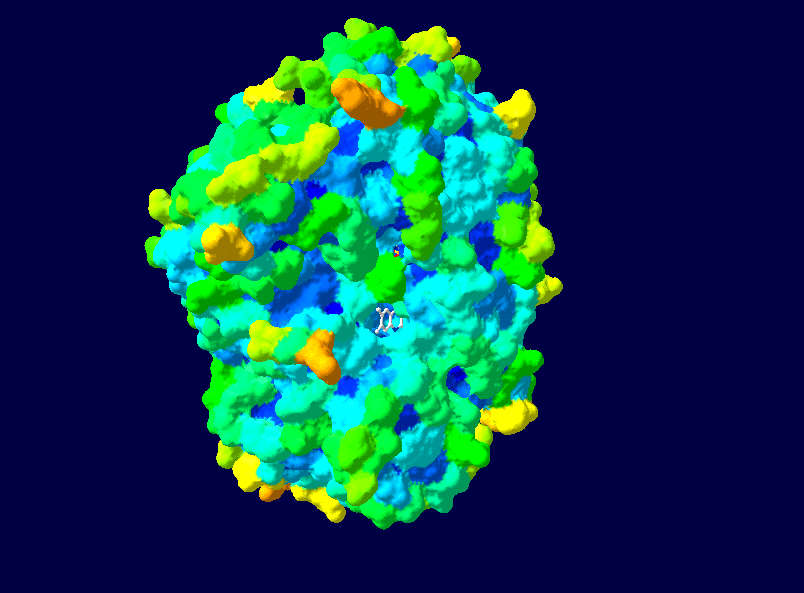
 AprA model shown from front side

# C

# D


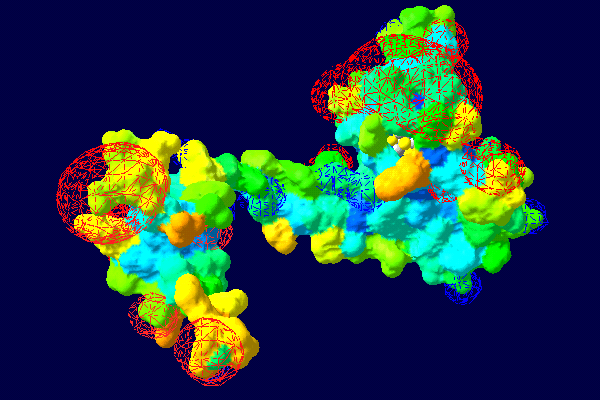

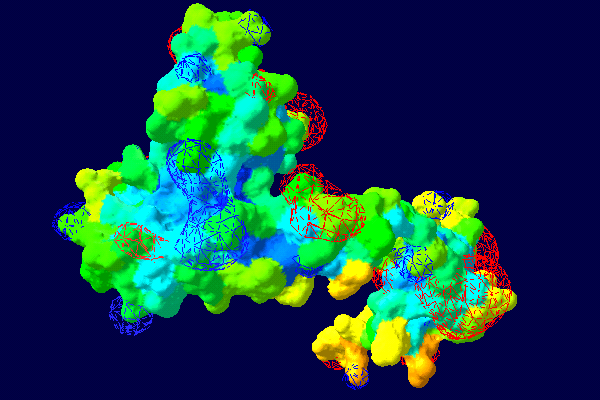
3D ribbon structure colored by model confidence Protein molecular surface colored by accessibility


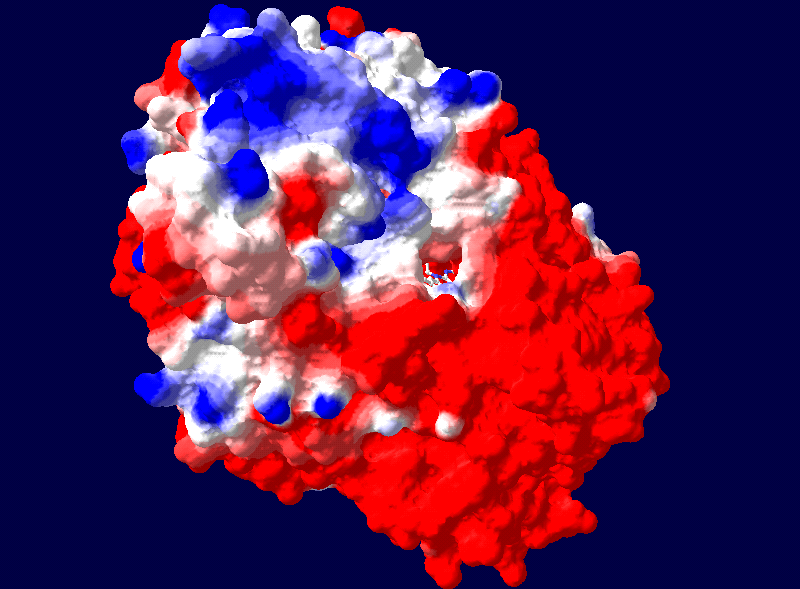

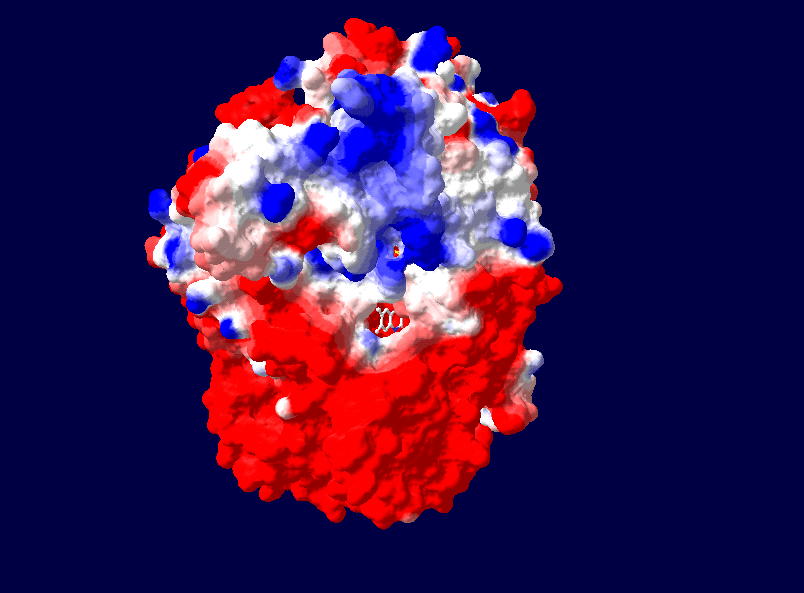
AprA model shown from top view
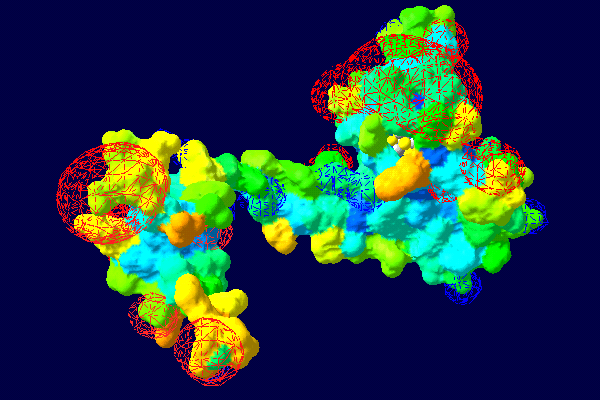

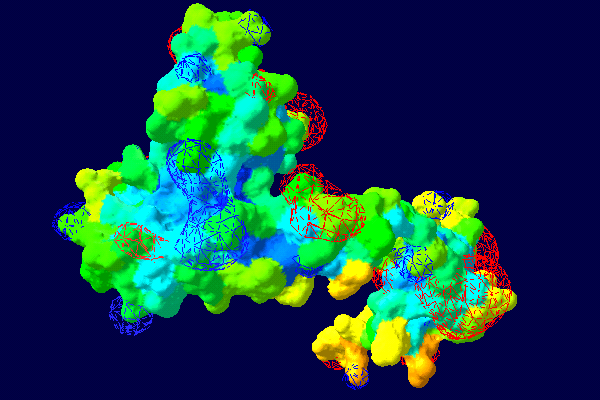


# E

# F

AprA model shown from top view AprA model shown from back side

Protein molecular surface colored by calculated electrostatic potential (electric charge at the molecular surface is colored with a red (negative), white (neutral, and blue (positive) color gradient)


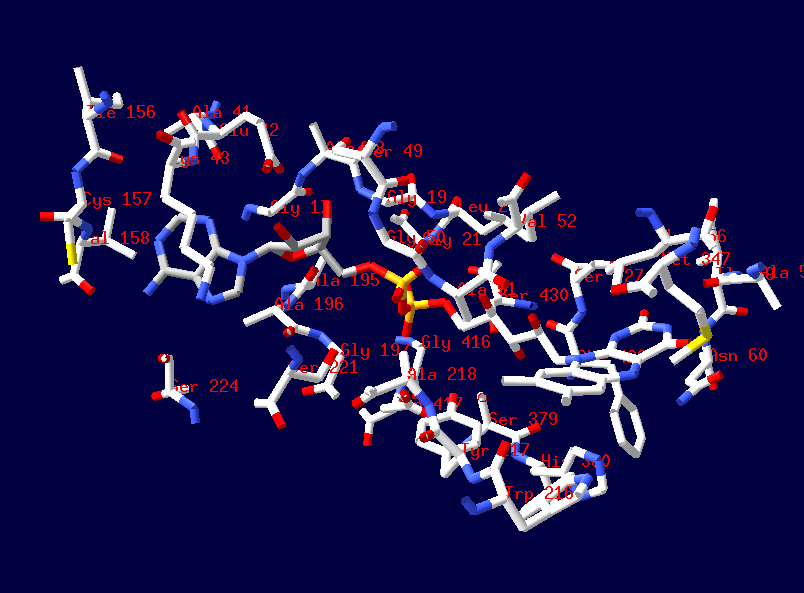

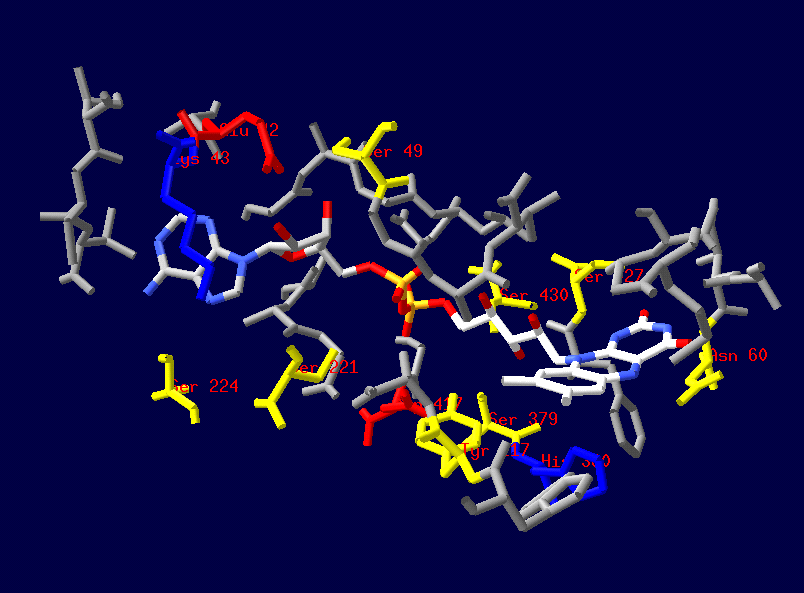


# G

# H

FAD surrounding protein matrix: residues present in a distance of < 4.1Å are shown (amino acids are coloured as follows: positively charged, basic AA, blue; negatively charged, acidic AA, red; polar AA, yellow; and unpolar, uncharged AA, grey)


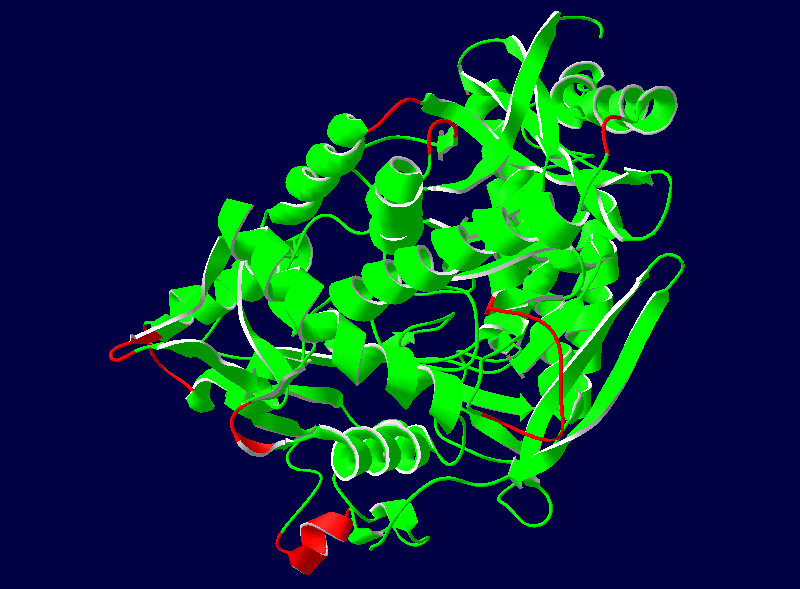

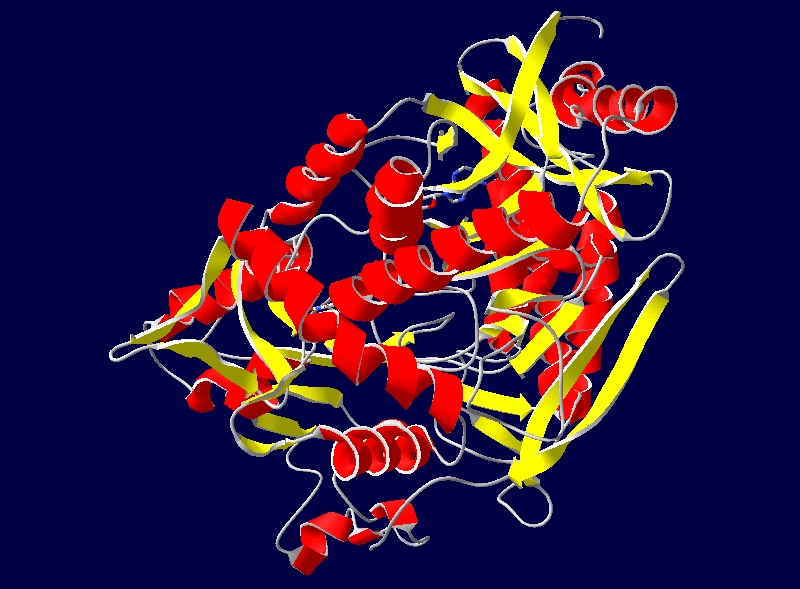
Thiobacillus denitrificans

# A

# B

3D ribbon structure colored by model confidence 3D ribbon structure colored by secondary structure elements

AprA model shown from front side


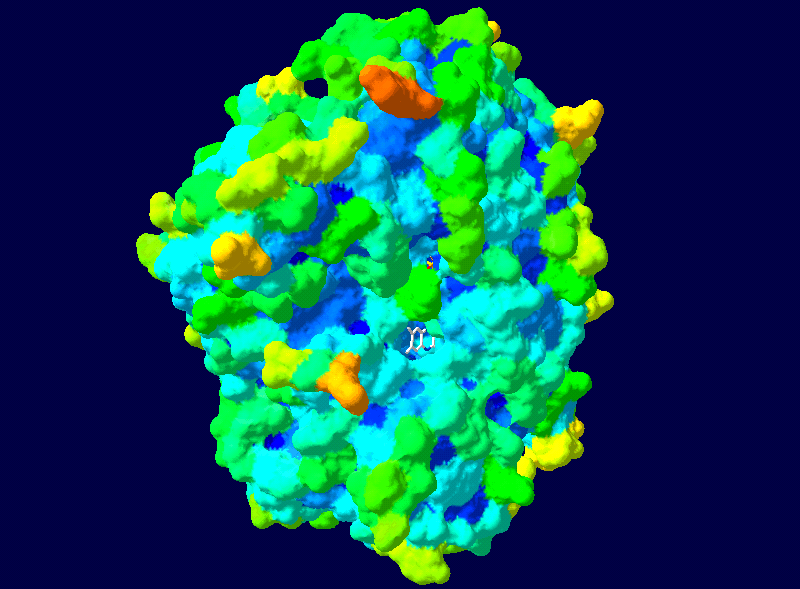

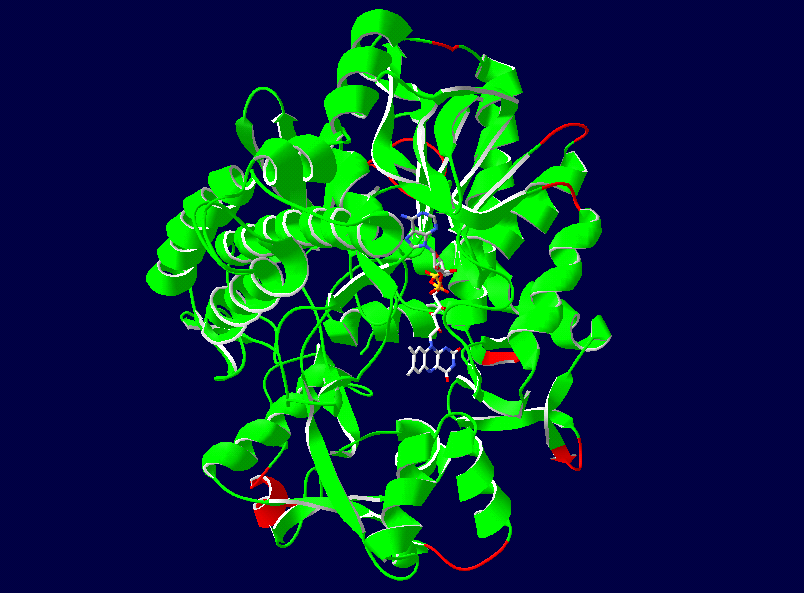


# C

# D


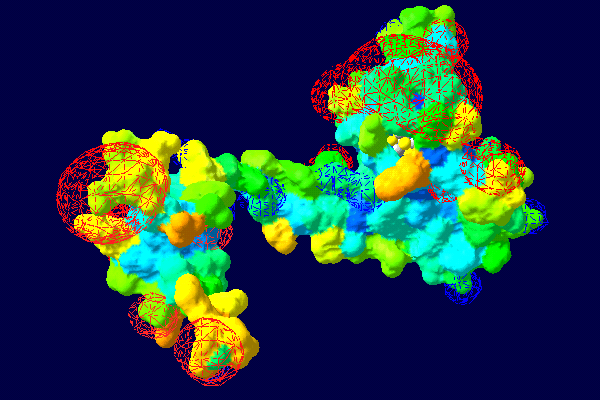

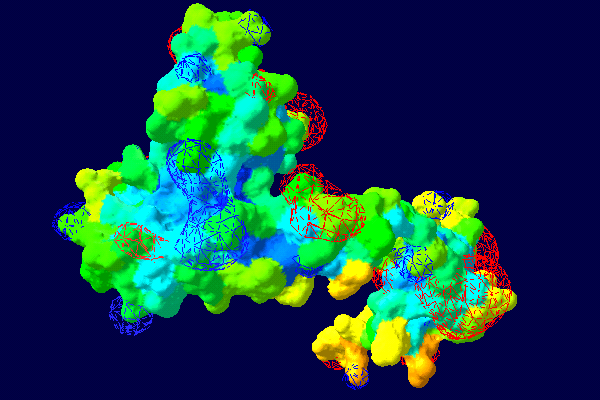
3D ribbon structure colored by model confidence Protein molecular surface colored by accessibility

AprA model shown from top view


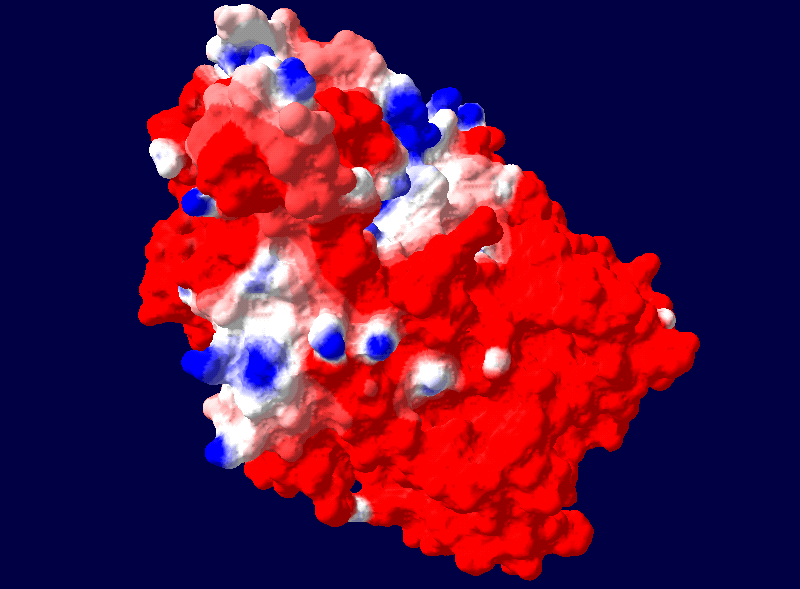

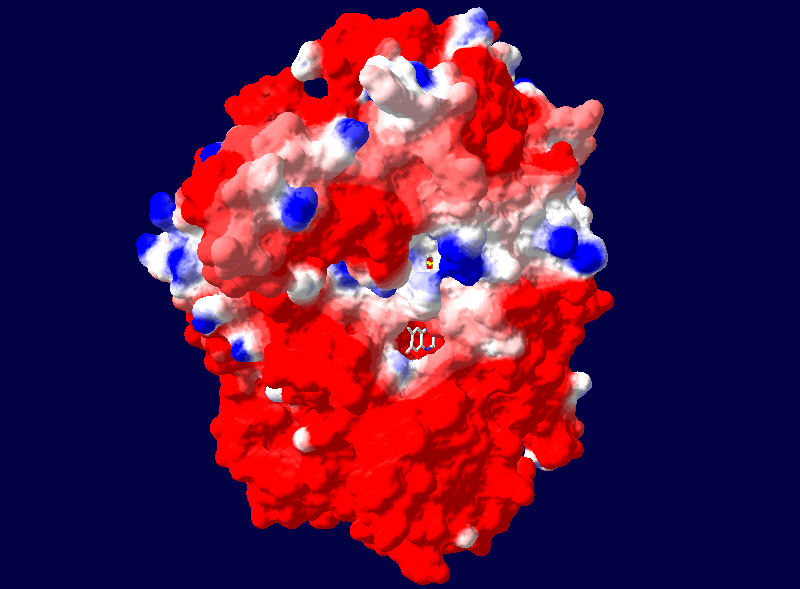

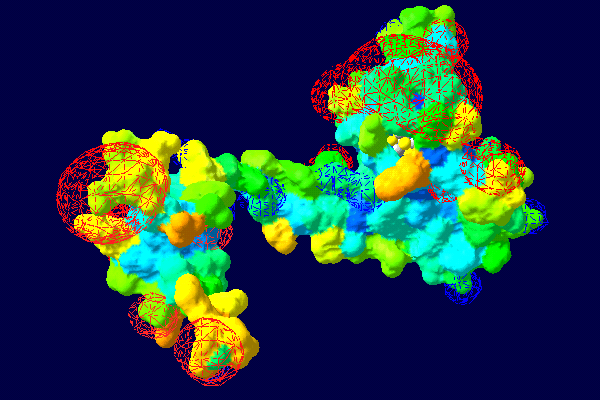

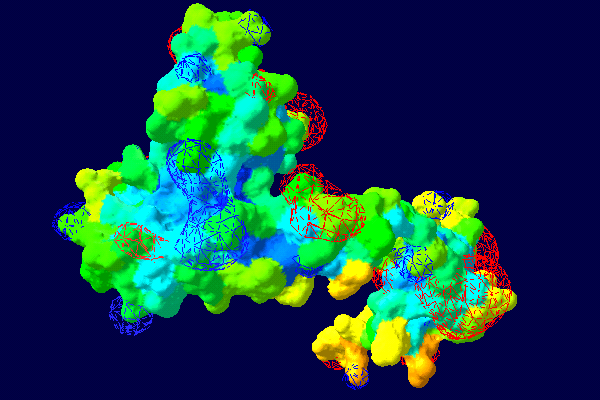


# E

# F

AprA model shown from top view AprA model shown from back side

Protein molecular surface colored by calculated electrostatic potential (electric charge at the molecular surface is colored with a red (negative), white (neutral, and blue (positive) color gradient)


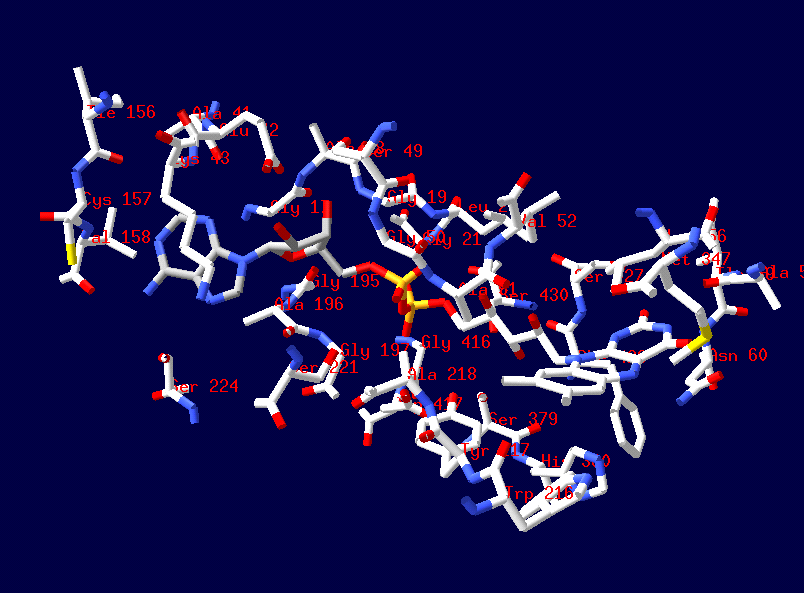

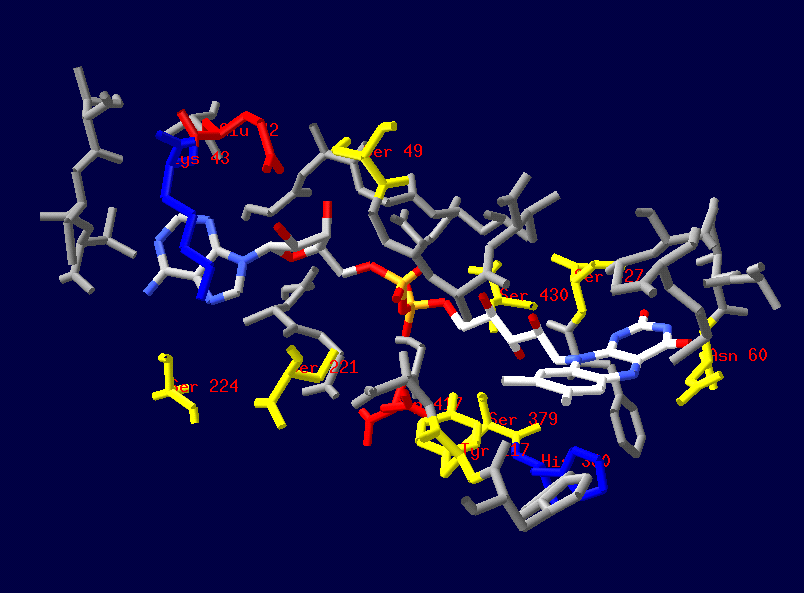


# G

# H

FAD surrounding protein matrix: residues present in a distance of < 4.1Å are shown (amino acids are coloured as follows: positively charged, basic AA, blue; negatively charged, acidic AA, red; polar AA, yellow; and unpolar, uncharged AA, grey)


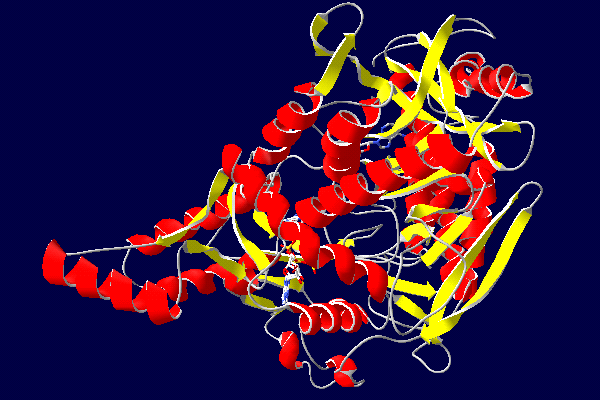

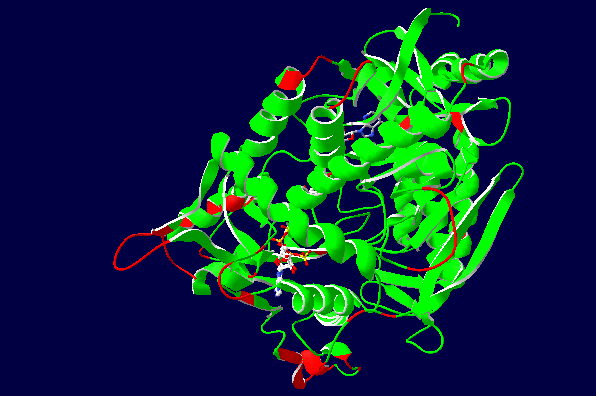
Cdt. Ruthia magnifica

# A

# B

3D ribbon structure colored by model confidence 3D ribbon structure colored by secondary structure elements

AprA model shown from front side


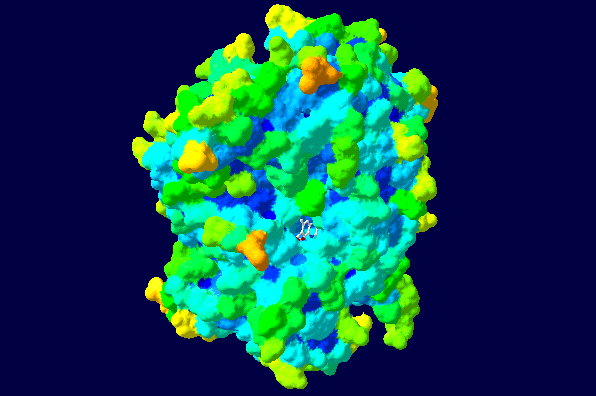

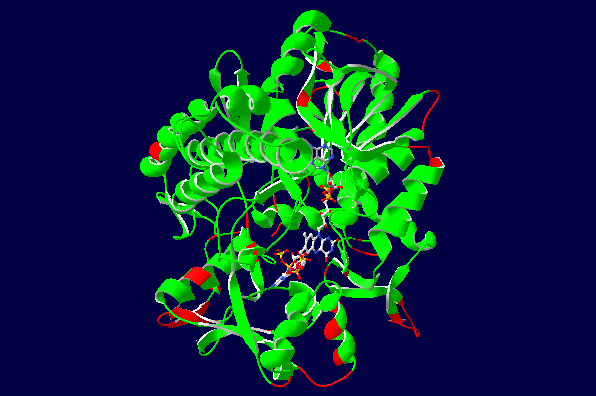


# C

# D

3D ribbon structure colored by model confidence Protein molecular surface colored by accessibility

AprA model shown from top view


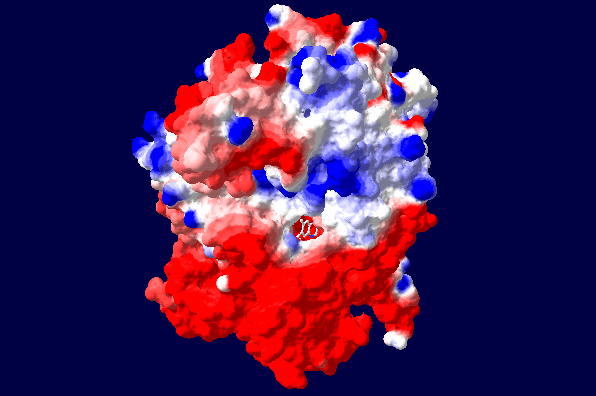

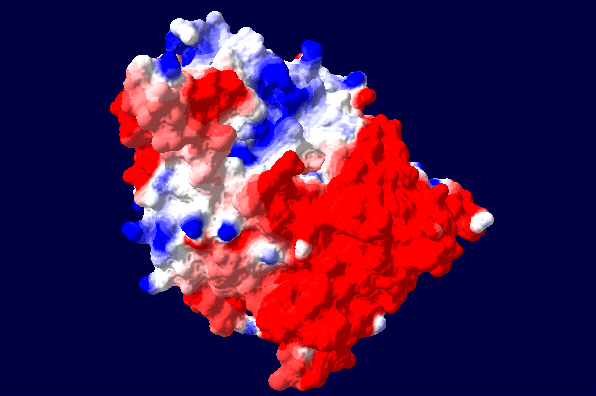

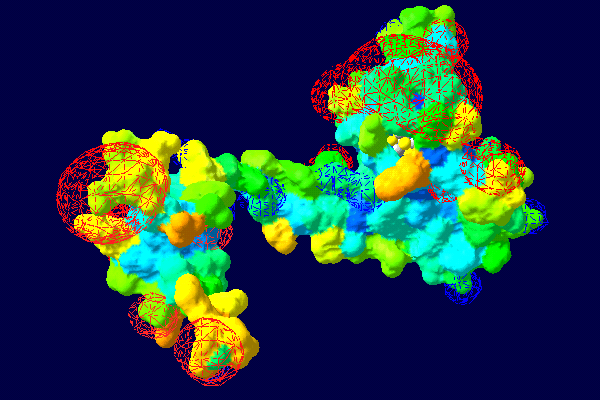

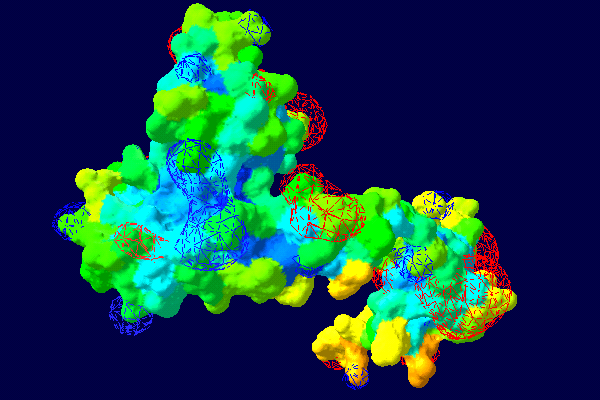


# E

# F

AprA model shown from top view AprA model shown from back side

Protein molecular surface colored by calculated electrostatic potential (electric charge at the molecular surface is colored with a red (negative), white (neutral, and blue (positive) color gradient)


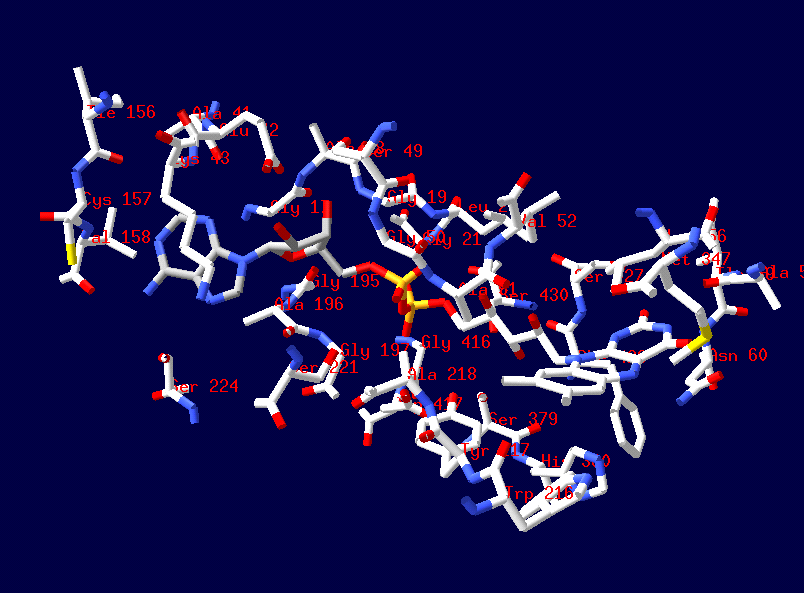

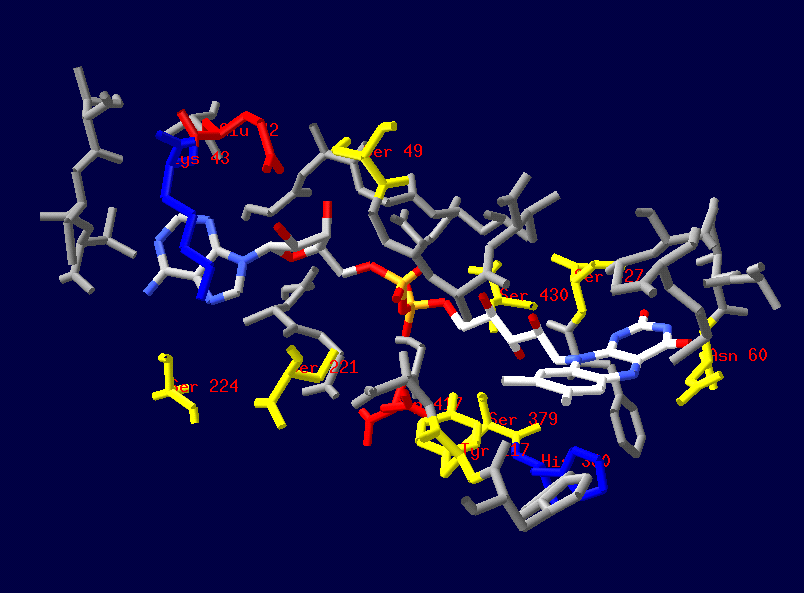


# G

# H

FAD surrounding protein matrix: residues present in a distance of < 4.1Å are shown (amino acids are coloured as follows: positively charged, basic AA, blue; negatively charged, acidic AA, red; polar AA, yellow; and unpolar, uncharged AA, grey)


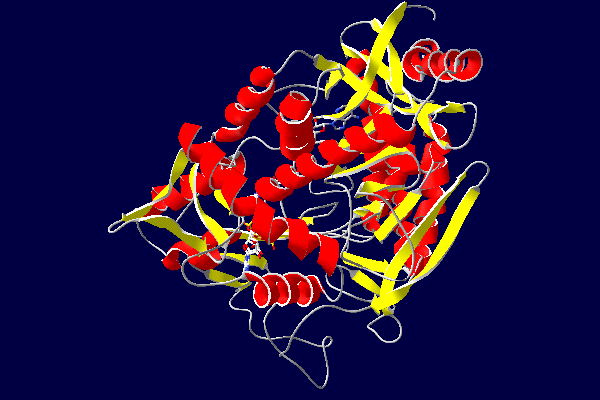

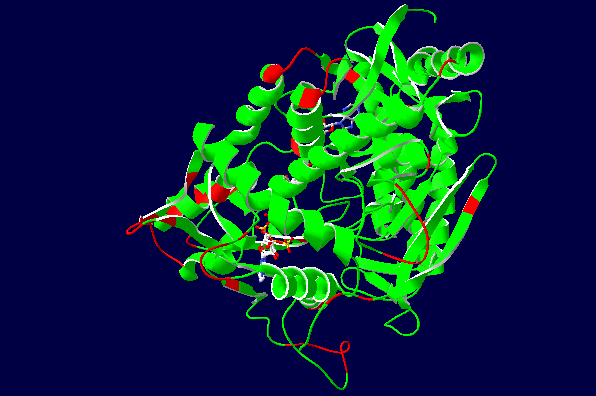
Pelagibacter ubique

# A

# B

3D ribbon structure colored by model confidence 3D ribbon structure colored by secondary structure elements

AprA model shown from front side


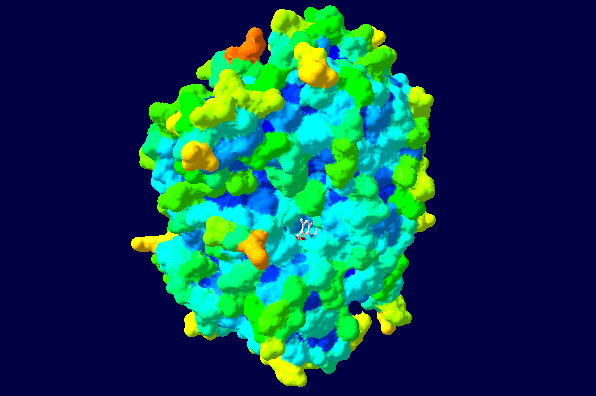

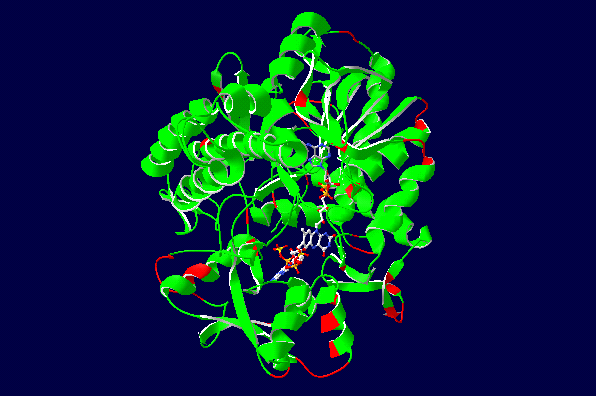


# C

# D

3D ribbon structure colored by model confidence Protein molecular surface colored by accessibility

AprA model shown from top view


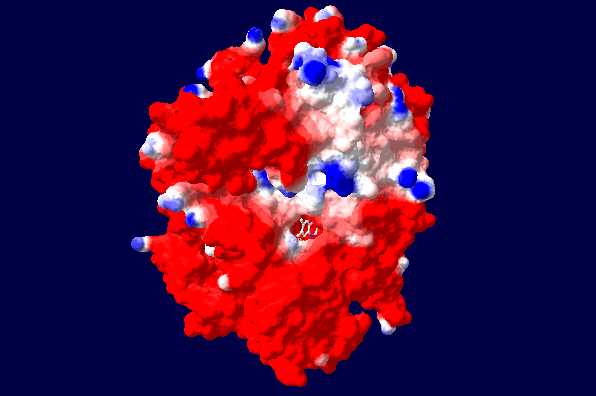

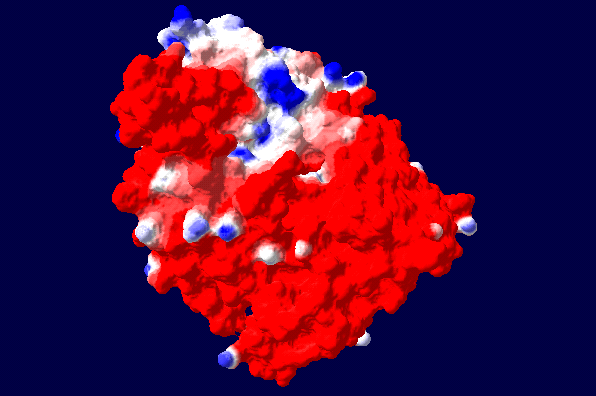

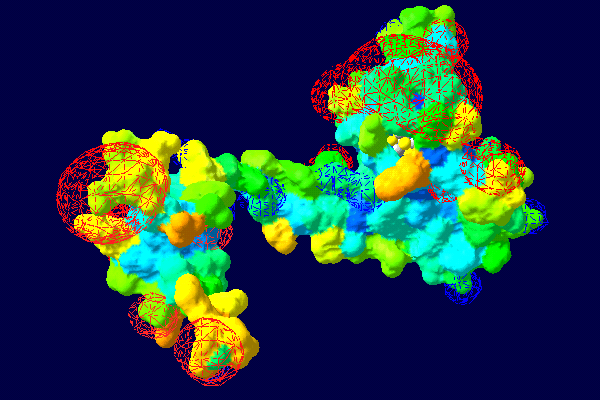

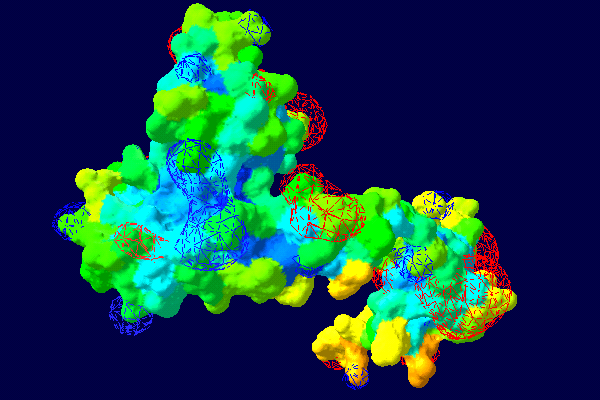


# E

# F

AprA model shown from top view AprA model shown from back side

Protein molecular surface colored by calculated electrostatic potential (electric charge at the molecular surface is colored with a red (negative), white (neutral, and blue (positive) color gradient)


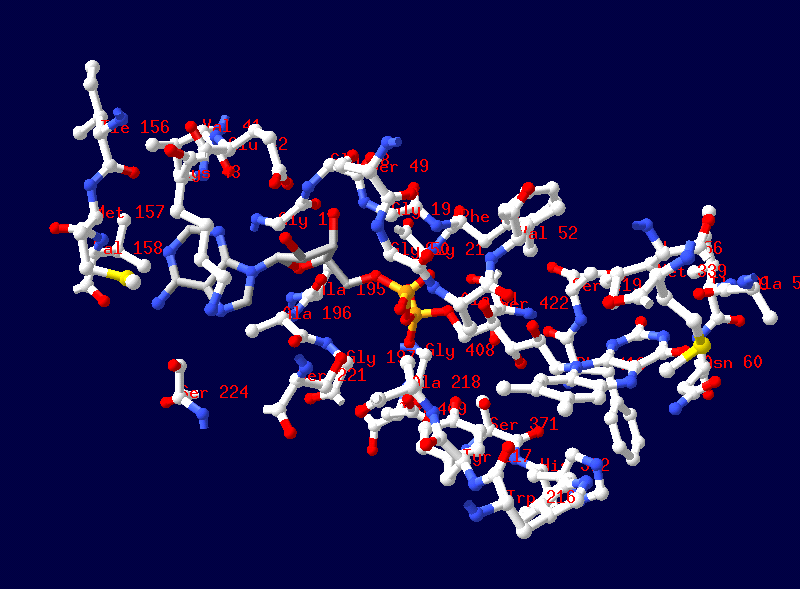

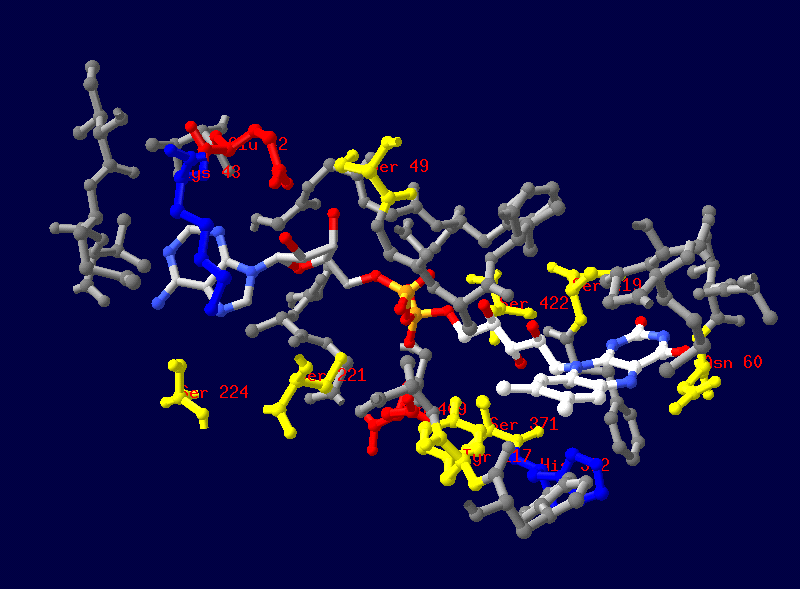


# G

# H

FAD surrounding protein matrix: residues present in a distance of < 4.1Å are shown (amino acids are coloured as follows: positively charged, basic AA, blue; negatively charged, acidic AA, red; polar AA, yellow; and unpolar, uncharged AA, grey)


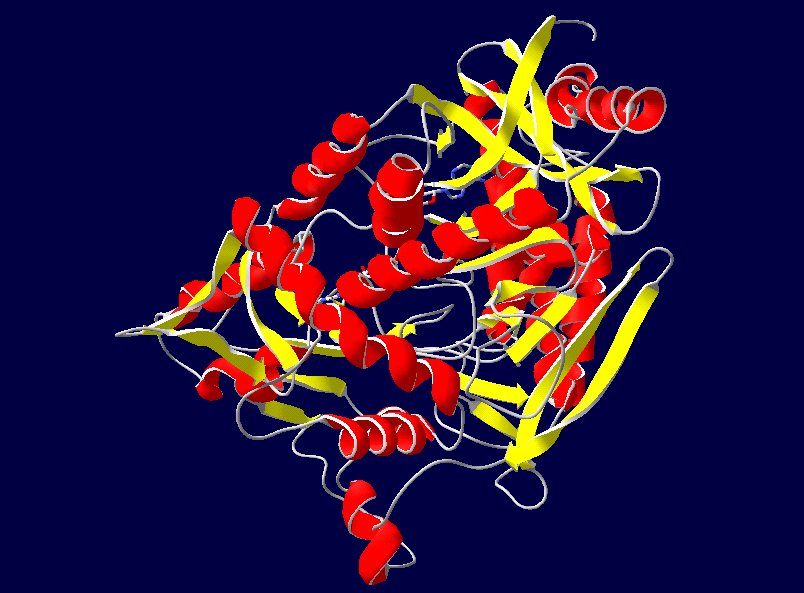

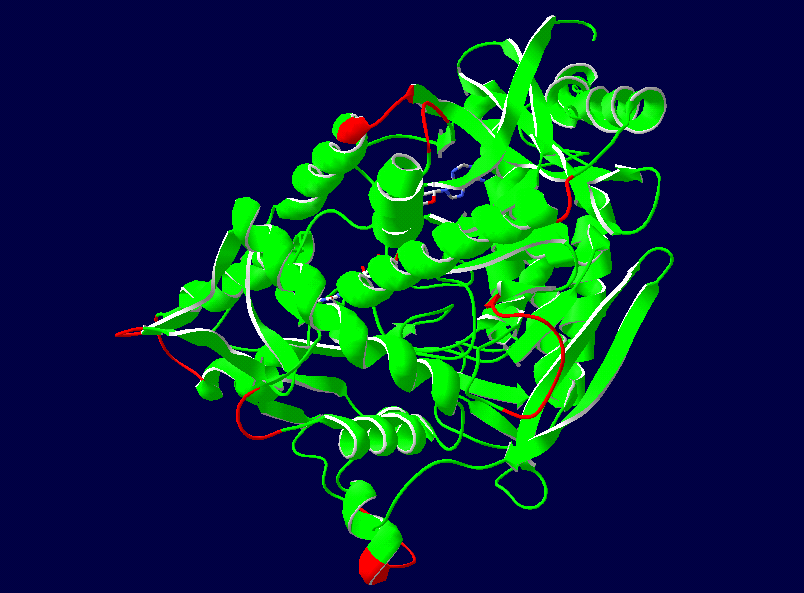
EBAC2C11

# A

# B

3D ribbon structure colored by model confidence 3D ribbon structure colored by secondary structure elements

AprA model shown from front side


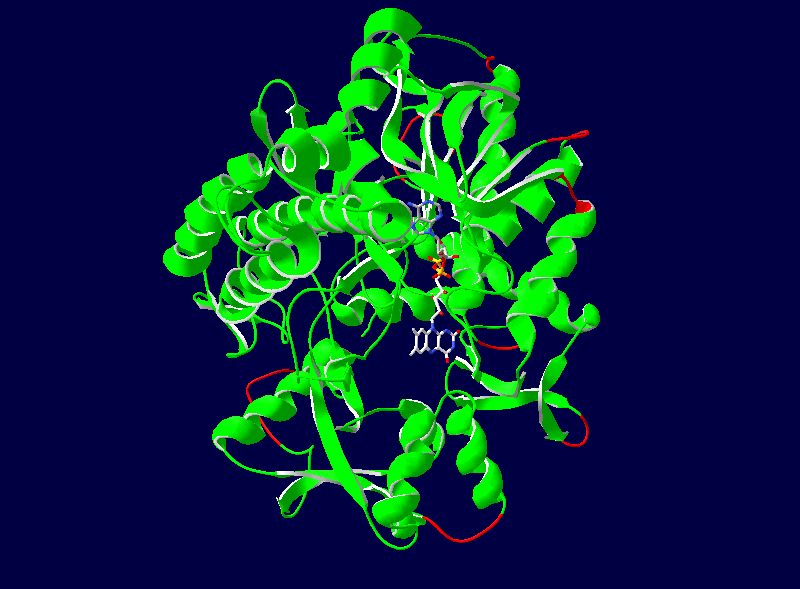

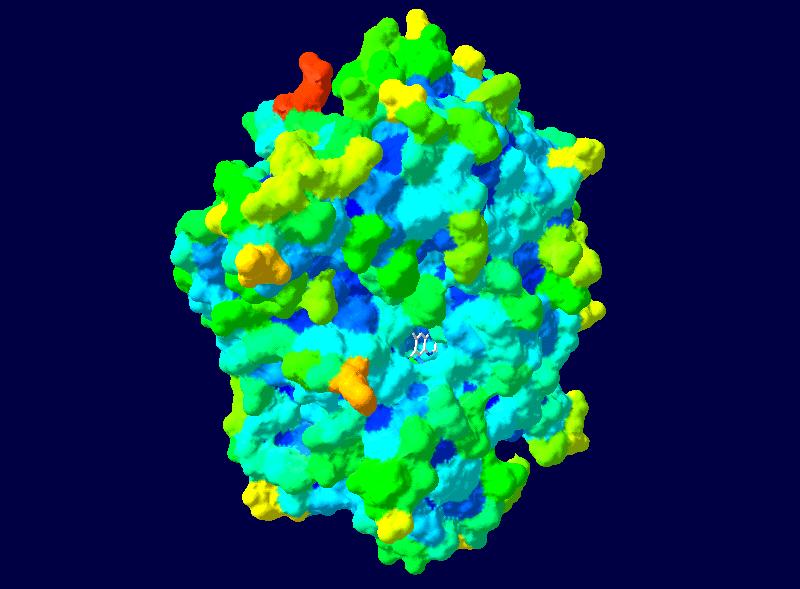


# C

# D

3D ribbon structure colored by model confidence Protein molecular surface colored by accessibility

AprA model shown from top view


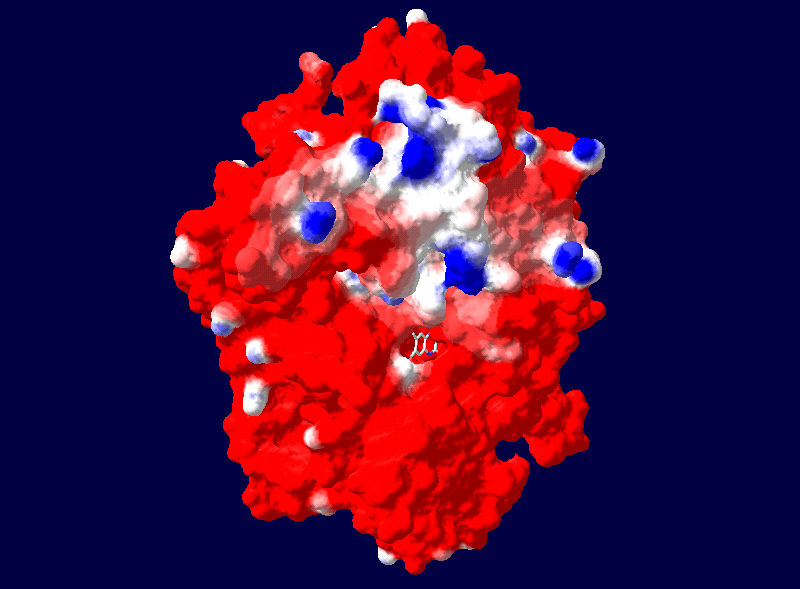

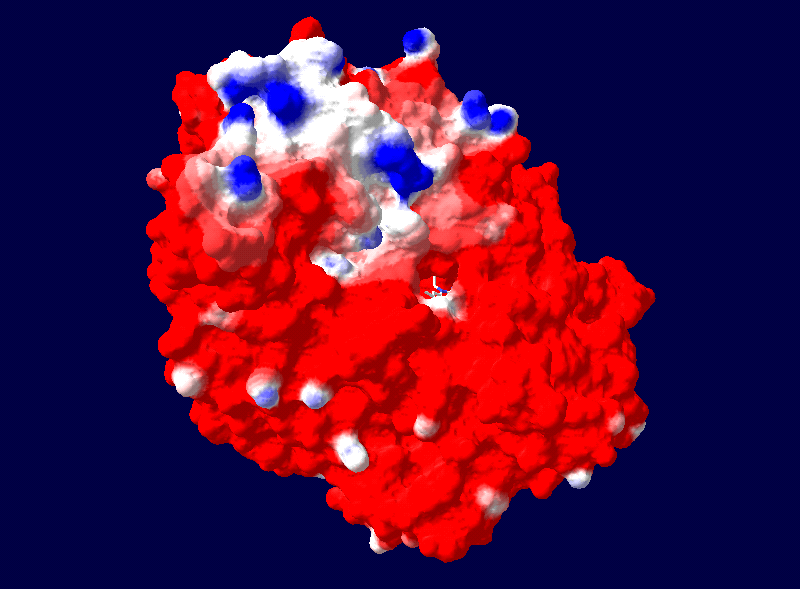

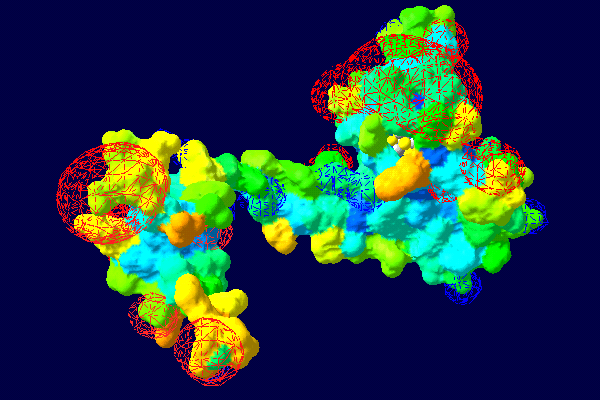

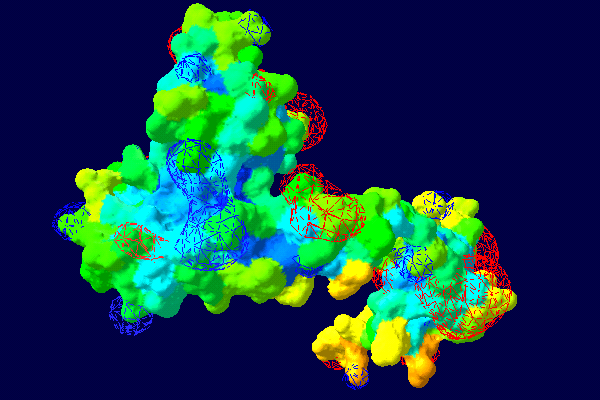


# E

# F

AprA model shown from top view AprA model shown from back side

Protein molecular surface colored by calculated electrostatic potential (electric charge at the molecular surface is colored with a red (negative), white (neutral, and blue (positive) color gradient)


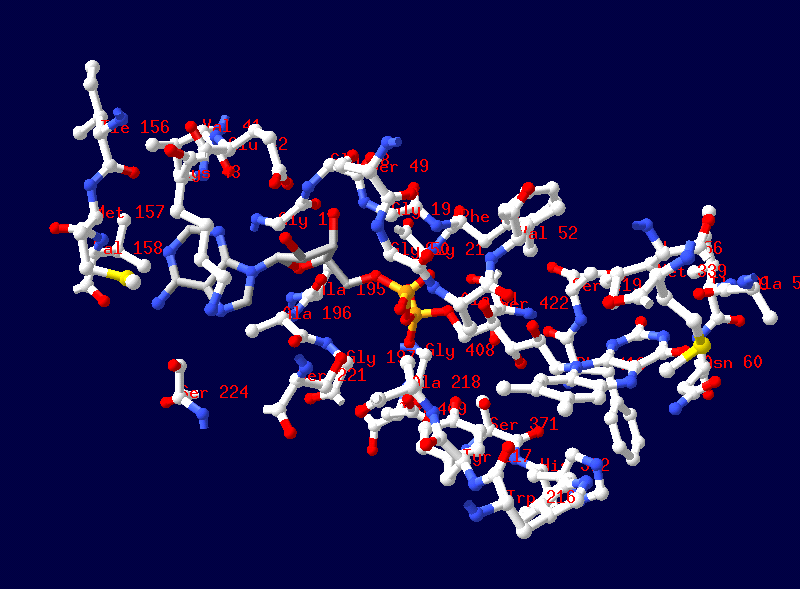

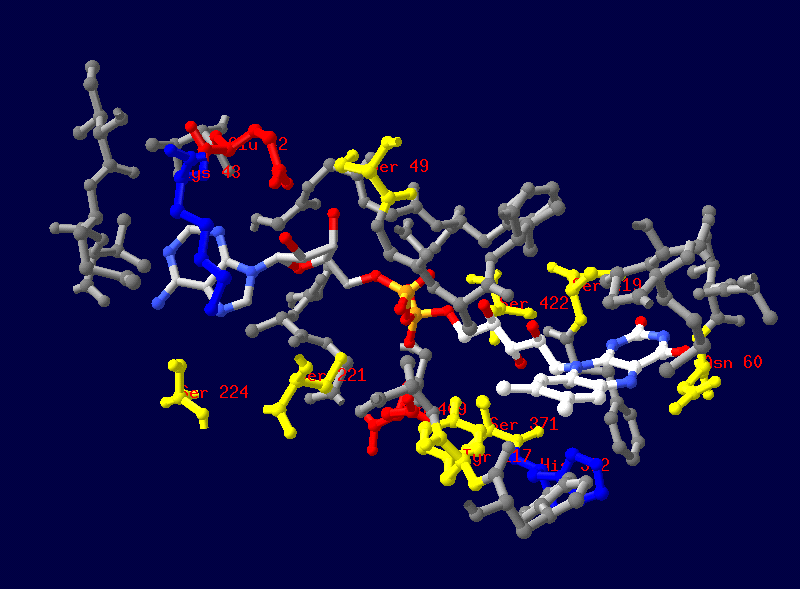


# G

# H

FAD surrounding protein matrix: residues present in a distance of < 4.1Å are shown (amino acids are coloured as follows: positively charged, basic AA, blue; negatively charged, acidic AA, red; polar AA, yellow; and unpolar, uncharged AA, grey)

###### Crenarchaeal SRP


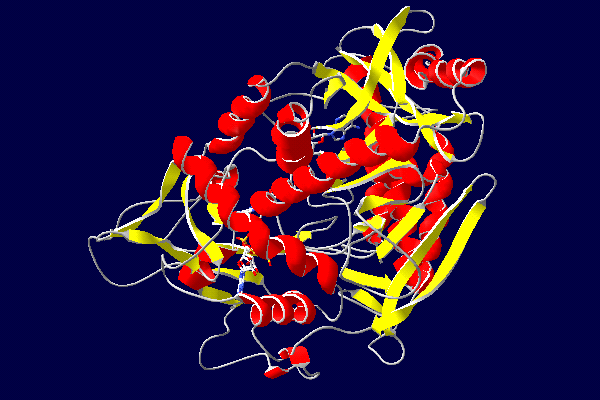

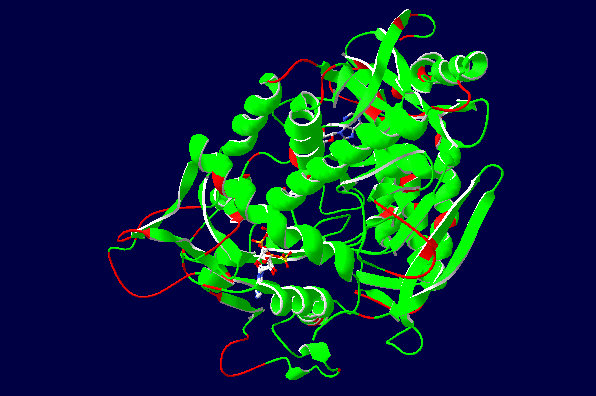
Pyrobaculum calidifontis

# A

# B

3D ribbon structure colored by model confidence 3D ribbon structure colored by secondary structure elements


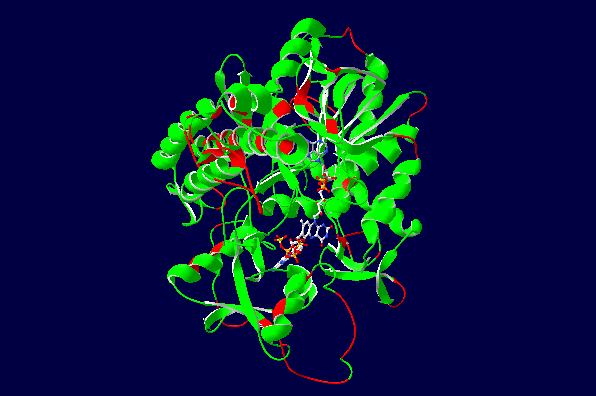

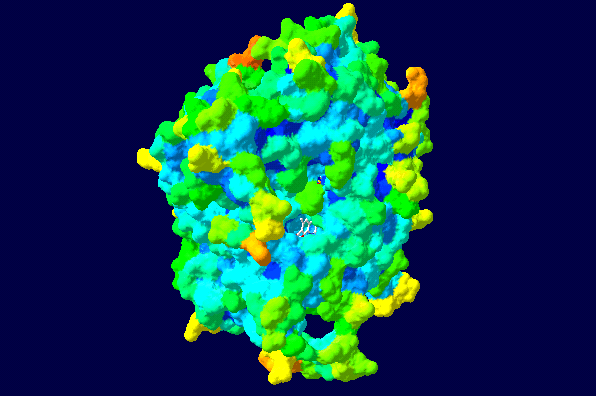
 AprA model shown from front side

# C

# D


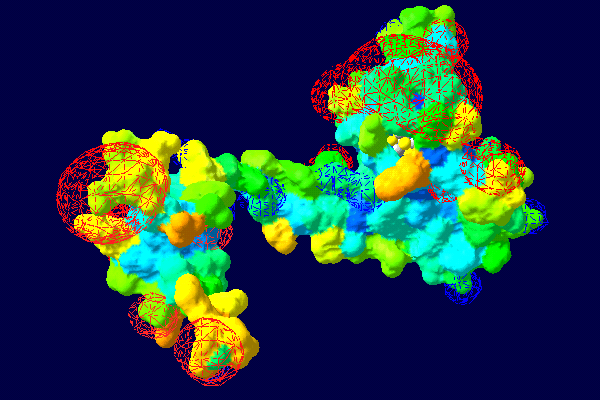

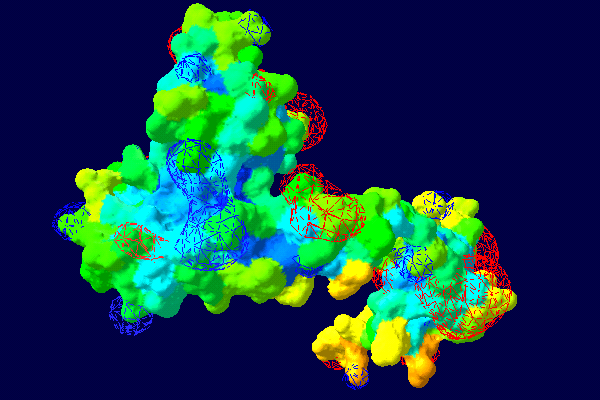
3D ribbon structure colored by model confidence Protein molecular surface colored by accessibility


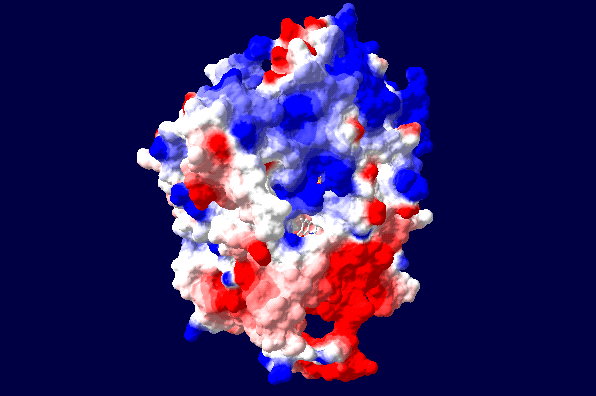

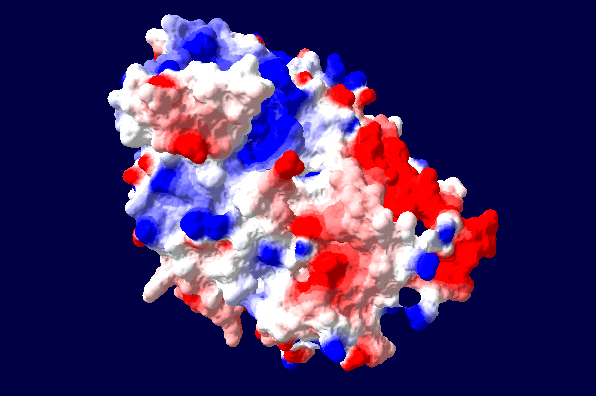
AprA model shown from top view
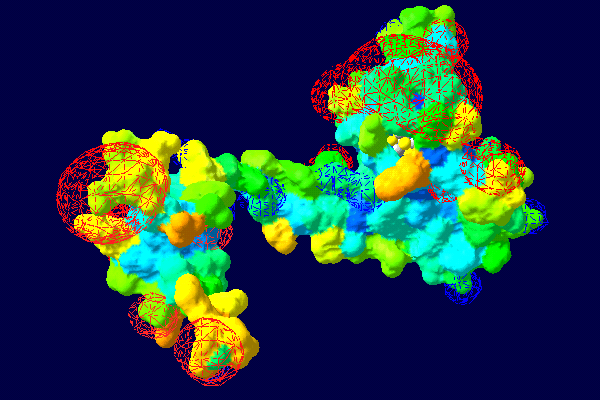

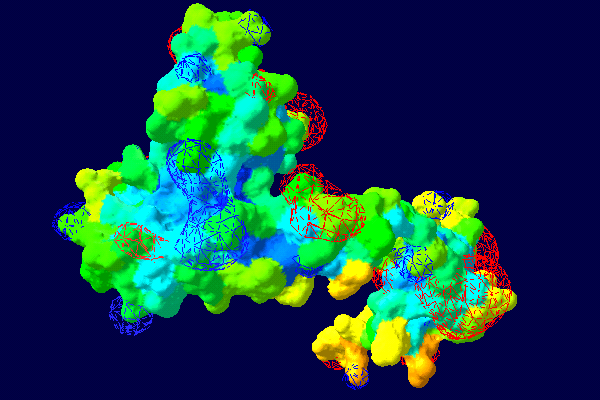


# E

# F

AprA model shown from top view AprA model shown from back side


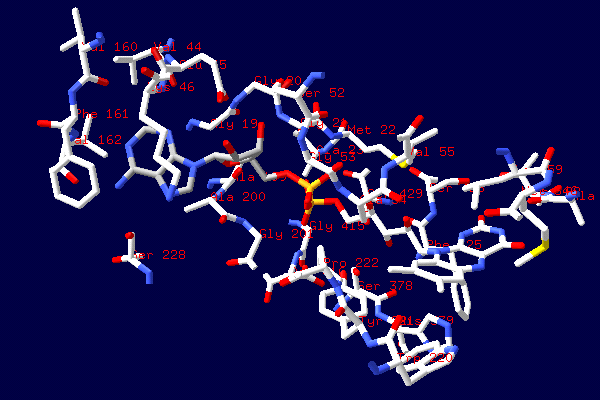
Protein molecular surface colored by calculated electrostatic potential (electric charge at the molecular surface is colored with a red (negative), white (neutral, and blue (positive) color gradient)


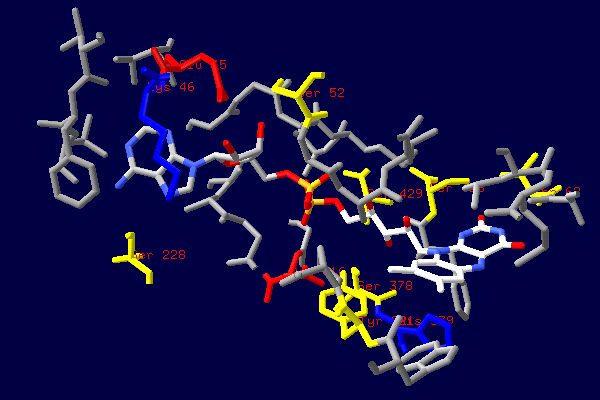


# G

# H

FAD surrounding protein matrix: residues present in a distance of < 4.1Å are shown (amino acids are coloured as follows: positively charged, basic AA, blue; negatively charged, acidic AA, red; polar AA, yellow; and unpolar, uncharged AA, grey)


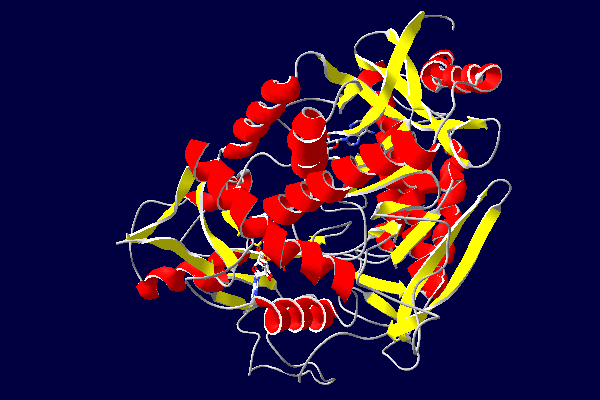

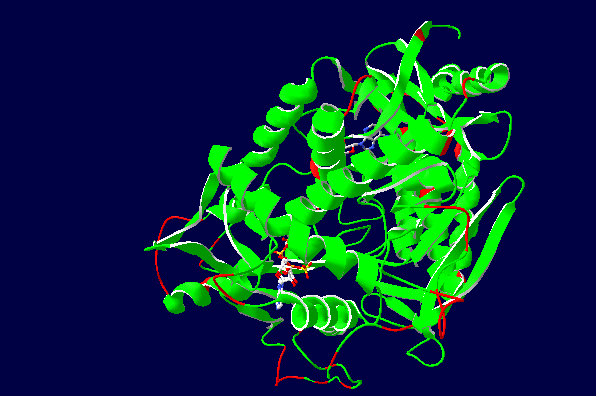
Caldivirga maquilingensis

# A

# B

3D ribbon structure colored by model confidence 3D ribbon structure colored by secondary structure elements

AprA model shown from front side


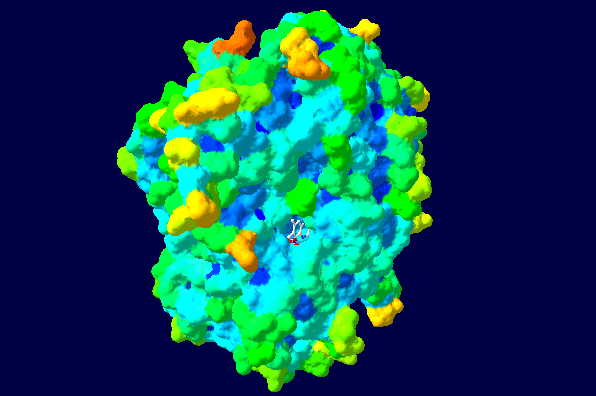

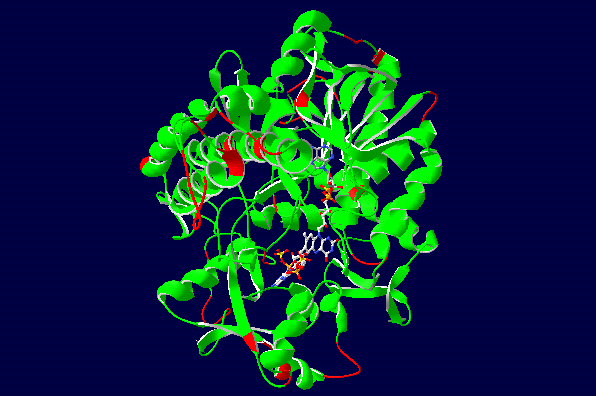


# C

# D

3D ribbon structure colored by model confidence Protein molecular surface colored by accessibility

AprA model shown from top view


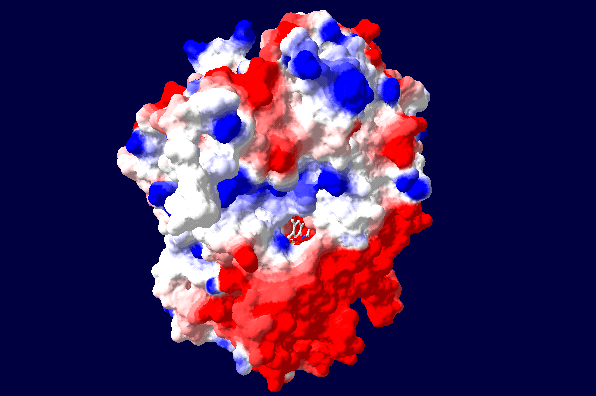

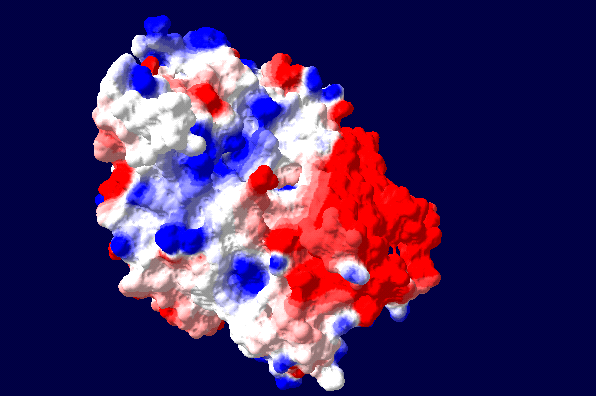

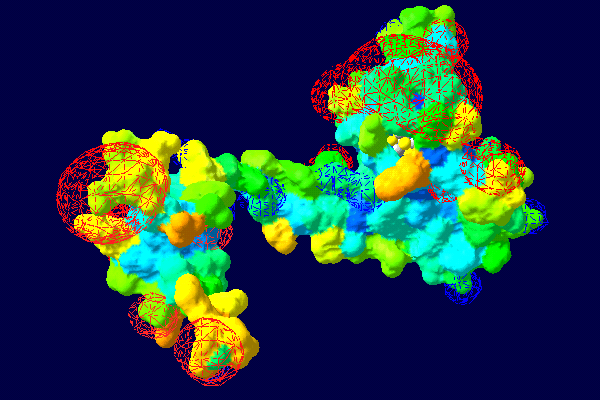

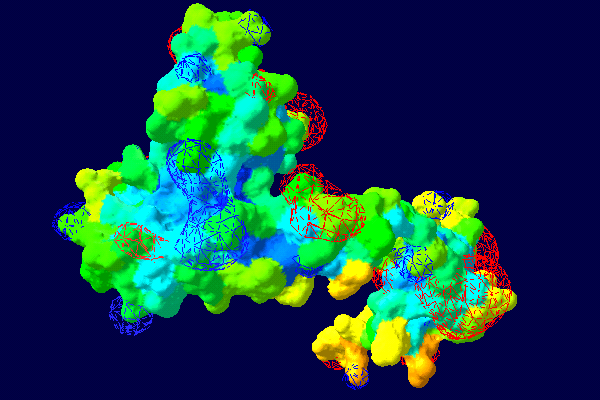


# E

# F

AprA model shown from top view AprA model shown from back side

Protein molecular surface colored by calculated electrostatic potential (electric charge at the molecular surface is colored with a red (negative), white (neutral, and blue (positive) color gradient)


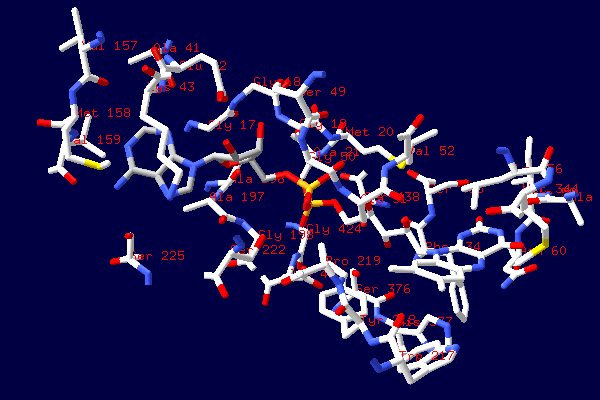

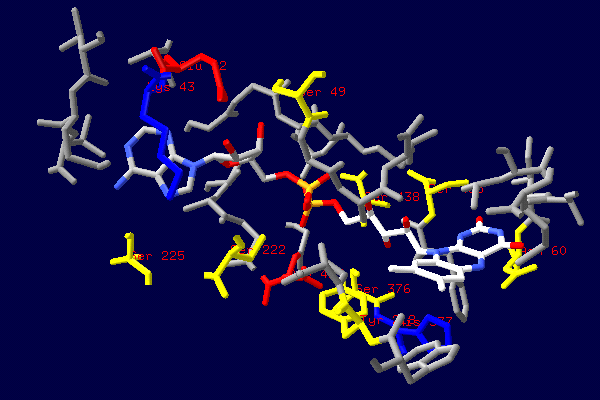


# G

# H

FAD surrounding protein matrix: residues present in a distance of < 4.1Å are shown (amino acids are coloured as follows: positively charged, basic AA, blue; negatively charged, acidic AA, red; polar AA, yellow; and unpolar, uncharged AA, grey)

###### SRB and related SOB Apr lineage II


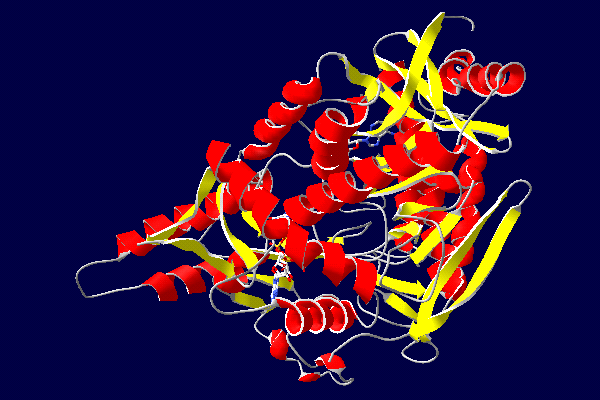

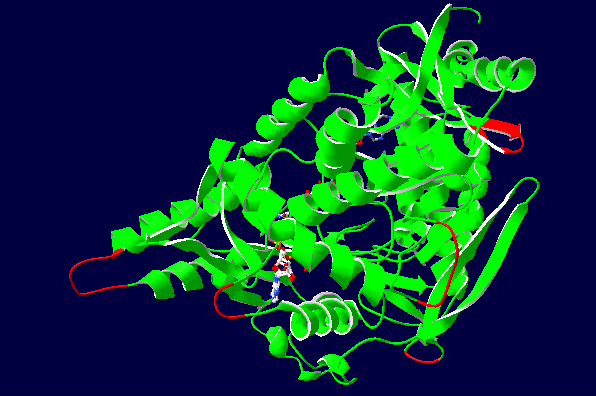
Desulfotomaculum reducens

# A

# B

3D ribbon structure colored by model confidence 3D ribbon structure colored by secondary structure elements


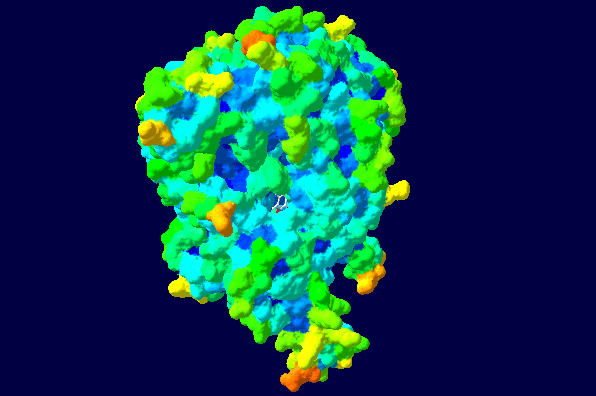

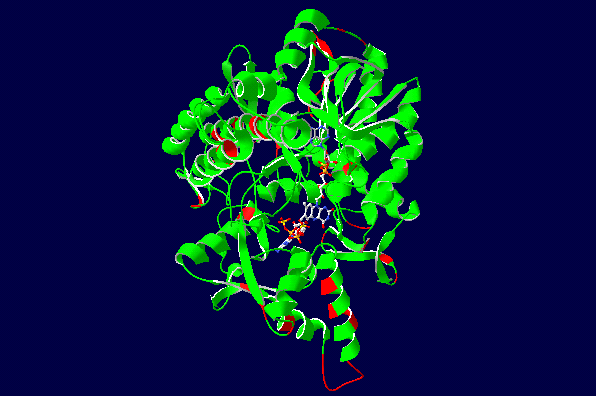
 AprA model shown from front side

# C

# D


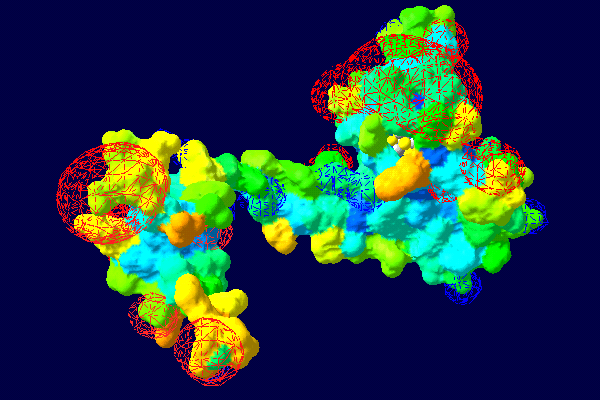

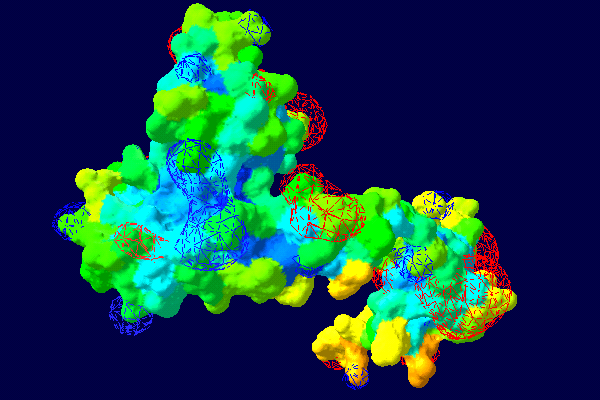
3D ribbon structure colored by model confidence Protein molecular surface colored by accessibility


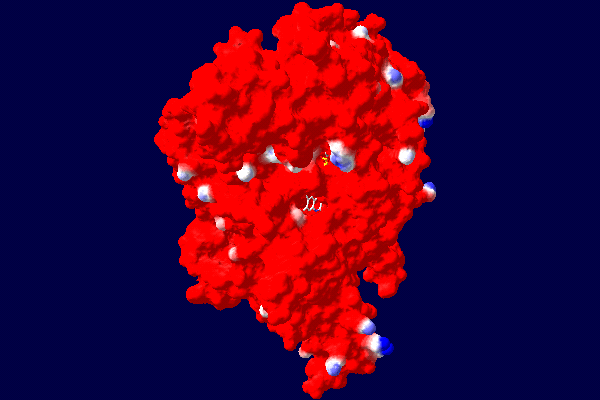

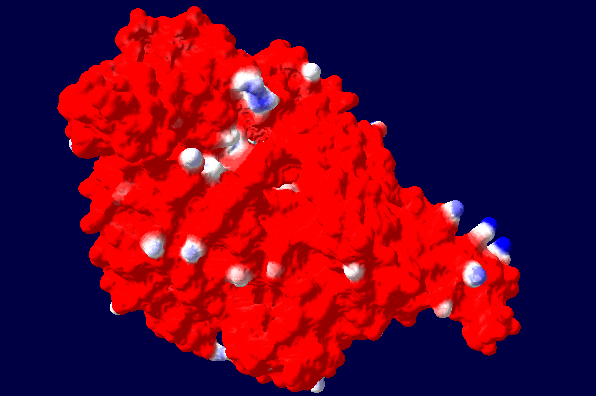
AprA model shown from top view
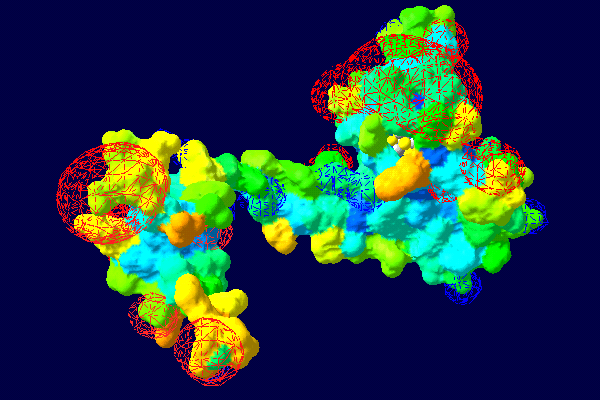

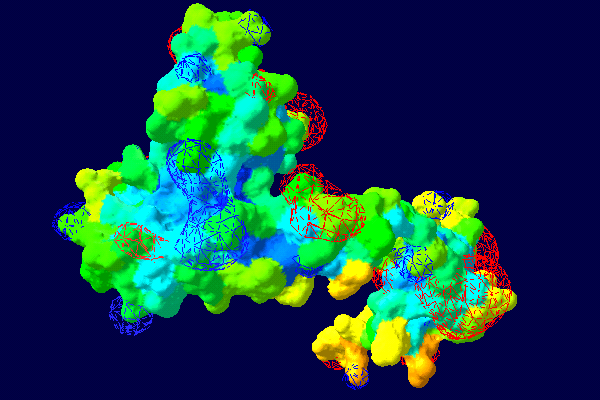


# E

# F

AprA model shown from top view AprA model shown from back side

Protein molecular surface colored by calculated electrostatic potential (electric charge at the molecular surface is colored with a red (negative), white (neutral, and blue (positive) color gradient)


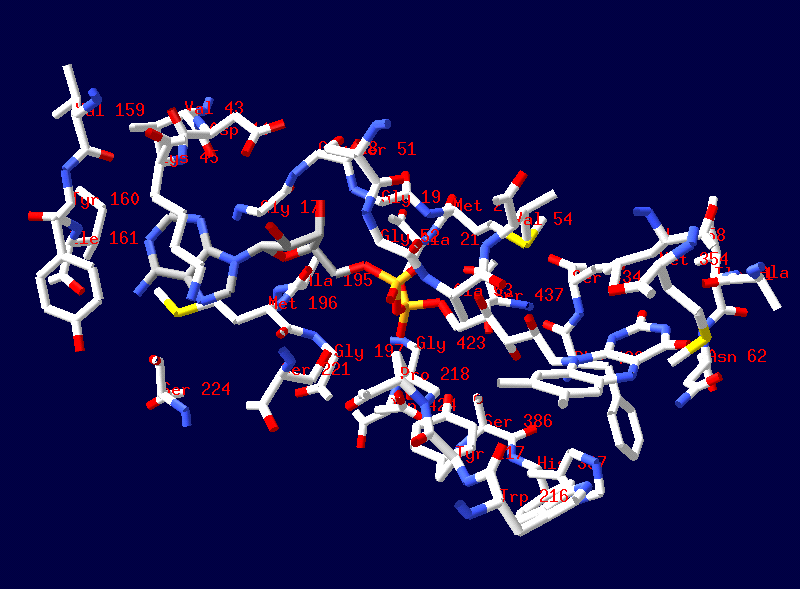

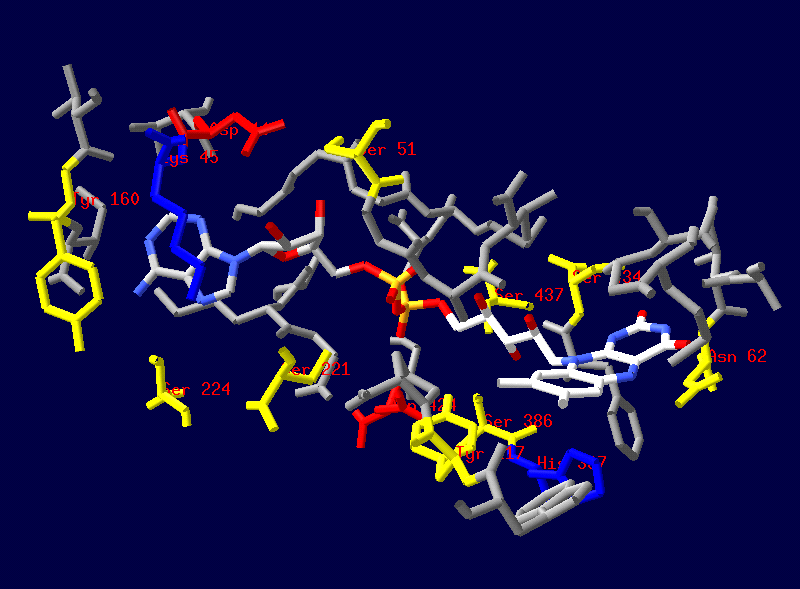


# G

# H

FAD surrounding protein matrix: residues present in a distance of < 4.1Å are shown (amino acids are coloured as follows: positively charged, basic AA, blue; negatively charged, acidic AA, red; polar AA, yellow; and unpolar, uncharged AA, grey)


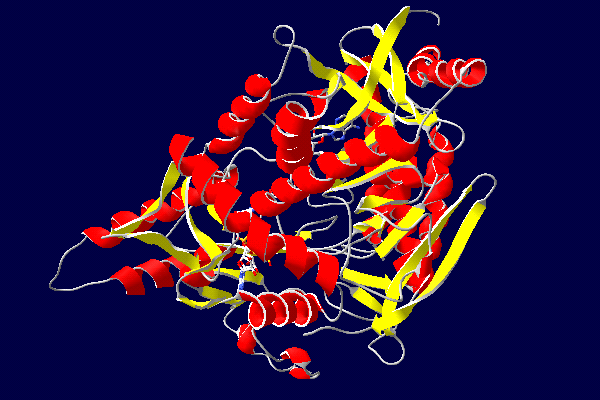
Syntrophobacter fumaroxidans

# A

# B

3D ribbon structure colored by model confidence 3D ribbon structure colored by secondary structure elements

AprA model shown from front side

# C

# D

3D ribbon structure colored by model confidence Protein molecular surface colored by accessibility

AprA model shown from top view

# E

# F

AprA model shown from top view AprA model shown from back side

Protein molecular surface colored by calculated electrostatic potential (electric charge at the molecular surface is colored with a red (negative), white (neutral, and blue (positive) color gradient)

# G

# H

FAD surrounding protein matrix: residues present in a distance of < 4.1Å are shown (amino acids are coloured as follows: positively charged, basic AA, blue; negatively charged, acidic AA, red; polar AA, yellow; and unpolar, uncharged AA, grey)

fosws39f7

# A

# B

3D ribbon structure colored by model confidence 3D ribbon structure colored by secondary structure elements

AprA model shown from front side

# C

# D

3D ribbon structure colored by model confidence Protein molecular surface colored by accessibility

AprA model shown from top view

# E

# F

AprA model shown from top view AprA model shown from back side

Protein molecular surface colored by calculated electrostatic potential (electric charge at the molecular surface is colored with a red (negative), white (neutral, and blue (positive) color gradient)

# G

# H

FAD surrounding protein matrix: residues present in a distance of < 4.1Å are shown (amino acids are coloured as follows: positively charged, basic AA, blue; negatively charged, acidic AA, red; polar AA, yellow; and unpolar, uncharged AA, grey)

fosws7f8

# A

# B

3D ribbon structure colored by model confidence 3D ribbon structure colored by secondary structure elements

AprA model shown from front side

# C

# D

3D ribbon structure colored by model confidence Protein molecular surface colored by accessibility

AprA model shown from top view

# E

# F

AprA model shown from top view AprA model shown from back side

Protein molecular surface colored by calculated electrostatic potential (electric charge at the molecular surface is colored with a red (negative), white (neutral, and blue (positive) color gradient)

# G

# H

FAD surrounding protein matrix: residues present in a distance of < 4.1Å are shown (amino acids are coloured as follows: positively charged, basic AA, blue; negatively charged, acidic AA, red; polar AA, yellow; and unpolar, uncharged AA, grey)

Thermodesulfobacterium commune

# A

# B

3D ribbon structure colored by model confidence 3D ribbon structure colored by secondary structure elements

AprA model shown from front side

# C

# D

3D ribbon structure colored by model confidence Protein molecular surface colored by accessibility

AprA model shown from top view

# E

# F

AprA model shown from top view AprA model shown from back side

Protein molecular surface colored by calculated electrostatic potential (electric charge at the molecular surface is colored with a red (negative), white (neutral, and blue (positive) color gradient)

# G

# H

FAD surrounding protein matrix: residues present in a distance of < 4.1Å are shown (amino acids are coloured as follows: positively charged, basic AA, blue; negatively charged, acidic AA, red; polar AA, yellow; and unpolar, uncharged AA, grey)

Desulfovibrio desulfuricans

# A

# B

3D ribbon structure colored by model confidence 3D ribbon structure colored by secondary structure elements

AprA model shown from front side

# C

# D

3D ribbon structure colored by model confidence Protein molecular surface colored by accessibility

AprA model shown from top view

# E

# F

AprA model shown from top view AprA model shown from back side

Protein molecular surface colored by calculated electrostatic potential (electric charge at the molecular surface is colored with a red (negative), white (neutral, and blue (positive) color gradient)

# G

# H

FAD surrounding protein matrix: residues present in a distance of < 4.1Å are shown (amino acids are coloured as follows: positively charged, basic AA, blue; negatively charged, acidic AA, red; polar AA, yellow; and unpolar, uncharged AA, grey)

Desulfovibrio vulgaris

# A

# B

3D ribbon structure colored by model confidence 3D ribbon structure colored by secondary structure elements

AprA model shown from front side

# C

# D

3D ribbon structure colored by model confidence Protein molecular surface colored by accessibility

AprA model shown from top view

# E

# F

AprA model shown from top view AprA model shown from back side

Protein molecular surface colored by calculated electrostatic potential (electric charge at the molecular surface is colored with a red (negative), white (neutral, and blue (positive) color gradient)

# G

# H

FAD surrounding protein matrix: residues present in a distance of < 4.1Å are shown (amino acids are coloured as follows: positively charged, basic AA, blue; negatively charged, acidic AA, red; polar AA, yellow; and unpolar, uncharged AA, grey)

Desulfobulbus sp.

# A

# B

3D ribbon structure colored by model confidence 3D ribbon structure colored by secondary structure elements

AprA model shown from front side

# C

# D

3D ribbon structure colored by model confidence Protein molecular surface colored by accessibility

AprA model shown from top view

# E

# F

AprA model shown from top view AprA model shown from back side

Protein molecular surface colored by calculated electrostatic potential (electric charge at the molecular surface is colored with a red (negative), white (neutral, and blue (positive) color gradient)

# G

# H

FAD surrounding protein matrix: residues present in a distance of < 4.1Å are shown (amino acids are coloured as follows: positively charged, basic AA, blue; negatively charged, acidic AA, red; polar AA, yellow; and unpolar, uncharged AA, grey)

Desulfotalea psychrophila

# A

# B

3D ribbon structure colored by model confidence 3D ribbon structure colored by secondary structure elements

AprA model shown from front side

# C

# D

3D ribbon structure colored by model confidence Protein molecular surface colored by accessibility

AprA model shown from top view

# E

# F

AprA model shown from top view AprA model shown from back side

Protein molecular surface colored by calculated electrostatic potential (electric charge at the molecular surface is colored with a red (negative), white (neutral, and blue (positive) color gradient)

# G

# H

FAD surrounding protein matrix: residues present in a distance of < 4.1Å are shown (amino acids are coloured as follows: positively charged, basic AA, blue; negatively charged, acidic AA, red; polar AA, yellow; and unpolar, uncharged AA, grey)

O. algarvensis Delta 1 symbiont

# A

# B

3D ribbon structure colored by model confidence 3D ribbon structure colored by secondary structure elements

AprA model shown from front side

# C

# D

3D ribbon structure colored by model confidence Protein molecular surface colored by accessibility

AprA model shown from top view

# E

# F

AprA model shown from top view AprA model shown from back side

Protein molecular surface colored by calculated electrostatic potential (electric charge at the molecular surface is colored with a red (negative), white (neutral, and blue (positive) color gradient)

# G

# H

FAD surrounding protein matrix: residues present in a distance of < 4.1Å are shown (amino acids are coloured as follows: positively charged, basic AA, blue; negatively charged, acidic AA, red; polar AA, yellow; and unpolar, uncharged AA, grey)

Thermodesulfovibrio yellowstonii

# A

# B

3D ribbon structure colored by model confidence 3D ribbon structure colored by secondary structure elements

AprA model shown from front side

# C

# D

3D ribbon structure colored by model confidence Protein molecular surface colored by accessibility

AprA model shown from top view

# E

# F

AprA model shown from top view AprA model shown from back side

Protein molecular surface colored by calculated electrostatic potential (electric charge at the molecular surface is colored with a red (negative), white (neutral, and blue (positive) color gradient)

# G

# H

FAD surrounding protein matrix: residues present in a distance of < 4.1Å are shown (amino acids are coloured as follows: positively charged, basic AA, blue; negatively charged, acidic AA, red; polar AA, yellow; and unpolar, uncharged AA, grey)

Chlorobaculum tepidum

# A

# B

3D ribbon structure colored by model confidence 3D ribbon structure colored by secondary structure elements

AprA model shown from front side

# C

# D

3D ribbon structure colored by model confidence Protein molecular surface colored by accessibility

AprA model shown from top view

# E

# F

AprA model shown from top view AprA model shown from back side

Protein molecular surface colored by calculated electrostatic potential (electric charge at the molecular surface is colored with a red (negative), white (neutral, and blue (positive) color gradient)

# G

# H

FAD surrounding protein matrix: residues present in a distance of < 4.1Å are shown (amino acids are coloured as follows: positively charged, basic AA, blue; negatively charged, acidic AA, red; polar AA, yellow; and unpolar, uncharged AA, grey)

Thiobacillus denitrificans

# A

# B

3D ribbon structure colored by model confidence 3D ribbon structure colored by secondary structure elements

AprA model shown from front side

# C

# D

3D ribbon structure colored by model confidence Protein molecular surface colored by accessibility

AprA model shown from top view

# E

# F

AprA model shown from top view AprA model shown from back side

Protein molecular surface colored by calculated electrostatic potential (electric charge at the molecular surface is colored with a red (negative), white (neutral, and blue (positive) color gradient)

# G

# H

FAD surrounding protein matrix: residues present in a distance of < 4.1Å are shown (amino acids are coloured as follows: positively charged, basic AA, blue; negatively charged, acidic AA, red; polar AA, yellow; and unpolar, uncharged AA, grey)
