# Supplementary material for: Homology Modeling of Dissimilatory APS Reductases (AprBA) of Sulfur-Oxidizing and Sulfate-Reducing Prokaryotes
Source: PLoS One. 2008 Jan 30;3(1):e1514. doi: 10.1371/journal.pone.0001514 (PMC2211403; doi:10.1371/journal.pone.0001514)

**Supplementary data material Figure S4**: **APS/ sulfite binding site – active center in AprA**

**models of SRP and SOB** (distances of the residues to the N5 atom of FAD are given in Å, isoalloxazine ring interacting residues are green coloured, polar and basic amino acids are coloured in yellow and blue, respectively)

Reference structure Archaeoglobus fulgidus


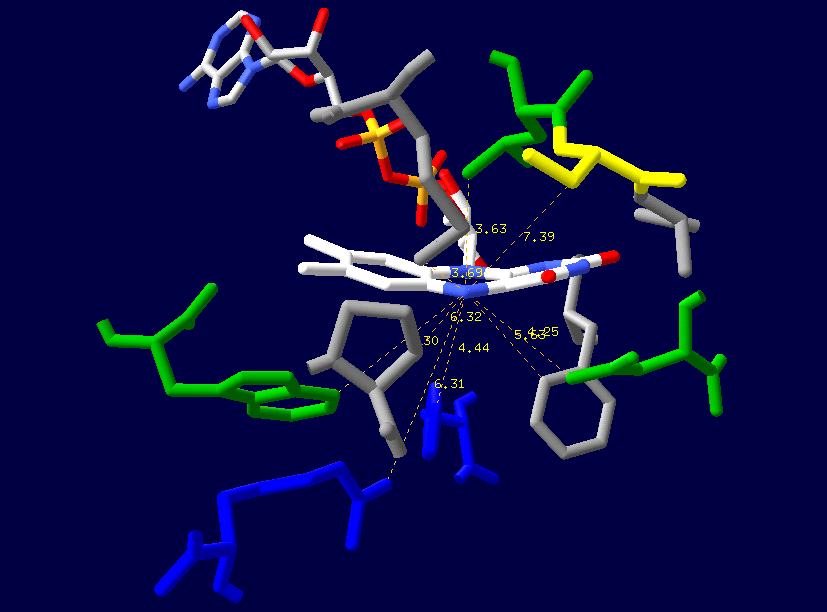

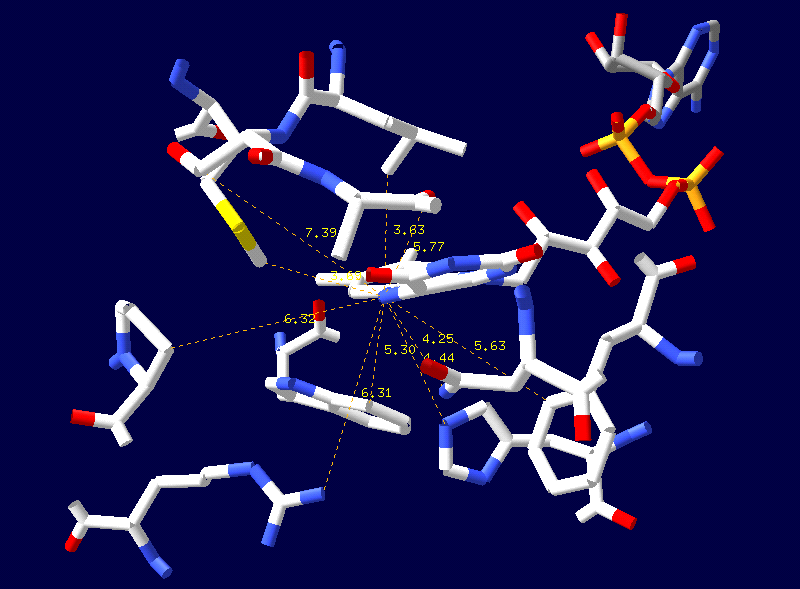


SOB Apr lineage I

*
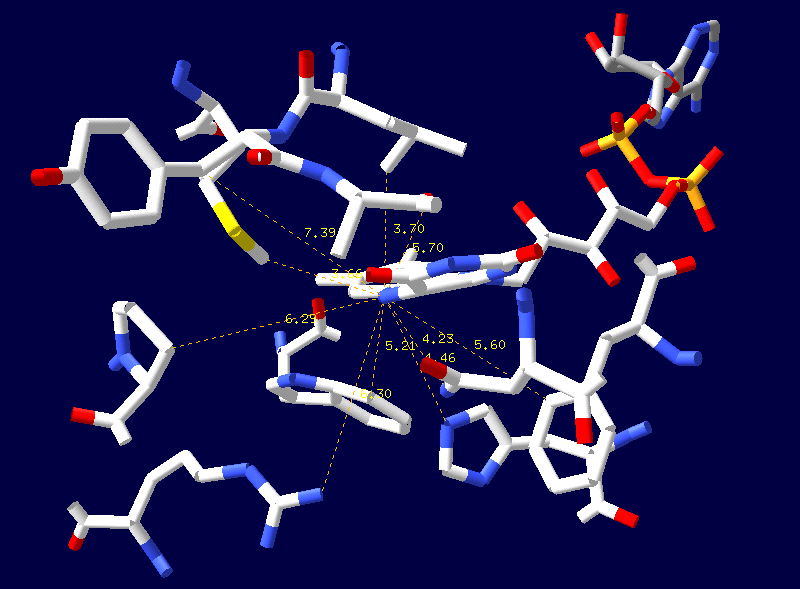

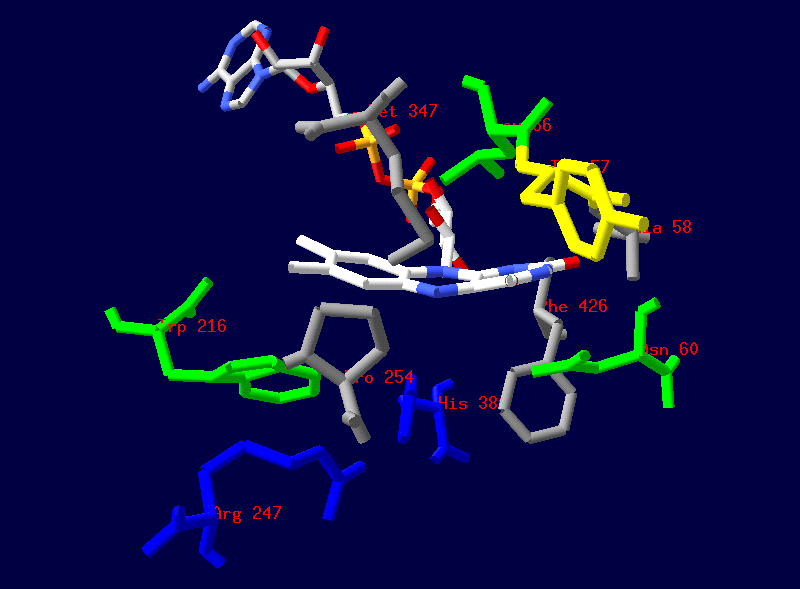
*

*Allochromatium vinosum*

*
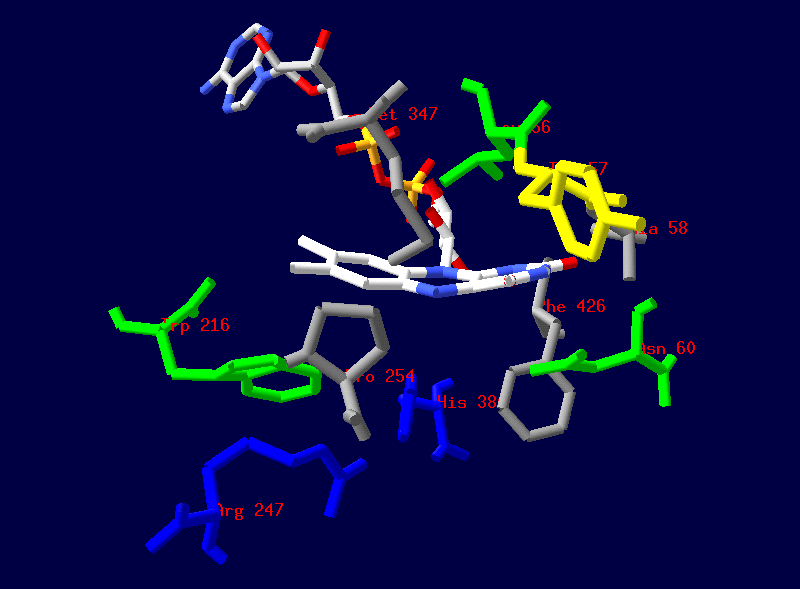

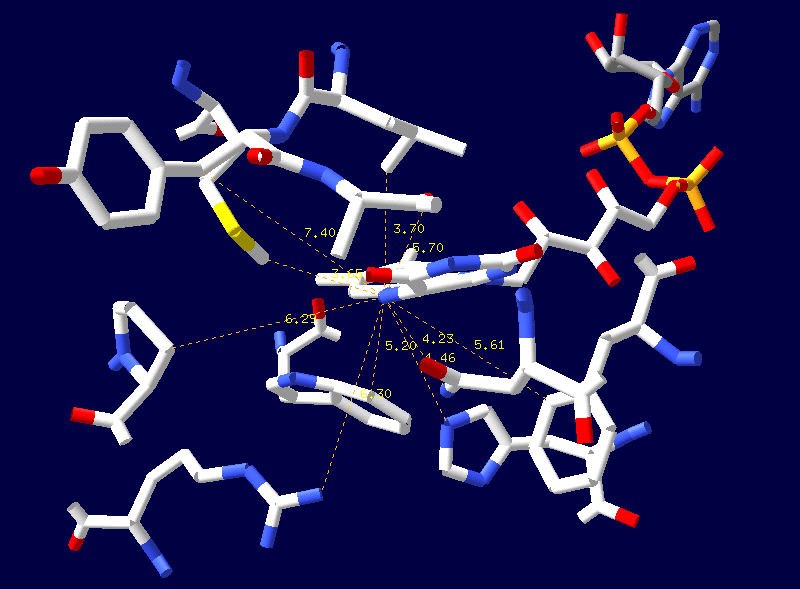
*

*
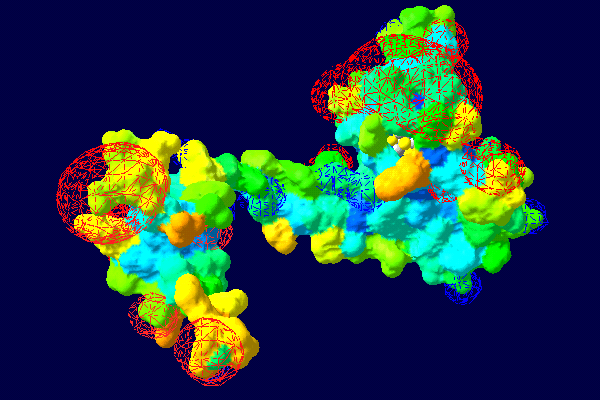

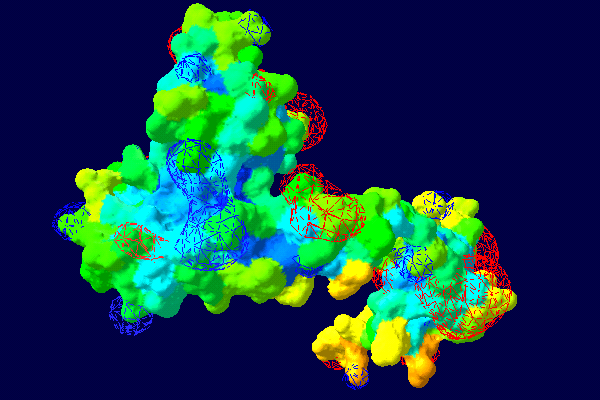

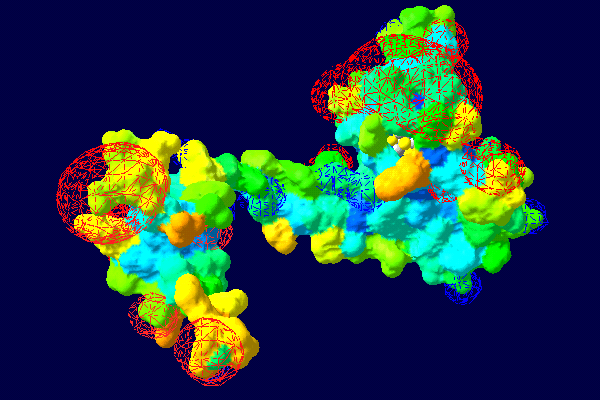

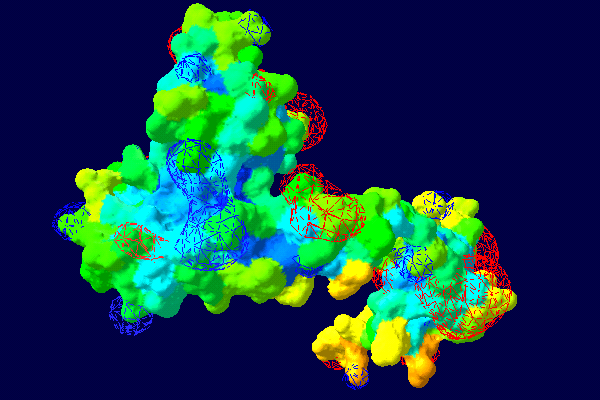
Thiobacillus denitrificans*

*
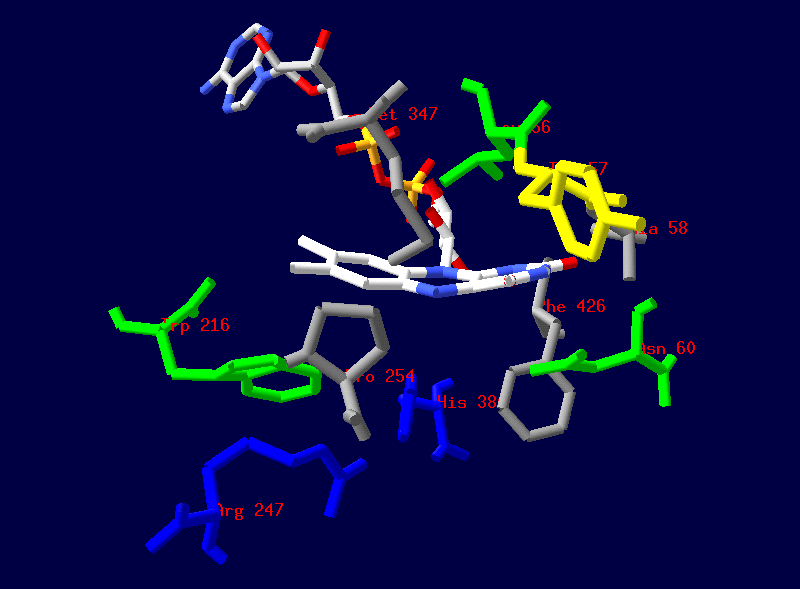

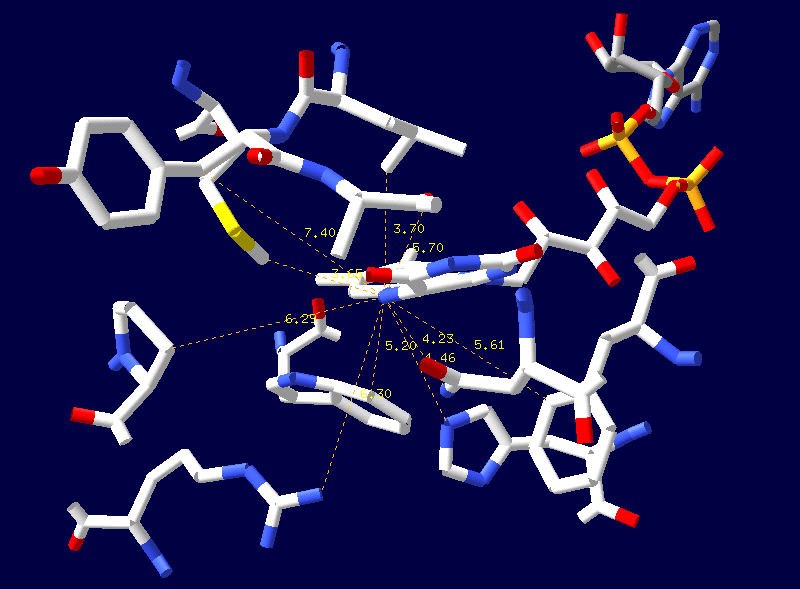
*

*Cdt.* Ruthia magnifica

SOB Apr lineage I

*
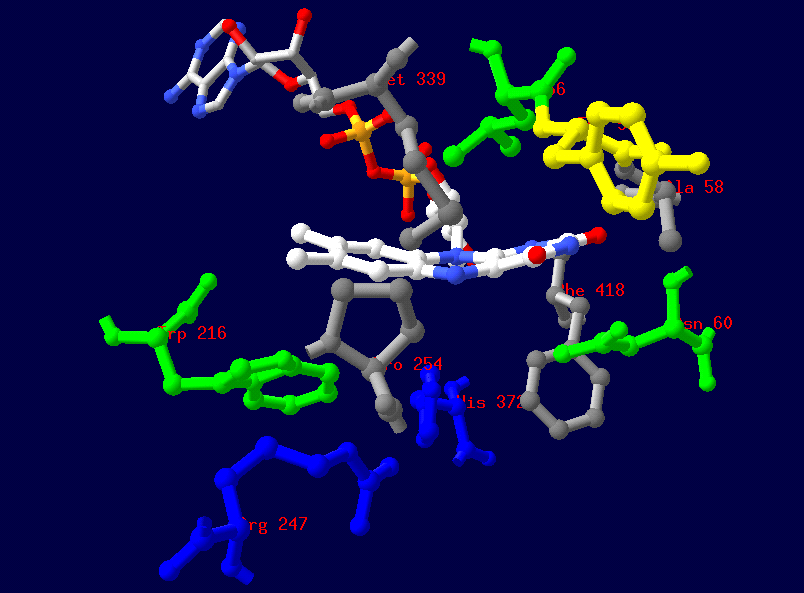

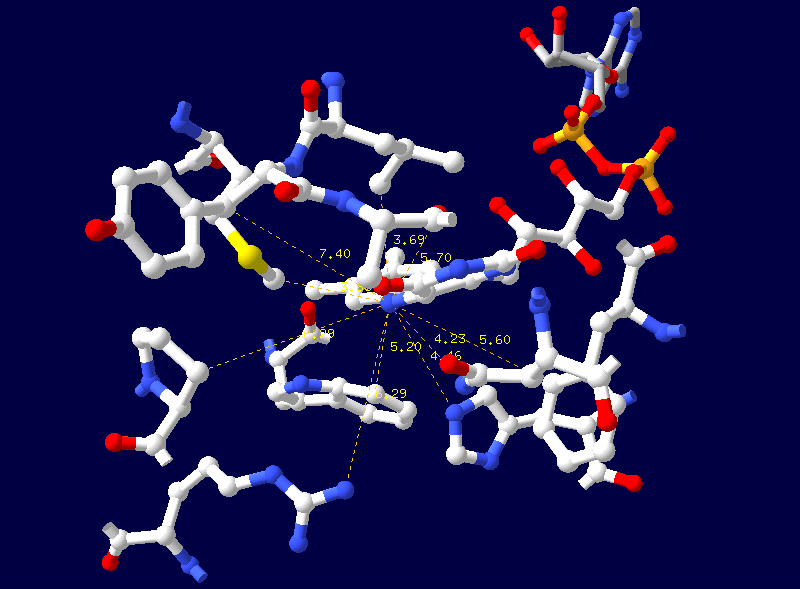
*

*Pelagibacter ubique*

*
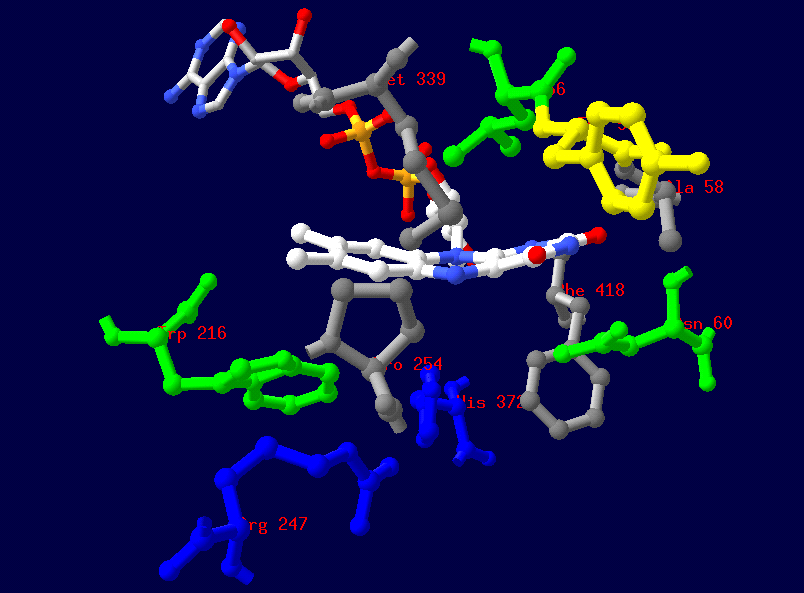

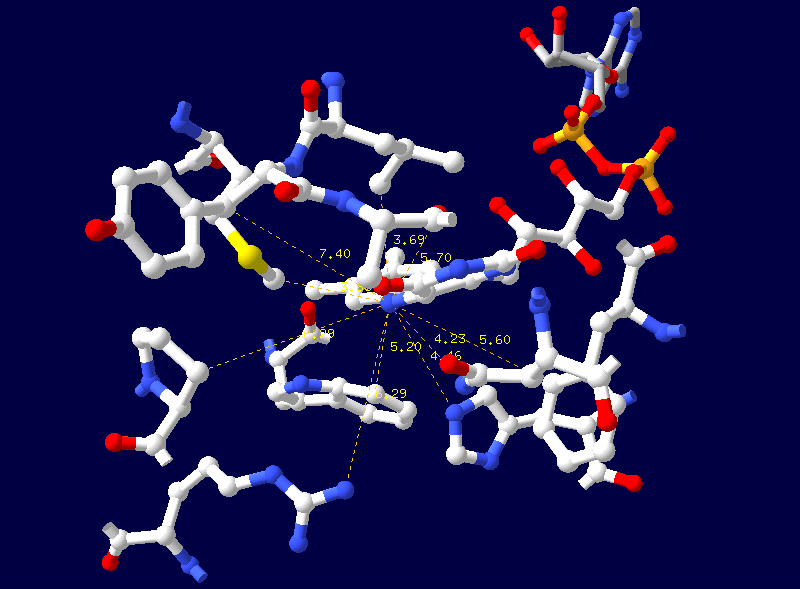
*

EBAC2C11

Crenarchaeal SRP


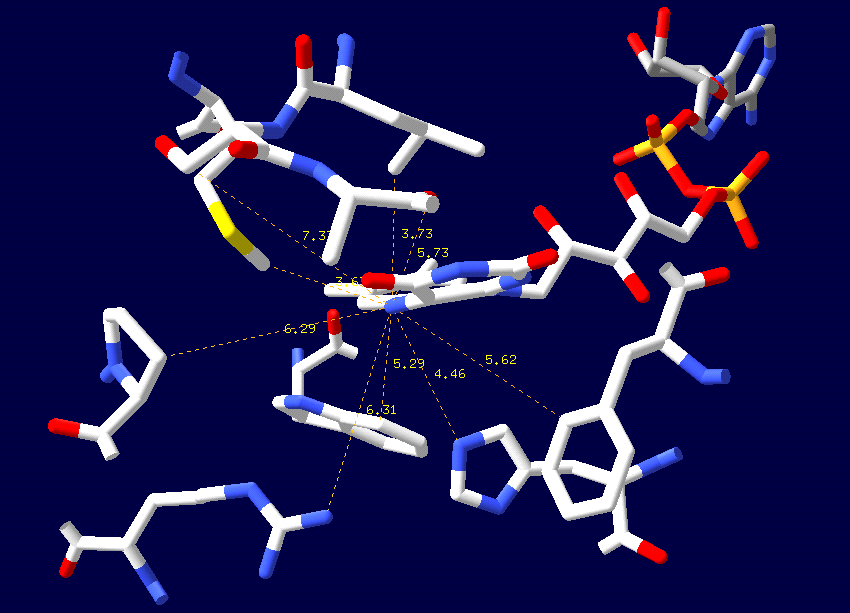

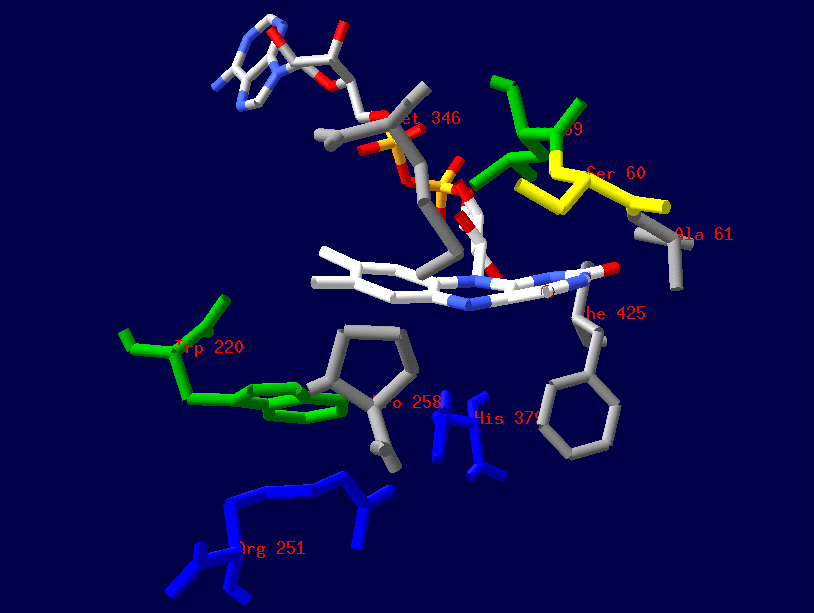


*Pyrobaculum calidifontis*


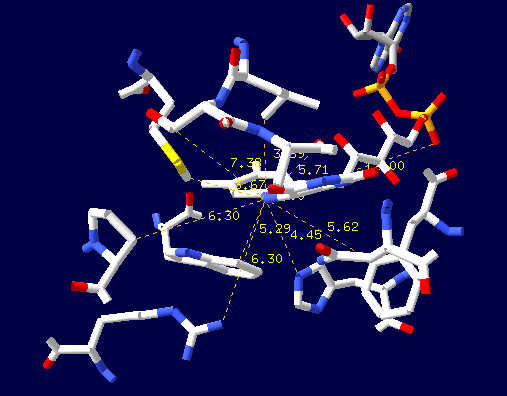

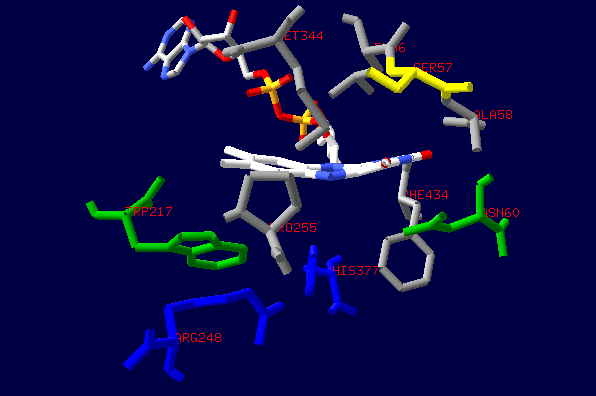


*Caldivirga maquilingensis*

SRB and related SOB Apr lineage II

*
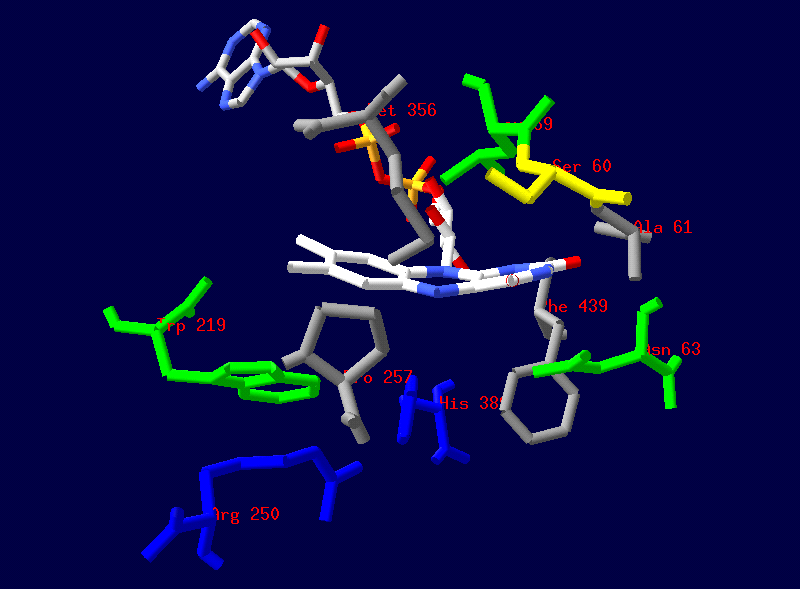

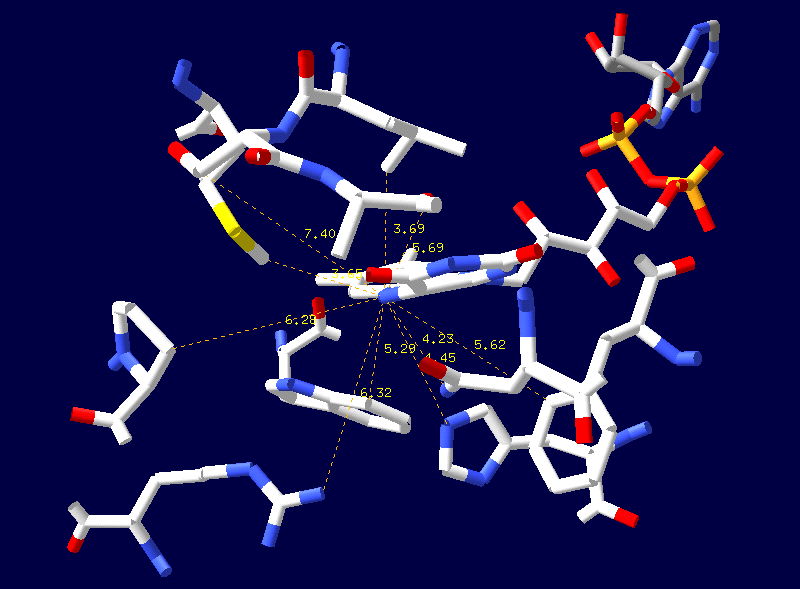
*

Gram-positive and related deltaproteobacterial SRB

*
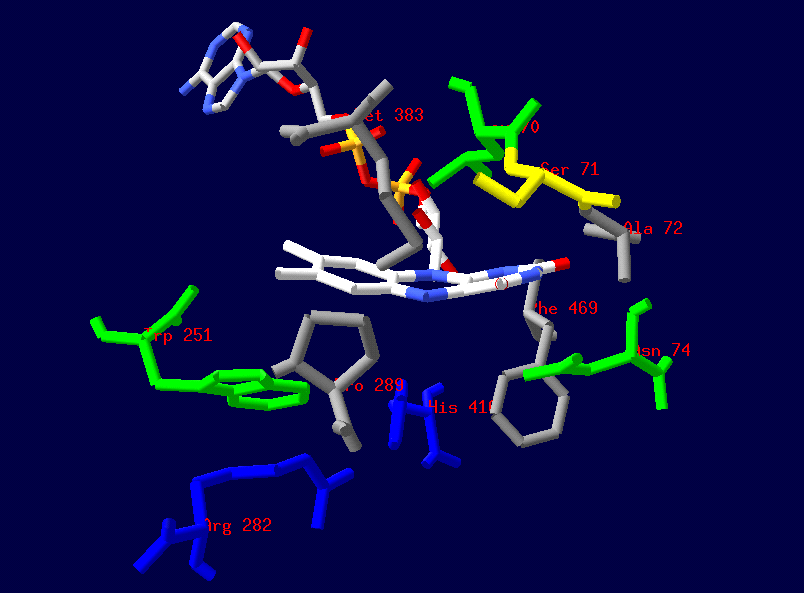

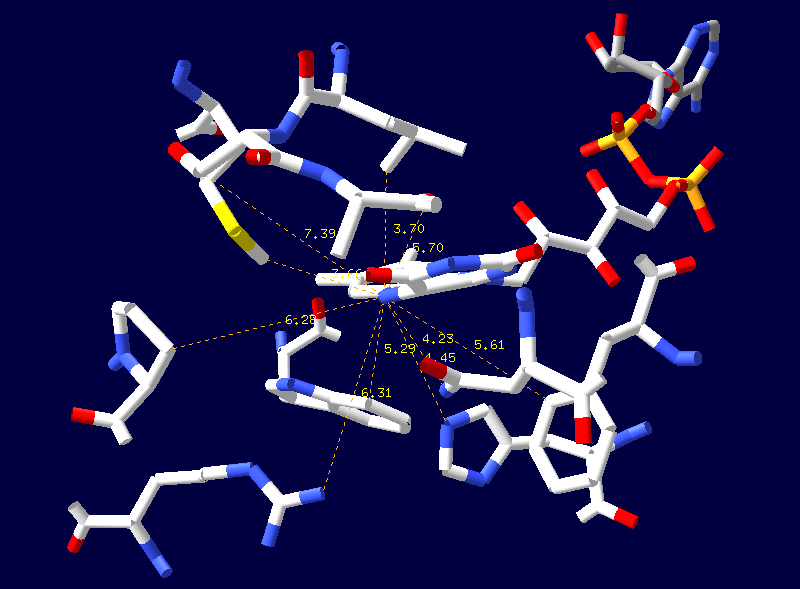
*


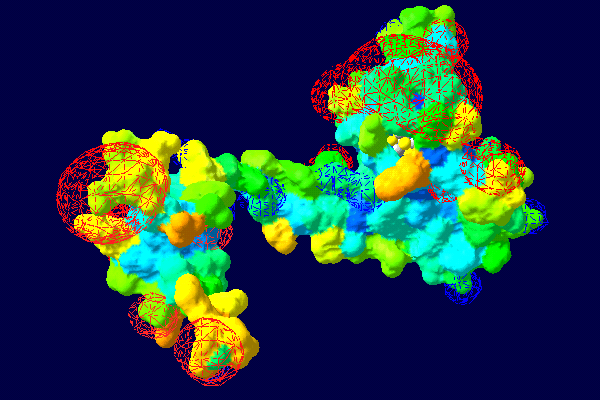

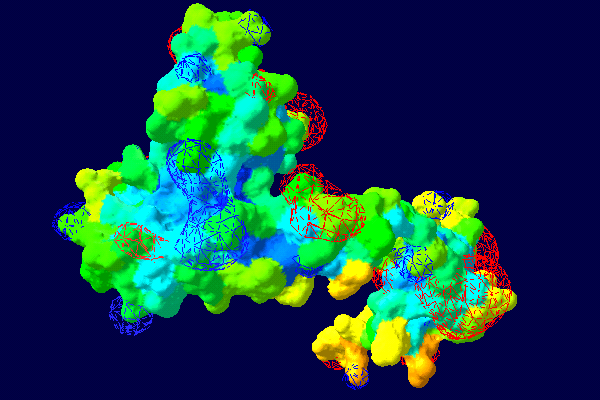
 *Thermodesulfobacterium commune*


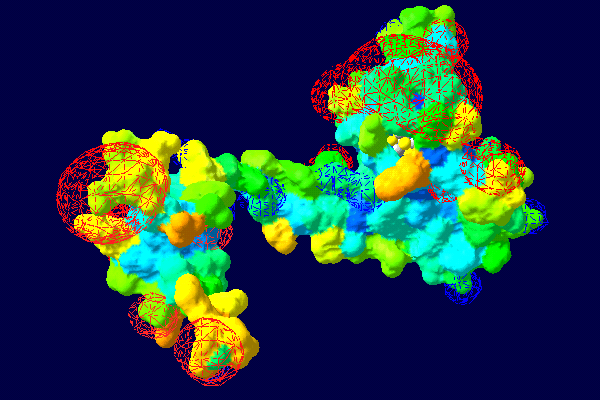

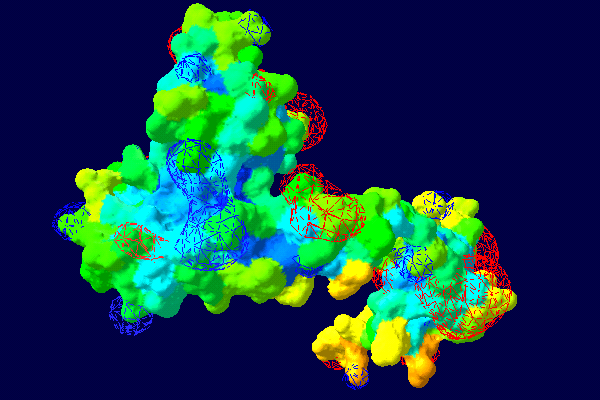

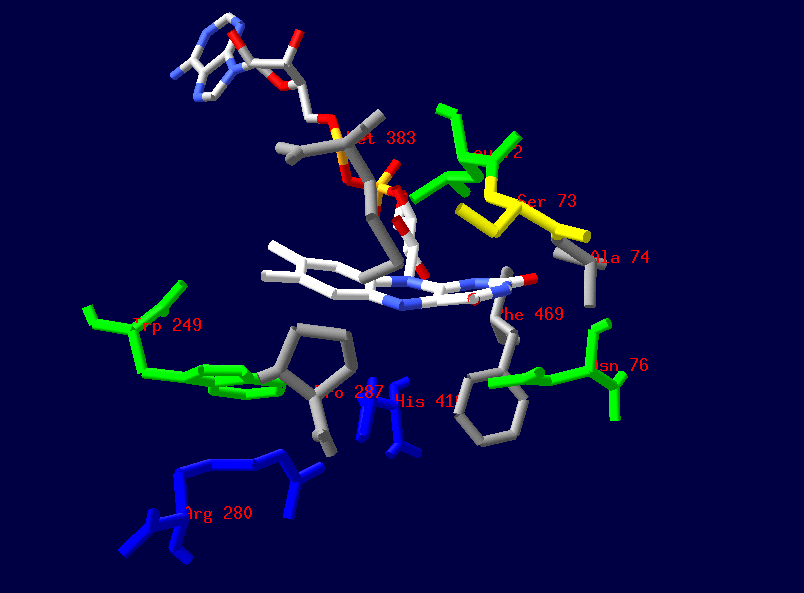

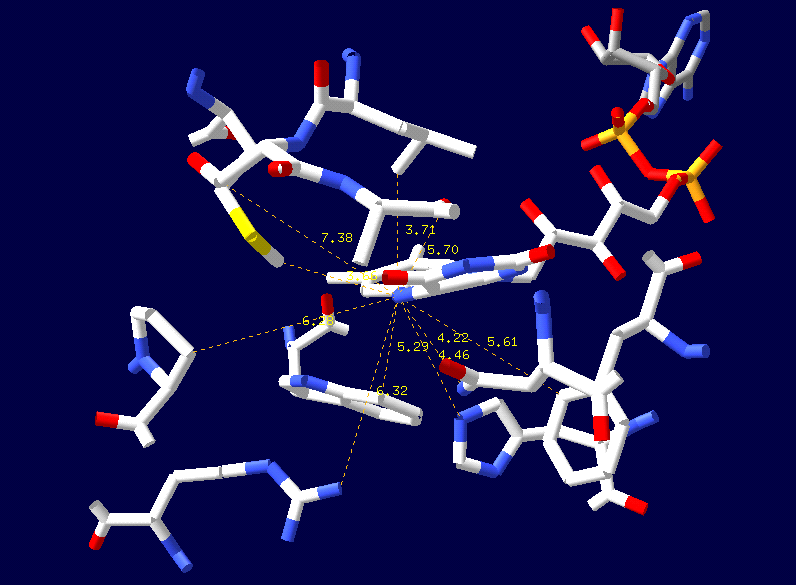


*Desulfovibrionaceae*


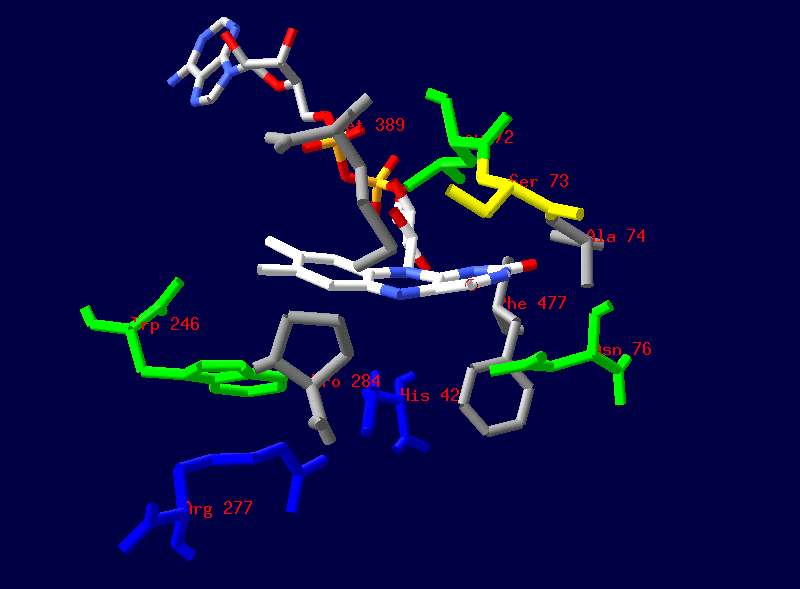

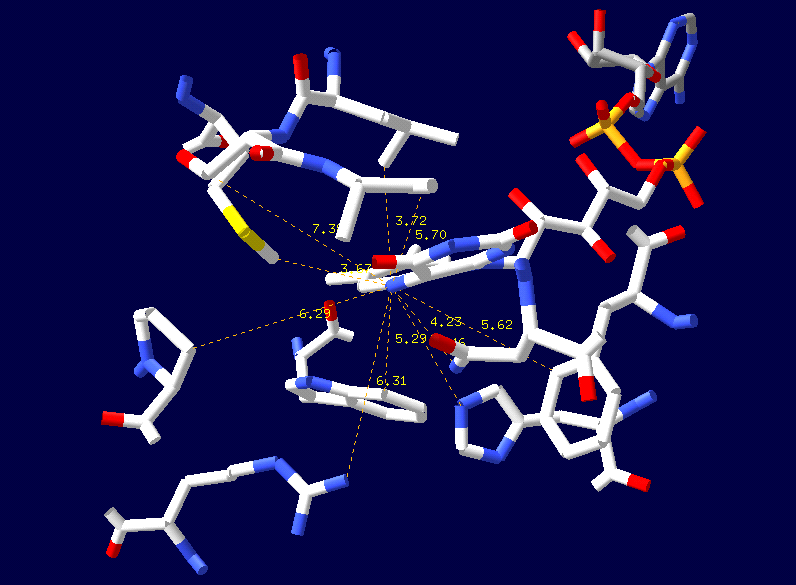


*Desulfobulbaceae* and *Desulfobacteraceae*


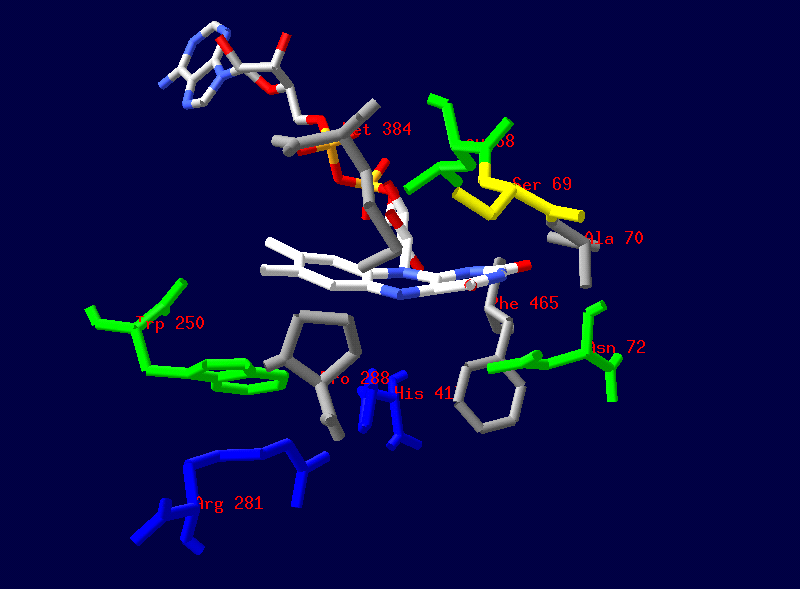

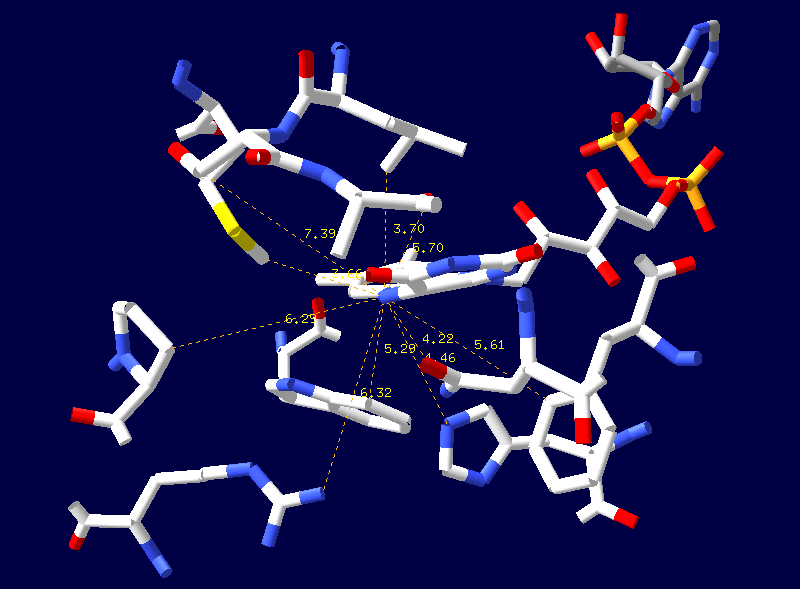


*Chlorobaculum tepidum*

*
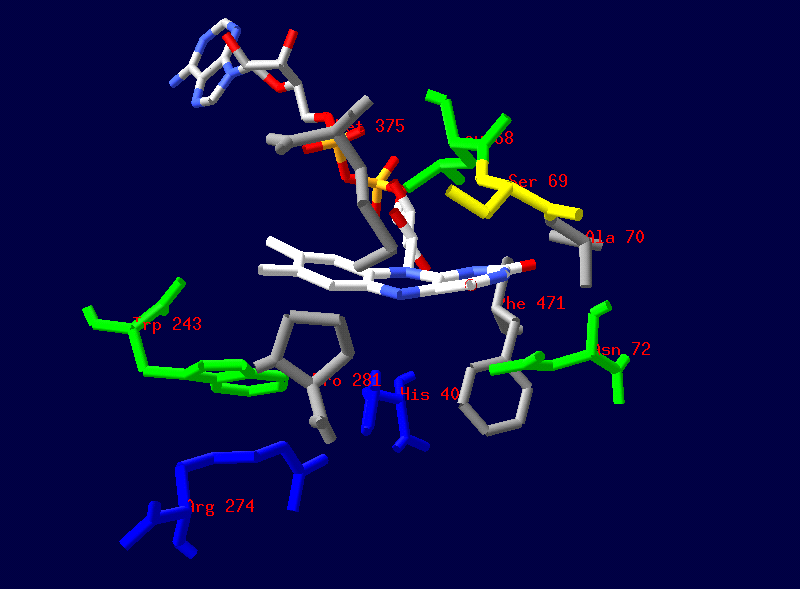

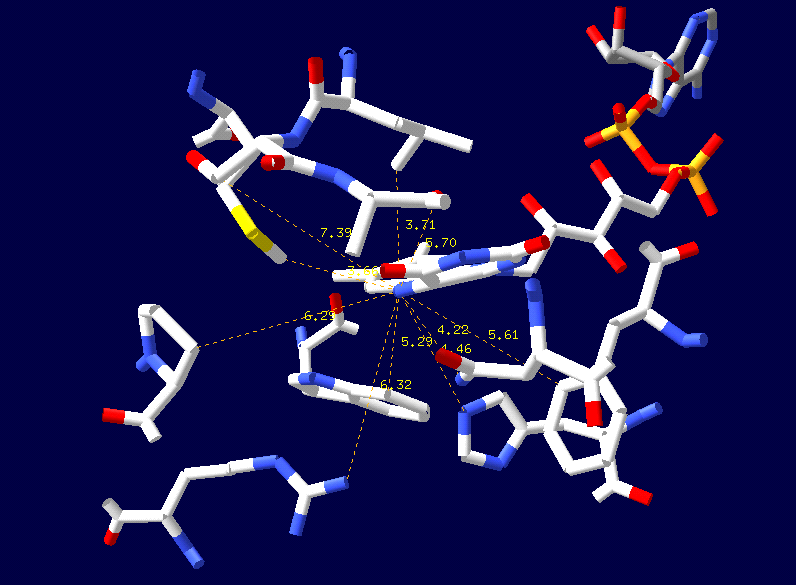
*


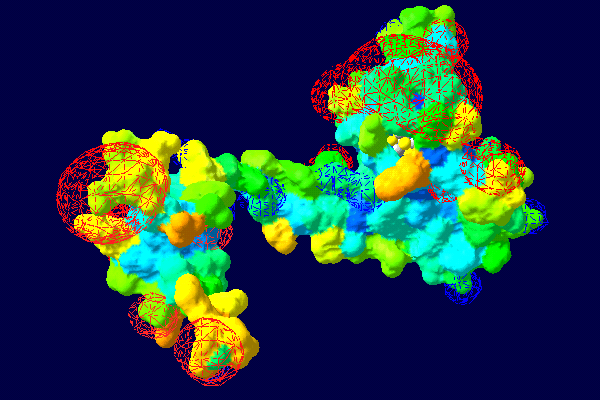

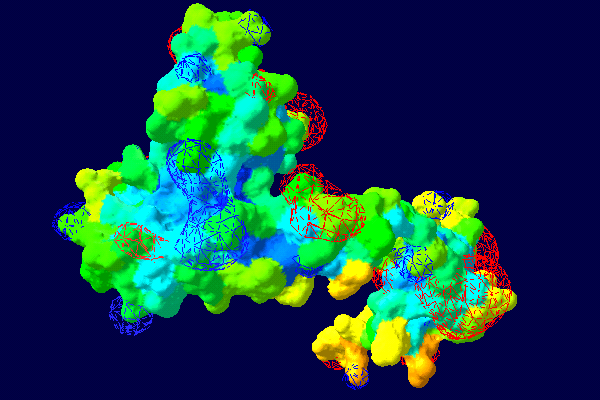
 *Thiobacillus denitrificans*


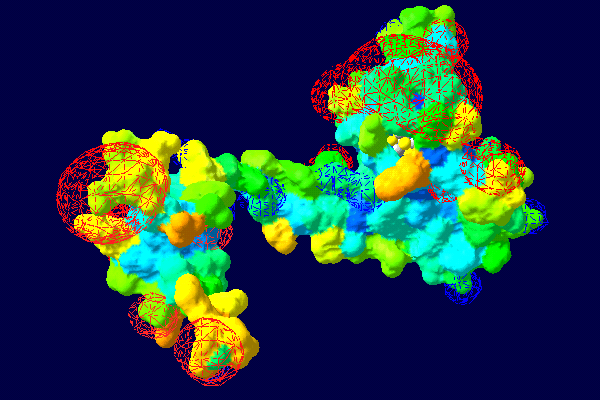

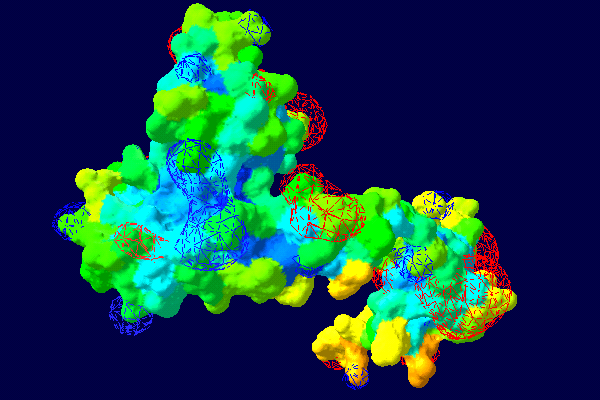

Supplement: Figure S4 — (1.17 MB DOC) [file pone.0001514.s004.doc]
