# Supplementary material for: Homology Modeling of Dissimilatory APS Reductases (AprBA) of Sulfur-Oxidizing and Sulfate-Reducing Prokaryotes
Source: PLoS One. 2008 Jan 30;3(1):e1514. doi: 10.1371/journal.pone.0001514 (PMC2211403; doi:10.1371/journal.pone.0001514)
Supplement: Table S1 — (0.76 MB DOC) [file pone.0001514.s005.doc]

#### Supplementary data material Table S1. AprB secondary structure element succession

# SOB Apr lineage I

# Secondary structure element succession AprB *Allochromatium vinosum*

| **AprB segments** | **No** | ***Archaeoglobus fulgidus* sequence** | AA position | **Sec. str. element** | ***Allochromatium vinosum* sequence** | AA position | **Sec. str. element** |
| --- | --- | --- | --- | --- | --- | --- | --- |
| Ferredoxin | 1 | P S F V N | 2-6 | beta-sheet | P T F V Y (Loop : missing) | 2-6 | beta-sheet |
| (2-68 AA) | 2 | A C E Y I | 20-24 | alpha-helix | C V D I **shortened** | 16-19 | alpha-helix |
|  | 3 | L M T L D | 29-33 | beta-sheet | I M H I D | 24-28 | beta-sheet |
|  | 4 | K A Y N R | 38-42 | beta-sheet | R A Y N I | 33-37 | beta-sheet |
|  | 5 | S C V K M | 52-56 | alpha-helix | S C V K A | 47-51 | alpha-helix |
|  | 6 | A I D V R | 61-65 | beta-sheet | A I D V R | 56-60 | beta-sheet |
| **3 antiparallel** | 7 | A C V P M R G | 76-82 | beta-sheet | S V R V R D | 71-76 | beta-sheet |
| **Beta-sheet**  (69-104 AA) | 8 | S D I M W T V K Y | 84-92 | beta-sheet | G V I A W R I I F (EK-Insert: 7 AA Loop, 5 charged AA) | 81-89 | beta-sheet |
|  | 9 | K V L R F K F A I | 96-104 | beta-sheet | K D M N L L A P I | 94-102 | beta-sheet |
| **Tail** | 10 | E E A L | 123-126 | alpha-helix | N E M R | 122-125 | alpha-helix |
| (105-148 AA) | 11 | E I | 136-137 | alpha-helix | **missing** |  |  |
|  |  |  |  |  |  |  |  |

**Structural differences:** 12-14 loop **156 AA total** **Protein problems: backbone**

77-80 loop **identical AA 67** 43,0% Pro2, Gln15, Asn132, His145

91-93 loop **similar AA** 91** 58,3% **sidechain**

112-114 loop **1.0Ǻ RMSD backbone 134** **85,9%** Gln15, Met25, Met41, Cys42, Arg76, 133-142 loop ** incl. identical AA

**Ramachandran Plot**

Gly14, Gln15, Gly12, Gly142, Gly69, Asn132, His145

**AA Accessibility**: **Trp43**, Asp64, Thr104

# Secondary structure element succession AprB *Thiobacillus denitrificans* ATCC25259

| **AprB segments** | **No** | ***Archaeoglobus fulgidus* sequence** | AA position | **Sec. str. element** | *Thiobacillus denitrificans* **sequence** | AA position | **Sec. str. element** |
| --- | --- | --- | --- | --- | --- | --- | --- |
| Ferredoxin | 1 | P S F V N | 2-6 | beta-sheet | P T F V Y (Loop: missing) | 2-6 | beta-sheet |
| (2-68 AA) | 2 | A C E Y I | 20-24 | alpha-helix | C V D I **shortened** | 16-19 | alpha-helix |
|  | 3 | L M T L D | 29-33 | beta-sheet | I M H I D | 24-28 | beta-sheet |
|  | 4 | K A Y N R | 38-42 | beta-sheet | R A Y N I | 33-37 | beta-sheet |
|  | 5 | S C V K M | 52-56 | alpha-helix | S C V K A | 47-51 | alpha-helix |
|  | 6 | A I D V R | 61-65 | beta-sheet | A I D V R | 56-60 | beta-sheet |
| **3 antiparallel** | 7 | A C V P M R G | 76-82 | beta-sheet | S V R V R R D | 71-77 | beta-sheet |
| **beta-sheet**  (69-104 AA) | 8 | S D I M W T V K Y | 84-92 | beta-sheet | G V I A W R I K F (EK-Insert: 7 AA Loop, 5 charged AA) | 81-89 | beta-sheet |
|  | 9 | K V L R F K F A I | 96-104 | beta-sheet | K D M E L L A P I | 94-102 | beta-sheet |
| **Tail** | 10 | E E A L | 123-126 | alpha-helix | Q A M R | 122-125 | alpha-helix |
| (105-148 AA) | 11 | E I | 136-137 | alpha-helix | **missing** |  |  |
|  |  |  |  |  |  |  |  |

**Structural deviations:** 12-14 loop **156 AA total** **Protein problems: backbone**

77-80 loop **identical AA 65** 41,7% Pro2, Tyr15, Lys94, Leu114, Ala115, Asp116, Asn132, Pro134, Asp143,

**similar AA** 89** 57,1% Arg138, His145

91-93 loop **1.0Ǻ RMSD backbone 133 85,3%** **sidechain**

111-114 loop** incl. identical AA Met25, Met41, Arg76, Lys109, Thr146 135-144 loop

**Ramachandran Plot**

Gly14, Tyr15, Gly69, Gly12, Leu114, Ala115, Asn132, Arg138, Asp143, His145

**AA Accessibility**: **Glu64, Trp43**

# Secondary structure element succession AprB *Candidatus* Ruthia magnifica

| **AprB segments** | **No** | ***Archaeoglobus fulgidus* sequence** | AA position | **Sec. str. element** | ***Candidatus* Ruthia magnifica sequence** | AA position | **Sec. str. element** |
| --- | --- | --- | --- | --- | --- | --- | --- |
| Ferredoxin | 1 | P S F V N | 2-6 | beta-sheet | P T F V Y (Loop : missing) | 2-6 | beta-sheet |
| (2-68 AA) | 2 | A C E Y I | 20-24 | alpha-helix | →C V D I **shortened** | 16-19 | alpha-helix |
|  | 3 | L M T L D | 29-33 | beta-sheet | I M H I D | 24-28 | beta-sheet |
|  | 4 | K A Y N R | 38-42 | beta-sheet | R A Y N I | 33-37 | beta-sheet |
|  | 5 | S C V K M | 52-56 | alpha-helix | S C V K A | 47-51 | alpha-helix |
|  | 6 | A I D V R | 61-65 | beta-sheet | A I D V R | 56-60 | beta-sheet |
| **3 antiparallel** | 7 | A C V P M R G | 76-82 | beta-sheet | S V R V I R D | 71-77 | beta-sheet |
| **beta-sheet**  (69-104 AA) | 8 | S D I M W T V K Y | 84-92 | beta-sheet | G T I A W R V K F (EK-Insert: 7 AA Loop, 5 charged AA) | 81-89 | beta-sheet |
|  | 9 | K V L R F K F A I | 96-104 | beta-sheet | R V K N F L S P I | 93-101 | beta-sheet |
| **Tail** | 10 | E E A L | 123-126 | alpha-helix | D D M R | 121-124 | alpha-helix |
| (105-148 AA) | 11 | E I | 136-137 | alpha-helix | **missing** |  |  |
|  |  |  |  |  |  |  |  |

**Abweichungen:** 12-14 loop **155 AA total** **Protein problems: backbone**

77-80 loop **identical AA 71** 45,8% Pro2, His15, His141

131-141 loop **similar AA** 96** 62,0% **sidechain**

**1.0Ǻ RMSD backbone 136 87,7%** His15, Met25, Tyr31, Met41, Cys42, Arg76, Arg114  107-112 loop ** incl. identical AA

### Ramachandran Plot

Gly12, Gly14, His15, Gly69, Gly92, Gly107, His144, Gly141

**AA Accessibility**: Lys30, **Trp43**, Asp64, Thr103

# Secondary structure element succession AprB *Pelagibacter ubique* HTCC1062

| **AprB segments** | **No** | ***Archaeoglobus fulgidus* sequence** | AA position | **Sec. str. element** | ***Pelagibacter ubique* sequence** | AA position | **Sec. str. element** |
| --- | --- | --- | --- | --- | --- | --- | --- |
| Ferredoxin | 1 | P S F V N | 2-6 | beta-sheet | S T F V Y (Loop : missing) | 2-6 | beta-sheet |
| (2-68 AA) | 2 | A C E Y I | 20-24 | alpha-helix | →C V D I **shortened** | 16-19 | alpha-helix |
|  | 3 | L M T L D | 29-33 | beta-sheet | I M H I D | 24-28 | beta-sheet |
|  | 4 | K A Y N R | 38-42 | beta-sheet | R A V N I | 33-37 | beta-sheet |
|  | 5 | S C V K M | 52-56 | alpha-helix | S C V K A | 47-51 | alpha-helix |
|  | 6 | A I D V R | 61-65 | beta-sheet | A I D V R | 56-60 | beta-sheet |
| **3 antiparallel** | 7 | A C V P M R G | 76-82 | beta-sheet | S V R V R R E | 71-77 | beta-sheet |
| **beta-sheet**  (69-104 AA) | 8 | S D I M W T V K Y | 84-92 | beta-sheet | G T I S W K I K F (EK-Insert: 7 AA Loop, 5 charged AA) | 81-89 | beta-sheet |
|  | 9 | K V L R F K F A I | 96-104 | beta-sheet | T T K D F V S P I | 93-101 | beta-sheet |
| **Tail** | 10 | E E A L | 123-126 | alpha-helix | Q G E I | 121-124 | alpha-helix |
| (105-148 AA) | 11 | E I | 136-137 | alpha-helix | **missing** |  |  |
|  |  |  |  |  |  |  |  |

**Structural deviations:** 12-14 loop **153 AA total** **Protein problems: backbone**

77-80 loop **identical AA 59** 38,6% His15, Leu113, Glu115, Thr131, Lys141, Leu142

132-141 loop **similar AA** 86** 56,2% **sidechain**

**1.0Ǻ RMSD backbone 133 87,0%** His15, Met25, Cys42, Lys108  109-113 loop ** incl. identical AA

### Ramachandran Plot

Gly12, Gly14, His15, Gly69, Leu113, Lys141, Gly92, Leu142, Glu115,

Thr131, Gly81

**AA Accessibility**: Asn30, **Trp43**, Asp64

# Secondary structure element succession AprB environmental sequence EBAC2C11

| **AprB segments** | **No** | ***Archaeoglobus fulgidus* sequence** | AA position | **Sec. str. element** | **EBAC2C11 sequence** | AA position | **Sec. str. element** |
| --- | --- | --- | --- | --- | --- | --- | --- |
| Ferredoxin | 1 | P S F V N | 2-6 | beta-sheet | P T F V Y (Loop : missing) | 2-6 | beta-sheet |
| (2-68 AA) | 2 | A C E Y I | 20-24 | alpha-helix | →C V D I **shortened** | 16-19 | alpha-helix |
|  | 3 | L M T L D | 29-33 | beta-sheet | I M H I D | 24-28 | beta-sheet |
|  | 4 | K A Y N R | 38-42 | beta-sheet | R A V N I | 33-37 | beta-sheet |
|  | 5 | S C V K M | 52-56 | alpha-helix | S C V K A | 47-51 | alpha-helix |
|  | 6 | A I D V R | 61-65 | beta-sheet | A I D A R | 56-60 | beta-sheet |
| **3 antiparallel** | 7 | A C V P M R G | 76-82 | beta-sheet | S V R V L R E | 71-77 | beta-sheet |
| **beta-sheet**  (69-104 AA) | 8 | S D I M W T V K Y | 84-92 | beta-sheet | G T I S W R L K F (DK-Insert: 7 AA Loop, 5 charged AA) | 81-89 | beta-sheet |
|  | 9 | K V L R F K F A I | 96-104 | beta-sheet | R E K H F E S P I | 93-101 | beta-sheet |
| **Tail** | 10 | E E A L | 123-126 | alpha-helix | L D D M | 120-123 | alpha-helix |
| (105-148 AA) | 11 | E I | 136-137 | alpha-helix | **missing** |  |  |
|  |  |  |  |  |  |  |  |

**Structural deviations:** 12-14 loop **153 AA** **Protein problems: backbone**

77-80 loop **identical AA 61** 40,0% Pro2, His15, His130, Leu141, Pro142

131-140 loop **similar AA** 90** 58,8% **sidechain**

**1.0Ǻ RMSD backbone 137 89,5%** His15, Met25, Cys42, Arg76, Lys110, Tyr115  ** incl. identical AA

### Ramachandran Plot

Gly12, Gly14, His15, Gly69, Gly81, Gly92, His130, Leu141, Ser143

# AA Accessibility: Trp43, Asp64, Thr103

# Crenarchaeal SRP

# Secondary structure element succession AprB *Pyrobaculum aerophilum*

| **AprB segments** | **No** | ***Archaeoglobus fulgidus* sequence** | AA position | **Sec. str. element** | *Pyrobaculum aerophilum* **sequence** | AA position | **Sec. str. element** |
| --- | --- | --- | --- | --- | --- | --- | --- |
| Ferredoxin | 1 | P S F V N | 2-6 | beta-sheet | P T F V Y (Loop : missing) | 2-6 | beta-sheet |
| (2-68 AA) | 2 | A C E Y I | 20-24 | alpha-helix | C V D I **shortened** | 16-19 | alpha-helix |
|  | 3 | L M T L D | 29-33 | beta-sheet | N M Q Y N | 24-28 | beta-sheet |
|  | 4 | K A Y N R | 38-42 | beta-sheet | K G Y N A | 33-37 | beta-sheet |
|  | 5 | S C V K M | 52-56 | alpha-helix | N C V K Y | 47-51 | alpha-helix |
|  | 6 | A I D V R | 61-65 | beta-sheet | A V D V R | 56-60 | beta-sheet |
| **3 antiparallel** | 7 | A C V P M R G | 76-82 | beta-sheet | E M V R D T K | 71-77 | beta-sheet |
| **beta-sheet**  (69-104 AA) | 8 | S D I M W T V K Y | 84-92 | beta-sheet | N I I Y W R I V Y | 79-87 | beta-sheet |
|  | 9 | K V L R F K F A I | 96-104 | beta-sheet | T V Y D F A S P I | 91-99 | beta-sheet |
| **Tail** | 10 | E E A L | 123-126 | alpha-helix | R Q D L | 116-119 | alpha-helix |
| (105-148 AA) | 11 | E I | 136-137 | alpha-helix | Insert: K D E | 129-131 | **beta-sheet** |
|  |  |  |  |  | **Insert: G F P** | 134-136 | **beta-sheet** |

**Structural deviations:** 12-14 loop **150 AA total** **Protein problems: backbone**

106-110 loop **identical AA 58** 38,7% Pro2, Lys15, Lys77, Ala110, Ala125, Pro130, Pro136, Arg140

128-129 loop-beta **similar AA** 81** 54,0% **sidechain**

132-137 loop-beta-loop **1.0Ǻ RMSD backbone 129 86,0%** Lys15, Met25, Tyr87 ** incl. identical AA

**Ramachandran Plot**

Gly14, Gly12, Ala110, Lys77, Lys15, Gly69, Gly90, Arg140, Ala125

**AA Accessibility**: **Ile40**, Lys30, Asp64, Thr101

# Secondary structure element succession AprB *Pyrobaculum calidifontis*

| **AprB segments** | **No** | ***Archaeoglobus fulgidus* sequence** | AA position | **Sec. str. element** | *Pyrobaculum calidifontis* **sequence** | AA position | **Sec. str. element** |
| --- | --- | --- | --- | --- | --- | --- | --- |
| Ferredoxin | 1 | P S F V N | 2-6 | beta-sheet | P T F V Y (Loop : missing) | 2-6 | beta-sheet |
| (2-68 AA) | 2 | A C E Y I | 20-24 | alpha-helix | C V D I **shortened** | 16-19 | alpha-helix |
|  | 3 | L M T L D | 29-33 | beta-sheet | N M H Y N | 24-28 | beta-sheet |
|  | 4 | K A Y N R | 38-42 | beta-sheet | K A Y N A | 33-37 | beta-sheet |
|  | 5 | S C V K M | 52-56 | alpha-helix | N C V K Y | 47-51 | alpha-helix |
|  | 6 | A I D V R | 61-65 | beta-sheet | A V E V R | 56-60 | beta-sheet |
| **3 antiparallel** | 7 | A C V P M R G | 76-82 | beta-sheet | R I G V V R D | 71-77 | beta-sheet |
| **beta-sheet**  (69-104 AA) | 8 | S D I M W T V K Y | 84-92 | beta-sheet | N I I Y W R V M Y | 81-89 | beta-sheet |
|  | 9 | K V L R F K F A I | 96-104 | beta-sheet | T V Y E F A S P I | 93-101 | beta-sheet |
| **Tail** | 10 | E E A L | 123-126 | alpha-helix | R P D L | 118-121 | alpha-helix |
| (105-148 AA) | 11 | E I | 136-137 | alpha-helix | Insert: N P E | 131-133 | **beta-sheet** |
|  |  |  |  |  | **Insert: G F K** | 136-138 | **beta-sheet** |

**Structural deviations:** 12-14 loop **151 AA total** **Protein problems: backbone**

108-112 loop **identical AA 64** 42,4% Pro2, Lys15, Asn81, Ala112, Glu139

130-131 loop-beta **similar AA** 86** 57,0% **sidechain**

134-148 loop-beta-loop **1.0Ǻ RMS backbone 119 78,8%** Lys15, Met25, Cys42, Arg76, Tyr8**9**  77-80 loop ** incl. identical AA

**Ramachandran Plot**

Gly14, Gly12, Ala112, Glu139, Lys15, Gly69, Gly92

**AA Accessibility**: Lys30, Asp64, Thr103

# Secondary structure element succession AprB *Caldivirga maquilingensis*

| **AprB segments** | **No** | ***Archaeoglobus fulgidus* sequence** | AA position | **Sec. str. element** | *Caldivirga maquilingensis* **sequence** | AA position | **Sec. str. element** |
| --- | --- | --- | --- | --- | --- | --- | --- |
| Ferredoxin | 1 | P S F V N | 2-6 | beta-sheet | P S Y V I (Loop : missing) | 2-6 | beta-sheet |
| (2-68 AA) | 2 | A C E Y I | 20-24 | alpha-helix | C V N I **shortened** | 16-19 | alpha-helix |
|  | 3 | L M T L D | 29-33 | beta-sheet | I M R F T K S **elongated** (3 AA Insert) | 24-30 | beta-sheet |
|  |  |  |  |  | Insert: V L G new | 32-34 | **beta-sheet** |
|  | 4 | K A Y N R | 38-42 | beta-sheet | K A V N I | 36-40 | beta-sheet |
|  | 5 | S C V K M | 52-56 | alpha-helix | N C V K H | 50-54 | alpha-helix |
| **3 antiparallel** | 6 | A I D V R | 61-65 | beta-sheet | A V Q I R | 59-63 | beta-sheet |
| **beta-sheet**  (69-104 AA) | 7 | A C V P M R G | 76-82 | beta-sheet | S V E V Y R D | 74-80 | beta-sheet |
|  | 8 | S D I M W T V K Y | 84-92 | beta-sheet | N R V Y W T R I Y | 84-92 | beta-sheet |
|  | 9 | K V L R F K F A I | 96-104 | beta-sheet | S V K H F V F P I | 96-104 | beta-sheet |
| **Tail** | 10 | E E A L | 123-126 | alpha-helix | R E L L | 123-126 | alpha-helix |
| (105-148 AA) | 11 | E I | 136-137 | alpha-helix | missing |  |  |
|  |  |  |  |  |  |  |  |

**Structural deviations:** 12-14 loop **154 AA total** **Protein problems: backbone**

29-33 loop-beta-loop **identical AA 70** 45,5% Pro2, Asp15, Asn84, Pro115, Pro122, Phe133

80-85 loop **similar AA** 95** 61,7% **sidechain**

134-145 loop **1.0Ǻ RMSD backbone 130 84,4%** Met25, Ser44, Cys45, Arg79, Tyr92, Lys113, Tyr118, Tyr130** incl. identical AA

**Ramachandran Plot**

Gly14, Gly12, Gly31, Asp15, Gly72, Gly95, Phe133, Asn84

Gly144

**AA Accessibility**: Gly31, **Trp46**, Asp67, Thr106

# SRB and affiliated SOB Apr lineage II

# Secondary structure element succession AprB *Desulfotomaculum reducens*

| **AprB segments** | **No** | ***Archaeoglobus fulgidus* sequence** | AA position | **Sec. str. element** | ***Desulfotomaculum reducens* sequence** | AA position | **Sec. str. element** |
| --- | --- | --- | --- | --- | --- | --- | --- |
| Ferredoxin | 1 | P S F V N | 2-6 | beta-sheet | P S F V I (Loop: KGQDK) | 2-6 | beta-sheet |
| (2-68 AA) | 2 | A C E Y I | 20-24 | alpha-helix | A C M Y I | 20-24 | alpha-helix |
|  | 3 | L M T L D | 29-33 | beta-sheet | L M V L D | 29-33 | beta-sheet |
|  | 4 | K A Y N R | 38-42 | beta-sheet | K A Y N R | 38-42 | beta-sheet |
|  | 5 | S C V K M | 52-56 | alpha-helix | C C V K I | 52-56 | alpha-helix |
|  | 6 | A I D V R | 61-65 | beta-sheet | A I D V R | 61-65 | beta-sheet |
| **3 antiparallel** | 7 | A C V P M RG | 76-82 | beta-sheet | S C V P L R S | 76-82 | beta-sheet |
| **beta-sheet** | 8 | S D I M W T V K Y | 84-92 | beta-sheet | D S I M W T V K F | 84-92 | beta-sheet |
| (69-104 AA) | 9 | K V L R F K F A I | 96-104 | beta-sheet | M L K R F K F P I | 96-104 | beta-sheet |
| **Tail** | 10 | E E A L | 123-126 | alpha-helix | **missing** |  |  |
| (105-148 AA) | 11 | E I | 136-137 | alpha-helix | **missing** |  |  |
|  |  |  |  |  |  |  |  |

**Structural deviations:** 123-125 loop **147 AA total** **Protein problems: backbone**

135-136 loop **identical AA 91** 61,9% Pro2, Lys14, Pro79, Ser82, Asn126, Thr132 Pro134, Pro143

**similar AA** 115** 78,2% **sidechain**

**1.0Ǻ RMSD backbone 135 91,8%** Lys18, Thr19, Met22, Met30, Lys36, Gln46, Cys47, Tyr118, Thr137 ** incl. identical AA

**Ramachandran Plot**

Lys14, Ser82, Asn126, Gly74, Gly95, Thr132

**AA Accessibility**: **Trp48, Glu8, Asp17**, Asp69, Thr106

# Secondary structure element succession AprB *Synthrophobacter fumaroxidans*

| **AprB segments** | **No** | ***Archaeoglobus fulgidus* sequence** | AA position | **Sec. str. element** | *Synthrophobacter fumaroxidans* **sequence** | AA position | **Sec. str. element** |
| --- | --- | --- | --- | --- | --- | --- | --- |
| Ferredoxin | 1 | P S F V N | 2-6 | beta-sheet | P S Y V L (Loop: KGQDK) | 2-6 | beta-sheet |
| (2-68 AA) | 2 | A C E Y I | 20-24 | alpha-helix | A C M Y I | 20-24 | alpha-helix |
|  | 3 | L M T L D | 29-33 | beta-sheet | L M V L D | 29-33 | beta-sheet |
|  | 4 | K A Y N R | 38-42 | beta-sheet | K A F N R | 38-42 | beta-sheet |
|  | 5 | S C V K M | 52-56 | alpha-helix | N C V K I | 52-56 | alpha-helix |
|  | 6 | A I D V R | 61-65 | beta-sheet | A I D V R | 61-65 | beta-sheet |
| **3 antiparallel** | 7 | A C V P M RG | 76-82 | beta-sheet | S V V P L R G | 76-82 | beta-sheet |
| **beta-sheet** | 8 | S D I M W T V K Y | 84-92 | beta-sheet | E D I M W T V K F | 84-92 | beta-sheet |
| (69-104 AA) | 9 | K V L R F K F A I | 96-104 | beta-sheet | M V K R F K F P I | 96-104 | beta-sheet |
| **Tail** | 10 | E E A L | 123-126 | alpha-helix | **missing** |  |  |
| (105-148 AA) | 11 | E I | 136-137 | alpha-helix | A S | 135-136 | alpha-helix |
|  |  |  |  |  |  |  |  |

**Structural deviations:** 123-125 loop **147 AA total** **Protein problems: backbone**

**identical AA 92** 62,6% Pro2, Lys14, Pro79, Thr132, Trp138

**similar AA** 120** 81,6% **sidechain**

**1.0Ǻ RMSD backbone 140 95,2%** Lys18, Thr19, Met22, Met30, Lys36, Met46, Cys47, Arg81, Thr122, Ser127, ** incl. identical AA Thr132, Ser136

**Ramachandran Plot**

Lys14, Gly82, Thr132, Gly74, Gly95, Trp138

# AA Accessibility: Trp48, Glu8, Asp17, Asp69, Thr106

# Secondary structure element succession AprB fosws39f7 (M. Mussmann)

| **AprB segments** | **No** | ***Archaeoglobus fulgidus* sequence** | AA position | **Sec. str. element** | fosws39f7 sequence | AA position | **Sec. str. element** |
| --- | --- | --- | --- | --- | --- | --- | --- |
| Ferredoxin | 1 | P S F V N | 2-6 | beta-sheet | P S F V L (Loop: KALDK) | 2-6 | beta-sheet |
| (2-68 AA) | 2 | A C E Y I | 20-24 | alpha-helix | A C Q Y V | 20-24 | alpha-helix |
|  | 3 | L M T L D | 29-33 | beta-sheet | L M V L D | 29-33 | beta-sheet |
|  | 4 | K A Y N R | 38-42 | beta-sheet | M A F N R | 38-42 | beta-sheet |
|  | 5 | S C V K M | 52-56 | alpha-helix | S C V K I | 52-56 | alpha-helix |
|  | 6 | A I D V R | 61-65 | beta-sheet | A I E V R | 61-65 | beta-sheet |
| **3 antiparallel** | 7 | A C V P M RG | 76-82 | beta-sheet | T V T P L R A | 76-82 | beta-sheet |
| **beta-sheet** | 8 | S D I M W T V K Y | 84-92 | beta-sheet | D S I M W T V K F | 84-92 | beta-sheet |
| (69-104 AA) | 9 | K V L R F K F A I | 96-104 | beta-sheet | M L K R F K F P I | 96-104 | beta-sheet |
| **Tail** | 10 | E E A L | 123-126 | alpha-helix | **missing** |  |  |
| (105-148 AA) | 11 | E I | 136-137 | alpha-helix | E A | 135-136 | alpha-helix |
|  |  |  |  |  |  |  |  |

**Structural deviations:** 121-123 loop **146 AA total** **Protein problems: backbone**

**identical AA 87** 59,6% Pro2, Lys14, Pro17, Ala82, Pro114, Glu132, Arg138

**similar AA** 115** 78,8% **sidechain**

**1.0Ǻ RMSD backbone 145 99,3%** Lys18, Thr19, Glu22, Met30, Lys36, Met46, Arg81, Lys111, Asp124, Leu125 ** incl. identical AA

**Ramachandran Plot**

Lys14, Ala82, Leu125, Gly74, Gly95, Arg138

**AA Accessibility**: **Trp48, Asp8, Asp17**, Phe70, Thr106

# Secondary structure element succession AprB fosws7f8

| **AprB segments** | **No** | ***Archaeoglobus fulgidus* sequence** | AA position | **Sec. str. element** | fosws7f8 sequence | AA position | **Sec. str. element** |
| --- | --- | --- | --- | --- | --- | --- | --- |
| Ferredoxin | 1 | P S F V N | 2-6 | beta-sheet | P S Y V I (Loop: KALDK) | 2-6 | beta-sheet |
| (2-68 AA) | 2 | A C E Y I | 20-24 | alpha-helix | A C Q Y V | 20-24 | alpha-helix |
|  | 3 | L M T L D | 29-33 | beta-sheet | L M V L D | 29-33 | beta-sheet |
|  | 4 | K A Y N R | 38-42 | beta-sheet | K A F N Q | 38-42 | beta-sheet |
|  | 5 | S C V K M | 52-56 | alpha-helix | C C V K I | 52-56 | alpha-helix |
|  | 6 | A I D V R | 61-65 | beta-sheet | A I E V R | 61-65 | beta-sheet |
| **3 antiparallel** | 7 | A C V P M RG | 76-82 | beta-sheet | L V T P L R A | 76-82 | beta-sheet |
| **beta-sheet** | 8 | S D I M W T V K Y | 84-92 | beta-sheet | D S I M W T L K F | 84-92 | beta-sheet |
| (69-104 AA) | 9 | K V L R F K F A I | 96-104 | beta-sheet | M L K R F K F P I | 96-104 | beta-sheet |
| **Tail** | 10 | E E A L | 123-126 | alpha-helix | **missing** |  |  |
| (105-148 AA) | 11 | E I | 136-137 | alpha-helix | A G | 135-136 | alpha-helix |
|  |  |  |  |  |  |  |  |

**Structural deviations:** 122-125 loop **149 AA total** **Protein problems: backbone**

**identical AA 85** 57,0% Pro2, Lys14, Pro79, Ala82, Asp124, Ser132, Pro134, Asn138

**similar AA** 111** 74,5% **sidechain**

**1.0Ǻ RMSD backbone 145 97,3%** Arg18, Thr19, Gln22, Met30, Met46, Cys47 ** incl. identical AA

**Ramachandran Plot**

Lys14, Ala82, Asp124, Gly74, Gly95, Asn138, Ser132

**AA Accessibility**: **Trp48, Glu8, Asp17**, Asp69, Thr106

# Secondary structure element succession AprB *Thermodesulfobacterium commune*

| **AprB segments** | **No** | ***Archaeoglobus fulgidus* sequence** | AA position | **Sec. str. element** | *Thermodesulfobacterium commune* sequence | AA position | **Sec. str. element** |
| --- | --- | --- | --- | --- | --- | --- | --- |
| Ferredoxin | 1 | P S F V N | 2-6 | beta-sheet | P S Y V N (Loop: KGGDR) | 2-6 | beta-sheet |
| (2-68 AA) | 2 | A C E Y I | 20-24 | alpha-helix | V C M Y I | 20-24 | alpha-helix |
|  | 3 | L M T L D | 29-33 | beta-sheet | L M I L D | 29-33 | beta-sheet |
|  | 4 | K A Y N R | 38-42 | beta-sheet | K A Y N Q | 38-42 | beta-sheet |
|  | 5 | S C V K M | 52-56 | alpha-helix | S C V K S | 52-56 | alpha-helix |
|  | 6 | A I D V R | 61-65 | beta-sheet | A I A I R | 61-65 | beta-sheet |
| **3 antiparallel** | 7 | A C V P M R G | 76-82 | beta-sheet | T C Q P M R G | 76-82 | beta-sheet |
| **beta-sheet** | 8 | S D I M W T V K Y | 84-92 | beta-sheet | T D I M W T I K F | 84-92 | beta-sheet |
| (69-104 AA) | 9 | K V L R F K F A I | 96-104 | beta-sheet | Y V L R F K F P I | 96-104 | beta-sheet |
| **Tail** | 10 | E E A L | 123-126 | alpha-helix | P S K L | 123-126 | alpha-helix |
| (105-148 AA) | 11 | E I | 136-137 | alpha-helix | A D | 136-137 | alpha-helix |
|  |  |  |  |  |  |  |  |

**Structural deviations:** 144-147 loop **152 AA total** **Protein problems: backbone**

**identical AA 92** 60,5% Pro2, Lys14, His74, Pro79, Thr133, Ile139, Leu148

**similar AA** 113** 74,3% **sidechain**

**1.0Ǻ RMSD backbone 148 97,4%** Arg18, Thr19, Met22, Met30, Gln46, Cys47, His66, His 74, Arg81, Lys118 ** incl. identical AA Thr133, Asp137

**Ramachandran Plot**

Gly82, Lys14, His74, Glu142, Gly95, Ile139, Thr133, Leu148

**AA Accessibility**: **Glu8**, **Trp48, Asp17**, Glu35, Lys14, Asp69, Thr106

# Secondary structure element succession AprB *Desulfovibrio desulfuricans*

| **AprB segments** | **No** | ***Archaeoglobus fulgidus* sequence** | AA position | **Sec. str. element** | *Desulfovibrio desulfuricans* sequence | AA position | **Sec. str. element** |
| --- | --- | --- | --- | --- | --- | --- | --- |
| Ferredoxin | 1 | P S F V N | 2-6 | beta-sheet | P T F V D (Loop: KGGEK) | 2-6 | beta-sheet |
| (2-68 AA) | 2 | A C E Y I | 20-24 | alpha-helix | A C M Y I | 20-24 | alpha-helix |
|  | 3 | L M T L D | 29-33 | beta-sheet | L M I L D | 29-33 | beta-sheet |
|  | 4 | K A Y N R | 38-42 | beta-sheet | K A Y N Q | 38-42 | beta-sheet |
|  | 5 | S C V K M | 52-56 | alpha-helix | S C V K I | 52-56 | alpha-helix |
|  | 6 | A I D V R | 61-65 | beta-sheet | A I T A R | 61-65 | beta-sheet |
| **3 antiparallel** | 7 | A C V P M R G | 76-82 | beta-sheet | T C I P M R S | 76-82 | beta-sheet |
| **beta-sheet** | 8 | S D I M W T V K Y | 84-92 | beta-sheet | D S I M W T V K F | 84-92 | beta-sheet |
| (69-104 AA) | 9 | K V L R F K F A I | 96-104 | beta-sheet | N V K R F K F P I | 96-104 | beta-sheet |
| **Tail** | 10 | E E A L | 123-126 | alpha-helix | **missing** |  |  |
| (105-148 AA) | 11 | E I | 136-137 | alpha-helix | A L | 134-135 | alpha-helix |
|  |  |  |  |  |  |  |  |

**Structural deviations:** 119-120 loop **148 AA total** **Protein problems: backbone**

**identical AA 91** 61,5% Pro2, Lys14, Pro79, Ser82, Ala122, Asn123, Thr131, Ala137

**similar AA** 111** 75,0% **sidechain**

**1.0Ǻ RMSD backbone 145 98,0%** Lys18, Thr19, Met22, Met30, Cys47, Lys113, Thr131, Thr133 ** incl. identical AA

**Ramachandran Plot**

Ser82, Lys14, Asn123, Gly74, Gly95, Ala137, Thr131, Ala122

**AA Accessibility**: **Trp48, Lys14, Glu17**, Asp69, Thr106

# Secondary structure element succession AprB *Desulfovibrio vulgaris*

| **AprB segments** | **No** | ***Archaeoglobus fulgidus* sequence** | AA position | **Sec. str. element** | *Desulfovibrio vulgaris* sequence | AA position | **Sec. str. element** |
| --- | --- | --- | --- | --- | --- | --- | --- |
| Ferredoxin | 1 | P S F V N | 2-6 | beta-sheet | P T Y V D (Loop: KGGEK) | 2-6 | beta-sheet |
| (2-68 AA) | 2 | A C E Y I | 20-24 | alpha-helix | A C M Y I | 20-24 | alpha-helix |
|  | 3 | L M T L D | 29-33 | beta-sheet | L M I L D | 29-33 | beta-sheet |
|  | 4 | K A Y N R | 38-42 | beta-sheet | R A Y N Q | 38-42 | beta-sheet |
|  | 5 | S C V K M | 52-56 | alpha-helix | S C V K I | 52-56 | alpha-helix |
|  | 6 | A I D V R | 61-65 | beta-sheet | A I T A R | 61-65 | beta-sheet |
| **3 antiparallel** | 7 | A C V P M R G | 76-82 | beta-sheet | T C I P M R S | 76-82 | beta-sheet |
| **beta-sheet** | 8 | S D I M W T V K Y | 84-92 | beta-sheet | D S I M W T V K F | 84-92 | beta-sheet |
| (69-104 AA) | 9 | K V L R F K F A I | 96-104 | beta-sheet | N V K R F K F P I | 96-104 | beta-sheet |
| **Tail** | 10 | E E A L | 123-126 | alpha-helix | **missing** |  |  |
| (105-148 AA) | 11 | E I | 136-137 | alpha-helix | A L | 134-135 | alpha-helix |
|  |  |  |  |  |  |  |  |

**Structural deviations:** 119-120 loop **148 AA total** **Protein problem: backbone**

**identical AA 91** 61,5% Pro2, Lys14, Pro79, Ser82, Asp123, Thr131, Ala137

**similar AA** 113** 76,4% **sidechain**

**1.0Ǻ RMSD backbone 145 98,0%** Lys18, Thr19, Met22, Met30, Cys47, Lys113, Lys118, Thr131, Thr133 ** incl. identical AA Thr136

**Ramachandran Plot**

Ser82, Lys14, Asp123, Gly74, Gly95, Ala137, Thr131, Ala122

**AA Accessibility**: **Trp48, Lys14, Glu17**, Asp69, Thr106

# Secondary structure element succession AprB *Desulfobulbus* sp. str. MLMS-1

| **AprB segments** | **No** | ***Archaeoglobus fulgidus* sequence** | AA position | **Sec. str. element** | *Desulfobulbus* sp. sequence | AA position | **Sec. str. element** |
| --- | --- | --- | --- | --- | --- | --- | --- |
| Ferredoxin | 1 | P S F V N | 2-6 | beta-sheet | P S Y V E (Loop: KGGDK) | 2-6 | beta-sheet |
| (2-68 AA) | 2 | A C E Y I | 20-24 | alpha-helix | A C M Y I | 20-24 | alpha-helix |
|  | 3 | L M T L D | 29-33 | beta-sheet | L M V L N | 29-33 | beta-sheet |
|  | 4 | K A Y N R | 38-42 | beta-sheet | K A Y N Q | 38-42 | beta-sheet |
|  | 5 | S C V K M | 52-56 | alpha-helix | S C V K I | 52-56 | alpha-helix |
|  | 6 | A I D V R | 61-65 | beta-sheet | A I A V R | 61-65 | beta-sheet |
| **3 antiparallel** | 7 | A C V P M R G | 76-82 | beta-sheet | V V H P M R S | 76-82 | beta-sheet |
| **beta-sheet** | 8 | S D I M W T V K Y | 84-92 | beta-sheet | D S I M W T V K F | 84-92 | beta-sheet |
| (69-104 AA) | 9 | K V L R F K F A I | 96-104 | beta-sheet | N M K R F K F P I | 96-104 | beta-sheet |
| **Tail** | 10 | E E A L | 123-126 | alpha-helix | **missing** |  |  |
| (105-148 AA) | 11 | E I | 136-137 | alpha-helix | D L | 133-134 | alpha-helix |
|  |  |  |  |  |  |  |  |

**Structural deviations:** 114-120 loop **139 AA total** **Protein problems: backbone**

**identical AA 79** 56,9% Pro2, Lys14, Pro79, Ser82, Asp121, Gln136

**similar AA** 103** 74,1% **sidechain**

**1.0Ǻ RMSD backbone 131 92,2%** Glu6, Lys18, Thr19, Met22, Met30, Glu36, Cys47, Asn122 ** incl. identical AA

**Ramachandran Plot**

Ser82, Lys14, Asp121, Gly74, Gly95, Gln136

**AA Accessibility**: **Trp48,** Lys14**, Asp17**, Glu8, **Lys34**, Thr106

# Secondary structure element succession AprB *Desulfotalea psychrophila*

| **AprB segments** | **No** | ***Archaeoglobus fulgidus* sequence** | AA position | **Sec. str. element** | *Desulfotalea psychrophila* sequence | AA position | **Sec. str. element** |
| --- | --- | --- | --- | --- | --- | --- | --- |
| Ferredoxin | 1 | P S F V N | 2-6 | beta-sheet | P S Y V D (Loop: KGGDK) | 2-6 | beta-sheet |
| (2-68 AA) | 2 | A C E Y I | 20-24 | alpha-helix | A C M Y I | 20-24 | alpha-helix |
|  | 3 | L M T L D | 29-33 | beta-sheet | L M V L D | 29-33 | beta-sheet |
|  | 4 | K A Y N R | 38-42 | beta-sheet | K A Y N Q | 38-42 | beta-sheet |
|  | 5 | S C V K M | 52-56 | alpha-helix | S C V K I | 52-56 | alpha-helix |
|  | 6 | A I D V R | 61-65 | beta-sheet | A I R V R | 61-65 | beta-sheet |
| **3 antiparallel** | 7 | A C V P M R G | 76-82 | beta-sheet | Q V H P M R S | 76-82 | beta-sheet |
| **beta-sheet** | 8 | S D I M W T V K Y | 84-92 | beta-sheet | D S I M W T V K F | 84-92 | beta-sheet |
| (69-104 AA) | 9 | K V L R F K F A I | 96-104 | beta-sheet | A L K R F K F P I | 96-104 | beta-sheet |
| **Tail** | 10 | E E A L | 123-126 | alpha-helix | **missing** |  |  |
| (105-148 AA) | 11 | E I | 136-137 | alpha-helix | E L | 133-134 | alpha-helix |
|  |  |  |  |  |  |  |  |

**Structural deviations:** 118-122 loop **138 AA total** **Protein problem: backbone**

**identical AA 82** 59,4% Pro2, Lys14, Pro79, Ser82, Thr136

**similar AA** 103** 74,6% **sidechain**

**1.0Ǻ RMSD backbone 133 96,4%** Lys18, Thr19, Met22, Met30, Cys47, Asp33, Ser36 ** incl. identical AA

**Ramachandran Plot**

Ser82, Lys14, Gly120, Gly74, Gly95, Thr136

**AA Accessibility**: **Trp48, Lys14, Asp17**, Lys34, Thr106

# Secondary structure element succession AprB *Olavius algarvensis* Delta 1 symbiont

| **AprB segments** | **No** | ***Archaeoglobus fulgidus* sequence** | AA position | **Sec. str. element** | *O. algarvensis* Delta 1 symbiont sequence | AA position | **Sec. str. element** |
| --- | --- | --- | --- | --- | --- | --- | --- |
| Ferredoxin | 1 | P S F V N | 2-6 | beta-sheet | P S F V D (Loop: KGGDK) | 2-6 | beta-sheet |
| (2-68 AA) | 2 | A C E Y I | 20-24 | alpha-helix | A C Q Y I | 20-24 | alpha-helix |
|  | 3 | L M T L D | 29-33 | beta-sheet | L M V L E | 29-33 | beta-sheet |
|  | 4 | K A Y N R | 38-42 | beta-sheet | K A Y N Q | 38-42 | beta-sheet |
|  | 5 | S C V K M | 52-56 | alpha-helix | S C V K I | 52-56 | alpha-helix |
|  | 6 | A I D V R | 61-65 | beta-sheet | A I E V R | 61-65 | beta-sheet |
| **3 antiparallel** | 7 | A C V P M R G | 76-82 | beta-sheet | S V M P M L G | 76-82 | beta-sheet |
| **beta-sheet** | 8 | S D I M W T V K Y | 84-92 | beta-sheet | E D V M W T C K F | 84-92 | beta-sheet |
| (69-104 AA) | 9 | K V L R F K F A I | 96-104 | beta-sheet | T I K R F K F P I | 96-104 | beta-sheet |
| **Tail** | 10 | E E A L | 123-126 | alpha-helix | **missing** |  |  |
| (105-148 AA) | 11 | E I | 136-137 | alpha-helix | A D | 133-134 | alpha-helix |
|  |  |  |  |  |  |  |  |

**Structural deviations:** 118-122 loop **147 AA total** **Protein problems: backbone**

**identical AA 78** 53,1% Pro2, Lys14, Pro79, Thr130, Thr136

**similar AA** 102** 69,4% **sidechain**

**1.0Ǻ RMSD backbone 142 96,6%** Lys18, Thr19, Gln22, Met30, Glu33, Glu36, Gln46, Cys47, Gln111, Ser125, ** incl. identical AA Thr130, Asp134

**Ramachandran Plot**

Gly82, Lys14, Gly120, Gly74, Gly95, Thr130, Thr136

**AA Accessibility**: **Glu8,** **Trp48,** Lys14, **Asp17**, Asp69, Thr106

# Secondary structure element succession AprB *Thermodesulfovibrio yellowstonii*

| **AprB segments** | **No** | ***Archaeoglobus fulgidus* sequence** | AA position | **Sec. str. element** | *Thermodesulfovibrio yellowstonii* sequence | AA position | **Sec. str. element** |
| --- | --- | --- | --- | --- | --- | --- | --- |
| Ferredoxin | 1 | P S F V N | 2-6 | beta-sheet | P S F V I (Loop: KAQDK) | 2-6 | beta-sheet |
| (2-68 AA) | 2 | A C E Y I | 20-24 | alpha-helix | A C Q Y I | 20-24 | alpha-helix |
|  | 3 | L M T L D | 29-33 | beta-sheet | L M T L D | 29-33 | beta-sheet |
|  | 4 | K A Y N R | 38-42 | beta-sheet | K A F N Q | 38-42 | beta-sheet |
|  | 5 | S C V K M | 52-56 | alpha-helix | N C V K I | 52-56 | alpha-helix |
|  | 6 | A I D V R | 61-65 | beta-sheet | A I E V R | 61-65 | beta-sheet |
| **3 antiparallel** | 7 | A C V P M R G | 76-82 | beta-sheet | S V I P M R G | 76-82 | beta-sheet |
| **beta-sheet** | 8 | S D I M W T V K Y | 84-92 | beta-sheet | D A I M W T I K F | 84-92 | beta-sheet |
| (69-104 AA) | 9 | K V L R F K F A I | 96-104 | beta-sheet | S I K R F K F P I | 96-104 | beta-sheet |
| **Tail** | 10 | E E A L | 123-126 | alpha-helix | Y G K I | 126-129 | alpha-helix |
| (105-148 AA) | 11 | E I | 136-137 | alpha-helix | A R | 139-140 | alpha-helix |
|  |  |  |  |  |  |  |  |

**Structural deviations:** 111-116 loop **142 AA total** **Protein problems: backbone**

**identical AA 81** 57,0% Pro2, Lys14, Pro79, Tyr118, Asn136, Glu142

**similar AA** 104** 73,2% **sidechain**

**1.0Ǻ RMSD backbone 136 95,8%** Lys18, Thr19, Gln22, Met30, Lys36, Gln46, Cys47, Cys71, Tyr111, Tyr118, ** incl. identical AA Tyr137, Lys141

**Ramachandran Plot**

Gly82, Lys14, Glu142, Gly74, Gly95, Asn136, Tyr118

**AA Accessibility**: **Glu8**, **Trp48, Asp17**, **Arg34**, Asp69, Leu106

# Secondary structure element succession AprB *Chlorobaculum tepidum*

| **AprB segments** | **No** | ***Archaeoglobus fulgidus* sequence** | AA position | **Sec. str. element** | *Chlorobaculum tepidum*  sequence | AA position | **Sec. str. element** |
| --- | --- | --- | --- | --- | --- | --- | --- |
| Ferredoxin | 1 | P S F V N | 2-6 | beta-sheet | P S F V I (Loop : KGQER) | 2-6 | beta-sheet |
| (2-68 AA) | 2 | A C E Y I | 20-24 | alpha-helix | A C M Y I | 20-24 | alpha-helix |
|  | 3 | L M T L D | 29-33 | beta-sheet | L M K L D | 29-33 | beta-sheet |
|  | 4 | K A Y N R | 38-42 | beta-sheet | K A W N Q | 38-42 | beta-sheet |
|  | 5 | S C V K M | 52-56 | alpha-helix | N C V K I | 52-56 | alpha-helix |
|  | 6 | A I D V R | 61-65 | beta-sheet | A I E V R | 61-65 | beta-sheet |
| **3 antiparallel** | 7 | A C V P M R G | 76-82 | beta-sheet | N V I P L R G | 76-82 | beta-sheet |
| **beta-sheet** | 8 | S D I M W T V K Y | 84-92 | beta-sheet | D A I M W T I K F | 84-92 | beta-sheet |
| (69-104 AA) | 9 | K V L R F K F A I | 96-104 | beta-sheet | I L K R Y K F P I | 96-104 | beta-sheet |
| **Tail** | 10 | E E A L | 123-126 | alpha-helix | Y A N L | 123-126 | alpha-helix |
| (105-148 AA) | 11 | E I | 136-137 | alpha-helix | E Y | 136-137 | alpha-helix |
|  |  |  |  |  |  |  |  |

**Structural deviations:** - **140 AA total** **Protein problems: backbone**

**identical AA 86** 61,4% nicht vorhanden

**similar AA** 104** 74,3% **sidechain**

**1.0Ǻ RMS AA 139 99,3%** Arg18, Thr19, Met30, Glu46, Cys47, Asp13, Lys118, Met134, Tyr137 ** incl. identical AA

**Ramachandran Plot**

Gly82, Lys14, Gly74, Gly95, Asn133, Thr139

**AA Accessibility**: **Trp48, Glu8, Glu17**, Lys14, Asp69, Thr106

# Secondary structure element succession AprB *Thiobacillus denitrificans* ATCC25259

| **AprB segments** | **No** | ***Archaeoglobus fulgidus* sequence** | AA position | **Sec. str. element** | *Thiobacillus denitrificans*  sequence | AA position | **Sec. str. element** |
| --- | --- | --- | --- | --- | --- | --- | --- |
| Ferredoxin | 1 | P S F V N | 2-6 | beta-sheet | P T Y V R (Loop: KGQDK) | 2-6 | beta-sheet |
| (2-68 AA) | 2 | A C E Y I | 20-24 | alpha-helix | A C M Y I | 20-24 | alpha-helix |
|  | 3 | L M T L D | 29-33 | beta-sheet | L M K L D **(additional loop: 36-41 AA)** | 29-33 | beta-sheet |
|  | 4 | K A Y N R | 38-42 | beta-sheet | R A F N Q | 44-48 | beta-sheet |
|  | 5 | S C V K M | 52-56 | alpha-helix | S C V K I | 58-62 | alpha-helix |
|  | 6 | A I D V R | 61-65 | beta-sheet | A I E A R | 67-71 | beta-sheet |
| **3 antiparallel** | 7 | A C V P M R G | 76-82 | beta-sheet | M V Q P L R G | 82-89 | beta-sheet |
| **beta-sheet** | 8 | S D I M W T V K Y | 84-92 | beta-sheet | D S I M W T I K F | 90-98 | beta-sheet |
| (69-104 AA) | 9 | K V L R F K F A I | 96-104 | beta-sheet | T L K R F K F P I | 102-110 | beta-sheet |
| **Tail** | 10 | E E A L | 123-126 | alpha-helix | L A D L | 129-132 | alpha-helix |
| (105-148 AA) | 11 | E I | 136-137 | alpha-helix | **missing** |  |  |
|  |  |  |  |  |  |  |  |

**Structural deviations:** 34-41 loop **157 AA total** **Protein problems: backbone**

142-150 loop **identical AA 77** 49,0% Pro2, Lys14, Ala42, Pro85, Thr139, Pro155

155-157 loop **similar AA** 106** 67,5% **sidechain**

**1.0Ǻ RMSD backbone 140 89,2%** Lys18, Thr19, Met22, Met30, His72, Ser141, Asp143

** incl. identical AA **Ramachandran Plot**

Lys14, Ala42, Gly88, Gly80, Gly101, Thr139, Gly145, Glu157

**AA Accessibility**: **Lys14, Asp17, Glu38, Glu51, Trp54**, Asp75, Thr112
