# Supplementary material for: Homology Modeling of Dissimilatory APS Reductases (AprBA) of Sulfur-Oxidizing and Sulfate-Reducing Prokaryotes
Source: PLoS One. 2008 Jan 30;3(1):e1514. doi: 10.1371/journal.pone.0001514 (PMC2211403; doi:10.1371/journal.pone.0001514)
Supplement: Table S2 — (0.21 MB DOC) [file pone.0001514.s006.doc]

**Supplementary data material Table S2. AprB protein matrix surrounding the [4Fe-4S] clusters I and II: appearance of residues in a [4Fe-4S] cluster distance range from 2.0 to 5.0Å**

| [4Fe-4S] cluster | Distance in Å | *Archaeoglobus fulgidus* | *Allochromatium vinosum* | Thiobacillus  *denitrificans* | *Cdt.* Ruthia  magnifica | *Pelagibacter ubique* | EBAC2C11 | *Pyrobaculum calidifontis* | *Pyrobaculum aerophilum* | *Caldivirga maquilingensis* | *Chlorobaculum tepidum* | *Thermodesulfovibrio yellowstonii* | Thiobacillus  *denitrificans* |
| --- | --- | --- | --- | --- | --- | --- | --- | --- | --- | --- | --- | --- | --- |
| Cluster I | 2.0 | - | Ile24 | Ile24 | Ile24 | Ile24 | Ile24 | - | - | Ile24 | - | - | - |
|  | 2.5 | Cys25, Cys47, Cys50, Cys53 | Cys20, Cys42, Cys45, Cys48 | Cys20, Cys42, Cys45, Cys48 | Cys20, Cys42, Cys45, Cys48 | Cys20, Cys42, Cys45, Cys48 | Cys20, Cys42, Cys45, Cys48 | Cys20, Cys42, Cys45, Cys48 | Cys20, Cys42, Cys45, Cys48 | Cys20, Cys45, Cys48, Cys51 | Cys25, Cys47, Cys50, Cys53 | Cys25, Cys47, Cys50, Cys53 | Cys25, Cys53, Cys56, Cys59 |
|  | 3.0 | - | Thr3 | Thr3 | Thr3 | Thr3 | Thr3 | Thr3 | Thr3 |  | - | - | Thr3 |
|  | 3.5 | Ser3, Asn41, Tyr51 | Tyr46 | Tyr46 | Tyr46 | Tyr46 | Tyr46 | Leu46 | Met46 | Ser3, Tyr49 | Ser3, Tyr51 | Ser3, Tyr51 | Tyr57 |
|  | 4.0 | Pro26, Leu29, Met30, Trp48, Glu49, Ser52 | Pro21, Met25, Asn36, Glu44, Trp43, Ser47 | Pro21, Met25, Asn36, Glu44, Trp43, Ser47 | Pro21, Met25, Asn36, Glu44, Trp43, Ser47 | Pro21, Met25, Asn36, Glu44, Trp43, Ser47 | Pro21, Met25, Asn36, Glu44, Trp43, Ser47 | Pro21, Met25, Asn36, Glu44, Ala43, Asn47 | Pro21, Met25, Asn36, Glu44, Ala43, Asn47 | Pro21, Met25, Asn39, Glu47, Trp46, Asn50, Ile62 | Pro26, Leu29, Met30, Asn41, Trp48, Glu49, Asn52 | Pro26, Leu29, Met30, Asn41, Trp48, Glu49, Asn52 | Pro26, His27, Leu29, Met30, Asn47, Trp54, Glu55,Ser58 |
|  | 4.5 | - | - | - | - | - | - | Asn24 | Asn24 | - | - | - | - |
|  | 5.0 | Asn27, Val64, Ile24, Met46 | Ile19, Ser22, Met41, Val59 | Ile19, Ser22, Met41, Val59 | Ile19, Ser22, Met41, Val59 | Ile19, Ser22, Phe41, Val59 | Ile19, Ser22, Phe41 | Ile19, Ala22, Ser41, Val59 | Ile19, Ala22, Ser41, Val59 | Ile19, Ser22, Ser44 | Ile24, Asn27, Gln46, Val64 | Ile24, Asn27, Gln46, Val64 | Ile24, Gln52 |
|  |  |  |  |  |  |  |  |  |  |  |  |  |  |
| Cluster II | 2.0 | - | Cys13 | Cys13, Tyr15 | Cys13 | Cys13 | Cys13 | Cys13 | Cys13 | Cys13 | - | - | - |
|  | 2.5 | Cys10, Cys21, Cys57 | Cys10, Gly12, Cys16, Cys52 | Cys10, Gly12, Cys16 | Cys10, Gly12, Cys16, Cys52 | Cys10, Gly12, Cys16, Cys52 | Cys10, Gly12, Cys16, Cys52 | Cys10, Gly12, Cys16, Cys52 | Cys10, Gly12, Cys16, Cys52 | Cys10, Gly12, Cys16, Cys55 | Cys10, Cys13, Cys21, Cys57 | Cys10, Cys13, Cys21, Cys57 | Cys10, Cys13, Cys21, Cys63 |
|  | 3.0 | Cys13 | Gly14 | Cys52, Gly14 | Gly14 | Gly14 | Gly14 | Gly14, Glu54 | Gly14, Glu54 | Gly14 | - | - | - |
|  | 3.5 | Asp11 | Asp11, His54, Ile57 | Asp11, Ile57 | Asp11, His15, Ile57 | Asp11, Asn54, Ile57 | Asp11, Ile57 | Tyr27 | Tyr27 | Phe27 | Asp11, Ala20 | Asp11, Ala20 | Asp11, Ala20 |
|  | 4.0 | Val5, Gly12, Thr19, Ala20, Ala39, Pro58, Gln59, Ile62 | Val5, Gln15, Ala34, Pro53, | Val5, Ala34, Met54, Pro53 | Val5, Ala34, His54, Pro53 | Val5, His15, Ala34, Pro53 | Val5, His15, Ala34, Pro53, Gln54 | Val5, Lys11, Lys15, Ala34, Pro53 | Val5, Lys11, Lys15, Pro53 | Val5, Thr11, Asp15, Ala37, Pro56, Gln57 | Val5, Gly12, Thr19, Ala39, Pro58, Gln59, Ile62 | Val5, Gly12, Thr19, Ala39, Pro58, Gln59, Ile62 | Gly12, Thr19, Ala45, Pro64, Gln65, Ile68 |
|  | 4.5 | Leu32, Ala61 | Ala56 | Ala56 | Ala56 | Ala56 | Ala56 | Ala56 | Gly34, Ala56 | Ala59 | Leu32, Ala61 | Leu32, Ala61 | Val5, Leu32, Ala67 |
|  | 5.0 | Lys9 | Arg9, Ile27 | Arg9 | Arg9, Ile27 | Arg9, Ile27 | Ser9, Ile27 | Leu9, Val57 | Leu9, Val57 | Lys9, Val60 | Lys9 | Lys9 | Lys9 |

| [4Fe-4S] cluster | Distance in Å | *Archaeoglobus fulgidus* | *Desulfotomaculum reducens* | *Syntrophobacter fumaroxidans* | fosws39f7 | fosws7f8 | *Thermodesulfobac-terium commune* | *Desulfovibrio desulfuricans* | *Desulfovibrio vulgaris* | *Desulfobulbus* sp. | *Desulfotalea psychrophila* | *O. algarvensis* Delta 1 symbiont |
| --- | --- | --- | --- | --- | --- | --- | --- | --- | --- | --- | --- | --- |
| Cluster I | 2.0 | - | - | - | - | - | - | - | - | - | - | - |
|  | 2.5 | Cys25, Cys47, Cys50, Cys53 | Cys25, Cys47, Cys50, Cys53 | Cys25, Cys47, Cys50, Cys53 | Cys25, Cys47, Cys50, Cys53 | Cys25, Cys47, Cys50, Cys53 | Cys25, Cys47, Cys50, Cys53 | Cys25, Cys47, Cys50, Cys53 | Cys25, Cys47, Cys50, Cys53 | Cys25, Cys47, Cys50, Cys53 | Cys25, Cys47, Cys50, Cys53 | Cys25, Cys47, Cys50, Cys53 |
|  | 3.0 | - | - | - | - | - | - | Thr3 | Thr3 |  |  |  |
|  | 3.5 | Ser3, Asn41, Tyr51 | Ser3, Tyr51 | Ser3, Tyr51 | Ser3, Asn41, Tyr51 | Ser3, Tyr51 | Ser3, Tyr51 | Tyr51 | Tyr51 | Ser3, Tyr51 | Ser3, Tyr51 | Ser3, Phe51 |
|  | 4.0 | Pro26, Leu29, Met30,  Trp48, Glu49, Ser52 | Pro26, Leu29, Met30, Asn41, Trp48, Glu49, Cys52 | Pro26, Leu29, Met30, Asn41, Trp48, Glu49, Asn52 | Pro26, Leu29, Met30,  Trp48, Glu49, Ser52 | Pro26, Leu29, Met30, Asn41, Trp48, Glu49, Cys52 | Pro26, Leu29, Met30, Asn41, Trp48, Glu49, Ser52 | Pro26, Leu29, Met30, Asn41, Trp48, Glu49, Ser52 | Pro26, Leu29, Met30, Asn41, Trp48, Glu49, Ser52 | Pro26, Leu29, Met30, Asn41, Trp48, Glu49, Ser52 | Pro26, Leu29, Met30, Asn41, Trp48, Glu49, Ser52 | Pro26, Leu29, Met30, Asn41, Trp48, Glu49, Ser52 |
|  | 4.5 | - | - | - | - | - | - | - | - | - | - | - |
|  | 5.0 | Asn27, Val64, Ile24, Met46 | Ile24, Asn27, Val64, Gln46 | Ile24, Asn27, Val64, Met46 | Asn27, Val54, Met46 | Asn27, Val64, Met46 | Ile24, Asn27, Ile64, Gln46 | Ile24, Asn27, Ala46 | Ile24, Asn27, Ala46 | Ile24, Asn27, Ala46, Val64 | Ile24, Asn27, Gly46, Val64 | Ile24, Asn27, Gln46, Val64 |
|  |  |  |  |  |  |  |  |  |  |  |  |  |
| Cluster II | 2.0 | - | - | - | - | - | - | - | - | - | - | - |
|  | 2.5 | Cys10, Cys21, Cys57 | Cys10, Cys13, Cys21, Cys57 | Cys10, Cys13, Cys21, Cys57 | Cys10, Cys21, Cys57 | Cys10, Cys13, Cys21, Cys57 | Cys10, Cys13, Cys21, Cys57, Val20 | Cys10, Cys13, Cys21, Cys57 | Cys10, Cys13, Cys21, Cys57 | Cys10, Cys13, Cys21, Cys57 | Cys10, Cys13, Cys21, Cys57 | Cys10, Cys13, Cys21, Cys57 |
|  | 3.0 | Cys13 | - | - | Cys13 | - | - | - | - | - | - | Thr59 |
|  | 3.5 | Asp11 | Asp11, Ala20, Ile62 | Asp11, Ala20 | Asp11 | Asp11, Ala20, Val59 | Asp11 | Asp11, Ala20, Ile62 | Asp11, Ala20, Ile62 | Asp11, Ala20 | Asp11, Ala20, Ile62 | Asp11, Ala20, Ile62 |
|  | 4.0 | Val5, Gly12, Thr19, Ala20, Ala39, Pro58, Gln59, Ile62 | Val5, Gly12, Thr19, Ala39, Pro58, Gln59 | Val5, Gly12, Thr19, Ala39, Pro58, Gln59, Ile62 | Val5, Gly12, Thr19, Ala20, Ala39, Pro58, Ile62 | Val5, Gly12, Thr19, Ala39, Pro58, Gln59, Ile62 | Val5, Gly12, Thr19, Ala39, Pro58, Gln59, Ile62 | Val5, Gly12, Thr19, Ala39, Pro58, Gln59 | Val5, Gly12, Thr19, Ala39, Pro58, Gln59 | Val5, Gly12, Thr19, Ala39, Pro58, Gln59, Ile62 | Val5, Gly12, Thr19, Ala39, Pro58, Gln59 | Val5, Gly12, Thr19, Ala39, Pro58, Thr59 |
|  | 4.5 | Leu32, Ala61 | Leu32, Ala61 | Leu32, Ala61 | Leu32, Val59, Ala61 | Leu32, Ala61 | Leu32, Ala61 | Leu32, Ala61 | Leu32, Ala61 | Leu32, Ala61 | Leu32, Ala61 | Leu32, Ala61 |
|  | 5.0 | Lys9 | Lys9 | Lys9 | Lys9, Ile56 | Lys9 | Lys9 | Lys9 | Lys9 | Lys9 | Lys9 | Lys9 |
