# Supplementary material for: Homology Modeling of Dissimilatory APS Reductases (AprBA) of Sulfur-Oxidizing and Sulfate-Reducing Prokaryotes
Source: PLoS One. 2008 Jan 30;3(1):e1514. doi: 10.1371/journal.pone.0001514 (PMC2211403; doi:10.1371/journal.pone.0001514)
Supplement: Table S3 — (2.10 MB DOC) [file pone.0001514.s007.doc]

#### Supplementary data material Table S3. AprA secondary structure element succession

# SOB Apr lineage I

# Secondary structure element succession AprA *Allochromatium vinosum*

| **AprA domains** | **No** | ***Archaeoglobus fulgidus* sequence** | AA position | **Sec. str. element** | *Allochromatium vinosum* sequence | AA position | **Sec. str. element** |
| --- | --- | --- | --- | --- | --- | --- | --- |
| FAD-Binding | 1 | P T E V V E T D I L I I G | 17-29 | beta-sheet | T I I E D G I D V L V V G | 5-17 | beta-sheet |
| **site I** | 2 | F S G C G G A A Y E A A Y W A K | 32-44 | alpha-helix | L G G T G A A F E A R Y W→ **shortened** | 20-32 | alpha-helix |
| (2-261 AA) | 3 | K V T L V E | 51-56 | beta-sheet | K I V I A E | 37-42 | beta-sheet |
|  | 4 | S A I | 71-73 | beta-sheet | Y A I **loop shortended** | 57-59 | beta-sheet |
|  | 5 | L E D Y V R Y V T L D M | 89-100 | alpha-helix | P E D H V R Y A R I D L | 72-83 | alpha-helix |
|  | 6 | E D L V A D Y A R H V D G T V H L F E K | 106-125 | alpha-helix | E D L L F D M A R H V D S A V H Q F E E W | 89-109 | alpha-helix |
|  | 7 | P I W K T | 129-133 | beta-sheet | P L M R N **loop elongated** | 112-116 | beta-sheet |
|  | 8 | K Y V R E | 137-141 | beta-sheet | A Y Q R E | 121-125 | beta-sheet |
|  | 9 | Q I M I H | 145-149 | beta-sheet | Q I M I H | 128-133 | beta-sheet |
|  | 10 | Y K P I I A E A A K M A V | 153-165 | alpha-helix | Y K P I V A E A A K K S→ **shortened** | 137-148 | alpha-helix |
|  | 11 | N I Y E R V F I F E L L K D | 169-182 | beta-sheet | →F N R I C V T H L L M D **shortened** | 153-164 | beta-sheet |
|  | 12 | A V A G A V G F S V | 188-197 | beta-sheet | R I A G A V G F N V | 170-179 | beta-sheet |
|  | 13 | K F Y V F K A | 201-207 | beta-sheet | N Y H V F K S | 183-189 | beta-sheet |
|  | 14 | A V I L A | 209-213 | beta-sheet | T V I V A | 191-195 | beta-sheet |
|  | 15 | G A T | 216-218 | beta-sheet | G A S | 198-200 | beta-sheet |
|  | 16 | A A | 229-230 | alpha-helix | G A | 211-212 | alpha-helix |
|  | 17 | D T G | 239-241 | beta- sheet | S S G | 221-223 | beta- sheet |
|  | 18 | S G Y Y M G L K A | 242-250 | alpha-helix | S A Y G L L I G A | 224-232 | alpha-helix |
|  | 19 | M L T Q | 253-256 | beta-sheet | K M T Q | 235-238 | beta-sheet |
| Capping | 20 | P F R F K | 263-267 | beta-sheet | L A R F K | 245-249 | beta-sheet |
| (262-393 AA) | 21 | G A W F L F | 274-279 | alpha-helix | G A Y F L H | 256-261 | alpha-helix |
|  | 22 | K A K N A | 283-287 | beta-sheet | Y T Q N G | 265-269 | beta-sheet |
|  | 23 | I K | 293-294 | alpha-helix | **missing** |  | alpha-helix |
|  | 24 | A | 298 | alpha-helix | **K E Y L D** **elongated alpha-helix** | 288-292 | alpha-helix |
|  | 25 | Y G | 306-307 | alpha-helix | H R | 297-298 | alpha-helix |
|  | 26 | T P L R N H Q V M L E I M D | 314-327 | alpha-helix | T C L R N H A L I S E V N A | 305-318 | alpha-helix |
|  | 27 | P I M Y M H | 331-335 | beta-sheet | P I H M V | 322-326 | beta-sheet |
|  | 28 | T E E A L A E L A | 336-344 | alpha-helix | T M E A→ **shortened** | 327-330 | alpha-helix |
|  | 29 | K K K L K H I Y E E A F E D F L | 348-363 | alpha-helix | →L E E I G W H N F L **shortened** | 336-344 | alpha-helix |
|  | 30 | S Q A L L W A C Q | 368-375 | alpha-helix | G Q A V L W A A T | 350-358 | alpha-helix |
|  | 31 | S E A A P A | 385-390 | beta-sheet | P E L T T S | 367-372 | beta-sheet |
| **FAD Binding** | 32 | G F W | 403-405 | beta-sheet | G A W | 385-387 | beta-sheet |
| **site II** | 33 | K | 419 | alpha-helix | **missing** |  | alpha-helix |
| (393-487 AA) | 34 | R M T | 427-429 | beta-sheet | R M T | 405-407 | beta-sheet |
|  | 35 | G L F A I | 433-437 | beta-sheet | G L F G A | 411-415 | beta-sheet |
|  | 36 | S S G S F T E G R I A A K A A V R F I L E Q | 449-470 | alpha-helix | S S G S F T E G R L A A K A A C K Y I D D G **loop elongated** | 427-448 | alpha-helix |
|  | 37 | D A V V E E L K K K | 478-487 | alpha-helix | D A Q I E R R R Q E | 457-466 | alpha-helix |
| Helical | 38 | P M E R F M Q | 491-497 | alpha-helix | P M E H Y R V | 470-476 | alpha-helix |
| (488-643 AA) | 39 | P W Q G L V R L Q K I M D E | 514-527 | alpha-helix | P R Q G L D R L Q K L M D E | 493-506 | alpha-helix |
|  | 40 | Y K T | 536-538 | beta-sheet | Y M T | 515-517 | beta-sheet |
|  | 41 | E K M L L Q R A L E L L A F L K E D L | 540-557 | alpha-helix | E N L L N I G L K K M K L L E E D L | 519-536 | alpha-helix |
|  | 42 | L H E L M R A W E L V H R V W T A E A H V R H M L F | 565-590 | alpha-helix | I H E L L R A W E L K H R Q L T S E A V L H H T L F | 544-569 | alpha-helix |
|  | 43 | Y R T | 601-603 | beta-sheet | Y R G | 580-582 | beta-sheet |
|  | 44 | K C F V C S K Y D | 614-622 | beta-sheet | H V L T V S R R D | 593-601 | beta-sheet |
|  | 45 | E W T F E K V P Y | 627-635 | beta-sheet | E Y T M E K A P C | 606-614 | beta-sheet |
|  |  |  |  |  |  |  |  |

**Structural deviations:** 33-35 loop **620 AA total** **Protein problems: backbone**

66-69 loop **identical AA 306** 49,4% Ala44, Asp250, Glu275, His335, Pro367, His380,

116-119 loop **similar AA** 437** 70,5% Tyr407, Phe426, Val455 148-151 loop **1.0Ǻ RMSD backbone 583 94,0%**

278-286 loop ** incl. identical AA

331-334 loop

396-402 alpha-Helix missing

452-454 loop

# Secondary structure element succession AprA *Thiobacillus denitrificans* ATCC25259

| **AprA domains** | **No** | ***Archaeoglobus fulgidus* sequence** | AA position | **Sec. str. element** | *Thiobacillus denitrificans* sequence | AA position | **Sec. str. element** |
| --- | --- | --- | --- | --- | --- | --- | --- |
| FAD-Binding | 1 | P T E V V E T D I L I I G | 17-29 | beta-sheet | T V V E D N I D I L V V G | 5-17 | beta-sheet |
| **site I** | 2 | F S G C G G A A Y E A A Y W A K | 32-44 | alpha-helix | L G G T G A A W E A R Y W→ **shortened** | 20-32 | alpha-helix |
| (2-261 AA) | 3 | K V T L V E | 51-56 | beta-sheet | K I V I A E | 39-42 | beta-sheet |
|  | 4 | S A I | 71-73 | beta-sheet | Y A I **loop elongated** | 57-59 | beta-sheet |
|  |  |  |  |  | M G T (new) | 63-65 | **beta-sheet** |
|  |  |  |  |  | G E N (new) | 68-70 | **beta-sheet** |
|  | 5 | L E D Y V R Y V T L D M | 89-100 | alpha-helix | P E D H V R Y A R M D L | 72-83 | alpha-helix |
|  | 6 | E D L V A D Y A R H V D G T V H L F E K | 106-125 | alpha-helix | E D L L F D M A R H V D S A V H Q F E E **W** verlängert | 89-109 | alpha-helix |
|  | 7 | P I W K T | 129-133 | beta-sheet | P I M R D **loop elongated** | 112-116 | beta-sheet |
|  | 8 | K Y V R E | 137-141 | beta-sheet | H Y Q R E | 121-125 | beta-sheet |
|  | 9 | Q I M I H | 145-149 | beta-sheet | Q I M I H | 129-133 | beta-sheet |
|  | 10 | Y K P I I A E A A K M A V | 153-165 | alpha-helix | Y K P I V A E A A K K S A→ **shortened** | 137-149 | alpha-helix |
|  | 11 | N I Y E R V F I F E L L K D | 169-182 | beta-sheet | →Y N R I C V T H L L M D **shortened** | 153-164 | beta-sheet |
|  | 12 | A V A G A V G F S V | 188-197 | beta-sheet | R V A G A V G F N V | 170-179 | beta-sheet |
|  | 13 | K F Y V F K A | 201-207 | beta-sheet | D Y H V F K S | 183-189 | beta-sheet |
|  | 14 | A V I L A | 209-213 | beta-sheet | T V I L G | 191-195 | beta-sheet |
|  | 15 | G A T | 216-218 | beta-sheet | G A S | 198-200 | beta-sheet |
|  | 16 | A A | 229-230 | alpha-helix | G A | 211-212 | alpha-helix |
|  | 17 | D T G | 239-241 | beta- sheet | S S G | 221-223 | beta- sheet |
|  | 18 | S G Y Y M G L K A | 242-250 | alpha-helix | S A Y G L M I Q A | 224-232 | alpha-helix |
|  | 19 | M L T Q | 253-256 | beta-sheet | K M T Q | 235-238 | beta-sheet |
| Capping | 20 | P F R F K | 263-267 | beta-sheet | L A R F K | 245-249 | beta-sheet |
| (262-393 AA) | 21 | G A W F L F | 274-279 | alpha-helix | G A Y F L H | 256-261 | alpha-helix |
|  | 22 | K A K N A | 283-287 | beta-sheet | Y T Q N C | 265-269 | beta-sheet |
|  | 23 | I K | 293-294 | alpha-helix | P A L T E alpha-helix elongated | 280-284 | alpha-helix |
|  | 24 | A | 298 | alpha-helix | E | 289 | alpha-helix |
|  | 25 | Y G | 306-307 | alpha-helix | H L | 297-298 | alpha-helix |
|  | 26 | T P L R N H Q V M L E I M D | 314-327 | alpha-helix | T C L R N H A F I S E V N A | 305-318 | alpha-helix |
|  | 27 | P I M Y M H | 331-335 | beta-sheet | P I H M V | 322-326 | beta-sheet |
|  | 28 | T E E A L A E L A | 336-344 | alpha-helix | T M E A→ **shortened** | 327-330 | alpha-helix |
|  | 29 | K K K L K H I Y E E A F E D F L | 348-363 | alpha-helix | →L E E I G W H N F L **shortened** | 336-345 | alpha-helix |
|  | 30 | S Q A L L W A C Q | 368-375 | alpha-helix | G Q A V L W A A T | 350-358 | alpha-helix |
|  | 31 | S E A A P A | 385-390 | beta-sheet | P E L T T S | 367-372 | beta-sheet |
| **FAD Binding** | 32 | G F W | 403-405 | beta-sheet | G A W | 385-387 | beta-sheet |
| **site II** | 33 | K | 419 | alpha-helix | **missing** |  |  |
| (393-487 AA) | 34 | R M T | 427-429 | beta-sheet | R M T | 405-407 | beta-sheet |
|  | 35 | G L F A I | 433-437 | beta-sheet | G L F G A | 411-415 | beta-sheet |
|  | 36 | S S G S F T E G R I A A K A A V R F I L E Q | 449-470 | alpha-helix | S S G S F T E G R L A A C K Y I D D G **loop elongated**. | 427-448 | alpha-helix |
|  | 37 | D A V V E E L K K K | 478-487 | alpha-helix | D E Q I Q R R R E E | 457-466 | alpha-helix |
| Helical | 38 | P M E R F M Q | 491-497 | alpha-helix | P M E H Y R I | 470-476 | alpha-helix |
| (488-643 AA) | 39 | P W Q G L V R L Q K I M D E | 514-527 | alpha-helix | P R Q G L D R L Q K L M D E | 493-506 | alpha-helix |
|  | 40 | Y K T | 536-538 | beta-sheet | Y M T | 515-517 | beta-sheet |
|  | 41 | E K M L L Q R A L E L L A F L K E D L | 540-557 | alpha-helix | D K L L Q I G L K K L K L M E E D L | 519-536 | alpha-helix |
|  | 42 | L H E L M R A W E L V H R V W T A E A H V R H M L F | 565-590 | alpha-helix | I H E L L R A W E L K H R H L T S E A V M Q H T L F | 544-569 | alpha-helix |
|  | 43 | Y R T | 601-603 | beta-sheet | Y R G | 580-582 | beta-sheet |
|  | 44 | K C F V C S K Y D | 614-622 | beta-sheet | H V L T V S R R D | 593-601 | beta-sheet |
|  | 45 | E W T F E K V P Y | 627-635 | beta-sheet | E Y M E K A P C | 606-614 | beta-sheet |
|  |  |  |  |  |  |  |  |

**Structural deviations:** 33-35 loop **622 AA total** **Protein problems: backbone**

- 1. loop-beta **identical AA 307** 49,4% Asn35, Ala44, Asp250, Glu275, Val 286,
  2. loop **similar AA** 424** 68,2% Pro367, His380, Phe426, Val455

148-151 loop **1.0Ǻ RMSD backbone 584 93,7%**

278-286 loop-helix-loop ** incl. identical AA

- 1. loop
  2. alpha-helix missing

452-454 loop

# Secondary structure element succession AprA *Candidatus* Ruthia magnifica

| **AprA domains** | **No** | ***Archaeoglobus fulgidus* sequence** | AA position | **Sec. str. element** | *Candidatus* Ruthia magnifica sequence | AA position | **Sec. str. element** |
| --- | --- | --- | --- | --- | --- | --- | --- |
| FAD-Binding | 1 | P T E V V E T D I L I I G | 17-29 | beta-sheet | T I V E D N I D I L V V G | 5-17 | beta-sheet |
| **site I** | 2 | F S G C G G A A Y E A A Y W A K | 32-44 | alpha-helix | L G G T G A A Y E A R → **shortened** | 20-30 | alpha-helix |
| (2-261 AA) | 3 | K V T L V E | 51-56 | beta-sheet | K I I I A E | 39-42 | beta-sheet |
|  | 4 | S A I | 71-73 | beta-sheet | Y A I **loop shortened** | 57-59 | beta-sheet |
|  | 5 | L E D Y V R Y V T L D M | 89-100 | alpha-helix | P E D H V R Y A R M D L | 72-83 | alpha-helix |
|  | 6 | E D L V A D Y A R H V D G T V H L F E K | 106-125 | alpha-helix | E D L L F D M A R H V D S A V H K F E E W | 89-109 | alpha-helix |
|  | 7 | P I W K T | 129-133 | beta-sheet | P L M K D **loop elongated** | 112-116 | beta-sheet |
|  | 8 | K Y V R E | 137-141 | beta-sheet | A Y M R E | 124-128 | beta-sheet |
|  | 9 | Q I M I H | 145-149 | beta-sheet | Q I M I H | 132-136 | beta-sheet |
|  | 10 | Y K P I I A E A A K M A V | 153-165 | alpha-helix | Y K P I V A E A A T K Q → **shortened** | 140-152 | alpha-helix |
|  | 11 | N I Y E R V F I F E L L K D | 169-182 | beta-sheet | →Y N R I M V T H L L M D **shortened** | 156-167 | beta-sheet |
|  | 12 | A V A G A V G F S V | 188-197 | beta-sheet | R I A G A V G F N V | 173-182 | beta-sheet |
|  | 13 | K F Y V F K A | 201-207 | beta-sheet | N Y H V F K S | 186-192 | beta-sheet |
|  | 14 | A V I L A | 209-213 | beta-sheet | T T I V G | 194-198 | beta-sheet |
|  | 15 | G A T | 216-218 | beta-sheet | G A S | 201-203 | beta-sheet |
|  | 16 | A A | 229-230 | alpha-helix | G M | 214-215 | alpha-helix |
|  | 17 | D T G | 239-241 | beta- sheet | S S G | 224-226 | beta- sheet |
|  | 18 | S G Y Y M G L K A | 242-250 | alpha-helix | S A Y G L L I E A | 227-235 | alpha-helix |
|  | 19 | M L T Q | 253-256 | beta-sheet | K M T Q | 238-241 | beta-sheet |
| Capping | 20 | P F R F K | 263-267 | beta-sheet | L A R F K | 248-252 | beta-sheet |
| (262-393 AA) | 21 | G A W F L F | 274-279 | alpha-helix | G A Y F L H | 259-264 | alpha-helix |
|  | 22 | K A K N A | 283-287 | beta-sheet | Y T Q N G | 268-272 | beta-sheet |
|  | 23 | I K | 293-294 | alpha-helix | W F P (new)K M V (new) | 281-283  287-289 | **beta-sheet**  **beta-sheet** |
|  | 24 | A | 298 | alpha-helix | K E | 291-292 | alpha-helix |
|  | 25 | Y G | 306-307 | alpha-helix | H L | 300-301 | alpha-helix |
|  | 26 | T P L R N H Q V M L E I M D | 314-327 | alpha-helix | T C L R N H A F I S E V N A | 308-321 | alpha-helix |
|  | 27 | P I M Y M H | 331-335 | beta-sheet | P I H M V | 325-329 | beta-sheet |
|  | 28 | T E E A L A E L A | 336-344 | alpha-helix | T M E A→ **shortened** | 330-333 | alpha-helix |
|  | 29 | K K K L K H I Y E E A F E D F L | 348-363 | alpha-helix | →L E E V G W E N F L **shortened** | 339-348 | alpha-helix |
|  | 30 | S Q A L L W A C Q | 368-375 | alpha-helix | G Q A V L W A A T | 353-361 | alpha-helix |
|  | 31 | S E A A P A | 385-390 | beta-sheet | P E L T T S | 370-375 | beta-sheet |
| **FAD Binding** | 32 | G F W | 403-405 | beta-sheet | G A W | 388-390 | beta-sheet |
| **site II** | 33 | K | 419 | alpha-helix | **missing** |  |  |
| (393-487 AA) | 34 | R M T | 427-429 | beta-sheet | R M T | 408-410 | beta-sheet |
|  | 35 | G L F A I | 433-437 | beta-sheet | G L F G A | 411-415 | beta-sheet |
|  | 36 | S S G S F T E G R I A A K A A V R F I L E Q | 449-470 | alpha-helix | S S G S F T E G R L A A K A A C K Y I D D G **loop elongated** | 430-451 | alpha-helix |
|  | 37 | D A V V E E L K K K | 478-487 | alpha-helix | D K Q I A E R K E Q | 460-469 | alpha-helix |
| Helical | 38 | P M E R F M Q | 491-497 | alpha-helix | P L E N Y T I | 473-479 | alpha-helix |
| (488-643 AA) | 39 | P W Q G L V R L Q K I M D E | 514-527 | alpha-helix | P M S G L Q R L Q K L M D E | 496-509 | alpha-helix |
|  | 40 | Y K T | 536-538 | beta-sheet | Y V T | 518-520 | beta-sheet |
|  | 41 | E K M L L Q R A L E L L A F L K E D L | 540-557 | alpha-helix | D K L L N I G L K K L A I L E E D L | 522-539 | alpha-helix |
|  | 42 | L H E L M R A W E L V H R V W T A E A H V R H M L F | 565-590 | alpha-helix | F H Q L M R G W E L R H R H R T S E C V T Q H T L F | 547-572 | alpha-helix |
|  | 43 | Y R T | 601-603 | beta-sheet | Y R G | 583-585 | beta-sheet |
|  | 44 | K C F V C S K Y D | 614-622 | beta-sheet | H V L T V S H R D | 593-601 | beta-sheet |
|  | 45 | E W T F E K V P Y | 627-635 | beta-sheet | K Y T L E K A P C | 609-617 | beta-sheet |
|  |  |  |  |  |  |  |  |

**Structural deviations:** 32-35 loop **625 AA total** **Protein problems: backbone**

66-69 loop-beta **identical AA 298** 47,7% Asn35, Ala44, Ala124, Lys154, Val155, Asp253, Glu278, Ser286,

117-123 loop **similar AA** 419** 67,0% Val289, His338, Pro370, His383*, Phe429*, Val458

151-154 loop **1.0Ǻ RMSD Bb 583 93,3%**

281-289 beta-loop-beta ** incl. identical AA

- 1. loop

399-405 alpha-helix missing

455-457 loop

# Secondary structure element succession AprA *Pelagibacter ubique*

| **AprA domains** | **No** | ***Archaeoglobus fulgidus* sequence** | AA position | **Sec. str. element** | *Pelagibacter ubique*  sequence | AA position | **Sec. str. element** |
| --- | --- | --- | --- | --- | --- | --- | --- |
| FAD-Binding | 1 | P T E V V E T D I L I I G | 17-29 | beta-sheet | K T H F E D C D V L V V G | 5-17 | beta-sheet |
| **site I** | 2 | F S G C G G A A Y E A A Y W A K | 32-44 | alpha-helix | M A G T G A T F E A R H W→ **shortened** | 20-32 | alpha-helix |
| (2-261 AA) | 3 | K V T L V E | 51-56 | beta-sheet | K I I C V E | 39-42 | beta-sheet |
|  | 4 | S A I | 71-73 | beta-sheet | Y A I **loop shortened** | 57-59 | beta-sheet |
|  | 5 | L E D Y V R Y V T L D M | 89-100 | alpha-helix | P E D H V R Y A R N D L | 72-83 | alpha-helix |
|  | 6 | E D L V A D Y A R H V D G T V H L F E K | 106-125 | alpha-helix | E D L G Y D M A R H V D S T V H M F D E W | 89-109 | alpha-helix |
|  | 7 | P I W K T | 129-133 | beta-sheet | P M M K N **loop elongated** | 112-116 | beta-sheet |
|  | 8 | K Y V R E | 137-141 | beta-sheet | R Y L R E | 121-125 | beta-sheet |
|  | 9 | Q I M I H | 145-149 | beta-sheet | Q I M I H | 129-134 | beta-sheet |
|  | 10 | Y K P I I A E A A K M A V | 153-165 | alpha-helix | Y K P I V A E A A K K A**→** **shortened** | 137-148 | alpha-helix |
|  | 11 | N I Y E R V F I F E L L K D | 169-182 | beta-sheet | **→**Y N R I M I T H L L M D **shortened** | 151-164 | beta-sheet |
|  | 12 | A V A G A V G F S V | 188-197 | beta-sheet | R V G G A V G F N M | 170-179 | beta-sheet |
|  | 13 | K F Y V F K A | 201-207 | beta-sheet | D F H V F R A | 183-189 | beta-sheet |
|  | 14 | A V I L A | 209-213 | beta-sheet | T V I V A | 191-195 | beta-sheet |
|  | 15 | G A T | 216-218 | beta-sheet | G A S | 198-200 | beta-sheet |
|  | 16 | A A | 229-230 | alpha-helix | G M | 211-212 | alpha-helix |
|  | 17 | D T G | 239-241 | beta- sheet | S N G | 221-223 | beta- sheet |
|  | 18 | S G Y Y M G L K A | 242-250 | alpha-helix | S A Y A L P I A V | 224-232 | alpha-helix |
|  | 19 | M L T Q | 253-256 | beta-sheet | K M T Q | 235-238 | beta-sheet |
| Capping | 20 | P F R F K | 263-267 | beta-sheet | L C R F K | 245-249 | beta-sheet |
| (262-393 AA) | 21 | G A W F L F | 274-279 | alpha-helix | G A Y F L H | 256-261 | alpha-helix |
|  | 22 | K A K N A | 283-287 | beta-sheet | Y T Q N A | 265-269 | beta-sheet |
|  | 23 | I K | 293-294 | alpha-helix | **missing** |  |  |
|  | 24 | A | 298 | alpha-helix | missing |  |  |
|  | 25 | Y G | 306-307 | alpha-helix | **missing** |  |  |
|  | 26 | T P L R N H Q V M L E I M D | 314-327 | alpha-helix | T C L R N H A F I Q E T I A | 297-310 | alpha-helix |
|  | 27 | P I M Y M H | 331-335 | beta-sheet | P I H M V | 314-318 | beta-sheet |
|  | 28 | T E E A L A E L A | 336-344 | alpha-helix | T T E A→ **shortened** | 319-322 | alpha-helix |
|  | 29 | K K K L K H I Y E E A F E D F L | 348-363 | alpha-helix | →L E T V G W E N F L **shortened** | 328-337 | alpha-helix |
|  | 30 | S Q A L L W A C Q | 368-375 | alpha-helix | G Q A V V W A S Q | 342-350 | alpha-helix |
|  | 31 | S E A A P A | 385-390 | beta-sheet | P E L T T S | 359-364 | beta-sheet |
| **FAD Binding** | 32 | G F W | 403-405 | beta-sheet | G A W | 377-379 | beta-sheet |
| **site II** | 33 | K | 419 | alpha-helix | **missing** |  |  |
| (393-487 AA) | 34 | R M T | 427-429 | beta-sheet | R M L | 397-399 | beta-sheet |
|  | 35 | G L F A I | 433-437 | beta-sheet | G L F G A | 403-407 | beta-sheet |
|  | 36 | S S G S F T E G R I A A K A A V R F I L E Q | 449-470 | alpha-helix | S S G S F T E G R L A A K A A V K Y I Q D K **loop elongated** | 419-440 | alpha-helix |
|  | 37 | D A V V E E L K K K | 478-487 | alpha-helix | D K Q C E D F K T A | 449-458 | alpha-helix |
| Helical | 38 | P M E R F M Q | 491-497 | alpha-helix | P L E T Y Q V | 462-468 | alpha-helix |
| (488-643 AA) | 39 | P W Q G L V R L Q K I M D E | 514-527 | alpha-helix | P I Q G L Q R L Q R I M D E | 485-498 | alpha-helix |
|  | 40 | Y K T | 536-538 | beta-sheet | Y M V | 507-509 | beta-sheet |
|  | 41 | E K M L L Q R A L E L L A F L K E D L | 540-557 | alpha-helix | G N M L K R G L E L L A W L E E D L | 511-528 | alpha-helix |
|  | 42 | L H E L M R A W E L V H R V W T A E A H V R H M L F | 565-590 | alpha-helix | L H Q L M R A W E L K H R A L T S Q C V T E H T M F | 536-561 | alpha-helix |
|  | 43 | Y R T | 601-603 | beta-sheet | Y R G | 572-574 | beta-sheet |
|  | 44 | K C F V C S K Y D | 614-622 | beta-sheet | H C L T V S R R D | 585-593 | beta-sheet |
|  | 45 | E W T F E K V P Y | 627-635 | beta-sheet | K F S L E K V P V | 598-606 | beta-sheet |
|  |  |  |  |  |  |  |  |

**Structural deviations:** 32-35 loop **614 AA total** **Protein problems: backbone**

66-69 loop **identical AA 309** 50,3% Met36, Ala44, Asp250, Glu288,

117-119 loop **similar AA** 431** 70,2% Pro359, His372*, Phe418* 148-151 loop **1.0Ǻ RMSD Bb 572 93,2%**

278-286 loop ** incl. identical AA

- 1. loop

388-394 alpha-helix missing

444-446 loop

# Secondary structure element succession AprA Environmental sequence EBAC2C11

| **AprA domains** | **No** | ***Archaeoglobus fulgidus* sequence** | AA position | **Sec. str. element** | Environmental sequence EBAC2C11 sequence | AA position | **Sec. str. element** |
| --- | --- | --- | --- | --- | --- | --- | --- |
| FAD-Binding | 1 | P T E V V E T D I L I I G | 17-29 | beta-sheet | K T V F V D S D I L V I G | 5-17 | beta-sheet |
| **site I** | 2 | F S G C G G A A Y E A A Y W A K | 32-44 | alpha-helix | F G G C G A A Y E S R Y W→ **shortened** | 20-32 | alpha-helix |
| (2-261 AA) | 3 | K V T L V E | 51-56 | beta-sheet | K V V V V E | 39-42 | beta-sheet |
|  | 4 | S A I | 71-73 | beta-sheet | Y A I **loop shortened** | 57-59 | beta-sheet |
|  | 5 | L E D Y V R Y V T L D M | 89-100 | alpha-helix | P E D Y V R Y Q R N D L | 72-83 | alpha-helix |
|  | 6 | E D L V A D Y A R H V D G T V H L F E K | 106-125 | alpha-helix | E D L G Y D I G R H V D S T V H K F E E **W verlängert** | 89-109 | alpha-helix |
|  | 7 | P I W K T | 129-133 | beta-sheet | P I M T D **loop elongated** | 112-116 | beta-sheet |
|  | 8 | K Y V R E | 137-141 | beta-sheet | R Y Q R E | 121-125 | beta-sheet |
|  | 9 | Q I M I H | 145-149 | beta-sheet | Q I M I H | 129-134 | beta-sheet |
|  | 10 | Y K P I I A E A A K M A V | 153-165 | alpha-helix | Y K P I V A E A A R K A**→** **shortened** | 137-148 | alpha-helix |
|  | 11 | N I Y E R V F I F E L L K D | 169-182 | beta-sheet | **→**A V Y N R I M V T H L L M D **shortened** | 151-164 | beta-sheet |
|  | 12 | A V A G A V G F S V | 188-197 | beta-sheet | R V A G A V G F N V | 170-179 | beta-sheet |
|  | 13 | K F Y V F K A | 201-207 | beta-sheet | D F Y V F Q S | 183-189 | beta-sheet |
|  | 14 | A V I L A | 209-213 | beta-sheet | A V I V A | 191-195 | beta-sheet |
|  | 15 | G A T | 216-218 | beta-sheet | G A S | 198-200 | beta-sheet |
|  | 16 | A A | 229-230 | alpha-helix | G M | 211-212 | alpha-helix |
|  | 17 | D T G | 239-241 | beta- sheet | S S A | 221-223 | beta- sheet |
|  | 18 | S G Y Y M G L K A | 242-250 | alpha-helix | S A Y A L P I R V | 224-232 | alpha-helix |
|  | 19 | M L T Q | 253-256 | beta-sheet | K M T Q | 235-238 | beta-sheet |
| Capping | 20 | P F R F K | 263-267 | beta-sheet | L T R F K | 245-249 | beta-sheet |
| (262-393 AA) | 21 | G A W F L F | 274-279 | alpha-helix | G A Y F L H | 256-261 | alpha-helix |
|  | 22 | K A K N A | 283-287 | beta-sheet | Y T Q N G | 265-269 | beta-sheet |
|  | 23 | I K | 293-294 | alpha-helix | **missing** |  |  |
|  | 24 | A | 298 | alpha-helix | M V G D Y V N H alpha-helix elongated | 285-292 | alpha-helix |
|  | 25 | Y G | 306-307 | alpha-helix | **missing** |  |  |
|  | 26 | T P L R N H Q V M L E I M D | 314-327 | alpha-helix | T C L R N H A F L K E V E A | 297-310 | alpha-helix |
|  | 27 | P I M Y M H | 331-335 | beta-sheet | P I R M V | 314-318 | beta-sheet |
|  | 28 | T E E A L A E L A | 336-344 | alpha-helix | T K E A→ **shortened** | 319-322 | alpha-helix |
|  | 29 | K K K L K H I Y E E A F E D F L | 348-363 | alpha-helix | →K E E V G W E N F L **shortened** | 328-337 | alpha-helix |
|  | 30 | S Q A L L W A C Q | 368-375 | alpha-helix | G Q A V V W A A N | 342-350 | alpha-helix |
|  | 31 | S E A A P A | 385-390 | beta-sheet | P E L V M S | 359-364 | beta-sheet |
| **FAD Binding** | 32 | G F W | 403-405 | beta-sheet | G A W | 377-379 | beta-sheet |
| **site II** | 33 | K | 419 | alpha-helix | **missing** |  | alpha-helix |
| (393-487 AA) | 34 | R M T | 427-429 | beta-sheet | R M M | 397-399 | beta-sheet |
|  | 35 | G L F A I | 433-437 | beta-sheet | G L F G A | 403-407 | beta-sheet |
|  | 36 | S S G S F T E G R I A A K A A V R F I L E Q | 449-470 | alpha-helix | S S G S F T E G R L A G K A A N K Y V D D → **shortened, loop elongated** | 419-439 | alpha-helix |
|  | 37 | D A V V E E L K K K | 478-487 | alpha-helix | E E E Y L E L K E Q | 449-458 | alpha-helix |
| Helical | 38 | P M E R F M Q | 491-497 | alpha-helix | P L E T Y R V | 462-468 | alpha-helix |
| (488-643 AA) | 39 | P W Q G L V R L Q K I M D E | 514-527 | alpha-helix | P L S G L Q R L E K I M D E | 485-498 | alpha-helix |
|  | 40 | Y K T | 536-538 | beta-sheet | Y M V | 507-509 | beta-sheet |
|  | 41 | E K M L L Q R A L E L L A F L K E D L | 540-557 | alpha-helix | E P L M T R G I E L L K M L K E D L | 511-528 | alpha-helix |
|  | 42 | L H E L M R A W E L V H R V W T A E A H V R H M L F | 565-590 | alpha-helix | L H Q L Q R A W E L H H R V L A S E C V T A H T M F | 536-561 | alpha-helix |
|  | 43 | Y R T | 601-603 | beta-sheet | Y R G | 572-574 | beta-sheet |
|  | 44 | K C F V C S K Y D | 614-622 | beta-sheet | H C F T L S Q Y D | 585-593 | beta-sheet |
|  | 45 | E W T F E K V P Y | 627-635 | beta-sheet | E F E M E K A P V | 598-606 | beta-sheet |
|  |  |  |  |  |  |  |  |

**Structural deviations:** 32-35 loop **613 AA total** **Protein problems: backbone**

66-69 loop **identical AA 316** 51,5% Ala44, Thr119, Asp250, Val295,

117-119 loop **similar AA** 426** 69,5% Pro326, His327, Pro359, His372, Phe418, 148-151 loop **1.0Ǻ RMSD Bb 570 93,0%** Thr442, Asn443

278-286 loop ** incl. identical AA

- 1. loop

388-394 alpha-helix missing

440-442 loop

# Crenarchaeal SRP

# Secondary structure element succession AprA *Pyrobaculum calidifontis*

| **AprA domains** | **No** | ***Archaeoglobus fulgidus* sequence** | AA position | **Sec. str. element** | *Pyrobaculum calidifontis* sequence | AA position | **Sec. str. element** |
| --- | --- | --- | --- | --- | --- | --- | --- |
| FAD-Binding | 1 | P T E V V E T D I L I I G | 17-29 | beta-sheet | P T K V V E T D I L V V G | 7-19 | beta-sheet |
| **site I** | 2 | F S G C G G A A Y E A A Y W A K | 32-44 | alpha-helix | M A G C G A V F E A K Y → **shortened** | 22-33 | alpha-helix |
| (2-261 AA) | 3 | K V T L V E | 51-56 | beta-sheet | K V T L V E | 30-45 | beta-sheet |
|  | 4 | S A I | 71-73 | beta-sheet | S A T **loop shortened** | 60-62 | beta-sheet |
|  | 5 | L E D Y V R Y V T L D M | 89-100 | alpha-helix | P E E F V R Y V R N D → **shortened** | 76-86 | alpha-helix |
|  | 6 | E D L V A D Y A R H V D G T V H L F E K | 106-125 | alpha-helix | E D L V Y D I A R H M T S T I K L F D M **W verlängert** | 93-113 | alpha-helix |
|  | 7 | P I W K T | 129-133 | beta-sheet | P I W R D **loop elongated** | 116-120 | beta-sheet |
|  | 8 | K Y V R E | 137-141 | beta-sheet | K Y L R T | 125-129 | beta-sheet |
|  | 9 | Q I M I H | 145-149 | beta-sheet | Q H P I H | 133-137 | beta-sheet |
|  | 10 | Y K P I I A E A A K M A V | 153-165 | alpha-helix | Y K A I I A E P C R K **→** **shortened**. | 141-151 | alpha-helix |
|  | 11 | N I Y E R V F I F E L L K D | 169-182 | beta-sheet | **→**Y E R V F V T H P L L D **shortened** | 157-168 | beta-sheet |
|  | 12 | A V A G A V G F S V | 188-197 | beta-sheet | R I A G V V G F H V | 174-183 | beta-sheet |
|  | 13 | K F Y V F K A | 201-207 | beta-sheet | T F Y V F K A | 187-193 | beta-sheet |
|  | 14 | A V I L A | 209-213 | beta-sheet | A V I V A | 195-199 | beta-sheet |
|  | 15 | G A T | 216-218 | beta-sheet | G T S | 202-204 | beta-sheet |
|  | 16 | A A | 229-230 | alpha-helix | G L | 215-216 | alpha-helix |
|  | 17 | D T G | 239-241 | beta- sheet | A S G | 225-227 | beta- sheet |
|  | 18 | S G Y Y M G L K A | 242-250 | alpha-helix | S A Y A I P L L A | 228-236 | alpha-helix |
|  | 19 | M L T Q | 253-256 | beta-sheet | E T V N | 239-242 | beta-sheet |
| Capping | 20 | P F R F K | 263-267 | beta-sheet | V V R F K | 249-253 | beta-sheet |
| (262-393 AA) | 21 | G A W F L F | 274-279 | alpha-helix | G F P Y L L | 260-265 | alpha-helix |
|  | 22 | K A K N A | 283-287 | beta-sheet | R S T D V | 269-273 | beta-sheet |
|  | 23 | I K | 293-294 | alpha-helix | **missing** |  |  |
|  | 24 | A | 298 | alpha-helix | E | 288 | alpha-helix |
|  | 25 | Y G | 306-307 | alpha-helix | Y A | 296-297 | alpha-helix |
|  | 26 | T P L R N H Q V M L E I M D | 314-327 | alpha-helix | T P I R T W V T I Q N L K E | 304-317 | alpha-helix |
|  | 27 | P I M Y M H | 331-335 | beta-sheet | P D I M Q | 321-325 | beta-sheet |
|  | 28 | T E E A L A E L A | 336-344 | alpha-helix | **missing** |  |  |
|  | 29 | K K K L K H I Y E E A F E D F L | 348-363 | alpha-helix | **missing** |  |  |
|  | 30 | S Q A L L W A C Q | 368-375 | alpha-helix | T Q V I Y W A S Q | 349-357 | alpha-helix |
|  | 31 | S E A A P A | 385-390 | beta-sheet | S E L L P T | 366-371 | beta-sheet |
| **FAD Binding** | 32 | G F W | 403-405 | beta-sheet | G M W | 384-386 | beta-sheet |
| **site II** | 33 | K | 419 | alpha-helix | **missing** |  |  |
| (393-487 AA) | 34 | R M T | 427-429 | beta-sheet | R M L | 404-406 | beta-sheet |
|  | 35 | G L F A I | 433-437 | beta-sheet | G L F G A | 410-414 | beta-sheet |
|  | 36 | S S G S F T E G R I A A K A A V R F I L E Q | 449-470 | alpha-helix | S S G S F T E G R I A G K S A A R Y V L T Q | 426-447 | alpha-helix |
|  | 37 | D A V V E E L K K K | 478-487 | alpha-helix | N D T I E R Y K E I | 457-466 | alpha-helix |
| Helical | 38 | P M E R F M Q | 491-497 | alpha-helix | P L E W Y H K | 470-476 | alpha-helix |
| (488-643 AA) |  |  |  |  | **T P (new)** | 483-484 | **alpha-Helix** |
|  | 39 | P W Q G L V R L Q K I M D E | 514-527 | alpha-helix | W Y Q L L T R L Q K I M D E | 503-516 | alpha-helix |
|  | 40 | Y K T | 536-538 | beta-sheet | Y T T | 525-527 | beta-sheet |
|  | 41 | E K M L L Q R A L E L L A F L K E D L | 540-557 | alpha-helix | M Y M L G R A W E L L K M L E E D F | 529-546 | alpha-helix |
|  | 42 | L H E L M R A W E L V H R V W T A E A H V R H M L F | 565-590 | alpha-helix | L H E L L R V W E V Y H R L I V G Q A V V F S M M N | 553-579 | alpha-helix |
|  | 43 | Y R T | 601-603 | beta-sheet | F N A | 588-590 | beta-sheet |
|  | 44 | K C F V C S K Y D | 614-622 | beta-sheet | H V F T H V R R D | 601-609 | beta-sheet |
|  | 45 | E W T F E K V P Y | 627-635 | beta-sheet | Q W S F R T S P V | 614-622 | beta-sheet |
|  |  |  |  |  |  |  |  |

**Str. deviations:** 35-37 loop **628 AA total** **Protein problems: backbone**

62-66 loop **identical AA 299** 47,6% Ala47, Phe67, Ser68, Pro94, Glu155, 121-124 loop **similar AA** 425** 67,7% Asp254, His379, Phe425, Asp450, 152-155 loop **1.0Ǻ RMSD Bb 562 89,5%** Pro453, Val454, Pro487, Leu490, Ile496

- 1. loop ** incl. identical AA

327-341 loop

395-402 alpha-helix missing

- 1. loop
  2. alpha-loop
  3. loop

# Secondary structure element succession AprA *Caldivirga maquilingensis*

| **AprA domains** | **No** | ***Archaeoglobus fulgidus* sequence** | AA position | **Sec. str. element** | *Caldivirga maquilingensis* sequence | AA position | **Sec. str. element** |
| --- | --- | --- | --- | --- | --- | --- | --- |
| FAD-Binding | 1 | P T E V V E T D I L I I G | 17-29 | beta-sheet | R V K Q V D S D I L I I G | 5-17 | beta-sheet |
| **site I** | 2 | F S G C G G A A Y E A A Y W A K | 32-44 | alpha-helix | M A G T G A A W E A K Y W → **shortened** | 20-32 | alpha-helix |
| (2-261 AA) | 3 | K V T L V E | 51-56 | beta-sheet | R I V L A E | 37-42 | beta-sheet |
|  | 4 | S A I | 71-73 | beta-sheet | S A I **loop shortened** | 57-59 | beta-sheet |
|  | 5 | L E D Y V R Y V T L D M | 89-100 | alpha-helix | V E D Y V K Y V R G D L | 72-83 | alpha-helix |
|  | 6 | E D L V A D Y A R H V D G T V H L F E K | 106-125 | alpha-helix | E D L V Y D Y A R H V D S T V H L F D E W | 89-109 | alpha-helix |
|  | 7 | P I W K T | 129-133 | beta-sheet | P I W P A | 112-116 | beta-sheet |
|  | 8 | K Y V R E | 137-141 | beta-sheet | C Y V R E | 120-124 | beta-sheet |
|  | 9 | Q I M I H | 145-149 | beta-sheet | Q I M I H | 128-132 | beta-sheet |
|  | 10 | Y K P I I A E A A K M A V | 153-165 | alpha-helix | Y K A I V A E A A R K A I | 136-148 | alpha-helix |
|  | 11 | N I Y E R V F I F E L L K D | 169-182 | beta-sheet | N I M N R V M V T H L L K S | 152-165 | beta-sheet |
|  | 12 | A V A G A V G F S V | 188-197 | beta-sheet | R V A G A L G F N V | 171-180 | beta-sheet |
|  | 13 | K F Y V F K A | 201-207 | beta-sheet | T I Y V F K A | 184-190 | beta-sheet |
|  | 14 | A V I L A | 209-213 | beta-sheet | A I I M A | 192-196 | beta-sheet |
|  | 15 | G A T | 216-218 | beta-sheet | G G S | 199-201 | beta-sheet |
|  | 16 | A A | 229-230 | alpha-helix | G L | 212-213 | alpha-helix |
|  | 17 | D T G | 239-241 | beta- sheet | S S A | 222-224 | beta- sheet |
|  | 18 | S G Y Y M G L K A | 242-250 | alpha-helix | S S Y G M M I E S | 225-233 | alpha-helix |
|  | 19 | M L T Q | 253-256 | beta-sheet | V M T M | 236-239 | beta-sheet |
| Capping | 20 | P F R F K | 263-267 | beta-sheet | V P R F K | 246-250 | beta-sheet |
| (262-393 AA) | 21 | G A W F L F | 274-279 | alpha-helix | G A Y Q L M | 257-262 | alpha-helix |
|  | 22 | K A K N A | 283-287 | beta-sheet | R I S N V | 266-270 | beta-sheet |
|  | 23 | I K | 293-294 | alpha-helix | **missing** |  |  |
|  | 24 | A | 298 | alpha-helix | missing |  |  |
|  | 25 | Y G | 306-307 | alpha-helix | **missing** |  |  |
|  | 26 | T P L R N H Q V M L E I M D | 314-327 | alpha-helix | →T L R V Y A Q R L E W I N **shortened** | 302-314 | alpha-helix |
|  | 27 | P I M Y M H | 331-335 | beta-sheet | P S L M R | 318-322 | beta-sheet |
|  | 28 | T E E A L A E L A | 336-344 | alpha-helix | T D E T V K K G → **shortened** | 323-330 | alpha-helix |
|  | 29 | K K K L K H I Y E E A F E D F L | 348-363 | alpha-helix | → E D F L **shortened** | 339-342 | alpha-helix |
|  | 30 | S Q A L L W A C Q | 368-375 | alpha-helix | S Q V A I W A G Q | 347-352 | alpha-helix |
|  | 31 | S E A A P A | 385-390 | beta-sheet | Y E V I T T | 364-369 | beta-sheet |
| **FAD Binding** | 32 | G F W | 403-405 | beta-sheet | G A W | 382-384 | beta-sheet |
| **site II** | 33 | K | 419 | alpha-helix | **missing** |  |  |
| (393-487 AA) | 34 | R M T | 427-429 | beta-sheet | R M T | 413-415 | beta-sheet |
|  | 35 | G L F A I | 433-437 | beta-sheet | G L F C A | 419-423 | beta-sheet |
|  | 36 | S S G S F T E G R I A A K A A V R F I L E Q | 449-470 | alpha-helix | S S G S F T E G R L A A K S A V L Y L M D H | 435-456 | alpha-helix |
|  | 37 | D A V V E E L K K K | 478-487 | alpha-helix | K G Q V D S M I S K | 465-474 | alpha-helix |
| Helical | 38 | P M E R F M Q | 491-497 | alpha-helix | P L E R W E E **loop elongated** | 478-484 | alpha-helix |
| (488-643 AA) | 39 | P W Q G L V R L Q K I M D E | 514-527 | alpha-helix | W K Q G L F R L Q K I M D E | 506-519 | alpha-helix |
|  | 40 | Y K T | 536-538 | beta-sheet | Y T T | 528-530 | beta-sheet |
|  | 41 | E K M L L Q R A L E L L A F L K E D L | 540-557 | alpha-helix | E W M L N R A I E L L Q F L K E D F | 532-549 | alpha-helix |
|  | 42 | L H E L M R A W E L V H R V W T A E A H V R H M L F | 565-590 | alpha-helix | W H E L M R T W E L W H R I L T A E A V V R H M L F | 558-583 | alpha-helix |
|  | 43 | Y R T | 601-603 | beta-sheet | V R A | 594-596 | beta-sheet |
|  | 44 | K C F V C S K Y D | 614-622 | beta-sheet | H V F V N S R Y D | 607-615 | beta-sheet |
|  | 45 | E W T F E K V P Y | 627-635 | beta-sheet | R W E L W K V P V | 620-628 | beta-sheet |
|  |  |  |  |  |  |  |  |

**Str. deviations:** 33-35 loop **635 AA** **Protein problems: backbone**

66-69 loop **identical AA 336** 52,9 Ala44, Pro115, Asp251, Glu281, **similar AA** 444** 70,0 Ile283, Thr301, Phe338, His377, Phe434, **1.0Ǻ RMSD Bb 587 92,4%** Arg403, Asp462, Ile463, Pro498, Asp557

- 1. loop ** incl. identical AA

298-300 loop

- 1. loop

394-410 alpha helix missing

460-462 loop

490-497 loop

554-556 loop

# SRB and affiliated SOB Apr lineage II

# Secondary structure element succession AprA *Desulfotomaculum reducens*

| **AprA domains** | **No** | ***Archaeoglobus fulgidus* sequence** | AA position | **Sec. str. element** | *Desulfotomaculum reducens*  sequence | AA position | **Sec. str. element** |
| --- | --- | --- | --- | --- | --- | --- | --- |
| FAD-Binding | 1 | P T E V V E T D I L I I G | 17-29 | beta-sheet | E T V V V N T D I L I L G | 6-18 | beta-sheet |
| **site I** | 2 | F S G C G G A A Y E A A Y W A K | 32-44 | alpha-helix | M A A C G A A V E A A Y W A K | 21-35 | alpha-helix |
| (2-261 AA) | 3 | K V T L V E | 51-56 | beta-sheet | K I T L V D | 40-45 | beta-sheet |
|  | 4 | S A I | 71-73 | beta-sheet | S A I **loop shortened** | 60-62 | beta-sheet |
|  | 5 | L E D Y V R Y V T L D M | 89-100 | alpha-helix | V E D Y V K Y V K N D L | 74-85 | alpha-helix |
|  | 6 | E D L V A D Y A R H V D G T V H L F E K | 106-125 | alpha-helix | D D Q V A D I A R H V D S S V H L F E K **W verlängert** | 91-111 | alpha-helix |
|  | 7 | P I W K T | 129-133 | beta-sheet | P I W T N | 114-118 | beta-sheet |
|  | 8 | K Y V R E | 137-141 | beta-sheet | N Y V R G | 122-126 | beta-sheet |
|  | 9 | Q I M I H | 145-149 | beta-sheet | Q I M L N | 130-134 | beta-sheet |
|  | 10 | Y K P I I A E A A K M A V | 153-165 | alpha-helix | Y K I I V A E A A K N A L | 138-150 | alpha-helix |
|  | 11 | N I Y E R V F I F E L L K D | 169-182 | beta-sheet | N I Y E R V Y I C E P I M D **loop shortened** | 154-167 | beta-sheet |
|  | 12 | A V A G A V G F S V | 188-197 | beta-sheet | R I A G A I G F S T | 170-179 | beta-sheet |
|  | 13 | K F Y V F K A | 201-207 | beta-sheet | K V Y V F K A | 183-189 | beta-sheet |
|  | 14 | A V I L A | 209-213 | beta-sheet | A V L A S | 191-195 | beta-sheet |
|  | 15 | G A T | 216-218 | beta-sheet | G A V | 198-200 | beta-sheet |
|  | 16 | A A | 229-230 | alpha-helix | G L | 211-212 | alpha-helix |
|  | 17 | D T G | 239-241 | beta- sheet | S S G | 221-223 | beta- sheet |
|  | 18 | S G Y Y M G L K A | 242-250 | alpha-helix | S S A Y F T M V A | 224-232 | alpha-helix |
|  | 19 | M L T Q | 253-256 | beta-sheet | E M T C | 235-238 | beta-sheet |
| Capping | 20 | P F R F K | 263-267 | beta-sheet | P V R F K | 245-249 | beta-sheet |
| (262-393 AA) | 21 | G A W F L F | 274-279 | alpha-helix | G A W F L L | 256-261 | alpha-helix |
|  | 22 | K A K N A | 283-287 | beta-sheet | R A V N G | 265-269 | beta-sheet |
|  | 23 | I K | 293-294 | alpha-helix | **Y** M V **alpha-helix elongated** | 274-276 | alpha-helix |
|  | 24 | A | 298 | alpha-helix | P | 280 | alpha-helix |
|  | 25 | Y G | 306-307 | alpha-helix | Y G | 288-289 | alpha-helix |
|  | 26 | T P L R N H Q V M L E I M D | 314-327 | alpha-helix | A N L R N Y L G M L D V S D | 296-309 | alpha-helix |
|  | 27 | P I M Y M H | 331-335 | beta-sheet | P L F M E | 313-317 | beta-sheet |
|  | 28 | T E E A L A E L A | 336-344 | alpha-helix | T D E A I A N L A **loop elongated** | 318-326 | alpha-helix |
|  | 29 | K K K L K H I Y E E A F E D F L | 348-363 | alpha-helix | K K K M K E L E N E A W E D F L | 337-352 | alpha-helix |
|  | 30 | S Q A L L W A C Q | 368-375 | alpha-helix | A Q A I L W A A S | 357-365 | alpha-helix |
|  | 31 | S E A A P A | 385-390 | beta-sheet | S E I A A A | 374-379 | beta-sheet |
| **FAD Binding** | 32 | G F W | 403-405 | beta-sheet | G A W | 392-394 | beta-sheet |
| **site II** | 33 | K | 419 | alpha-helix | **missing** |  |  |
| (393-487 AA) | 34 | R M T | 427-429 | beta-sheet | N M T | 412-414 | beta-sheet |
|  | 35 | G L F A I | 433-437 | beta-sheet | G L F A A | 418-422 | beta-sheet |
|  | 36 | S S G S F T E G R I A A K A A V R F I L E Q | 449-470 | alpha-helix | S S G S H A E G R I A G K S M V R F V V E N | 434-455 | alpha-helix |
|  | 37 | D A V V E E L K K K | 478-487 | alpha-helix | D A K V E E L K A K | 463-472 | alpha-helix |
| Helical | 38 | P M E R F M Q | 491-497 | alpha-helix | P L A V W E E | 476-482 | alpha-helix |
| (488-643 AA) | 39 | P W Q G L V R L Q K I M D E | 514-527 | alpha-helix | P K M Y M F R L Q K M M D E | 499-512 | alpha-helix |
|  | 40 | Y K T | 536-538 | beta-sheet | F T T | 521-523 | beta-sheet |
|  | 41 | E K M L L Q R A L E L L A F L K E D L | 540-557 | alpha-helix | K T L L E V G L E L L T M L K E D S | 525-542 | alpha-helix |
|  | 42 | L H E L M R A W E L V H R V W T A E A H V R H M L F | 565-590 | alpha-helix | L H E L M R A W E N I H R T L Q A E S H I R T V L F | 550-575 | alpha-helix |
|  | 43 | Y R T | 601-603 | beta-sheet | F R A | 586-588 | beta-sheet |
|  | 44 | K C F V C S K Y D | 614-622 | beta-sheet | E V F C N C K Y D | 698-606 | beta-sheet |
|  | 45 | E W T F E K V P Y | 627-635 | beta-sheet | E W E M I K R P I | 611-619 | beta-sheet |
|  |  |  |  |  |  |  |  |

**Str. deviations:** 68-71 loop **624 AA** **Protein problems: backbone**

**identical AA 359** 57,5% Pro2, Ala46, Pro114, Pro164, Asp250, Pro287,

166-171 loop **similar AA** 478** 76,6% His387, Phe433, Pro596, Asp596

328-336 loop **1.0Ǻ RMSD Bb 598 95,8%**

403-409 alpha-helix missing ** incl. identical AA

593-595 loop

# Secondary structure element succession AprA *Synthrophobacter fumarooxidans*

| **AprA domains** | **No** | ***Archaeoglobus fulgidus* sequence** | AA position | **Sec. str. element** | *Synthrophobacter fumarooxidans*  sequence | AA position | **Sec. str. element** |
| --- | --- | --- | --- | --- | --- | --- | --- |
| FAD-Binding | 1 | P T E V V E T D I L I I G | 17-29 | beta-sheet | E T V V V E T D L L I I G | 5-17 | beta-sheet |
| **site I** | 2 | F S G C G G A A Y E A A Y W A K | 32-44 | alpha-helix | M S A C G A A F E A A Y W A K | 20-34 | alpha-helix |
| (2-261 AA) | 3 | K V T L V E | 51-56 | beta-sheet | K V T V V D | 39-44 | beta-sheet |
|  | 4 | S A I | 71-73 | beta-sheet | S A I | 59-61 | beta-sheet |
|  |  |  |  |  | I G Y (new) | 65-67 | **beta-sheet** |
|  |  |  |  |  | G E N (new) | 70-72 | **beta-sheet** |
|  | 5 | L E D Y V R Y V T L D M | 89-100 | alpha-helix | L E D Y V K Y V R Q D L | 74-85 | alpha-helix |
|  | 6 | E D L V A D Y A R H V D G T V H L F E K | 106-125 | alpha-helix | E D L V Y N I A R H V D S S V H L F E K W | 91-111 | alpha-helix |
|  | 7 | P I W K T | 129-133 | beta-sheet | P I W K D | 115-119 | beta-sheet |
|  | 8 | K Y V R E | 137-141 | beta-sheet | K Y V H E | 122-126 | beta-sheet |
|  | 9 | Q I M I H | 145-149 | beta-sheet | Q I M I N | 130-134 | beta-sheet |
|  | 10 | Y K P I I A E A A K M A V | 153-165 | alpha-helix | Y K V L V A E A A K N A **→ shortened** | 138-149 | alpha-helix |
|  | 11 | N I Y E R V F I F E L L K D | 169-182 | beta-sheet | E L L E R V F I V E P L → **shortened** | 158-169 | beta-sheet |
|  | 12 | A V A G A V G F S V | 188-197 | beta-sheet | →V G G V G F S V **shortened** | 176-183 | beta-sheet |
|  | 13 | K F Y V F K A | 201-207 | beta-sheet | K F Y V I K A | 187-193 | beta-sheet |
|  | 14 | A V I L A | 209-213 | beta-sheet | A T I A A | 195-199 | beta-sheet |
|  | 15 | G A T | 216-218 | beta-sheet | G A V | 202-204 | beta-sheet |
|  | 16 | A A | 229-230 | alpha-helix | G L | 215-216 | alpha-helix |
|  | 17 | D T G | 239-241 | beta- sheet | N A G | 225-227 | beta- sheet |
|  | 18 | S G Y Y M G L K A | 242-250 | alpha-helix | S T A Y F T I R A | 228-236 | alpha-helix |
|  | 19 | M L T Q | 253-256 | beta-sheet | E M T C | 239-242 | beta-sheet |
| Capping | 20 | P F R F K | 263-267 | beta-sheet | P V R F K | 249-253 | beta-sheet |
| (262-393 AA) | 21 | G A W F L F | 274-279 | alpha-helix | G A W F L L | 260-265 | alpha-helix |
|  | 22 | K A K N A | 283-287 | beta-sheet | R A V N A | 269-273 | beta-sheet |
|  | 23 | I K | 293-294 | alpha-helix | **Y** M V **alpha-helix elongated** | 278-280 | alpha-helix |
|  | 24 | A | 298 | alpha-helix | P | 287 | alpha-helix |
|  | 25 | Y G | 306-307 | alpha-helix | Y G | 292-293 | alpha-helix |
|  | 26 | T P L R N H Q V M L E I M D | 314-327 | alpha-helix | A N L R N W L G M E D V M A | 300-313 | alpha-helix |
|  | 27 | P I M Y M H | 331-335 | beta-sheet | P I Y M K | 317-321 | beta-sheet |
|  | 28 | T E E A L A E L A | 336-344 | alpha-helix | T E E A I A N L A **loop elongated** | 322-330 | alpha-helix |
|  | 29 | K K K L K H I Y E E A F E D F L | 348-363 | alpha-helix | → K K M K E L E S E A W E D F L **shortened** | 342-356 | alpha-helix |
|  | 30 | S Q A L L W A C Q | 368-375 | alpha-helix | S Q A L L W A G S | 361-369 | alpha-helix |
|  | 31 | S E A A P A | 385-390 | beta-sheet | S E I A A A | 378-383 | beta-sheet |
| **FAD Binding** | 32 | G F W | 403-405 | beta-sheet | G A W | 396-398 | beta-sheet |
| **site II** | 33 | K | 419 | alpha-helix | K **loop elongated** | 412 | alpha-helix |
| (393-487 AA) | 34 | R M T | 427-429 | beta-sheet | N M T | 422-424 | beta-sheet |
|  | 35 | G L F A I | 433-437 | beta-sheet | A L F T C | 428-432 | beta-sheet |
|  | 36 | S S G S F T E G R I A A K A A V R F I L E Q | 449-470 | alpha-helix | S S G S H A E G R V A A K G A I A Y I L D N | 444-465 | alpha-helix |
|  | 37 | D A V V E E L K K K | 478-487 | alpha-helix | D A K V E A L K K I | 473-482 | alpha-helix |
| Helical | 38 | P M E R F M Q | 491-497 | alpha-helix | P M D R F E E | 486-492 | alpha-helix |
| (488-643 AA) | 39 | P W Q G L V R L Q K I M D E | 514-527 | alpha-helix | P K M F M F R L Q K I M D E | 509-522 | alpha-helix |
|  | 40 | Y K T | 536-538 | beta-sheet | F T T | 531-533 | beta-sheet |
|  | 41 | E K M L L Q R A L E L L A F L K E D L | 540-557 | alpha-helix | K S L L T R G L E L L T M L K E D S | 535-552 | alpha-helix |
|  | 42 | L H E L M R A W E L V H R V W T A E A H V R H M L F | 565-590 | alpha-helix | L H E L L R A W E N T H R L W I A E S H L R H V M F | 560-585 | alpha-helix |
|  | 43 | Y R T | 601-603 | beta-sheet | Y R A | 596-598 | beta-sheet |
|  | 44 | K C F V C S K Y D | 614-622 | beta-sheet | K C F V N S V Y N | 609-617 | beta-sheet |
|  | 45 | E W T F E K V P Y | 627-635 | beta-sheet | E W T M K K V P V | 622-630 | beta-sheet |
|  |  |  |  |  |  |  |  |

**Str. deviations:** 68-71 beta-loop-beta **634 AA** **Protein problems: backbone**

150-155 loop **identical AA 385** 60,7% Pro2, Ala46, Pro114, Asp155, Lys156, Pro168, Cys175, Asp254, Pro291,

171-174 loop-beta **similarAA** 490** 77,3% Phe340, Lys341, His391, Val419, Phe443

332-340 loop **1.0Ǻ RMSD Bb 608 95,9%**

416-419 loop ** incl. identical AA

# Secondary structure element succession AprA fosws39f7 (M. Mussmann)

| **AprA domains** | **No** | ***Archaeoglobus fulgidus* sequence** | AA position | **Sec. str. element** | fosws39f7 sequence | AA position | **Sec. str. element** |
| --- | --- | --- | --- | --- | --- | --- | --- |
| FAD-Binding | 1 | P T E V V E T D I L I I G | 17-29 | beta-sheet | E T V E V T T D L L I L G | 6-18 | beta-sheet |
| **site I** | 2 | F S G C G G A A Y E A A Y W A K | 32-44 | alpha-helix | M S A C G A A V E A S Y W A K | 21-35 | alpha-helix |
| (2-261 AA) | 3 | K V T L V E | 51-56 | beta-sheet | K V T L V D | 40-45 | beta-sheet |
|  | 4 | S A I | 71-73 | beta-sheet | S A L **loop shortened** | 60-62 | beta-sheet |
|  | 5 | L E D Y V R Y V T L D M | 89-100 | alpha-helix | I K D Y V D Y V R M D | 75-85 | alpha-helix |
|  | 6 | E D L V A D Y A R H V D G T V H L F E K | 106-125 | alpha-helix | E D L V A N I A R H V D S S V H L F E K W | 92-112 | alpha-helix |
|  | 7 | P I W K T | 129-133 | beta-sheet | P I W T D | 115-119 | beta-sheet |
|  | 8 | K Y V R E | 137-141 | beta-sheet | R Y V H E | 123-127 | beta-sheet |
|  | 9 | Q I M I H | 145-149 | beta-sheet | Q L M I N | 131-135 | beta-sheet |
|  | 10 | Y K P I I A E A A K M A V | 153-165 | alpha-helix | Y K V V V A E A A K N A L A → **elongated** | 139-152 | alpha-helix |
|  | 11 | N I Y E R V F I F E L L K D | 169-182 | beta-sheet | E Y L E R I F I T E P L → **shortened** | 157-168 | beta-sheet |
|  | 12 | A V A G A V G F S V | 188-197 | beta-sheet | →A G A V G F S V **shortened** | 175-182 | beta-sheet |
|  | 13 | K F Y V F K A | 201-207 | beta-sheet | K F Y V F K A | 186-192 | beta-sheet |
|  | 14 | A V I L A | 209-213 | beta-sheet | A T I V A | 194-198 | beta-sheet |
|  | 15 | G A T | 216-218 | beta-sheet | G A V | 201-203 | beta-sheet |
|  | 16 | A A | 229-230 | alpha-helix | G L | 214-215 | alpha-helix |
|  | 17 | D T G | 239-241 | beta- sheet | N S G | 224-226 | beta- sheet |
|  | 18 | S G Y Y M G L K A | 242-250 | alpha-helix | S S A Y F T L R A | 227-235 | alpha-helix |
|  | 19 | M L T Q | 253-256 | beta-sheet | E M T S | 238-241 | beta-sheet |
| Capping | 20 | P F R F K | 263-267 | beta-sheet | P V R F K | 248-252 | beta-sheet |
| (262-393 AA) | 21 | G A W F L F | 274-279 | alpha-helix | G A W F L L | 259-264 | alpha-helix |
|  | 22 | K A K N A | 283-287 | beta-sheet | A A T N A | 268-272 | beta-sheet |
|  | 23 | I K | 293-294 | alpha-helix | **Y** M V **alpha-helix elongated** | 277-279 | alpha-helix |
|  | 24 | A | 298 | alpha-helix | E | 286 | alpha-helix |
|  | 25 | Y G | 306-307 | alpha-helix | Y G | 291-292 | alpha-helix |
|  | 26 | T P L R N H Q V M L E I M D | 314-327 | alpha-helix | A N L R N W L G M L D I M D | 299-312 | alpha-helix |
|  | 27 | P I M Y M H | 331-335 | beta-sheet | P I S M R | 316-320 | beta-sheet |
|  | 28 | T E E A L A E L A | 336-344 | alpha-helix | T E Q A I Q K I A **loop elongated** | 321-329 | alpha-helix |
|  | 29 | K K K L K H I Y E E A F E D F L | 348-363 | alpha-helix | →K K L K E L E S E A W E D F L **shortened** | 340-354 | alpha-helix |
|  | 30 | S Q A L L W A C Q | 368-375 | alpha-helix | S Q A I L W A A S | 359-367 | alpha-helix |
|  | 31 | S E A A P A | 385-390 | beta-sheet | S E I A A A | 376-381 | beta-sheet |
| **FAD Binding** | 32 | G F W | 403-405 | beta-sheet | G A W | 394-396 | beta-sheet |
| **site II** | 33 | K | 419 | alpha-helix | K | 410 | alpha-helix |
| (393-487 AA) | 34 | R M T | 427-429 | beta-sheet | H M A | 418-420 | beta-sheet |
|  | 35 | G L F A I | 433-437 | beta-sheet | G M F C A | 424-428 | beta-sheet |
|  | 36 | S S G S F T E G R I A A K A A V R F I L E Q | 449-470 | alpha-helix | S S G S H A E G R I V G K A A V K Y I T E N | 440-461 | alpha-helix |
|  | 37 | D A V V E E L K K K | 478-487 | alpha-helix | G G Q V E E I K G R | 469-478 | alpha-helix |
| Helical | 38 | P M E R F M Q | 491-497 | alpha-helix | P L E T F E T | 482-488 | alpha-helix |
| (488-643 AA) | 39 | P W Q G L V R L Q K I M D E | 514-527 | alpha-helix | P K M F M F R L Q K I M D E | 505-518 | alpha-helix |
|  | 40 | Y K T | 536-538 | beta-sheet | F S T | 527-529 | beta-sheet |
|  | 41 | E K M L L Q R A L E L L A F L K E D L | 540-557 | alpha-helix | K A C L D R A L E L L T M L S E D S | 531-548 | alpha-helix |
|  | 42 | L H E L M R A W E L V H R V W T A E A H V R H M L F | 565-590 | alpha-helix | L H E L M R C W E N V Q R M W Q A E A H T R T I L F | 556-581 | alpha-helix |
|  | 43 | Y R T | 601-603 | beta-sheet | R R A | 592-594 | beta-sheet |
|  | 44 | K C F V C S K Y D | 614-622 | beta-sheet | H A F A N C R W D | 605-613 | beta-sheet |
|  | 45 | E W T F E K V P Y | 627-635 | beta-sheet | D W E M I K R P M | 618-626 | beta-sheet |
|  |  |  |  |  |  |  |  |

**Str. deviations:** 69-72 loop **634 AA** **Protein problems: backbone**

151-156 helix-loop **identical AA 361** 56,9 Pro2, Ala59, Pro115, Glu157, Pro167, Asp253, Pro290, Lys339,

169-175 loop-beta **similar AA** 480** 75,7 His389, Phe439, Pro465, Pro633

331-338 loop **1.0Ǻ RMSD Bb 607 95,7%**

- 1. loop ** incl. identical AA

# Secondary structure element succession AprA fosws7f8 (M. Mussmann)

| **AprA domains** | **No** | ***Archaeoglobus fulgidus* sequence** | AA position | **Sec. str. element** | fosws7f8 sequence | AA position | **Sec. str. element** |
| --- | --- | --- | --- | --- | --- | --- | --- |
| FAD-Binding | 1 | P T E V V E T D I L I I G | 17-29 | beta-sheet | E T V T V E T D L L L A G | 5-17 | beta-sheet |
| **site I** | 2 | F S G C G G A A Y E A A Y W A K | 32-44 | alpha-helix | M A A C G A A V E A S Y W A K | 20-34 | alpha-helix |
| (2-261 AA) | 3 | K V T L V E | 51-56 | beta-sheet | K V T L V D | 39-44 | beta-sheet |
|  | 4 | S A I | 71-73 | beta-sheet | S A I **loop shortened** | 59-61 | beta-sheet |
|  | 5 | L E D Y V R Y V T L D M | 89-100 | alpha-helix | L K D Y C D Y V R N D L | 74-85 | alpha-helix |
|  | 6 | E D L V A D Y A R H V D G T V H L F E K | 106-125 | alpha-helix | E D Q V A N I A R H V D S T V H L F E K W | 91-111 | alpha-helix |
|  | 7 | P I W K T | 129-133 | beta-sheet | P I W T D | 114-118 | beta-sheet |
|  | 8 | K Y V R E | 137-141 | beta-sheet | N Y V H E | 122-126 | beta-sheet |
|  | 9 | Q I M I H | 145-149 | beta-sheet | Q L M I N | 130-134 | beta-sheet |
|  | 10 | Y K P I I A E A A K M A V | 153-165 | alpha-helix | Y K I I V A E A A K N A→ **shortened** | 138-150 | alpha-helix |
|  | 11 | N I Y E R V F I F E L L K D | 169-182 | beta-sheet | E Y F E R V F L T H P L **loop shortened** | 156-167 | beta-sheet |
|  | 12 | A V A G A V G F S V | 188-197 | beta-sheet | →V G A I G F S V **shortened** | 174-181 | beta-sheet |
|  | 13 | K F Y V F K A | 201-207 | beta-sheet | K F Y V F K A | 185-191 | beta-sheet |
|  | 14 | A V I L A | 209-213 | beta-sheet | A V L C A | 193-197 | beta-sheet |
|  | 15 | G A T | 216-218 | beta-sheet | G A V | 200-202 | beta-sheet |
|  | 16 | A A | 229-230 | alpha-helix | G F | 213-214 | alpha-helix |
|  | 17 | D T G | 239-241 | beta- sheet | N S G | 222-225 | beta- sheet |
|  | 18 | S G Y Y M G L K A | 242-250 | alpha-helix | A S A F F T L Y A | 226-234 | alpha-helix |
|  | 19 | M L T Q | 253-256 | beta-sheet | E M T C | 237-240 | beta-sheet |
| Capping | 20 | P F R F K | 263-267 | beta-sheet | P V R F K | 247-251 | beta-sheet |
| (262-393 AA) | 21 | G A W F L F | 274-279 | alpha-helix | G A W F L L | 258-263 | alpha-helix |
|  | 22 | K A K N A | 283-287 | beta-sheet | V A T N A | 267-271 | beta-sheet |
|  | 23 | I K | 293-294 | alpha-helix | **Y** M Q **alpha-helix elongated** | 276-278 | alpha-helix |
|  | 24 | A | 298 | alpha-helix | D | 285 | alpha-helix |
|  | 25 | Y G | 306-307 | alpha-helix | Y G | 290-291 | alpha-helix |
|  | 26 | T P L R N H Q V M L E I M D | 314-327 | alpha-helix | A N L R N R W M M L D V M E | 298-311 | alpha-helix |
|  | 27 | P I M Y M H | 331-335 | beta-sheet | P I Y M Q | 315-319 | beta-sheet |
|  | 28 | T E E A L A E L A | 336-344 | alpha-helix | T A E A I Q R I A **loop elongated** | 320-328 | alpha-helix |
|  | 29 | K K K L K H I Y E E A F E D F L | 348-363 | alpha-helix | →R K L K E L E A E A W E D F L **shortened** | 339-353 | alpha-helix |
|  | 30 | S Q A L L W A C Q | 368-375 | alpha-helix | S Q A I L W A A S | 358-366 | alpha-helix |
|  | 31 | S E A A P A | 385-390 | beta-sheet | S E I M A C | 375-380 | beta-sheet |
| **FAD Binding** | 32 | G F W | 403-405 | beta-sheet | G A W | 393-395 | beta-sheet |
| **site II** | 33 | K | 419 | alpha-helix | **D T K G elongated** | 406-409 | alpha-helix |
| (393-487 AA) | 34 | R M T | 427-429 | beta-sheet | Q M T | 417-419 | beta-sheet |
|  | 35 | G L F A I | 433-437 | beta-sheet | G L F A A | 423-427 | beta-sheet |
|  | 36 | S S G S F T E G R I A A K A A V R F I L E Q | 449-470 | alpha-helix | S S G S H A E G R I A G K A A I S Y I V D N | 439-459 | alpha-helix |
|  | 37 | D A V V E E L K K K | 478-487 | alpha-helix | Q G Q I D A L T A E | 468-477 | alpha-helix |
| Helical | 38 | P M E R F M Q | 491-497 | alpha-helix | P L D T Y E E | 481-487 | alpha-helix |
| (488-643 AA) | 39 | P W Q G L V R L Q K I M D E | 514-527 | alpha-helix | A K Q F M F R L Q K I M D E | 504-517 | alpha-helix |
|  | 40 | Y K T | 536-538 | beta-sheet | F K T | 526-528 | beta-sheet |
|  | 41 | E K M L L Q R A L E L L A F L K E D L | 540-557 | alpha-helix | D K L I E K G L E L M A F L K E D A | 530-547 | alpha-helix |
|  | 42 | L H E L M R A W E L V H R V W T A E A H V R H M L F | 565-590 | alpha-helix | L H D L M R C W E A Y H R M F Q G E A H M R S I L F | 555-580 | alpha-helix |
|  | 43 | Y R T | 601-603 | beta-sheet | F R S | 591-593 | beta-sheet |
|  | 44 | K C F V C S K Y D | 614-622 | beta-sheet | L A F C N C K Y D | 604-612 | beta-sheet |
|  | 45 | E W T F E K V P Y | 627-635 | beta-sheet | E W E M M K K P V | 617-625 | beta-sheet |
|  |  |  |  |  |  |  |  |

**Str. deviations:** 68-71 loop **630 AA total** **Protein problems: backbone**

150-156 loop **identical AA 342** 54,3% Ala47, Cys173, Asp252, Ala337, Lys338, His388*, Phe438* 169-173 loop **similar AA** 463** 73,5%

**1.0Ǻ RMSD Bb 610 96,8%**

330-337 loop ** incl. identical AA

406-409 alpha-helix elongated

# Secondary structure element succession AprA *Thermodesulfobacterium commune*

| **AprA domains** | **No** | ***Archaeoglobus fulgidus* sequence** | AA position | **Sec. str. element** | *Thermodesulfobacterium commune* sequence | AA position | **Sec. str. element** |
| --- | --- | --- | --- | --- | --- | --- | --- |
| FAD-Binding | 1 | P T E V V E T D I L I I G | 17-29 | beta-sheet | E I V E K E T D I L I V G | 17-29 | beta-sheet |
| **site I** | 2 | F S G C G G A A Y E A A Y W A K | 32-44 | alpha-helix | M A A C G A A V E A V Q W A K | 32-46 | alpha-helix |
| (2-261 AA) | 3 | K V T L V E | 51-56 | beta-sheet | R I L L C D | 51-56 | beta-sheet |
|  | 4 | S A I | 71-73 | beta-sheet | S A I **loop shortened** | 71-73 | beta-sheet |
|  | 5 | L E D Y V R Y V T L D M | 89-100 | alpha-helix | P D D Y V R M V R C D L | 82-93 | alpha-helix |
|  | 6 | E D L V A D Y A R H V D G T V H L F E K | 106-125 | alpha-helix | E D L V F D V G R H V D D T V H C F E E W | 99-119 | alpha-helix |
|  | 7 | P I W K T | 129-133 | beta-sheet | P I W K K D P**→ beta-sheet elongated** | 122-128 | beta-sheet |
|  |  |  |  |  | N G A (new) | 148-150 | **alpha-helix** |
|  | 8 | K Y V R E | 137-141 | beta-sheet | → Q P V R S **beta-sheet elongated** | 151-155 | beta-sheet |
|  | 9 | Q I M I H | 145-149 | beta-sheet | Q I M I N | 159-163 | beta-sheet |
|  | 10 | Y K P I I A E A A K M A V | 153-165 | alpha-helix | Y K V I V A E A A K N A L  **loop elongated** | 167-179 | alpha-helix |
|  | 11 | N I Y E R V F I F E L L K D | 169-182 | beta-sheet | E I I E R C F I V R P L L D | 186-199 | beta-sheet |
|  | 12 | A V A G A V G F S V | 188-197 | beta-sheet | R C A G A V G F S V | 205-214 | beta-sheet |
|  | 13 | K F Y V F K A | 201-207 | beta-sheet | K I Y I I K A | 218-224 | beta-sheet |
|  | 14 | A V I L A | 209-213 | beta-sheet | A T L L A | 226-230 | beta-sheet |
|  | 15 | G A T | 216-218 | beta-sheet | G A V | 233-235 | beta-sheet |
|  | 16 | A A | 229-230 | alpha-helix | G K | 246-247 | alpha-helix |
|  | 17 | D T G | 239-241 | beta- sheet | N P G | 256-258 | beta- sheet |
|  | 18 | S G Y Y M G L K A | 242-250 | alpha-helix | T G Y A M C A M S | 259-267 | alpha-helix |
|  | 19 | M L T Q | 253-256 | beta-sheet | K L V L | 270-273 | beta-sheet |
| Capping | 20 | P F R F K | 263-267 | beta-sheet | P A R F K | 280-284 | beta-sheet |
| (262-393 AA) | 21 | G A W F L F | 274-279 | alpha-helix | G A W F L L | 291-296 | alpha-helix |
|  | 22 | K A K N A | 283-287 | beta-sheet | R A T N A | 300-304 | beta-sheet |
|  | 23 | I K | 293-294 | alpha-helix | V A | 310-311 | alpha-helix |
|  | 24 | A | 298 | alpha-helix | K | 318 | alpha-helix |
|  | 25 | Y G | 306-307 | alpha-helix | Y G | 323-324 | alpha-helix |
|  | 26 | T P L R N H Q V M L E I M D | 314-327 | alpha-helix | T C L R N H A M L I E M E Q | 332-345 | alpha-helix |
|  | 27 | P I M Y M H | 331-335 | beta-sheet | P I Y M H | 349-353 | beta-sheet |
|  | 28 | T E E A L A E L A | 336-344 | alpha-helix | T E W A L Q E E A | 354-362 | alpha-helix |
|  | 29 | K K K L K H I Y E E A F E D F L | 348-363 | alpha-helix | →K E F K H L I A E A W E D F L **shortened** | 367-381 | alpha-helix |
|  | 30 | S Q A L L W A C Q | 368-375 | alpha-helix | T Q A G L W A C L | 386-394 | alpha-helix |
|  | 31 | S E A A P A | 385-390 | beta-sheet | S E I M P T | 403-408 | beta-sheet |
| **FAD Binding** | 32 | G F W | 403-405 | beta-sheet | G A W **loop elongated** | 421-423 | beta-sheet |
| **site II** | 33 | K | 419 | alpha-helix | **missing** |  | alpha-helix |
| (393-487 AA) | 34 | R M T | 427-429 | beta-sheet | R M T | 448-450 | beta-sheet |
|  | 35 | G L F A I | 433-437 | beta-sheet | G L F C A | 454-458 | beta-sheet |
|  | 36 | S S G S F T E G R I A A K A A V R F I L E Q | 449-470 | alpha-helix | S S G S H V E G R I A A K A M V Q Y C L D N | 470-491 | alpha-helix |
|  | 37 | D A V V E E L K K K | 478-487 | alpha-helix | **→** T A E E L K K E **shortened** | 501-508 | alpha-helix |
| Helical | 38 | P M E R F M Q | 491-497 | alpha-helix | P W Y R F E E | 512-518 | alpha-helix |
| (488-643 AA) | 39 | P W Q G L V R L Q K I M D E | 514-527 | alpha-helix | P R H I Q A R L M K L M D E | 535-548 | alpha-helix |
|  | 40 | Y K T | 536-538 | beta-sheet | Y K T | 557-559 | beta-sheet |
|  | 41 | E K M L L Q R A L E L L A F L K E D L | 540-557 | alpha-helix | K V M L E R G L D L L R M L K E D M | 561-578 | alpha-helix |
|  | 42 | L H E L M R A W E L V H R V W T A E A H V R H M L F | 565-590 | alpha-helix | L H E L M R A W E N R H R V W T A E A H L L H I L F | 586-611 | alpha-helix |
|  | 43 | Y R T | 601-603 | beta-sheet | Y R T | 621-623 | beta-sheet |
|  | 44 | K C F V C S K Y D | 614-622 | beta-sheet | R C F T L S S W D | 635-643 | beta-sheet |
|  | 45 | E W T F E K V P Y | 627-635 | beta-sheet | E W T L E T Y P Y | 648-656 | beta-sheet |
|  |  |  |  |  |  |  |  |

**Str. deviations:** 8-15 loop **664 AA total** **Protein problems: backbone**

**identical AA 369** 55,6% Pro2, Pro16, Ala58, Pro122, Pro128, Gln151, Ala185, Pro196, Asp244,

77-79 loop **similar AA** 463** 70,0% Pro257, Asp285, Pro322, Lys363, Asp365, Lys366, His416, Asp430, Pro439, 128-150 alpha **1.0Ǻ RMSD Bb 610 91,9%** Asn447, Phe469, Lys499, Pro663 180-184 loop ** incl. identical AA

363-365 loop 429-431 loop 442-446 loop

# Secondary structure element succession AprA *Desulfovibrio vulgaris*

| **AprA domains** | **No** | ***Archaeoglobus fulgidus* sequence** | AA position | **Sec. str. element** | *Desulfovibrio vulgaris* sequence | AA position | **Sec. str. element** |
| --- | --- | --- | --- | --- | --- | --- | --- |
| FAD-Binding | 1 | P T E V V E T D I L I I G | 17-29 | beta-sheet | T V K E H D V D L L I V G | 19-31 | beta-sheet |
| **site I** | 2 | F S G C G G A A Y E A A Y W A K | 32-44 | alpha-helix | M G A C G T A F E A V R W →**shortened.** | 34-46 | alpha-helix |
| (2-261 AA) | 3 | K V T L V E | 51-56 | beta-sheet | K I L L I D | 55-60 | beta-sheet |
|  | 4 | S A I | 71-73 | beta-sheet | S A I **loop shortened** | 75-77 | beta-sheet |
|  | 5 | L E D Y V R Y V T L D M | 89-100 | alpha-helix | A D D Y V R M V R T D L | 86-97 | alpha-helix |
|  | 6 | E D L V A D Y A R H V D G T V H L F E K | 106-125 | alpha-helix | E D L I F D L G R H V D D S V H L F E E W | 103-123 | alpha-helix |
|  | 7 | P I W K T | 129-133 | beta-sheet | P C W I K **→ elongated** | 126-130 | beta-sheet |
|  |  |  |  |  | D E H G H N L D G A (new) | 131-140 | **beta-sheet** |
|  |  |  |  |  | K A A G K S L R N G D (new) | 143-153 | **beta-sheet** |
|  | 8 | K Y V R E | 137-141 | beta-sheet | **→** D P V R S **elongated** | 154-158 | beta-sheet |
|  | 9 | Q I M I H | 145-149 | beta-sheet | Q I M I N | 162-166 | beta-sheet |
|  | 10 | Y K P I I A E A A K M A V | 153-165 | alpha-helix | Y K C I V A E A A K N A L | 170-182 | alpha-helix |
|  | 11 | N I Y E R V F I F E L L K D | 169-182 | beta-sheet | R I M E R I F I V K L L L D | 186-199 | beta-sheet |
|  | 12 | A V A G A V G F S V | 188-197 | beta-sheet | R V A G AV G F N L | 205-214 | beta-sheet |
|  | 13 | K F Y V F K A | 201-207 | beta-sheet | E V H I F R S | 218-224 | beta-sheet |
|  | 14 | A V I L A | 209-213 | beta-sheet | A M L V A | 226-230 | beta-sheet |
|  | 15 | G A T | 216-218 | beta-sheet | G A V | 223-225 | beta-sheet |
|  | 16 | A A | 229-230 | alpha-helix | G M | 246-247 | alpha-helix |
|  | 17 | D T G | 239-241 | beta- sheet | N A G | 256-258 | beta- sheet |
|  | 18 | S G Y Y M G L K A | 242-250 | alpha-helix | S T Y T M C A Q V | 259-267 | alpha-helix |
|  | 19 | M L T Q | 253-256 | beta-sheet | E M T M | 270-273 | beta-sheet |
| Capping | 20 | P F R F K | 263-267 | beta-sheet | P A R F K | 280-284 | beta-sheet |
| (262-393 AA) | 21 | G A W F L F | 274-279 | alpha-helix | G A W F L L | 291-296 | alpha-helix |
|  | 22 | K A K N A | 283-287 | beta-sheet | K A T N Y | 300-304 | beta-sheet |
|  | 23 | I K | 293-294 | alpha-helix | C A | 310-311 | alpha-helix |
|  | 24 | A | 298 | alpha-helix | A | 315 | alpha-helix |
|  | 25 | Y G | 306-307 | alpha-helix | R G **loop elongated** | 323-324 | alpha-helix |
|  | 26 | T P L R N H Q V M L E I M D | 314-327 | alpha-helix | T C L R N H M M L R E M R E | 333-346 | alpha-helix |
|  | 27 | P I M Y M H | 331-335 | beta-sheet | P I Y M D | 350-354 | beta-sheet |
|  | 28 | T E E A L A E L A | 336-344 | alpha-helix | T K T A L Q S T F | 355-362 | alpha-helix |
|  | 29 | K K K L K H I Y E E A F E D F L | 348-363 | alpha-helix | →E Q Q K H L E S E A W E D F L **shortened** | 369-383 | alpha-helix |
|  | 30 | S Q A L L W A C Q | 368-375 | alpha-helix | G Q A N L W A S M | 388-396 | alpha-helix |
|  | 31 | S E A A P A | 385-390 | beta-sheet | S E I M P T | 405-410 | beta-sheet |
| **FAD Binding** | 32 | G F W | 403-405 | beta-sheet | G I W **loop elongated** | 423-425 | beta-sheet |
| **site II** | 33 | K | 419 | alpha-helix | S N **alpha-helix elongated** | 443-444 | alpha-helix |
| (393-487 AA) | 34 | R M T | 427-429 | beta-sheet | R M T | 450-452 | beta-sheet |
|  | 35 | G L F A I | 433-437 | beta-sheet | G L W T C | 456-460 | beta-sheet |
|  | 36 | S S G S F T E G R I A A K A A V R F I L E Q | 449-470 | alpha-helix | S S G S H A E G R I C G K Q M V R W C L D H | 472-493 | alpha-helix |
|  | 37 | D A V V E E L K K K | 478-487 | alpha-helix | → S A D E L V K L  **shortened** | 503-510 | alpha-helix |
| Helical | 38 | P M E R F M Q | 491-497 | alpha-helix | P Y Y N Y M E | 514-520 | alpha-helix |
| (488-643 AA) | 39 | P W Q G L V R L Q K I M D E | 514-527 | alpha-helix | P K N F M M R L V K C T D E | 537-550 | alpha-helix |
|  | 40 | Y K T | 536-538 | beta-sheet | Y T T | 559-561 | beta-sheet |
|  | 41 | E K M L L Q R A L E L L A F L K E D L | 540-557 | alpha-helix | A A A L D T G F S L L G M L E E D S | 563-580 | alpha-helix |
|  | 42 | L H E L M R A W E L V H R V W T A E A H V R H M L F | 565-590 | alpha-helix | L H E L L R C W E N Y H R L W T V R L H M Q H I R F | 588-613 | alpha-helix |
|  | 43 | Y R T | 601-603 | beta-sheet | Y R A | 624-626 | beta-sheet |
|  | 44 | K C F V C S K Y D | 614-622 | beta-sheet | K C F V N S K Y D | 637-645 | beta-sheet |
|  | 45 | E W T F E K V P Y | 627-635 | beta-sheet | E T K I F K K A Y | 650-658 | beta-sheet |
|  |  |  |  |  |  |  |  |

**Str. deviations:** 12-14 loop **664 AA** **Protein problems: backbone**

81-83 loop **identical AA 329** 49,5 Pro2, Glu17, Pro18, Glu53, Leu54, Ala62, Pro126, Gln141, Asp285, Trp433,

**similar AA** 432** 65,1 Ile331, Pro368, His418, Lys439, Ile447, Arg450, Ala461, Phe471

132-153 beta-loop-beta **1.0Ǻ RMSD Bb 595 89,6%**

327-330 loop ** incl. identical AA

- 1. loop

430-433 loop verlängert d. +1 As Insertion

436-447 alpha-helix

- 1. loop

495-501 loop

# Secondary structure element succession AprA *Desulfovibrio desulfuricans*

| **AprA domains** | **No** | ***Archaeoglobus fulgidus* sequence** | AA position | **Sec. str. element** | *Desulfovibrio desulfuricans* sequence | AA position | **Sec. str. element** |
| --- | --- | --- | --- | --- | --- | --- | --- |
| FAD-Binding | 1 | P T E V V E T D I L I I G | 17-29 | beta-sheet | E V K E H A V D L L I V G | 19-31 | beta-sheet |
| **site I** | 2 | F S G C G G A A Y E A A Y W A K | 32-44 | alpha-helix | M G S C G T A F E A V R W G D | 34-48 | alpha-helix |
| (2-261 AA) | 3 | K V T L V E | 51-56 | beta-sheet | K I M L V D | 53-58 | beta-sheet |
|  | 4 | S A I | 71-73 | beta-sheet | S A I **loop shortened** | 73-75 | beta-sheet |
|  | 5 | L E D Y V R Y V T L D M | 89-100 | alpha-helix | A D D Y V R M V R T D L | 84-95 | alpha-helix |
|  | 6 | E D L V A D Y A R H V D G T V H L F E K | 106-125 | alpha-helix | E D L I F D V G R H V D D S V H L F E D W | 101-121 | alpha-helix |
|  | 7 | P I W K T | 129-133 | beta-sheet | P C W I K | 126-130 | beta-sheet |
|  |  |  |  |  | D G P H L E G A A (new) | 131-139 | **beta-sheet** |
|  |  |  |  |  | V A G K S L R K G D (new) | 142-150 | **beta-sheet** |
|  | 8 | K Y V R E | 137-141 | beta-sheet | A P V R S | 154-156 | beta-sheet |
|  | 9 | Q I M I H | 145-149 | beta-sheet | Q I M I N | 160-164 | beta-sheet |
|  | 10 | Y K P I I A E A A K M A V | 153-165 | alpha-helix | Y K C I V A E A A K N A L | 168-180 | alpha-helix |
|  | 11 | N I Y E R V F I F E L L K D | 169-182 | beta-sheet | R I M E R I F I V K L L L D | 184-197 | beta-sheet |
|  | 12 | A V A G A V G F S V | 188-197 | beta-sheet | R I A G A V G F N L | 203-212 | beta-sheet |
|  | 13 | K F Y V F K A | 201-207 | beta-sheet | E V H I F K A | 216-222 | beta-sheet |
|  | 14 | A V I L A | 209-213 | beta-sheet | T I M V A | 224-228 | beta-sheet |
|  | 15 | G A T | 216-218 | beta-sheet | G A V | 231-233 | beta-sheet |
|  | 16 | A A | 229-230 | alpha-helix | G M | 244-245 | alpha-helix |
|  | 17 | D T G | 239-241 | beta- sheet | N A G | 254-256 | beta- sheet |
|  | 18 | S G Y Y M G L K A | 242-250 | alpha-helix | S T Y T M C A Q V | 257-265 | alpha-helix |
|  | 19 | M L T Q | 253-256 | beta-sheet | E M T M | 268-271 | beta-sheet |
| Capping | 20 | P F R F K | 263-267 | beta-sheet | P A R F K | 278-282 | beta-sheet |
| (262-393 AA) | 21 | G A W F L F | 274-279 | alpha-helix | G A W F L L | 289-294 | alpha-helix |
|  | 22 | K A K N A | 283-287 | beta-sheet | K A T N S | 298-302 | beta-sheet |
|  | 23 | I K | 293-294 | alpha-helix | C A | 308-309 | alpha-helix |
|  | 24 | A | 298 | alpha-helix | A | 313 | alpha-helix |
|  | 25 | Y G | 306-307 | alpha-helix | R G **loop elongated** | 321-322 | alpha-helix |
|  | 26 | T P L R N H Q V M L E I M D | 314-327 | alpha-helix | T C L R N H M M L R E M R E | 331-344 | alpha-helix |
|  | 27 | P I M Y M H | 331-335 | beta-sheet | P I Y M D | 347-352 | beta-sheet |
|  | 28 | T E E A L A E L A | 336-344 | alpha-helix | T K S A L Q N T F | 353-361 | alpha-helix |
|  | 29 | K K K L K H I Y E E A F E D F L | 348-363 | alpha-helix | →E Q Q K D L E S E A W E D F L **shortened** | 367-381 | alpha-helix |
|  | 30 | S Q A L L W A C Q | 368-375 | alpha-helix | G Q A N L W A C T | 386-394 | alpha-helix |
|  | 31 | S E A A P A | 385-390 | beta-sheet | S E I M P T | 403-408 | beta-sheet |
| **FAD Binding** | 32 | G F W | 403-405 | beta-sheet | G I W **loop elongated** | 421-423 | beta-sheet |
| **site II** | 33 | K | 419 | alpha-helix | **missing** |  |  |
| (393-487 AA) | 34 | R M T | 427-429 | beta-sheet | R M T | 448-450 | beta-sheet |
|  | 35 | G L F A I | 433-437 | beta-sheet | G L F T C | 454-458 | beta-sheet |
|  | 36 | S S G S F T E G R I A A K A A V R F I L E Q | 449-470 | alpha-helix | S S G S H A E G R M A G K Q M V R W C L D H | 470-491 | alpha-helix |
|  | 37 | D A V V E E L K K K | 478-487 | alpha-helix | **→** T A E E L K K A **shortened** | 501-508 | alpha-helix |
| Helical | 38 | P M E R F M Q | 491-497 | alpha-helix | P F Y N F E E | 512-518 | alpha-helix |
| (488-643 AA) | 39 | P W Q G L V R L Q K I M D E | 514-527 | alpha-helix | P K N F M M R L V K C T D E | 535-548 | alpha-helix |
|  | 40 | Y K T | 536-538 | beta-sheet | Y T T | 557-559 | beta-sheet |
|  | 41 | E K M L L Q R A L E L L A F L K E D L | 540-557 | alpha-helix | K A L L D T G F N L L A M M E E D S | 561-578 | alpha-helix |
|  | 42 | L H E L M R A W E L V H R V W T A E A H V R H M L F | 565-590 | alpha-helix | L H E L L R C W E N Y H R L W T V R L H M Q H I S F | 586-611 | alpha-helix |
|  | 43 | Y R T | 601-603 | beta-sheet | Y R A | 622-624 | beta-sheet |
|  | 44 | K C F V C S K Y D | 614-622 | beta-sheet | K C F V N S K Y N | 634-643 | beta-sheet |
|  | 45 | E W T F E K V P Y | 627-635 | beta-sheet | E T K I F K K P Y | 648-656 | beta-sheet |
|  |  |  |  |  |  |  |  |

**Str. deviations:** 12-15 loop **662 AA total** **Protein problems: backbone**

79-81 loop **identical AA 332** 50,2 Pro2, Ala16, Glu17, Ala60, Pro124, Pro133, Val142, Asp283,

**similar AA** 444** 67,1 Asn365, Glu366, His416, Val432, Pro433, Ala459, Phe469, 131-151 beta-loop-beta **1.0Ǻ RMSD Bb 601 90,8%** Lys437, Val445 325-328 loop ** incl. identical AA

362-365 loop

430-432 loop

- 1. alpha-helix missing

493-499 loop

# Secondary structure element succession AprA *Desulfobulbus* sp. Str. MLMS-1

| **AprA domains** | **No** | ***Archaeoglobus fulgidus* sequence** | AA position | **Sec. str. element** | *Desulfobulbus* sp. sequence | AA position | **Sec. str. element** |
| --- | --- | --- | --- | --- | --- | --- | --- |
| FAD-Binding | 1 | P T E V V E T D I L I I G | 17-29 | beta-sheet | E V V E H D V D V L I I G | 18-30 | beta-sheet |
| **site I** | 2 | F S G C G G A A Y E A A Y W A K | 32-44 | alpha-helix | M A A C G T A F E I K K W→ **shortened** | 33-45 | alpha-helix |
| (2-261 AA) | 3 | K V T L V E | 51-56 | beta-sheet | K I K L V D | 51-56 | beta-sheet |
|  | 4 | S A I | 71-73 | beta-sheet | S A I **loop shortened** | 71-73 | beta-sheet |
|  | 5 | L E D Y V R Y V T L D M | 89-100 | alpha-helix | I E N Y V K M V R N D L | 82-93 | alpha-helix |
|  | 6 | E D L V A D Y A R H V D G T V H L F E K | 106-125 | alpha-helix | E D L I Y D L G R H V D E S V K L F E E W | 99-119 | alpha-helix |
|  | 7 | P I W K T | 129-133 | beta-sheet | P I W K K | 122-126 | beta-sheet |
|  |  |  |  |  | **G D N (new)** | 130-132 | **beta-sheet** |
|  |  |  |  |  | **R E G (new)** | 143-145 | **beta-sheet** |
|  | 8 | K Y V R E | 137-141 | beta-sheet | T P V R T | 147-151 | beta-sheet |
|  | 9 | Q I M I H | 145-149 | beta-sheet | Q I M I N | 155-159 | beta-sheet |
|  | 10 | Y K P I I A E A A K M A V | 153-165 | alpha-helix | Y K C I V A E P A K T A L | 163-175 | alpha-helix |
|  | 11 | N I Y E R V F I F E L L K D | 169-182 | beta-sheet | N I L E R V F I V K L I L D | 179-192 | beta-sheet |
|  | 12 | A V A G A V G F S V | 188-197 | beta-sheet | Q I A G A V G F S T | 198-207 | beta-sheet |
|  | 13 | K F Y V F K A | 201-207 | beta-sheet | K V H V F R C | 211-217 | beta-sheet |
|  | 14 | A V I L A | 209-213 | beta-sheet | T A L C A | 219-223 | beta-sheet |
|  | 15 | G A T | 216-218 | beta-sheet | G A V | 226-228 | beta-sheet |
|  | 16 | A A | 229-230 | alpha-helix | G K | 239-240 | alpha-helix |
|  | 17 | D T G | 239-241 | beta- sheet | N A G | 249-251 | beta- sheet |
|  | 18 | S G Y Y M G L K A | 242-250 | alpha-helix | S T Y T M C A Q V | 252-260 | alpha-helix |
|  | 19 | M L T Q | 253-256 | beta-sheet | T L T M | 263-266 | beta-sheet |
| Capping | 20 | P F R F K | 263-267 | beta-sheet | P A R F K | 273-277 | beta-sheet |
| (262-393 AA) | 21 | G A W F L F | 274-279 | alpha-helix | G A W F L L | 284-289 | alpha-helix |
|  | 22 | K A K N A | 283-287 | beta-sheet | K V Q N G | 293-297 | beta-sheet |
|  | 23 | I K | 293-294 | alpha-helix | **missing** |  |  |
|  | 24 | A | 298 | alpha-helix | missing |  |  |
|  | 25 | Y G | 306-307 | alpha-helix | **missing** |  |  |
|  | 26 | T P L R N H Q V M L E I M D | 314-327 | alpha-helix | T C L R N H L L L N E L K A | 326-339 | alpha-helix |
|  | 27 | P I M Y M H | 331-335 | beta-sheet | P I Y M A | 343-347 | beta-sheet |
|  | 28 | T E E A L A E L A | 336-344 | alpha-helix | T D V A L N A F L→ **shortened** | 348-355 | alpha-helix |
|  |  |  |  |  | R R E A G M (new) | 359-364 | **alpha-helix** |
|  | 29 | K K K L K H I Y E E A F E D F L | 348-363 | alpha-helix | →K F W K H L E S E A W E D F L **shortened** | 371-385 | alpha-helix |
|  | 30 | S Q A L L W A C Q | 368-375 | alpha-helix | G Q A G L W A G M | 390-398 | alpha-helix |
|  | 31 | S E A A P A | 385-390 | beta-sheet | S E I M P T | 407-412 | beta-sheet |
| **FAD Binding** | 32 | G F W | 403-405 | beta-sheet | G I W **loop elongated** | 425-427 | beta-sheet |
| **site II** | 33 | K | 419 | alpha-helix | **missing** |  |  |
| (393-487 AA) | 34 | R M T | 427-429 | beta-sheet | R M T | 453-455 | beta-sheet |
|  | 35 | G L F A I | 433-437 | beta-sheet | G L F T A | 459-463 | beta-sheet |
|  | 36 | S S G S F T E G R I A A K A A V R F I L E Q | 449-470 | alpha-helix | S S G S H A E G R I V A K M M V R F C R D N | 475-496 | alpha-helix |
|  | 37 | D A V V E E L K K K | 478-487 | alpha-helix | →S A Q E Y A D E **shortened** | 506-513 | alpha-helix |
| Helical | 38 | P M E R F M Q | 491-497 | alpha-helix | P V K R Y L E | 517-523 | alpha-helix |
| (488-643 AA) | 39 | P W Q G L V R L Q K I M D E | 514-527 | alpha-helix | P A G L M M R L M K A T D E | 540-553 | alpha-helix |
|  | 40 | Y K T | 536-538 | beta-sheet | Y M T | 562-564 | beta-sheet |
|  | 41 | E K M L L Q R A L E L L A F L K E D L | 540-557 | alpha-helix | G K L L N I C L D L L K M M R E D A | 566-583 | alpha-helix |
|  | 42 | L H E L M R A W E L V H R V W T A E A H V R H M L F | 565-590 | alpha-helix | L H E L M R A W E N Y H R I W C V E T H I R H I E F | 591-616 | alpha-helix |
|  | 43 | Y R T | 601-603 | beta-sheet | Y R S | 627-629 | beta-sheet |
|  | 44 | K C F V C S K Y D | 614-622 | beta-sheet | K A F V N S T F D | 640-648 | beta-sheet |
|  | 45 | E W T F E K V P Y | 627-635 | beta-sheet | E W K C E K V E C | 653-661 | beta-sheet |
|  |  |  |  |  |  |  |  |

**Str. deviations:** 5-14 loop **669AA total** **Protein problems: backbone**

46-48 loop **identical AA 334** 49,9% Ala15, Pro81, Asp127, Pro134, Ala139, Thr147, Asp278,

77-79 loop **similar AA** 446** 66,7% Gln309, Pro317, Lys370, His420, Asp434, Pro442, Asn452, **1.0Ǻ RMSD Bb 600 89,7%** Pro504, Lys505, Phe474

129-146 beta-loop-beta ** incl. identical AA

305-308 loop

- 1. loop

361-371 helix-loop

359-369 loop-helix-loop

- 1. loop

447-451 alpha-helix missing

498-504 loop

# Secondary structure element succession AprA *Desulfotalea psychrophila*

| **AprA domains** | **No** | ***Archaeoglobus fulgidus* sequence** | AA position | **Sec. str. element** | *Desulfotalea psychrophila* sequence | AA position | **Sec. str. element** |
| --- | --- | --- | --- | --- | --- | --- | --- |
| FAD-Binding | 1 | P T E V V E T D I L I I G | 17-29 | beta-sheet | E I V E H D I D V L I V G | 20-32 | beta-sheet |
| **site I** | 2 | F S G C G G A A Y E A A Y W A K | 32-44 | alpha-helix | M A A C G T A F E I K K W→ **shortened** | 35-47 | alpha-helix |
| (2-261 AA) | 3 | K V T L V E | 51-56 | beta-sheet | K I M L V D | 55-59 | beta-sheet |
|  | 4 | S A I | 71-73 | beta-sheet | S A I **loop shortened** | 73-75 | beta-sheet |
|  | 5 | L E D Y V R Y V T L D M | 89-100 | alpha-helix | I E D Y V K M V R N D L | 84-95 | alpha-helix |
|  | 6 | E D L V A D Y A R H V D G T V H L F E K | 106-125 | alpha-helix | E D L I V D C G R H V D E S V K L F E E W | 101-121 | alpha-helix |
|  | 7 | P I W K T | 129-133 | beta-sheet | P V W K K **→ elongated** | 124-128 | beta-sheet |
|  |  |  |  |  | **D A A G D N L D (new)** | 129-136 | **beta-sheet** |
|  |  |  |  |  | **P A A S L R E G G (new)** | 140-148 | **beta-sheet** |
|  | 8 | K Y V R E | 137-141 | beta-sheet | → T P V R T **elongated** | 149-153 | beta-sheet |
|  | 9 | Q I M I H | 145-149 | beta-sheet | Q I M I N | 157-161 | beta-sheet |
|  | 10 | Y K P I I A E A A K M A V | 153-165 | alpha-helix | Y K P I V A E P A A T A L | 165-177 | alpha-helix |
|  | 11 | N I Y E R V F I F E L L K D | 169-182 | beta-sheet | N I E R V F I V K L L L D | 181-194 | beta-sheet |
|  | 12 | A V A G A V G F S V | 188-197 | beta-sheet | Q I A G A A G F S T | 200-209 | beta-sheet |
|  | 13 | K F Y V F K A | 201-207 | beta-sheet | T V H I F R C | 213-219 | beta-sheet |
|  | 14 | A V I L A | 209-213 | beta-sheet | A A M V A | 221-225 | beta-sheet |
|  | 15 | G A T | 216-218 | beta-sheet | G A V | 228-230 | beta-sheet |
|  | 16 | A A | 229-230 | alpha-helix | G K | 241-242 | alpha-helix |
|  | 17 | D T G | 239-241 | beta- sheet | N A G | 251-253 | beta- sheet |
|  | 18 | S G Y Y M G L K A | 242-250 | alpha-helix | S T Y T M C A Q V | 254-262 | alpha-helix |
|  | 19 | M L T Q | 253-256 | beta-sheet | T L T M | 265-268 | beta-sheet |
| Capping | 20 | P F R F K | 263-267 | beta-sheet | P A R F K | 275-279 | beta-sheet |
| (262-393 AA) | 21 | G A W F L F | 274-279 | alpha-helix | G A W F L L | 286-291 | alpha-helix |
|  | 22 | K A K N A | 283-287 | beta-sheet | K V A N G | 295-299 | beta-sheet |
|  | 23 | I K | 293-294 | alpha-helix | **missing** |  |  |
|  | 24 | A | 298 | alpha-helix | K E elongated | 311-312 | alpha-helix |
|  | 25 | Y G | 306-307 | alpha-helix | Y G **loop elongated** | 320-321 | alpha-helix |
|  | 26 | T P L R N H Q V M L E I M D | 314-327 | alpha-helix | T C L R N H L M I K E L K E | 328-341 | alpha-helix |
|  | 27 | P I M Y M H | 331-335 | beta-sheet | P I Y M A | 345-349 | beta-sheet |
|  | 28 | T E E A L A E L A | 336-344 | alpha-helix | T D V A L N A F L→ **shortened** | 350-358 | alpha-helix |
|  |  |  |  |  | R K E A G L (new) | 361-366 | **alpha-helix** |
|  | 29 | K K K L K H I Y E E A F E D F L | 348-363 | alpha-helix | →K F W K H L E S E A W E D F L **shortened** | 373-387 | alpha-helix |
|  | 30 | S Q A L L W A C Q | 368-375 | alpha-helix | G Q A G L W A G N | 392-400 | alpha-helix |
|  | 31 | S E A A P A | 385-390 | beta-sheet | S E I M P T | 409-414 | beta-sheet |
| **FAD Binding** | 32 | G F W | 403-405 | beta-sheet | G I W **loop elongated** | 427-429 | beta-sheet |
| **site II** | 33 | K | 419 | alpha-helix | E **loop elongated** | 444 | alpha-helix |
| (393-487 AA) | 34 | R M T | 427-429 | beta-sheet | R M T | 456-458 | beta-sheet |
|  | 35 | G L F A I | 433-437 | beta-sheet | G L F T A | 462-466 | beta-sheet |
|  | 36 | S S G S F T E G R I A A K A A V R F I L E Q | 449-470 | alpha-helix | S S G S H A E G R I A A K Q M V K F C R→**shortened** | 478-497 | alpha-helix |
|  | 37 | D A V V E E L K K K | 478-487 | alpha-helix | →P Q E I A D E **shortened** | 509-515 | alpha-helix |
| Helical | 38 | P M E R F M Q | 491-497 | alpha-helix | P V R L Y N E | 519-525 | alpha-helix |
| (488-643 AA) | 39 | P W Q G L V R L Q K I M D E | 514-527 | alpha-helix | P A G L M M R L M K A T D E | 542-555 | alpha-helix |
|  | 40 | Y K T | 536-538 | beta-sheet | Y M T | 564-566 | beta-sheet |
|  | 41 | E K M L L Q R A L E L L A F L K E D L | 540-557 | alpha-helix | G K L L N I C L D L L L L L R E D A | 568-585 | alpha-helix |
|  | 42 | L H E L M R A W E L V H R V W T A E A H V R H M L F | 565-590 | alpha-helix | L H E L L R C W E N Y H R I W C V E T H I R H I E F | 593-618 | alpha-helix |
|  | 43 | Y R T | 601-603 | beta-sheet | Y R S | 629-631 | beta-sheet |
|  | 44 | K C F V C S K Y D | 614-622 | beta-sheet | K C F V N S T F N | 642-650 | beta-sheet |
|  | 45 | E W T F E K V P Y | 627-635 | beta-sheet | E W L C E K V E C | 655-663 | beta-sheet |
|  |  |  |  |  |  |  |  |

**Str. deviations:** 7-16 loop **670AA total** **Protein problems: backbone**

48-50 loop **identical AA 332** 49,6% Val16, Val17, Ala60, Asp136, Asp280,

79-81 loop **similar AA** 446** 66,6% Ala310, Pro319, Thr326, Lys372, His422, Phe477, Thr508 **1.0Ǻ RMSD Bb 598 89,3%**

130-148 beta-loop-beta ** incl. identical AA

306-310 loop

- 1. loop

361-371 helix-loop

435-437 loop

448-454 loop

498-507 loop

# Secondary structure element succession AprA *Olavius algarvensis* Delta 1 symbiont

| **AprA domains** | **No** | ***Archaeoglobus fulgidus* sequence** | AA position | **Sec. str. element** | *O. algarvensis* Delta 1 symbiont sequence | AA position | **Sec. str. element** |
| --- | --- | --- | --- | --- | --- | --- | --- |
| FAD-Binding | 1 | P T E V V E T D I L I I G | 17-29 | beta-sheet | E V E E R E V D I L I V G | 18-30 | beta-sheet |
| **site I** | 2 | F S G C G G A A Y E A A Y W A K | 32-44 | alpha-helix | M A A C G A A F E V K K W **→ shortened** | 33-45 | alpha-helix |
| (2-261 AA) | 3 | K V T L V E | 51-56 | beta-sheet | S V L L V D | 51-55 | beta-sheet |
|  | 4 | S A I | 71-73 | beta-sheet | S A I **loop shortened** | 71-73 | beta-sheet |
|  | 5 | L E D Y V R Y V T L D M | 89-100 | alpha-helix | P E D Y V R M V R N D L | 82-93 | alpha-helix |
|  | 6 | E D L V A D Y A R H V D G T V H L F E K | 106-125 | alpha-helix | E D L I F D L G C H V D D S V H L F E E W | 99-119 | alpha-helix |
|  | 7 | P I W K T | 129-133 | beta-sheet | P V W K K | 122-126 | beta-sheet |
|  |  |  |  |  | G Q K M G T L K S (nee) | 138-146 | **alpha-helix** |
|  | 8 | K Y V R E | 137-141 | beta-sheet | S P V R T | 149-153 | beta-sheet |
|  | 9 | Q I M I H | 145-149 | beta-sheet | Q I M I N | 157-161 | beta-sheet |
|  | 10 | Y K P I I A E A A K M A V | 153-165 | alpha-helix | Y K R I V A E A G K L A L | 165-177 | alpha-helix |
|  | 11 | N I Y E R V F I F E L L K D | 169-182 | beta-sheet | N I L E R V F I V E L L L D | 181-194 | beta-sheet |
|  | 12 | A V A G A V G F S V | 188-197 | beta-sheet | Q I A G A V G F S V | 200-209 | beta-sheet |
|  | 13 | K F Y V F K A | 201-207 | beta-sheet | K V I I F K C | 213-219 | beta-sheet |
|  | 14 | A V I L A | 209-213 | beta-sheet | T M M V A | 221-225 | beta-sheet |
|  | 15 | G A T | 216-218 | beta-sheet | G A V | 228-230 | beta-sheet |
|  | 16 | A A | 229-230 | alpha-helix | G K | 241-242 | alpha-helix |
|  | 17 | D T G | 239-241 | beta- sheet | N S G | 251-253 | beta- sheet |
|  | 18 | S G Y Y M G L K A | 242-250 | alpha-helix | S T Y T L C M K V | 254-262 | alpha-helix |
|  | 19 | M L T Q | 253-256 | beta-sheet | E L S M | 265-268 | beta-sheet |
| Capping | 20 | P F R F K | 263-267 | beta-sheet | P A R F K | 275-279 | beta-sheet |
| (262-393 AA) | 21 | G A W F L F | 274-279 | alpha-helix | G A W F L L | 286-291 | alpha-helix |
|  | 22 | K A K N A | 283-287 | beta-sheet | K T L N G | 295-299 | beta-sheet |
|  | 23 | I K | 293-294 | alpha-helix | **missing** |  |  |
|  | 24 | A | 298 | alpha-helix | A | 312 | alpha-helix |
|  | 25 | Y G | 306-307 | alpha-helix | Y G **loop elongated** | 320-321 | alpha-helix |
|  | 26 | T P L R N H Q V M L E I M D | 314-327 | alpha-helix | T C L R N H L M L F E M K E | 328-341 | alpha-helix |
|  | 27 | P I M Y M H | 331-335 | beta-sheet | P I I M D | 345-349 | beta-sheet |
|  | 28 | T E E A L A E L A | 336-344 | alpha-helix | T V S A L A A L G | 350-358 | alpha-helix |
|  | 29 | K K K L K H I Y E E A F E D F L | 348-363 | alpha-helix | →K E L K H L E S E A W E D F L **shortened** | 364-378 | alpha-helix |
|  | 30 | S Q A L L W A C Q | 368-375 | alpha-helix | G Q A N L W C A T | 383-391 | alpha-helix |
|  | 31 | S E A A P A | 385-390 | beta-sheet | S E V M P T | 400-405 | beta-sheet |
| **FAD Binding** | 32 | G F W | 403-405 | beta-sheet | G L W **loop elongated** | 418-420 | beta-sheet |
| **site II** | 33 | K | 419 | alpha-helix | **missing** |  |  |
| (393-487 AA) | 34 | R M T | 427-429 | beta-sheet | R M T | 445-447 | beta-sheet |
|  | 35 | G L F A I | 433-437 | beta-sheet | G L F T A | 451-455 | beta-sheet |
|  | 36 | S S G S F T E G R I A A K A A V R F I L E Q | 449-470 | alpha-helix | S S G S H A E G R M A V K Q M V R Y A K D H | 467-488 | alpha-helix |
|  | 37 | D A V V E E L K K K | 478-487 | alpha-helix | **→** Q S N E E L V D M **shortened** | 497-505 | alpha-helix |
| Helical | 38 | P M E R F M Q | 491-497 | alpha-helix | P V R T Y L D | 509-515 | alpha-helix |
| (488-643 AA) | 39 | P W Q G L V R L Q K I M D E | 514-527 | alpha-helix | P N G M M Y R M M K A G H E | 532-545 | alpha-helix |
|  | 40 | Y K T | 536-538 | beta-sheet | Y Q T | 554-556 | beta-sheet |
|  | 41 | E K M L L Q R A L E L L A F L K E D L | 540-557 | alpha-helix | S K N L E I A M D L L E T M R E D N | 558-575 | alpha-helix |
|  | 42 | L H E L M R A W E L V H R V W T A E A H V R H M L F | 565-590 | alpha-helix | L H E L M R A W E I Q H R I W T L E S H L R H I Q Y | 583-608 | alpha-helix |
|  | 43 | Y R T | 601-603 | beta-sheet | Y Q A | 619-621 | beta-sheet |
|  | 44 | K C F V C S K Y D | 614-622 | beta-sheet | F C F V N S K Y D | 631-640 | beta-sheet |
|  | 45 | E W T F E K V P Y | 627-635 | beta-sheet | K W D I F K K D Y | 645-653 | beta-sheet |
|  |  |  |  |  |  |  |  |

**Str. deviations:** 5-14 loop **659 AA total** **Protein problems: backbone**

47-50 loop **identical AA 339** 51,4% Val14, Arg15, Gln50, Ala58, Asn132, Asp280, Ala310, Pro319, Pro327,

77-79 loop **similar AA** 451** 68,4% Glu359, Lys363, His413, Asp427, Ile435, Tyr443, Phe466 128-148 alpha **1.0Ǻ RMS AA 589 89,4%**  305-310 loop ** incl. identical AA

322-326 loop

359-362 loop

426-428 loop

431-443 alpha-helix missing

490-496 loop

# Secondary structure element succession AprA *Thermodesulfovibrio yellowstonii*

| **AprA domains** | **No** | ***Archaeoglobus fulgidus* sequence** | AA position | **Sec. str. element** | *Thermodesulfovibrio yellowstonii* sequence | AA position | **Sec. str. element** |
| --- | --- | --- | --- | --- | --- | --- | --- |
| FAD-Binding | 1 | P T E V V E T D I L I I G | 17-29 | beta-sheet | D V V E V E T D F L I I G | 15-27 | beta-sheet |
| **site I** | 2 | F S G C G G A A Y E A A Y W A K | 32-44 | alpha-helix | M S A C G A A Y E A A R W A T | 30-44 | alpha-helix |
| (2-261 AA) | 3 | K V T L V E | 51-56 | beta-sheet | K V T L V D | 49-54 | beta-sheet |
|  | 4 | S A I | 71-73 | beta-sheet | S A I **loop shortened** | 69-71 | beta-sheet |
|  | 5 | L E D Y V R Y V T L D M | 89-100 | alpha-helix | V V D Y V K Y V R A D L | 80-91 | alpha-helix |
|  | 6 | E D L V A D Y A R H V D G T V H L F E K | 106-125 | alpha-helix | E D L V Y D L G R H V D N S V H L F E E W | 97-117 | alpha-helix |
|  | 7 | P I W K T | 129-133 | beta-sheet | P I W K K | 120-124 | beta-sheet |
|  |  |  |  |  | L D G F Q A R D A G K P A L (new) | 131-144 | **alpha-helix** |
|  | 8 | K Y V R E | 137-141 | beta-sheet | V P C R S | 149-153 | beta-sheet |
|  | 9 | Q I M I H | 145-149 | beta-sheet | Q I M I N | 157-161 | beta-sheet |
|  | 10 | Y K P I I A E A A K M A V | 153-165 | alpha-helix | Y K V I V A E A A K A **→shortened** | 165-175 | alpha-helix |
|  |  |  |  |  | **L E F (new)** | 177-179 | **beta-sheet** |
|  |  |  |  |  | **T G Q (new)** | 184-186 | **beta-sheet** |
|  | 11 | N I Y E R V F I F E L L K D | 169-182 | beta-sheet | N I Y E R V F I V K L L K D | 189-202 | beta-sheet |
|  | 12 | A V A G A V G F S V | 188-197 | beta-sheet | R V A G A I G F S V | 208-217 | beta-sheet |
|  | 13 | K F Y V F K A | 201-207 | beta-sheet | K I Y L F K A | 221-227 | beta-sheet |
|  | 14 | A V I L A | 209-213 | beta-sheet | A I L A G | 229-233 | beta-sheet |
|  | 15 | G A T | 216-218 | beta-sheet | G A V | 236-238 | beta-sheet |
|  | 16 | A A | 229-230 | alpha-helix | G Q | 249-250 | alpha-helix |
|  | 17 | D T G | 239-241 | beta- sheet | N S G | 259-261 | beta- sheet |
|  | 18 | S G Y Y M G L K A | 242-250 | alpha-helix | S G Y Y L G M T V | 262-270 | alpha-helix |
|  | 19 | M L T Q | 253-256 | beta-sheet | E M T M | 273-276 | beta-sheet |
| Capping | 20 | P F R F K | 263-267 | beta-sheet | P A R F K | 283-287 | beta-sheet |
| (262-393 AA) | 21 | G A W F L F | 274-279 | alpha-helix | G A W F L F | 294-299 | alpha-helix |
|  | 22 | K A K N A | 283-287 | beta-sheet | K A T N A | 303-307 | beta-sheet |
|  | 23 | I K | 293-294 | alpha-helix | **Y C** A K **alpha-helix elongated** | 312-315 | alpha-helix |
|  | 24 | A | 298 | alpha-helix | K E elongated | 321-322 | alpha-helix |
|  | 25 | Y G | 306-307 | alpha-helix | YG | 327-328 | alpha-helix |
|  | 26 | T P L R N H Q V M L E I M D | 314-327 | alpha-helix | T A I R N H L M M Q S M K Q | 336-349 | alpha-helix |
|  | 27 | P I M Y M H | 331-335 | beta-sheet | P I L M N | 353-357 | beta-sheet |
|  | 28 | T E E A L A E L A | 336-344 | alpha-helix | T H T A M Q E L A | 358-366 | alpha-helix |
|  | 29 | K K K L K H I Y E E A F E D F L | 348-363 | alpha-helix | →K R L K H L E A E A W E D F L **shortened** | 372-386 | alpha-helix |
|  | 30 | S Q A L L W A C Q | 368-375 | alpha-helix | G Q A G L W A A Q | 391-399 | alpha-helix |
|  | 31 | S E A A P A | 385-390 | beta-sheet | S E I M P T | 408-413 | beta-sheet |
| **FAD-Binding** | 32 | G F W | 403-405 | beta-sheet | G F W **loop elongated** | 426-428 | beta-sheet |
| **site II** | 33 | K | 419 | alpha-helix | **missing** |  |  |
| (393-487 AA) | 34 | R M T | 427-429 | beta-sheet | R M S | 448-450 | beta-sheet |
|  | 35 | G L F A I | 433-437 | beta-sheet | G L F M S | 454-458 | beta-sheet |
|  | 36 | S S G S F T E G R I A A K A A V R F I L E Q | 449-470 | alpha-helix | S S G S H A E G R I A A K A A I A F I L D N | 470-491 | alpha-helix |
|  | 37 | D A V V E E L K K K | 478-487 | alpha-helix | **→** D I N A L A A E **shortened** | 501-508 | alpha-helix |
| Helical | 38 | P M E R F M Q | 491-497 | alpha-helix | P F E L Y E K | 512-518 | alpha-helix |
| (488-643 AA) | 39 | P W Q G L V R L Q K I M D E | 514-527 | alpha-helix | P D M Y Q A R L Q K I A D E | 535-548 | alpha-helix |
|  | 40 | Y K T | 536-538 | beta-sheet | Y M T | 557-559 | beta-sheet |
|  | 41 | E K M L L Q R A L E L L A F L K E D L | 540-557 | alpha-helix | K T M I E E G L N K L Q L L K E D A | 561-578 | alpha-helix |
|  | 42 | L H E L M R A W E L V H R V W T A E A H V R H M L F | 565-590 | alpha-helix | L H E L L R C W E N V H R T L S L E A H A R H I L F | 586-611 | alpha-helix |
|  | 43 | Y R T | 601-603 | beta-sheet | Y R G | 621-623 | beta-sheet |
|  | 44 | K C F V C S K Y D | 614-622 | beta-sheet | R A F V N S V Y D | 635-643 | beta-sheet |
|  | 45 | E W T F E K V P Y | 627-635 | beta-sheet | T F T L K K V P Y | 648-656 | beta-sheet |
|  |  |  |  |  |  |  |  |

**Str. deviations:** 6-12 loop **661 AA total** **Protein problems: backbone**

**identical AA 352** 53,3% Cys11, Ala56, Gln186, Ala187, Arg247, Asp288, Ala333, Leu334, Ala371,

75-77 loop **similar AA** 450** 68,1% His421, Pro439, Phe469, Glu500 126-148 alpha-helix **1.0Ǻ RMSD Bb 595 90,0%**

176-186 beta-loop-beta ** incl. identical AA

316-319 loop

- 1. loop

367-370 loop

434-438 loop

441-445 alpha-helix missing

493-499 loop

# Secondary structure element succession AprA *Chlorobaculum tepidum*

| **AprA domains** | **No** | ***Archaeoglobus fulgidus* sequence** | AA position | **Sec. str. element** | *Chlorobaculum tepidum* sequence | AA position | **Sec. str. element** |
| --- | --- | --- | --- | --- | --- | --- | --- |
| FAD-Binding | 1 | P T E V V E T D I L I I G | 17-29 | beta-sheet | E V V Y V D T D I L L I G | 15-27 | beta-sheet |
| **site I** | 2 | F S G C G G A A Y E A A Y W A K | 32-44 | alpha-helix | M A C C G A A Y E A A K W A T | 30-44 | alpha-helix |
| (2-261 AA) | 3 | K V T L V E | 51-56 | beta-sheet | R I T M V D | 49-52 | beta-sheet |
|  | 4 | S A I | 71-73 | beta-sheet | S A I **loop shortened** | 69-71 | beta-sheet |
|  | 5 | L E D Y V R Y V T L D M | 89-100 | alpha-helix | P A D Y V K Y V R A D L | 80-91 | alpha-helix |
|  | 6 | E D L V A D Y A R H V D G T V H L F E K | 106-125 | alpha-helix | E D L V Y D L G R H V D N S V H L F E E W | 97-117 | alpha-helix |
|  | 7 | P I W K T | 129-133 | beta-sheet | P I W K R **→ elongated** | 120-124 | beta-sheet |
|  |  |  |  |  | **D E D G S (new)** | 125-129 | **beta-sheet** |
|  |  |  |  |  | **L T E G G (new)** | 140-144 | **beta-sheet** |
|  | 8 | K Y V R E | 137-141 | beta-sheet | **→** K P V R S **elongated** | 145-149 | beta-sheet |
|  | 9 | Q I M I H | 145-149 | beta-sheet | Q I M I N | 153-157 | beta-sheet |
|  | 10 | Y K P I I A E A A K M A V | 153-165 | alpha-helix | Y K V I V A E A A K K **→shortened** | 161-171 | alpha-helix |
|  |  |  |  |  | **L E Y (new)** | 173-175 | **beta-sheet** |
|  |  |  |  |  | **T G V (new)** | 180-182 | **beta-sheet** |
|  | 11 | N I Y E R V F I F E L L K D | 169-182 | beta-sheet | N L Y E R V F I S E L I H D | 184-198 | beta-sheet |
|  | 12 | A V A G A V G F S V | 188-197 | beta-sheet | K V A G A I G F S V | 204-213 | beta-sheet |
|  | 13 | K F Y V F K A | 201-207 | beta-sheet | K A Y V F T A | 217-223 | beta-sheet |
|  | 14 | A V I L A | 209-213 | beta-sheet | T M L L A | 225-229 | beta-sheet |
|  | 15 | G A T | 216-218 | beta-sheet | G A V | 232-234 | beta-sheet |
|  | 16 | A A | 229-230 | alpha-helix | G Q | 245-246 | alpha-helix |
|  | 17 | D T G | 239-241 | beta- sheet | N A G | 255-257 | beta- sheet |
|  | 18 | S G Y Y M G L K A | 242-250 | alpha-helix | T T Y A L A A Q A | 258-266 | alpha-helix |
|  | 19 | M L T Q | 253-256 | beta-sheet | E L V L | 269-272 | beta-sheet |
| Capping | 20 | P F R F K | 263-267 | beta-sheet | P A R F K | 279-283 | beta-sheet |
| (262-393 AA) | 21 | G A W F L F | 274-279 | alpha-helix | G A W F L F | 290-295 | alpha-helix |
|  | 22 | K A K N A | 283-287 | beta-sheet | K A T N S | 299-303 | beta-sheet |
|  | 23 | I K | 293-294 | alpha-helix | C A | 309-310 | alpha-helix |
|  | 24 | A | 298 | alpha-helix | A | 314 | alpha-helix |
|  | 25 | Y G | 306-307 | alpha-helix | **missing** |  |  |
|  | 26 | T P L R N H Q V M L E I M D | 314-327 | alpha-helix | T A M R N H M M M I D M K A | 332-345 | alpha-helix |
|  | 27 | P I M Y M H | 331-335 | beta-sheet | P I L M R | 349-353 | beta-sheet |
|  | 28 | T E E A L A E L A | 336-344 | alpha-helix | T H E A M A A L A **loop elongated** | 354-362 | alpha-helix |
|  | 29 | K K K L K H I Y E E A F E D F L | 348-363 | alpha-helix | →K Q I K H L E A E A W E D F L **shortened** | 368-382 | alpha-helix |
|  | 30 | S Q A L L W A C Q | 368-375 | alpha-helix | G Q A V V W A G N | 387-395 | alpha-helix |
|  | 31 | S E A A P A | 385-390 | beta-sheet | S E L M P T | 404-409 | beta-sheet |
| **FAD-Binding** | 32 | G F W | 403-405 | beta-sheet | G I W **loop elongated** | 422-424 | beta-sheet |
| **site II** | 33 | K | 419 | alpha-helix | **missing** |  |  |
| (393-487 AA) | 34 | R M T | 427-429 | beta-sheet | R M T | 444-446 | beta-sheet |
|  | 35 | G L F A I | 433-437 | beta-sheet | G L F T A | 450-454 | beta-sheet |
|  | 36 | S S G S F T E G R I A A K A A V R F I L E Q | 449-470 | alpha-helix | S S G S H A E G R I A G K S M T A Y C L D H | 466-487 | alpha-helix |
|  | 37 | D A V V E E L K K K | 478-487 | alpha-helix | →R D V D E V I A E **shortened** | 495-504 | alpha-helix |
| Helical | 38 | P M E R F M Q | 491-497 | alpha-helix | P M E T F A K | 508-514 | alpha-helix |
| (488-643 AA) | 39 | P W Q G L V R L Q K I M D E | 514-527 | alpha-helix | P K M F Q A R L Q K I M D E | 531-544 | alpha-helix |
|  | 40 | Y K T | 536-538 | beta-sheet | Y T T | 553-555 | beta-sheet |
|  | 41 | E K M L L Q R A L E L L A F L K E D L | 540-557 | alpha-helix | K T M L E K G L E H L S L L K E D A | 557-574 | alpha-helix |
|  | 42 | L H E L M R A W E L V H R V W T A E A H V R H M L F | 565-590 | alpha-helix | L H E L M R A W E N Y H R L M A G E A H A R H I L F | 581-607 | alpha-helix |
|  | 43 | Y R T | 601-603 | beta-sheet | F R A | 618-620 | beta-sheet |
|  | 44 | K C F V C S K Y D | 614-622 | beta-sheet | K C F T I S K Y D | 631-639 | beta-sheet |
|  | 45 | E W T F E K V P Y | 627-635 | beta-sheet | E W T L S K R D Y | 644-652 | beta-sheet |
|  |  |  |  |  |  |  |  |

**Str. deviations:** 6-10 loop **658 AA total** **Protein problems: backbone**

75-77 loop **identical AA 355** 54,0% Phe10, Ala56, Ala134, Glu142, Val182, Glu183, Ala243,

**similar AA** 466** 70,8% Asp284, Leu330, Glu363, Pro367, His417, Asp430, Pro435 126-144 beta-loop-beta **1.0Ǻ RMSD Bb 598 91,0%** Phe 465

174-182 beta-loop-beta ** incl. identical AA

324-330 loop

363-366 loop

- 1. loop

436-441 alpha-helix missing

- 1. loop

# Secondary structure element succession AprA *Thiobacillus denitrificans* ATCC25259

| **AprA domains** | **No** | ***Archaeoglobus fulgidus* sequence** | AA position | **Sec. str. element** | *Thiobacillus denitrificans* sequence | AA position | **Sec. str. element** |
| --- | --- | --- | --- | --- | --- | --- | --- |
| FAD-Binding | 1 | P T E V V E T D I L I I G | 17-29 | beta-sheet | E V V Q E D V D V L L I G | 9-21 | beta-sheet |
| **site I** | 2 | F S G C G G A A Y E A A Y W A K | 32-44 | alpha-helix | M A C C G A G Y E I M R W A D **loop elongated** | 24-38 | alpha-helix |
| (2-261 AA) | 3 | K V T L V E | 51-56 | beta-sheet | K I K L V D | 49-54 | beta-sheet |
|  | 4 | S A I | 71-73 | beta-sheet | S A I **loop shortened** | 69-71 | beta-sheet |
|  | 5 | L E D Y V R Y V T L D M | 89-100 | alpha-helix | P A D Y A R M V S N D L →  **shortened** | 81-91 | alpha-helix |
|  | 6 | E D L V A D Y A R H V D G T V H L F E K | 106-125 | alpha-helix | D D L A Y D L G D D S V H L F E E W | 98-105 | alpha-helix |
|  | 7 | P I W K T | 129-133 | beta-sheet | P I W K T | 121-125 | beta-sheet |
|  |  |  |  |  | N G E (new) | 128-130 | **beta-sheet** |
|  |  |  |  |  | L K D (new) | 141-143 | **beta-sheet** |
|  | 8 | K Y V R E | 137-141 | beta-sheet | K P V R S | 146-150 | beta-sheet |
|  | 9 | Q I M I H | 145-149 | beta-sheet | Q I M I N | 154-158 | beta-sheet |
|  | 10 | Y K P I I A E A A K M A V | 153-165 | alpha-helix | Y K W I V A E T K K A L | 162-174 | alpha-helix |
|  | 11 | N I Y E R V F I F E L L K D | 169-182 | beta-sheet | R I E E R I F I V K L V N D | 178-191 | beta-sheet |
|  | 12 | A V A G A V G F S V | 188-197 | beta-sheet | R I A G A V G F S T | 197-206 | beta-sheet |
|  | 13 | K F Y V F K A | 201-207 | beta-sheet | K V V V Y K A | 210-216 | beta-sheet |
|  | 14 | A V I L A | 209-213 | beta-sheet | A I L L A | 218-222 | beta-sheet |
|  | 15 | G A T | 216-218 | beta-sheet | G C V | 225-227 | beta-sheet |
|  | 16 | A A | 229-230 | alpha-helix | G T | 238-239 | alpha-helix |
|  | 17 | D T G | 239-241 | beta- sheet | N A G | 248-250 | beta- sheet |
|  | 18 | S G Y Y M G L K A | 242-250 | alpha-helix | S T Y A M A A E A | 251-259 | alpha-helix |
|  | 19 | M L T Q | 253-256 | beta-sheet | E L T M | 262-265 | beta-sheet |
| Capping | 20 | P F R F K | 263-267 | beta-sheet | P A R F K | 272-276 | beta-sheet |
| (262-393 AA) | 21 | G A W F L F | 274-279 | alpha-helix | G A W F L L | 283-288 | alpha-helix |
|  | 22 | K A K N A | 283-287 | beta-sheet | Q A V N A | 292-296 | beta-sheet |
|  | 23 | I K | 293-294 | alpha-helix | **Y** M Q **elongated** | 301-303 | alpha-helix |
|  | 24 | A | 298 | alpha-helix | E | 307 | alpha-helix |
|  | 25 | Y G | 306-307 | alpha-helix | Y G | 321-322 | alpha-helix |
|  | 26 | T P L R N H Q V M L E I M D | 314-327 | alpha-helix | →C L R N H L M L K E M Q E **shortened** | 324-336 | alpha-helix |
|  | 27 | P I M Y M H | 331-335 | beta-sheet | P I Y M D | 340-344 | beta-sheet |
|  | 28 | T E E A L A E L A | 336-344 | alpha-helix | T V T A L A K L R **loop elongated** | 345-353 | alpha-helix |
|  | 29 | K K K L K H I Y E E A F E D F L | 348-363 | alpha-helix | →R E V K H L E A E A W E D F L **shortened** | 359-373 | alpha-helix |
|  | 30 | S Q A L L W A C Q | 368-375 | alpha-helix | G Q C G I W V G E | 378-386 | alpha-helix |
|  | 31 | S E A A P A | 385-390 | beta-sheet | S E L M P T | 395-400 | beta-sheet |
| **FAD-Binding** | 32 | G F W | 403-405 | beta-sheet | G I W **loop elongated** | 413-415 | beta-sheet |
| **site II** | 33 | K | 419 | alpha-helix | A **H elongated, loop elongated** | 438-439 | alpha-helix |
| (393-487 AA) | 34 | R M T | 427-429 | beta-sheet | S M T | 450-452 | beta-sheet |
|  | 35 | G L F A I | 433-437 | beta-sheet | G L F T A | 456-460 | beta-sheet |
|  | 36 | S S G S F T E G R I A A K A A V R F I L E Q | 449-470 | alpha-helix | S S G S H A E G R M C A K S M V K Y C I D N | 472-493 | alpha-helix |
|  | 37 | D A V V E E L K K K | 478-487 | alpha-helix | **→**T P V E Q L V E E **shortened** | 502-510 | alpha-helix |
| Helical | 38 | P M E R F M Q | 491-497 | alpha-helix | P V R T F L E | 514-520 | alpha-helix |
| (488-643 AA) | 39 | P W Q G L V R L Q K I M D E | 514-527 | alpha-helix | P K M L Q F R L Q K I M D E | 537-550 | alpha-helix |
|  | 40 | Y K T | 536-538 | beta-sheet | Y K T | 559-561 | beta-sheet |
|  | 41 | E K M L L Q R A L E L L A F L K E D L | 540-557 | alpha-helix | A N M L A V A E D K L G M L K E D A | 563-580 | alpha-helix |
|  | 42 | L H E L M R A W E L V H R V W T A E A H V R H M L F | 565-590 | alpha-helix | L H E L L R A W E N Y H R I L T A E A H M K H I Q F | 589-613 | alpha-helix |
|  | 43 | Y R T | 601-603 | beta-sheet | Y R M | 624-626 | beta-sheet |
|  | 44 | K C F V C S K Y D | 614-622 | beta-sheet | H C F V N S V Y D | 637-647 | beta-sheet |
|  | 45 | E W T F E K V P Y | 627-635 | beta-sheet | Q W N C F K R A H |  | beta-sheet |
|  |  |  |  |  |  |  |  |

**Str. deviations:** 3-7 loop **666 AA total** **Protein problems: backbone**

40-47 loop **identical AA 334** 50,2% Pro8, Asp47, Ala56, Gln136, Pro139, Asp277, Pro314, Glu354,

75-77 loop **similar AA** 448** 67,3% Pro358, His408, Ala429, Lys435, Ile436, Tyr448, Phe471

**1.0Ǻ RMS Bb 596 89,5%**

127-145 beta-loop-beta ** incl. identical AA

318-322 loop

- 1. loop
  2. loop
  3. loop-alpha-loop
  4. loop

496-501 loop
