# Supplementary material for: Homology Modeling of Dissimilatory APS Reductases (AprBA) of Sulfur-Oxidizing and Sulfate-Reducing Prokaryotes
Source: PLoS One. 2008 Jan 30;3(1):e1514. doi: 10.1371/journal.pone.0001514 (PMC2211403; doi:10.1371/journal.pone.0001514)
Supplement: Table S4 — (0.18 MB DOC) [file pone.0001514.s008.doc]

**Supplementary data material Table S4.** **FAD-binding site: residues adjacent to the FAD**

**cofactor in AprA models of SRP and SOB**

| Species (no. of AA involved in binding) | **FAD-binding domain** | | | **Capping domain** | |
| --- | --- | --- | --- | --- | --- |
|  |  | Isoalloxazine-binding | AA at a distance of < 4.1Å to FAD |  | AA at a distance of < 4.1Å to FAD |
| ***Archaeoglobus fulgidus***  **(35 AA)** | A2-A261  A394-A487 | Leu70, Asn74, Trp234 | Gly29, Gly30, Gly31, Phe32, Ser33, Val55, Glu56, Lys57, Ser63, Gly64, Ala65, Val66, Leu70, Ala72, Ile73, Asn74, Val174, Phe175, Ile176, Ala213, Thr214, Gly215, Trp234, Tyr235, Ala236, Asp239, Ser242  Ser397, His398, Gly438, Asp439, Phe448, Ser449, Ser452 | A262-A393 | Met365 |
| SRB and rel. SOB |  |  |  |  |  |
| ***Desulfotomaculum reducens***  **(35 AA)** | A2-A243  A382-A472 | Leu58, Asn62, Trp216 | Gly17, Gly18, Gly 19, Met20, Ala21, Val43, Asp44, Lys45, Ser51, Gly52, Ala53, Val54, Leu58, Ala60, Ile61, Asn62, Val162, Phe163, Ile164, Gly198, Met199, Gly200, Trp216, Tyr217, Pro218, Ser221, Ser224  Ser386, His387, Gly423, Asp424, Phe433, Ser434, Ser437 | A244-A382 | **Met354** |
| ***Syntrophobacter fumaroxidans***  **(35 AA)** | A2-A248  A387-A482 | Leu58, Asn62, Trp220 | Gly17, Gly18, Gly 19, Met20, Ser21, Val43, Asp44, Lys45, Ser51, Gly52, Ala53, Val54, Leu58, Ala60, Ile61, Asn62, Val163, Phe164, Ile165, Ala199, Met200, Gly201, Trp220, Tyr221, Pro222, Asn225, Ser228  Ser390, His391, Gly433, Asp434, Phe443, Ser444, Ser447 | A249-A386 | **Met358** |
| Fosws7f8  **(36 AA)** | A2-A245  A384-A477 | Leu58, Asn62, Trp218 | Gly17, Gly18, Gly19, Met20, Ala21, Cys22, Val43, Asp44, Lys45, Ser51, Gly52, Ala53, Val54, Leu58, Ala60, Ile61, Asn62, Val161, Phe162, Leu163, Ala197, Met198, Gly199, Trp218, Tyr219, Pro220, Asn223, Ala226  Ser387, His388, Gly428, Asp429, Phe438, Ser439, Ser442 | A246-A383 | Met355 |
| Fosws39f7 **(36 AA)** | A2-A246  A385-A478 | Leu59, Asn63, Trp219 | Gly18, Gly19, Gly 20, Met21, Ser22, Val44, Asp45, Lys46, Ser52, Gly53, Ala54, Val55, Leu59, Ala61, Leu62?, Asn63, Ile162, Phe163, Ile164, Ala198, Met199, Gly200, Trp219, Tyr220, Pro221, Asn224, Ser227  Ser388, His389, Gly429, Asp430, Phe439, Ser440, Ser443, Gln567 | A247-A384 | Met356 |
| ***Thermodesulfo- bacterium commune***  **(35 AA)** | A2-A278  A412-A508 | Leu70, Asn74, Trp251 | Gly29, Gly30, Gly31, Met32, Ala33, Cys55, Asp56, Lys57, Ser63, Gly64, Ala65, Val66, Leu70, Ala72, Ile73, Asn74, Cys191, Phe192, Ile193, Ala230, Thr231, Gly232, Trp251, Tyr252, Pro253, Asn256, Thr259  Ser415, His416, Gly459, Asp460, Phe469, Ser470, Ser473 | A279-A411 | **Met383** |
| ***Desulfovibrio vulgaris***  **(35 AA)** | A2-A278  A414-A510 | Leu74, Asn78, Trp251 | Gly31, Gly32, Gly 33, Met34, Gly35, Ile59, Asp60, Lys61, Ser67, Gly68, Ala69, Val70, Leu74, Ala76, Ile77, Asn78, Ile191, Phe192, Ile193, Ala230, Cys231, Gly232, Trp251, Tyr252, Pro253, Asn256, Ser259  Ser417, His418, Ala461, Asp462, Phe471, Ser472, Ser475 | A279-A413 | Met385 |
| ***Desulfovibrio desulfuricans***  **(35 AA)** | A2-A276  A412-A508 | Leu72, Asn76, Trp249 | Gly31, Gly32, Gly 33, Met34, Gly35, Val57, Asp58, Lys59, Ser65, Gly66, Ala67, Val68, Leu72, Ala74, Ile75, Asn76, Ile189, Phe190, Ile191, Ala228, Ala229, Gly230, Trp249, Tyr250, Pro251, Asn254, Ser257  Ser415, His416, Ala459, Asp460, Phe469, Ser470, Ser473 | A277-A411 | **Met383** |
| ***Desulfotalea psychrophila***  **(35 AA)** | A2-A273  A418-A515 | Leu72, Asn76, Trp246 | Gly32, Gly33, Gly 34, Met35, Ala36, Val57, Asp58, Lys59, Ser65, Gly66, Ala67, Val68, Leu72, Ala74, Ile75, Asn76, Val186, Phe187, Ile188, Ala225, Cys226, Gly227, Trp246, Tyr247, Pro248, Asn251, Ser254  Ser421, His422, Gly467, Asp468, Phe477, Ser478, Ser481 | A274-A417 | **Met389** |
| ***Desulfobulbus* sp. MLMS1**  **(35 AA)** | A2-A271  A416-A513 | Leu70, Asn74, Trp244 | Gly30, Gly31, Gly 32, Met33, Ala34, Val55, Asp56, Lys57, Ser63, Gly64, Ala65, Val66, Leu70, Ala72, Ile73, Asn74, Ile184, Phe185, Ile186, Ala223, Cys224, Gly225, Trp244, Tyr245, Pro246, Asn249, Ser252  Thr419, His420, Gly464, Asp465, Phe474, Ser475, Ser478 | A272-A415 | **Met387** |
| ***O. algarvensis* Delta 1 symbiont**  **(35 AA)** | A2-A273  A409-A505 | Leu70, Asn74, Trp246 | Gly30, Gly31, Gly 32, Met33, Ala34, Val55, Asp56, Lys57, Ser63, Gly64, Ala65, Val66, Leu70, Ala72, Ile73, Asn74, Val186, Phe187, Ile188, Ala225, Cys226, Gly227, Trp246, Tyr247, Pro248, Asn251, Ser254  Ser412, His413, Gly456 Asp457, Phe466, Ser467, Ser470 | A274-A408 | **Met380** |
| ***Thermodesulfo- vibrio yellowstonii***  **(35 AA)** | A2-A281  A417-A508 | Leu68, Asn72, Trp254 | Gly27, Gly28, Gly29, Met30, Ser31, Val53, Asp54, Lys55, Ser61, Gly62, Ala63, Val64, Leu68, Ala70, Ile71, Asn72, Val194, Phe195, Ile196, Gly233, Cys234, Gly235, Trp254, Tyr255, Pro256, Asn259, Ser262  Ser420, His421, Gly459, Asp460, Phe469, Ser470, Ser473 | A282-A416 | **Met388** |
| ***Chlorobaculum tepidum***  **(36 AA)** | A2-A277  A413-A504 | Leu68, Asn72, Trp250 | Gly27, Gly28, Gly29, Met30, Ala31, Cys32, Val53, Asp54, Lys55, Ser61, Gly62, Ala63, Val64, Leu68, Ala70, Ile71, Asn72, Val190, Phe191, Ile192, Ala229, Cys230, Gly231, Trp250, Tyr251, Pro252, Asn255, Thr258  Ser416, His417, Gly455, Asp456, Phe465, Ser466, Ser469 | A278-A412 | **Met384** |
| ***Thiobacillus denitrificans* 25259**  **(36 AA)** | A2-A270  A404-A510 | Leu68, Asn72, Trp243 | Gly21, Gly22, Gly23, Met24, Ala25, Cys26, Val53, Asp54, Lys55, Ser61, Gly62, Ala63, Val64, Leu68, Ala70, Ile71, Asn72, Ile183, Phe184, Ile185, Ala222, Ala223, Gly224, Trp243, Tyr244, Pro245, Asn248, Ser251  Ser407, His408, Gly461, Asp462, Phe471, Ser472, Ser475 | A271-A403 | Met375 |
| **Crenarch. SRP** |  |  |  |  |  |
| ***Caldivirga maquilingensis***  **(35 AA)** | A2-A244  A373-A474 | Leu56, Asn60, Trp217 | Gly17, Gly18, Gly19, Met20, Ala21, Ala41, Glu42, Lys43, Ser49, Gly50, Ala51, Val52, Leu56, Ala58, Ile59, Asn60, Val157, Met158, Val159, Ala196, Ala197, Gly198, Trp217, Tyr218, Pro219, Ser222, Ser225  Ser376, His377, Gly424, Asp425, Phe434, Ser435, Ser438 | A245-A372 | Met344 |
| ***Pyrobaculum calidifontis***  **(33 AA)** | A2-A247  A375-A466 | Leu59,  -, (Asn63)  Trp220 | Gly19, Gly20, Gly21, Met22, Ala23, Val44, Glu45, Lys46, Ser52, Gly53, Ala54, Val55, Leu59, Ala61, Thr62,  -, Val160, Phe161, Val162, Ala199, Ala200, Gly201, Trp220, Tyr221, Pro222, -, Ser228  Ser378, His379, Gly415, Asp426, Phe425, Ser426, Ser429 | A248-A374 | Met346 |
| SOB Apr lineage I |  |  |  |  |  |
| ***Allochromatium vinosum***  **(35 AA)** | A2-A243  A376-A466 | Leu56, Asn60, Trp216 | Gly17, Ala18, Gly 19, Leu20, Gly21, Ala41, Glu42, Lys43, Ser49, Gly50, Ala51, Val52, Leu56, Ala58, Ile59, Asn60, Ile156, Cys157, Val158, Ala195, Ala196, Gly197, Trp216, Tyr217, Ala218, Ser221, Ser224  Ser379, His380, Gly416, Asp417, Phe426, Ser427, Ser430 | A244-A375 | **Met347** |
| *Thiobacillus den.itrificans* 25259  **(35 AA)** | A2-A243  A376-A466 | Leu56, Asn60, Trp216 | Gly17, Ala18, Gly 19, Leu20, Gly21, Ala41, Glu42, Lys43, Ser49, Gly50, Ala51, Val52, Leu56, Ala58, Ile59, Asn60, Ile156, Cys157, Val158, Gly195, Ala196, Gly197, Trp216, Tyr217, Ala218, Ser221, Ser224  Ser379, His380, Gly416, Asp417, Phe426, Ser427, Ser430 | A244-A375 | **Met347** |
| *Cdt.* Ruthia magnifica  **(35 AA)** | A2-A247  A379-A469 | Leu56, Asn60, Trp219 | Gly17, Ala18, Gly 19, Leu20, Gly21, Ala41, Glu42, Lys43, Ser49, Gly50, Ala51, Val52, Leu56, Ala58, Ile59, Asn60, Ile159 Met160, Val161, Gly198, Ala199, Gly200, Trp219, Tyr220, Ala221, Ser224, Ser227  Ser382, His383, Gly419, Asp420, Phe429, Ser430, Ser433 | A248-A378 | **Met350** |
| *Pelagibacter ubique*  **(35 AA)** | A2-A243  A367-A458 | Leu56, Asn60, Trp216 | Gly17, Gly18, Gly 19, Met20, Ala21, Val41, Glu42, Lys43, Ser49, Gly50, Ala51, Val52, Leu56, Ala58, Ile59, Asn60, Ile156, Met157, Ile158, Ala195, Ala196, Gly197, Trp216, Tyr217, Ala218, Ser221, Ser224  Ser371, His372, Gly408, Asp409, Phe418, Ser419, Ser422 | A244-A366 | **Met339** |
| EBAC2C11  **(35 AA)** | A2-A243  A367-A458 | Leu56, Asn60, Trp216 | Gly17, Gly18, Gly 19, Phe20, Gly21, Val41, Glu42, Lys43, Ser49, Gly50, Ala51, Val52, Leu56, Ala58, Ile59, Asn60, Ile156, Met157, Val158, Ala195, Ala196, Gly197, Trp216, Tyr217, Ala218, Ser221, Ser224  Ser371, His372, Gly408, Asp409, Phe418, Ser419, Ser422 | A244-A366 | **Met339** |
