# Supplementary material for: Homology Modeling of Dissimilatory APS Reductases (AprBA) of Sulfur-Oxidizing and Sulfate-Reducing Prokaryotes
Source: PLoS One. 2008 Jan 30;3(1):e1514. doi: 10.1371/journal.pone.0001514 (PMC2211403; doi:10.1371/journal.pone.0001514)
Supplement: Table S5 — (0.18 MB DOC) [file pone.0001514.s009.doc]

**Supplementary data material Table S5. APS/sulfite-AMP binding site: residues adjacent to the substrates APS/sulfite-AMP in AprA models**

**of SRP and SOB**

| Spezies **(no. of AA involved in substrate binding)** | **FAD-binding domain** | | **Capping domain** | | **AA in a distance of < 5.0Å to**  **(deviations to the *A. fulgidus* AprA template structure are listed and highlighted by red colour)** | | | |
| --- | --- | --- | --- | --- | --- | --- | --- | --- |
|  |  | AA in a distance of < 5.0Å to APS/ sulfite-AMP |  | AA in a distance of < 5.0Å to APS/ sulfite-AMP | Sulfate moiety of APS/ sulfite | Phosphate moiety of APS/ AMP | Ribityl moiety of APS/ AMP | Adenine ring of APS/ AMP |
| ***Archaeoglobus fulgidus*** (22 AA) | A2-A261  A394-A487 | Asn74, Tyr95, Glu141, Trp144, Gln145, Trp234, Phe261  His398, Ser399, His446, Phe448 | A262-A393 | Arg265, Pro272, Val273, Gly274, Ala275, Phe277, Leu278, Pro311, Thr314, Arg317, Met365 | Asn74, Trp234, Arg265, His398,  (Phe261, Thr314, Ser399, Met365) | Arg265, Pro272, Val273, Gly274 | Tyr95, Glu141, Gln145, Gly274, Leu278, Thr314, His446, Phe448 | Gly274, Phe277, Leu278, Pro311, Arg317 |
| SRB and rel. SOB |  |  |  |  |  |  |  |  |
| ***Desulfotomaculum reducens***  **(21 AA)** | A2-A243  A382-A472 | Asn62, Tyr80, Trp129, Gln130, Trp216, Phe243  His387, Ser388, His431, Phe433 | A244-A382 | Arg247, Pro254, Val255, Gly256, Ala257, Phe259, Leu260, Pro293, Ala296, Arg299, Met354 | (Thr314 substituted by Ala296) | Identical to ***A. fulgidus*** | Glu141 is missing unsubstituted  (Thr314 unsubstituted by Ala299) | Identical to ***A. fulgidus*** |
| ***Syntrophobacter fumaroxidans***  **(22 AA)** | A2-A248  A387-A482 | Asn62, Tyr80, Glu126, Trp129, Gln130, Trp220, Phe247  His391, Ser392, His441, Phe443 | A249-A386 | Arg251, Pro258, Val259, Gly260, Ala261, Phe263, Leu264, Pro297, Ala300, Arg303, Met358 | (Thr314 substituted by Ala299) | Identical to ***A. fulgidus*** | Identical to Archglobus fulgidus  (Thr314 unsubstituted by Ala299) | Identical to ***A. fulgidus*** |
| **Fosws7f8**  **(22 AA)** | A2-A245  A384-A477 | Asn62, Tyr80, Glu126, Trp129, Gln130, Trp218, Phe245  His388, Ser389, His436, Phe438 | A246-A383 | Arg249, Pro256, Val257, Gly258, Ala259, Phe261, Leu262, Pro295, Ala298, Arg301, Met355 | Identical to ***A. fulgidus*** | Identical to ***A. fulgidus*** | Identical to ***A. fulgidus*** | Identical to ***A. fulgidus*** |
| **Fosws39f7**  **(22 AA)** | A2-A246  A385-A478 | Asn63, Tyr81, Glu127, Trp130, Gln131, Trp219, Phe246  His389, Ser390, His437, Phe439 | A247-A384 | Arg250, Pro257, Val258, Gly259, Ala260, Phe262, Leu263, Pro296, Ala299, Arg302, Met356 | (Thr314 substituted by Ala299) | Identical to ***A. fulgidus*** | Identical to ***A. fulgidus***  (Thr314 unsubstituted by Ala299) | Identical to ***A. fulgidus*** |
| ***Thermodesulfo- bacterium commune*** (20 AA) | A2-A278  A412-A508 | Asn74, Trp158, Gln159, Trp251, Phe278  His416, Ala417, His467, Phe469 | A279-A411 | Arg282, Pro289, Val290, Gly291, Ala292, Phe294, Leu295, His328, Thr331, Arg334, Met383 | (Ser399 substituted by Ala417) | Identical to ***A. fulgidus*** | Glu141 is missing unsubstituted  Tyr95 is missing unsubstituted | Pro311 is substituted by His328 |
| ***Desulfovibrio vulgaris***  **(20 AA)** | A2-A278  A414-A510 | Asn78, Trp161, Gln162, Trp251, Phe278  His418, Ser419, His469, Phe471 | A279-A413 | Arg282, Pro289, Val290, Gly291, Ala292, Phe294, Leu295, Val330, Thr333, Arg336, Met385 | Identical to ***A. fulgidus*** | Identical to ***A. fulgidus*** | Tyr95, Glu141 are missing unsubstituted | Pro311 is missing unsubstituted  Additional Aa Val330 |
| ***Desulfovibrio desulfuricans***  **(20 AA)** | A2-A276  A412-A508 | Asn76, Trp159, Gln160, Trp249, Phe276  His416, Ser417, His467, Phe469 | A277-A411 | Arg280, Pro287, Val288, Gly289, Ala290, Phe292, Leu293, Val328, Thr331, Arg334, Met383 | Identical to ***A. fulgidus*** | Identical to ***A. fulgidus*** | Tyr95, Glu141 are missing unsubstituted | Pro311 is missing unsubstituted  Additional Aa Val328 |
| ***Desulfotalea psychrophila***  **(20 AA)** | A2-A273  A418-A515 | Asn76, Trp156, Gln157, Trp246, Phe273  His422, Ser423, His475, Phe477 | A274-A417 | Arg277, Pro284, Val285, Gly286, Ala287, Phe289, Leu290, Val325, Thr328, Arg331, Met389 | Identical to ***A. fulgidus*** | Identical to ***A. fulgidus*** | Tyr95, Glu141 are missing unsubstituted | Pro311 is missing unsubstituted  Additional Aa Val325 |
| ***Desulfobulbus* sp. MLMS1**  **(19 AA)** | A2-A271 A416-A513 | Asn62, Trp154, Gln155, Trp244, Phe271  His420, Ser421, His472, Phe474 | A272-A415 | Arg275, Pro282, Val283, Gly284, Ala285, Phe287, Leu288, Thr326, Arg329, Met387 | Identical to ***A. fulgidus*** | Identical to ***A. fulgidus*** | Tyr95, Glu141 are missing unsubstituted | Pro311 is missing unsubstituted |
| ***O. algarvensis* Delta 1 symbiont** (20 AA) | A2-A273  A409-A505 | Asn74, Trp156, Gln157, Trp246, Phe273  His413, Ser414, His464, Phe466 | A274-A408 | Arg277, Pro284, Val285, Gly286, Ala287, Phe289, Leu290, Ile325, Thr328, Arg331, Met380 | Identical to ***A. fulgidus*** | Identical to ***A. fulgidus*** | Tyr95, Glu141 are missing unsubstituted | Pro311 substituted by Ile325 |
| ***Thermodesulfovibrio yellowstonii*** (22 AA) | A2-A281 A417-A508 | Asn72, Tyr86, Trp156, Gln157, Trp254, Phe281  His421, Ala422, His467, Phe469 | A282-A416 | Arg285, Pro292, Val293, Gly294, Ala295, Phe297, Leu298, Glu332, Ala333, Thr336, Arg339, Met388 | (Ser399 substituted by Ala422) | Identical to ***A. fulgidus*** | Glu141 is missing unsubstituted | Pro311 is substituted by additional Aa Glu332 and Ala333 |
| ***Chlorobaculum tepidum***  **(22 AA)** | A2-A277 A413-A504 | Asn72, Tyr86, Trp152, Gln153, Trp250, Phe277  His417, Ala418, His463, Phe465 | A278-A412 | Arg281, Pro288, Val289, Gly290, Ala291, Phe293, Leu294, His328, Leu330, Thr332, Arg335, Met384 | (Ser399 substituted by Ala418) | Identical to ***A. fulgidus*** | Glu141 is missing unsubstituted | Pro311 is substituted by additional Aa His328 and Leu330 |
| ***Thiobacillus denitrificans* 25259**  **(22 A)** | A2-A270 A404-A510 | Asn72, Trp153, Gln154, Trp234, Phe270  His408, Ser409, His469, Phe471 | A271-A403 | Arg274, Pro281, Val282, Gly283, Ala284, Phe286, Leu287, Val320, Pro321, Ala322, Ser323, Arg326, Met375 | (Thr314 substituted by Ser323) | Identical to ***A. fulgidus*** | Tyr95, Glu141 are missing unsubstituted | Pro311 is substituted by additional Aa Val320, Pro321 and Ala322 |
| **Crenarch. SRP** |  |  |  |  |  |  |  |  |
| ***Caldivirga maquilingensis*** (24 AA) | A2-A244  A373-A474 | Asn60, Tyr78, Glu124, Trp127, Gln128, Trp217, Phe244  His377, Ala378, His432, Phe434 | A245-A372 | Val246, Arg248, Pro255, Val256, Gly257, Ala258, Gln260, Leu261, Pro297, Thr299, Thr301, Arg304, Met344 | (Ser399 substituted by Ala378  Additional Aa Val249) | Identical to ***A. fulgidus*** | Identical to Archglobus fulgidus | Phe277 is substituted by Gln260  Additional Aa Thr299 |
| ***Pyrobaculum calidifontis*** (20 AA) | A2-A247  A375-A466 | Tyr82, Trp132, Gln133, Trp220, Leu247  His379, His423, Phe425 | A248-A374 | Val249, Arg251, Pro258, Val259, Gly260, Phe261, Tyr263, Leu264, Pro301, Thr304, Arg307, Met346 | Asn74 is missing (Asn63 is improper placed)  (Ser399 is missing unsubstituted,  Additional Aa Val249) | Identical to ***A. fulgidus*** | Glu141 is missing unsubstituted | Phe277 is substituted by Tyr263 |
| **SOB Apr lineage I** |  |  |  |  |  |  |  |  |
| ***Allochromatium vinosum***  **(24 AA)** | A2-A243 A376-A466 | Asn60, Tyr78, Glu125, Trp128, Gln129, Trp216, Ile243  His380, Ala381, His424, Phe426 | A244-A375 | Leu245, Arg247, Pro254, Val255, Gly256, Ala257, Phe259, Leu260, Glu275, Pro302, Thr305, Arg308, Met347 | Additional Aa Leu245  Phe261 substituted by Ile243  Ser399 substituted by Ala 381 | Identical to ***A. fulgidus*** | Identical to ***A. fulgidus*** | Additional Aa Glu275 |
| *Thiobacillus denitrificans* 25259  **(24 AA)** | A2-A243 A376-A466 | Asn60, Tyr78, Glu125, Trp128, Gln129, Trp216, Ile243  His380, Ala381, His424, Phe426 | A244-A375 | Leu245, Arg247, Pro254, Val255, Gly256, Ala257, Phe259, Leu260, Glu275, Pro302, Thr305, Arg308, Met347 | Additional Aa Leu245  Phe261 substituted by Ile243  Ser399 substituted by Ala 381 | Identical to ***A. fulgidus*** | Identical to ***A. fulgidus*** | Additional Aa Glu275 |
| *Cdt.* Ruthia magnifica  **(24 AA)** | A2-A247 A379-A469 | Asn60, Tyr78, Glu128, Trp131, Gln132, Trp219, Ile246  His383, Ala384, His427, Phe429 | A248-A378 | Leu248, Arg250, Pro257, Val258, Gly259, Ala260, Phe262, Leu263, Glu278, Pro305, Thr308, Arg311, Met350 | Additional Aa Leu278  Phe261 substituted by Ile246  Ser399 substituted by Ala 384 | Identical to ***A. fulgidus*** | Identical to ***A. fulgidus*** | Additional Aa Glu278 |
| *Pelagibacter ubique*  **(22 AA)** | A2-A243 A367-A458 | Asn60, Tyr78, Glu125, Trp128, Gln129, Trp216, Ile243  His372, Ala373, His416, Phe418 | A244-A366 | Leu245, Arg247, Pro254, Val255, Gly256, Ala257, Phe259, Leu260, Thr297, Arg300, Met339 | Additional Aa Leu245  Phe261 substituted by Ile243  Ser399 substituted by Ala 373 | Identical to ***A. fulgidus*** | Identical to ***A. fulgidus*** | Pro311 is missing unsubstituted |
| EBAC2C11  **(23 AA)** | A2-A243 A367-A458 | Asn60, Tyr78, Glu125, Trp128, Gln129, Trp216, Ile243  His372, Ala373, His416, Phe418 | A244-A366 | Leu245, Arg247, Pro254, Val255, Gly256, Ala257, Phe259, Leu260, Pro294, Thr297, Arg300, Met339 | Additional Aa Leu245  Phe261 substituted by Ile243  Ser399 substituted by Ala 371 | Identical to ***A. fulgidus*** | Identical to ***A. fulgidus*** | Identical to ***A. fulgidus*** |
