# Supplementary material for: Homology Modeling of Dissimilatory APS Reductases (AprBA) of Sulfur-Oxidizing and Sulfate-Reducing Prokaryotes
Source: PLoS One. 2008 Jan 30;3(1):e1514. doi: 10.1371/journal.pone.0001514 (PMC2211403; doi:10.1371/journal.pone.0001514)
Supplement: Table S6 — (0.19 MB DOC) [file pone.0001514.s010.doc]

**Supplementary data material Table S6. Structure of the active center at the substrate binding site: residues adjacent to the catalytically**

**active N5 atom of FAD in AprA models of SRP and SOB (distances in Å are listed)**

| Spezies | **FAD-binding domain I** |  |  | **Capping domain** |  |  | **FAD-binding domain II** |  |  | **Helical domain** |  |
| --- | --- | --- | --- | --- | --- | --- | --- | --- | --- | --- | --- |
|  |  | AA adjacent to the N5-FAD atom | distance in Å |  | AA adjacent to the N5-FAD atom | distance in Å |  | AA adjacent to the N5-FAD atom | distance in Å |  | AA adjacent to the N5-FAD atom |
| ***Archaeoglobus fulgidus*** | A2-A261 | Leu 70  Ser 71  Ala 72  Asn 74  Trp 234 | 3.63  7.39  5.77  4.25  5.30 | A262-A393 | Arg 265  Pro 272  Met 365 | 6.31  6.32  3.69 | A394-A487 | His 398  Phe 448 | 4.44  5.63 | A488-A643 | - |
| ***Desulfotomaculum reducens*** | A2-A243 | Leu 58  Ser 59  Ala 60  Asn 62  Trp 216 | 3.69  7.40  5.69  4.23  5.29 | A244-A382 | Arg 247  Pro 254  Met 354 | 6.32  6.28  3.65 | A383-A472 | His 387  Phe 433 | 4.45  5.29 | A473-624 | - |
| ***Syntrophobacter fumaroxidans*** | A2-A248 | Leu 58  Ser 59  Ala 60  Asn 62  Trp 220 | 3.69  7.39  5.70  4.23  5.29 | A249-A386 | Arg 251  Pro 258  Met 358 | 6.32  6.28  3.65 | A387-A482 | His 391  Phe 443 | 4.45  5.62 | A483-A634 | - |
| **Fosws7f8** | A2-A245 | Leu 58  Ser 59  Ala 60  Asn 62  Trp 218 | 3.60  7.39  5.69  4.23  5.29 | A246-A383 | Arg 249  Pro 256  Met 355 | 6.32  6.29  3.65 | A384-A477 | His 388  Phe 438 | 4.46  5.62 | A478-A630 | - |
| **Fosws39f7** | A2-A246 | Leu 59  Ser 60  Ala 61  Asn 63  Trp 219 | 3.69  7.39  5.70  4.23  5.29 | A247-A384 | Arg 250  Pro 257  Met 356 | 6.32  6.29  3.65 | A385-A478 | His 389  Phe 439 | 4.45  5.62 | A479-A634 | - |
| ***Thermodesulfobacterium commune*** | A2-A278 | Leu 70  Ser 71  Ala 72  Asn 74  Trp 251 | 3.68  7.39  5.71  4.23  5.29 | A279-A411 | Arg 282  Pro 289  Met 383 | 6.31  6.28  3.67 | A412-A508 | His 416  Phe 469 | 4.45  5.61 | A509-A664 | - |
| ***Desulfovibrio vulgaris*** | A2-A278 | Leu 74  Ser 75  Ala 76  Asn 78  Trp 251 | 3.72  7.38  5.70  4.23  5.29 | A279-A413 | Arg 282  Pro 289  Met 385 | 6.31  6.28  3.67 | A414-A510 | His 418  Phe 471 | 4.46  5.62 | A511-A664 | - |
| ***Desulfovibrio desulfuricans*** | A2-A276 | Leu 72  Ser 73  Ala 74  Asn 76  Trp 249 | 3.71  7.38  5.70  4.22  5.29 | A277-A411 | Arg 280  Pro 287  Met 383 | 6.32  6.28  3.66 | A412-A508 | His 416  Phe 469 | 4.46  5.61 | A509-A662 | - |
| ***Desulfobulbus* sp.** | A2-A271 | Leu 70  Ser 71  Ala 72  Asn 74  Trp 244 | 3.70  7.39  5.70  4.22  5.29 | A272-A415 | Arg 275  Pro 282  Met 387 | 6.32  6.29  3.65 | A416-A513 | His 420  Phe 474 | 4.46  5.61 | A514-A669 | - |
| ***Desulfotalea psychrophila*** | A2-A273 | Leu 72  Ser 73  Ala 74  Asn 76  Trp 246 | 3.72  7.38  5.70  4.23  5.29 | A274-A417 | Arg 277  Pro 284  Met 389 | 6.31  6.29  3.67 | A418-A515 | His 422  Phe 477 | 4.46  5.62 | A516-A671 | - |
| ***Olavius algarvensis* Delta 1 symbiont** | A2-A273 | Leu 70  Ser 71  Ala 72  Asn 74  Trp 246 | 3.70  7.39  5.70  4.22  5.29 | A274-A408 | Arg 277  Pro 284  Met 380 | 6.32  6.29  3.65 | A409-A505 | His 413  Phe 466 | 4.45  5.61 | A506-A659 | - |
| ***Thermodesulfovibrio yellowstonii*** | A2-A281 | Leu 68  Ser 69  Ala 70  Asn 72  Trp 254 | 3.69  7.39  5.70  4.22  5.29 | A282-A416 | Arg 285  Pro 292  Met 388 | 6.32  6.29  3.65 | A417-A508 | His 421  Phe 469 | 4.45  5.61 | A509-A662 | - |
| ***Chlorobaculum tepidum*** | A2-A277 | Leu 68  Ser 69  Ala 70  Asn 72  Trp 250 | 3.70  7.39  5.70  4.22  5.29 | A278-A412 | Arg 281  Pro 288  Met 384 | 6.32  6.29  3.66 | A413-A504 | His 417  Phe 465 | 4.46  5.61 | A505-A658 | - |
| ***Thiobacillus denitrificans* 25259** | A2-A270 | Leu 68  Ser 69  Ala 70  Asn 72  Trp 243 | 3.71  7.39  5.70  4.22  5.29 | A271-A403 | Arg 274  Pro 281  Met 375 | 6.32  6.29  3.66 | A404-A510 | His 408  Phe 471 | 4.46  5.61 | A511-A666 | - |
| ***Pyrobaculum calidifontis*** | A2-A247 | Leu 59  Ser 60  Ala 61  Trp 220 | 3.73  7.37  5.73  5.29 | A248-A374 | Arg 251  Pro 258  Met 346 | 6.31  6.29  3.67 | A375-A466 | His 379  Phe 425 | 4.46  5.62 | A467-A627 | - |
| ***Caldivirga maquilingensis*** | A2-A244 | Leu 56  Ser 57  Ala 58  Asn 60  Trp 217 | 3.69  7.38  5.71  4.23  5.29 | A245-A372 | Arg 248  Pro 255  Met 344 | 6.30  6.30  3.67 | A373-A474 | His 377  Phe 434 | 4.45  5.62 | A475-A635 | - |
| ***Allochromatium vinosum*** | A2-A243 | Leu 56  **Tyr 57**  Ala 58  Asn 60  Trp 216 | 3.70  7.39  5.70  4.23  5.20 | A244-A375 | Arg 247  Pro 254  Met 347 | 6.30  6.29  3.66 | A376-A466 | His 380  Phe 426 | 4.46  5.60 | A467-A620 | - |
| ***Thiobacillus denitrificans* 25259** | A2-A243 | Leu 56  **Tyr 57**  Ala 58  Asn 60  Trp 216 | 3.70  7.40  5.70  4.23  5.20 | A244-A375 | Arg 247  Pro 254  Met 347 | 6.30  6.29  3.65 | A376-A466 | His 380  Phe 426 | 4.46  5.61 | A467-A622 | - |
| ***Cdt.* Ruthia magnifica** | A2-A247 | Leu 56  **Tyr 57**  Ala 58  Asn 60  Trp 219 | 3.70  7.39  5.70  4.23  5.21 | A248-A378 | Arg 250  Pro 257  Met 350 | 6.30  6.29  3.66 | A379-A469 | His 383  Phe 429 | 4.46  5.61 | A470-A625 | - |
| ***Pelagibacter ubique*** | A2-A243 | Leu 56  **Tyr 57**  Ala 58  Asn 60  Trp 216 | 3.69  7.40  5.70  4.23  5.19 | A244-A366 | Arg 247  Pro 254  Met 339 | 6.30  6.29  3.65 | A367-A458 | His 372  Phe 418 | 4.46  5.60 | A459-A614 | - |
| **EBAC2C11** | A2-A243 | Leu 56  **Tyr 57**  Ala 58  Asn 60  Trp 216 | 3.69  7.40  5.70  4.23  5.20 | A244-A366 | Arg 247  Pro 254  Met 339 | 6.29  6.29  3.65 | A367-A458 | His 372  Phe 418 | 4.46  5.60 | A459-A621 | - |
